# One hundred and one new species of *Trigonopterus* weevils from New Guinea

**DOI:** 10.3897/zookeys.280.3906

**Published:** 2013-03-27

**Authors:** Alexander Riedel, Katayo Sagata, Suriani Surbakti, Rene Tänzler, Michael Balke

**Affiliations:** 1 Museum of Natural History Karlsruhe, Erbprinzenstr. 13, D-76133 Karlsruhe, Germany; 2 Papua New Guinea Institute for Biological research (PNG-IBR), Goroka, Papua New Guinea; 3 Jurusan Biology, FMIPA-Universitas Cendrawasih, Kampus Baru, Jayapura, Papua, Indonesia; 4 Zoological State Collection, Münchhausenstr. 21, D-81247 Munich, Germany; 5 GeoBioCenter, Ludwig-Maximilians-University, Munich, Germany

**Keywords:** Melanesia, integrative taxonomy, turbo-taxonomy, weevils, hyperdiverse, morphology, nuclear DNA, *cox1*, DNA barcoding, Coleoptera, Curculionidae, Cryptorhynchinae

## Abstract

A species discovery and description pipeline to accelerate and improve taxonomy is outlined, relying on concise expert descriptions, combined with DNA sequencing, digital imaging, and automated wiki species page creation from the journal. One hundred and one new species of *Trigonopterus* Fauvel, 1862 are described to demonstrate the feasibility of this approach: *Trigonopterus aeneipennis* **sp. n.**, *Trigonopterus aeneus* **sp. n.**, *Trigonopterus agathis* **sp. n.**, *Trigonopterus agilis* **sp. n.**, *Trigonopterus amplipennis* **sp. n.**, *Trigonopterus ancoruncus* **sp. n.**, *Trigonopterus angulatus* **sp. n.**, *Trigonopterus angustus* **sp. n.**, *Trigonopterus apicalis* **sp. n.**, *Trigonopterus armatus* **sp. n.**, *Trigonopterus ascendens* **sp. n.**, *Trigonopterus augur* **sp. n.**, *Trigonopterus balimensis* **sp. n.**, *Trigonopterus basalis* **sp. n.**, *Trigonopterus conformis* **sp. n.**, *Trigonopterus constrictus* **sp. n.**, *Trigonopterus costatus* **sp. n.**, *Trigonopterus costicollis* **sp. n.**, *Trigonopterus crassicornis* **sp. n.**, *Trigonopterus cuneipennis* **sp. n.**, *Trigonopterus cyclopensis* **sp. n.**, *Trigonopterus dentirostris* **sp. n.**, *Trigonopterus discoidalis* **sp. n.**, *Trigonopterus dromedarius* **sp. n.**, *Trigonopterus durus* **sp. n.**, *Trigonopterus echinus* **sp. n.**, *Trigonopterus edaphus* **sp. n.**, *Trigonopterus eremitus* **sp. n.**, *Trigonopterus euops* **sp. n.**, *Trigonopterus ferrugineus* **sp. n.**, *Trigonopterus fusiformis* **sp. n.**, *Trigonopterus glaber* **sp. n.**, *Trigonopterus gonatoceros* **sp. n.**, *Trigonopterus granum* **sp. n.**, *Trigonopterus helios* **sp. n.**, *Trigonopterus hitoloorum* **sp. n.**, *Trigonopterus imitatus* **sp. n.**, *Trigonopterus inflatus* **sp. n.**, *Trigonopterus insularis* **sp. n.**, *Trigonopterus irregularis* **sp. n.**, *Trigonopterus ixodiformis* **sp. n.**, *Trigonopterus kanawiorum* **sp. n.**, *Trigonopterus katayoi* **sp. n.**, *Trigonopterus koveorum* **sp. n.**, *Trigonopterus kurulu* **sp. n.**, *Trigonopterus lekiorum* **sp. n.**, *Trigonopterus lineatus* **sp. n.**, *Trigonopterus lineellus* **sp. n.**, *Trigonopterus maculatus* **sp. n.**, *Trigonopterus mimicus* **sp. n.**, *Trigonopterus monticola* **sp. n.**, *Trigonopterus montivagus* **sp. n.**, *Trigonopterus moreaorum* **sp. n.**, *Trigonopterus myops* **sp. n.**, *Trigonopterus nangiorum* **sp. n.**, *Trigonopterus nothofagorum* **sp. n.**, *Trigonopterus ovatus* **sp. n.**, *Trigonopterus oviformis* **sp. n.**, *Trigonopterus parumsquamosus* **sp. n.**, *Trigonopterus parvulus* **sp. n.**, *Trigonopterus phoenix* **sp. n.**, *Trigonopterus plicicollis* **sp. n.**, *Trigonopterus politoides* **sp. n.**, *Trigonopterus pseudogranum* **sp. n.**, *Trigonopterus pseudonasutus* **sp. n.**, *Trigonopterus ptolycoides* **sp. n.**, *Trigonopterus punctulatus* **sp. n.**, *Trigonopterus ragaorum* **sp. n.**, *Trigonopterus rhinoceros* **sp. n.**, *Trigonopterus rhomboidalis* **sp. n.**, *Trigonopterus rubiginosus* **sp. n.**, *Trigonopterus rubripennis* **sp. n.**, *Trigonopterus rufibasis* **sp. n.**, *Trigonopterus scabrosus* **sp. n.**, *Trigonopterus scissops* **sp. n.**, *Trigonopterus scharfi* **sp. n.**, *Trigonopterus signicollis* **sp. n.**, *Trigonopterus simulans* **sp. n.**, *Trigonopterus soiorum* **sp. n.**, *T sordidus* **sp. n.**, *Trigonopterus squamirostris* **sp. n.**, *Trigonopterus striatus* **sp. n.**, *Trigonopterus strigatus* **sp. n.**, *Trigonopterus strombosceroides* **sp. n.**, *Trigonopterus subglabratus* **sp. n.**, *Trigonopterus sulcatus* **sp. n.**, *Trigonopterus taenzleri* **sp. n.**, *Trigonopterus talpa* **sp. n.**, *Trigonopterus taurekaorum* **sp. n.**, *Trigonopterus tialeorum* **sp. n.**, *Trigonopterus tibialis* **sp. n.**, *Trigonopterus tridentatus* **sp. n.**, *Trigonopterus uniformis* **sp. n.**, *Trigonopterus variabilis* **sp. n.**, *Trigonopterus velaris* **sp. n.**, *Trigonopterus verrucosus* **sp. n.**, *Trigonopterus violaceus* **sp. n.**, *Trigonopterus viridescens* **sp. n.**, *Trigonopterus wamenaensis* **sp. n.**, *Trigonopterus wariorum* **sp. n.**, *Trigonopterus zygops* **sp. n.**. All new species are authored by the taxonomist-in-charge, Alexander Riedel.

## Introduction

The number of undescribed species on Earth is immense (Scheffers et al. 2012). Large scale studies on morphology, functional biology, community ecology, and phylogeny lead to the discovery of large numbers of new species, but suffer from the lack of a sound taxonomic foundation. DNA barcoding and molecular biodiversity assessment studies do indeed suffer from the same issue. The reason is apparent – it is comparably easy to collect many species and create large datasets, but it is not so easy to identify them, especially if numerous samples from tropical localities are involved. It is easy to obtain hundreds or thousands of DNA sequences or insect samples for a beta diversity study, even for a student project. Identification of samples from moderately to poorly studied regions, and more specifically the recognition and formal scientific description of new species however require taxonomic expertise.

Here, we will not review the significant body of literature addressing the various suggestions how to overcome the “taxonomic impediment”. Rather we report a species discovery and description pipeline (Riedel et al. 2013) that accelerates and improves the way taxonomy flanks research in related disciplines such as biogeography, phylogenetics and not the least community ecology. The term “turbo-taxonomy” was coined for a similar procedure describing 178 new species of parasitic wasps (Butcher et al. 2012) and is discussed below. When faced with a large number of morphologically similar, undescribed species, it is not an option to carry on “business as usual” and prepare very detailed descriptions with an output of only few species per year. Such a strategy will not achieve a sustained success within this century.

The first step is to select a suitable study group (see also Riedel et al. 2013 for a process chart). After an initial taxon screening, we have selected the hyperdiverse weevil genus *Trigonopterus* Fauvel for our research on biodiversity patterns and biogeography across the Indomalayan Archipelago and Melanesia. *Trigonopterus* are flightless weevils placed in the subfamily Cryptorhynchinae of Curculionidae (Alonso-Zarazaga and Lyal 1999). It contains 91 described species ranging from Sumatra to Samoa, and from the Philippines to New Caledonia. To date, 50 species of *Trigonopterus* have formally been described from New Guinea, the center of its diversity. The majority of these species were described from the Papuan peninsula (Faust 1898, 1899) and from the Sattelberg area of the Huon peninsula (Voss 1960), both in present day Papua New Guinea.

We have previously established that *Trigonopterus* are suitable for accelerated taxonomic study combining morphology and the DNA barcoding approach using mitochondrial *cox1* data (Riedel et al. 2010; Tänzler et al. 2012). *Trigonopterus* species were clearly delineated by both molecular data (nuclear as well as mitochondrial sequences) and morphology, and both data sets were fully compatible. These preliminary surveys already resulted in the recognition of 279 *Trigonopterus* species from seven localities across New Guinea. Most of these were undescribed. DNA barcoding is recommended as an identification tool for *Trigonopterus* since the sequence data in a dynamic identification engine represent an efficient substitute for a traditional species-level key. Considering the high proportion of unknown and usually morphologically similar species both traditional dichotomous keys and computer-based interactive keys would be of very limited use. In the following we provide short diagnostic descriptions with photographs of habitus and male genitalia. In keeping these descriptions concise, it is possible to increase the number of species treated dramatically. This study demonstrates that the taxonomy of hyperdiverse groups can be tackled with the combination of DNA-barcoding and taxonomic expertise. Such work does neither proceed at lightning speed, nor can it be fully automated. However, fully embracing technological development, work can be sped up and results are more sustainable. Significant workloads can be trusted to technicians and students, while the taxonomist can focus on the actual comparative taxonomic work.

Some of the historic *Trigonopterus* species from New Guinea were revised by Riedel (2011). Types of all relevant Papuan species have been examined and additional revisions of the previously described species are in preparation. In the following, we concentrate on species which are not closely related to the ones already known to science. A selection of 101 species covering the morphological diversity of Papuan *Trigonopterus* is described below providing a scaffold for ongoing, future work on this genus.

## Materials and methods

This study is based on a selection of 101 out of 279 species recognized by Tänzler et al. (2012). The number of 101 species was chosen as large enough to cover a major portion of diversity and small enough to complete the task within the scheduled time frame in 2012. Species represented only by females were not included in this selection. Care was taken that all major groups are represented, based on our unpublished phylogenetic analysis. Moreover, we describe some clades of closely related species to demonstrate that our technique also works well for these. Four cryptic species (*Trigonopterus granum* sp. n., *Trigonopterus imitatus* sp. n., *Trigonopterus pseudogranum* sp. n., and *Trigonopterus velaris* sp. n.) are here described; they differ only in minor morphological characters but exhibit a marked genetic divergence (9.9–13.9 % uncorrected *p*-distance in our *cox1* fragment). In all, 50 species of *Trigonopterus* were previously known from the Papuan region, and three of these species could be identified with confidence in our full dataset. Many of the other previously described species have type series of mixed species which requires additional taxonomic work. This will be done in the near future after the completion of ongoing field campaigns might reveal fresh specimens for study. Species resembling the historic described species were excluded to avoid the risk of creating synonyms. Therefore, species from the Papuan and the Huon peninsula are somewhat underrepresented here.

Holotypes were selected from the sequenced specimens of Tänzler et al. (2012); their DNA had been extracted nondestructively as described by Riedel et al. (2010) and in our laboratory wiki (http://zsm-entomology.de/wiki/The_Beetle_D_N_A_Lab ). The genitalia of most specimens did not require maceration after DNA-extraction; they could be directly stained with an alcoholic Chlorazol Black solution and stored in glycerol in microvials attached to the pin of the specimens. Genitalia of collection specimens or specimens whose abdominal muscle tissue was not sufficiently digested after DNA extraction were macerated with 10% KOH and rinsed in diluted acetic acid before staining. Illustrations of habitus and genitalia were prepared from holotypes. Finally, type series were supplemented with specimens stored in ethanol and older material from the dry collection. As always the case in paratypes, there is a chance that some of these are incorrectly assigned; this is especially true for specimens without sequence-data as an identification based on external morphological characters is more prone to error than an identification based on a *cox1* sequence (Tänzler et al. 2012). Altogether, the selection of 101 species herein is represented by 4,624 specimens. Type depositories are cited using the following codens:

ARC Alexander Riedel Collection, stored in SMNK, Germany.

MZB LIPI Research Center of Biology, Division of Zoology, Museum Zoologicum Bogoriense, Widyasatwaloka, Cibinong, Indonesia.

NAIC National Agriculture Insect Collection, Kilakila, Port Moresby, Papua New Guinea.

NHMB Naturhistorisches Museum Basel, Switzerland.

NKME Naturkundemuseum Erfurt, Germany.

SMNK Staatliches Museum für Naturkunde, Karlsruhe, Germany.

ZSM Zoologische Staatssammlung, München, Germany.

The methods applied for DNA sequencing and sequence analysis are described by Riedel et al. (2010) and Tänzler et al. (2012).

Morphological descriptions are limited to major diagnostic characters. For example, the aedeagus often bears characters suitable to separate closely related species and is therefore illustrated and briefly described. Tegmen and sternite VIII of males show peculiar characters in some species, but these are usually not species-specific. Therefore, they are omitted from the diagnostic descriptions. Measurements such as length / width ratio of elytra or pronotum are avoided and can be taken from the photographs if needed. Identification of females is difficult and is best done based on *cox1*-sequences. Illustrations of female genitalia would alleviate this situation only marginally and the time required to prepare the relevant illustrations did not appear justified. Negative character states (i.e. the absence of a character) are only mentioned explicitly where it appears appropriate. For example, there are few species with swollen or denticulate epistome. In these cases the character state is described, but for the majority of species with simple epistome it is not mentioned. Common practice would require to state explicitly “epistome simple”. Although formally accurate, in groups comprising hundreds of species this leads to inflated descriptions that distract the reader from the important information by enumerating the absence of rare character states. Except in the case of cryptic species no mention is made of “closely related species”, as their choice is highly subjective. The data provided by the *cox1*-sequences should be sufficient at the moment. At a later stage a phylogeny will be published based on several markers and then suitable subgroups may be formally named as subgenera.

Describing large numbers of new species belonging to the same genus makes the invention of suitable species epithets increasingly difficult. We propose a solution by naming ten species based on family names found in the phonebook of Papua New Guinea.

As proposed by Beutel and Leschen (2005) the terms “mesoventrite” / “metaventrite” are used instead of “mesosternite” / “metasternite”, and “mesanepisternum” / “metanepisternum” instead of “mesepisternum” / “metepisternum”. Descriptions were prepared using a Leica MZ16 dissecting microscope and a fluorescent desk lamp for illumination. Measurements were taken with the help of an ocular grid. The length of the body was measured in dorsal aspect from the elytral apex to the front of the pronotum. Legs were described in an idealized laterally extended position; there is a dorsal / ventral and an anterior / posterior surface. Habitus illustrations were compiled using the Automontage© software (Syncroscopy, Cambridge, UK) with a JVC KY70 camera (JVC Professional Products) adapted to a Leica Z6 APO (Leica Microsystems, Wetzlar, Germany). Photographic illustrations of genitalia were made using the same software / camera combination adapted to a Leica Diaplan, and for this purpose the genitalia were embedded in glycerol gelatin as described by Riedel (2005). Genitalia were photographed with their longitudinal axis somewhat lifted anteriorly, to adequately illustrate structures of the curved down apex. All photographs were enhanced using Adobe Photoshop CS2. However, care was taken not to obscure or alter any features of the specimens illustrated. Sequence data were submitted to the European Molecular Biology Laboratory (EMBL), and the accession numbers are provided under each species e.g. as “(EMBL # FN429236)”.

## Taxonomy

### Trigonopterus

Fauvel, 1862

http://species-id.net/wiki/Trigonopterus\according_to_Riedel_et_al_2013

#### Type-species:

*Trigonopterus insignis* Fauvel, 1862,by monotypy.

#### Diagnosis.

Fully apterous genus of Cryptorhynchinae. Length 1.5–6.0 mm. Rostrum in repose not reaching center of mesocoxa. Scutellum completely absent externally. Mesothoracic receptacle deep, posteriorly closed. Metanepisternum completely absent externally. Elytra with 9 striae (sometimes superficially effaced). Tarsal claws minute. Usually body largely unclothed, without dense vestiture. For additional information see http://species-id.net/wiki/Trigonopterus

### Descriptions of the species

#### 1. Trigonopterus aeneipennis

Riedel sp. n.

urn:lsid:zoobank.org:act:7746E1DE-0AFD-443A-A8D5-FBD6DD369F43

http://species-id.net/wiki/Trigonopterus_aeneipennis

##### Diagnostic description.

**Figure 1.**
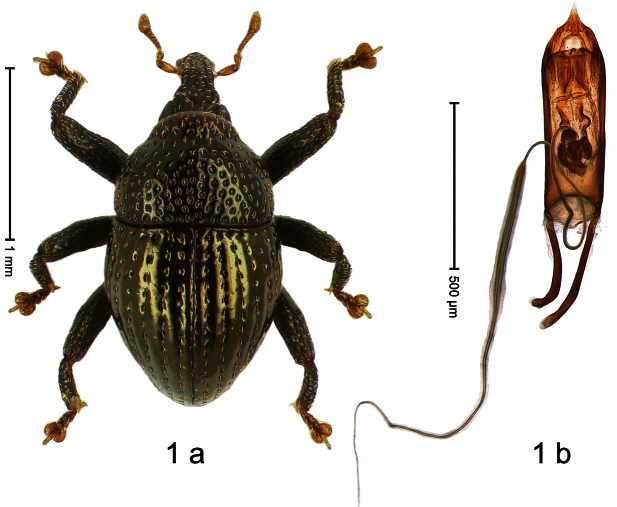
*Trigonopterus aeneipennis* Riedel, sp. n., holotype; (**a**) Habitus (**b**) Aedeagus.

Holotype, male (Fig. 1a). Length 1.74 mm. Color black; elytra and pronotum with greenish-bronze lustre; antenna and tarsi ferruginous. Body subovate; in dorsal aspect and in profile with weak constriction between pronotum and elytron. Rostrum with indistinct, irregular, longitudinal ridges. Pronotum with weak subapical constriction; densely punctate. Elytra with striae deeply impressed, intervals weakly costate, subglabrous. Meso- and metafemur ventrally weakly dentate. Metafemur subapically without stridulatory patch. Aedeagus (Fig. 1b) with apex extended, pointed; body in profile at middle with marked depression; transfer apparatus relatively complex, symmetrical; ductus ejaculatorius with bulbus. **Intraspecific variation**. Length 1.50–1.74 mm. Female rostrum dorsally subglabrous.

##### Material examined.

Holotype (MZB): ARC0530 (EMBL # FN429236), WEST NEW GUINEA, Jayapura Reg., Cyclops Mts, S02°31.912', E140°30.416', 785 m, 02-XII-2007, sifted. Paratypes (SMNK, ZSM): WEST NEW GUINEA, Jayapura Reg., Cyclops Mts, Sentani: 3 exx, ARC0531 (EMBL # FN429237), same data as holotype; 2 exx, ARC0550 (EMBL # FN429256), S02°31.776', E140°30.215', 945 m, 21-XI-2007, sifted; 4 exx, ARC0564 (EMBL # FN429270), S02°31.912', E140°30.416', 785 m, 02-XII-2007, sifted.

##### Distribution.

Jayapura Reg. (Cyclops Mts). Elevation: 785–945 m.

##### Biology.

Sifted from leaf litter in montane forest.

##### Etymology.

This epithet is based on a combination of the Latin adjective *aeneus* (of bronze) and the noun *penna* (wing, elytron) and refers to its coloration.

##### Notes.

*Trigonopterus aeneipennis* Riedel, sp. n. was coded as “*Trigonopterus* sp. 50” by Riedel et al. (2010) and Tänzler et al. (2012), respectively “*Trigonopterus* spax” in the EMBL/GenBank/DDBJ databases.

#### 2. Trigonopterus aeneus

Riedel sp. n.

urn:lsid:zoobank.org:act:469B5A6C-6773-4E35-8F0D-5CF4EE85D658

http://species-id.net/wiki/Trigonopterus_aeneus

##### Diagnostic description.

**Figure 2.**
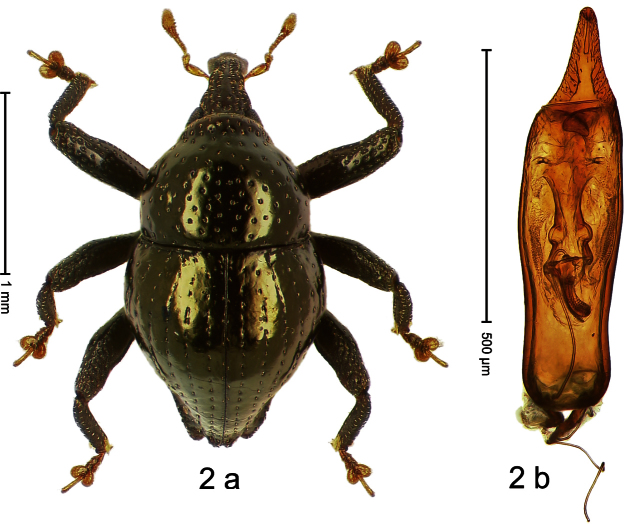
*Trigonopterus aeneus* Riedel, sp. n., holotype; (**a**) Habitus (**b**) Aedeagus.

Holotype, male (Fig. 2a). Length 1.91 mm. Color black with greenish-bronze lustre; antenna and tarsi ferruginous. Body subrhomboid; in dorsal aspect and in profile with distinct constriction between pronotum and elytron. Rostrum with weak median wrinkle; epistome simple. Eyes small. Pronotum with weak subapical constriction; disk sparsely punctate. Elytra with strial punctures distinct; intervals flat, subglabrous; interval 7 subapically costate, forming angulate ridge; sutural interval apically with knob. Meso- and metafemur ventrally weakly dentate. Metafemur subapically without stridulatory patch. Onychium ca. 1.8× longer than tarsomere 3. Aedeagus (Fig. 2b) with apex weakly asymmetrical; long median extension somewhat shifted to the left; transfer apparatus hook-shaped; ductus ejaculatorius with indistinct bulbus, torn off in holotype. **Intraspecific variation**. Length 1.63–1.91 mm. Female rostrum dorsally subglabrous, sparsely punctate.

##### Material examined.

Holotype (SMNK): ARC1089 (EMBL # HE615719), PAPUA NEW GUINEA, Simbu Prov., Karimui Dist., Haia, S06°43.948', E144°59.856', 915 m, 26-IX-2009. Paratypes (NAIC, SMNK, ZSM): PAPUA NEW GUINEA, Simbu Prov.: 12 exx, ARC1090 (EMBL # HE615720), ARC1091 (EMBL # HE615721), same data as holotype; 1 ex, Haia, S06°41.624', E145°00.728', 960 m, 25-IX-2009, sifted; 1 ex, ARC1105 (EMBL # HE615734, Haia, S06°41.018', E145°00.995', 1090 m, 04-X-2009.

##### Distribution.

Simbu Prov. (Haia). Elevation: 915–1090 m.

##### Biology.

Sifted from leaf litter in primary forest.

##### Etymology.

This epithet is based on the Latin adjective *aeneus* (of bronze) and refers to its coloration.

##### Notes.

*Trigonopterus aeneus* Riedel, sp. n. was coded as “*Trigonopterus* sp. 267” by Tänzler et al. (2012).

#### 3. Trigonopterus agathis

Riedel sp. n.

urn:lsid:zoobank.org:act:C893B010-52FB-4D88-8049-5F688480488B

http://species-id.net/wiki/Trigonopterus_agathis

##### Diagnostic description.

**Figure 3.**
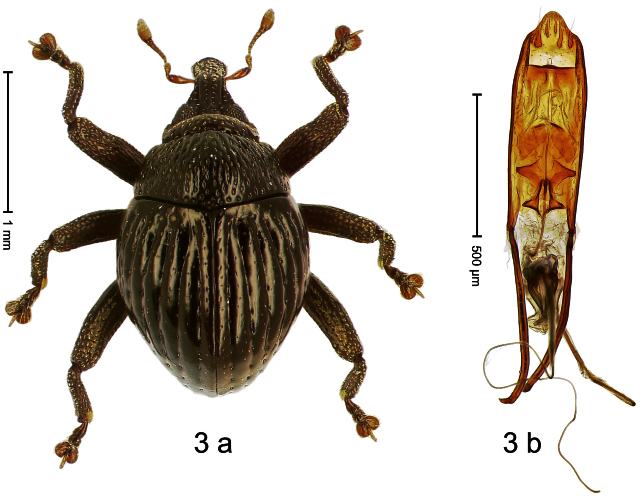
*Trigonopterus agathis* Riedel, sp. n., holotype; (**a**) Habitus (**b**) Aedeagus.

Holotype, male (Fig. 3a). Length 2.04 mm. Color black; legs and antenna ferruginous. Body subglobose; in dorsal aspect with weak constriction between pronotum and elytron; with more distinct constriction in profile. Rostrum in basal half with 3 ridges posteriorly continued to and uniting on forehead; apical half scabrous; epistome smooth, forming indistinct transverse ridge. Pronotum punctate-rugose, interspaces between punctures forming longitudinal wrinkles; with distinct subapical constriction. Elytra subglabrous, striae deeply impressed, intervals costate; apex extended ventrad, beak-shaped. Femora edentate. Metafemur with denticulate dorsoposterior edge, subapically without stridulatory patch. Abdominal venter steeply flexed dorsad, concealed in elytral capsule. Aedeagus (Fig. 3b) with apex medially pointed; body in apical half with broad depression visible in lateral aspect; in basal half with x-shaped sclerite; subglabrous, with sparse indistinct setae; transfer apparatus markedly flagelliform, longer than body, curled, pointing apicad. **Intraspecific variation**. Length 1.86–2.04 mm.

##### Material examined.

Holotype (MZB): ARC1688 (EMBL # HE615975), WEST NEW GUINEA, Jayapura Reg., Cyclops Mts, Angkasa indah, S02°30.346', E140°42.087', 490 m, 28-VI-2010, sifted. Paratypes (SMNK): 4 exx, ARC1689 (EMBL # HE615976), same data as holotype.

##### Distribution.

Jayapura Reg. (Cyclops Mts). Elevation: 490 m.

##### Biology.

Sifted from leaf litter in primary forest.

##### Etymology.

This epithet is based on the Greek noun *agathis* (ball, globe) in apposition and refers to the species' habitus.

##### Notes.

*Trigonopterus agathis* Riedel, sp. n. was coded as “*Trigonopterus* sp. 109” by Tänzler et al. (2012).

#### 4. Trigonopterus agilis

Riedel sp. n.

urn:lsid:zoobank.org:act:5CADDA0B-E91B-4A91-8140-44D3137E69EB

http://species-id.net/wiki/Trigonopterus_agilis

##### Diagnostic description.

**Figure 4.**
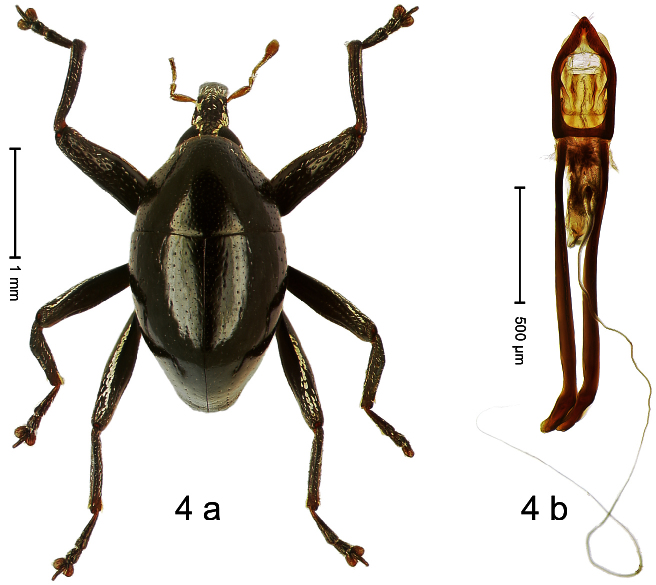
*Trigonopterus agilis* Riedel, sp. n., holotype; (**a**) Habitus (**b**) Aedeagus.

Holotype, male (Fig. 4a). Length 2.55 mm. Color black with slight bluish lustre; legs deep ferruginous, antenna light ferruginous. Body ovate; without constriction between pronotum and elytron; in profile evenly convex. Rostrum dorsally in basal half with median ridge, coarsely punctate and with sparse cream-colored scales; apically smooth, with small punctures. Eyes large. Pronotum dorsally densely punctate with minute punctures; laterally with larger punctures, anteriorly above procoxa squamose with subtriangular cream-colored scales. Elytra dorsally subglabrous, punctures minute, striae hardly visible; laterally strial punctures deep, lateral 5 striae distinct. Femora elongate, edentate. Profemur converging from base to apex. Metafemur with simple dorsoposterior edge; subapically without stridulatory patch. Tibiae simple, without rows or brushes of long setae. Metathoracic and abdominal venter with sparse cream-colored scales. Aedeagus (Fig. 4b) symmetrical, apically pointed; ductus ejaculatorius without bulbus. **Intraspecific variation**. Length 2.54–2.84 mm. Female rostrum dorsally subglabrous, in apical half with minute punctures.

##### Material examined.

Holotype (MZB): ARC0488 (EMBL # FN429195), WEST NEW GUINEA, Jayapura Reg., Cyclops Mts, Sentani, S02°31.7', E140°30.3', 850–1000 m, 30-XI-2007, beaten. Paratypes (ARC, SMNK, ZSM): WEST NEW GUINEA, Jayapura Reg., Cyclops Mts, Sentani: 4 exx, ARC0489 (EMBL # FN429196), ARC0490 (EMBL # FN429197), same data as holotype; 2 exx, S02°31.7', E140°30.3', 860–1150 m, 21-XI-2007, beaten; 3 exx, S02°31.6', E140°30.4', 900–1100 m, 28-XI-2007, beaten; 5 exx, S02°31.6', E140°30.4', 1000–1200 m, 30-XI-2007, beaten; 5 exx, 950–1450 m, 03-X-1992; 1 ex, 1100–1600 m, 05-X-1991; 1 ex, 700–1400 m, 23-XII-2004; 5 exx, 300–1400 m, 10-VIII-1991.

##### Distribution.

Jayapura Reg. (Cyclops Mts). Elevation: 700–1100 m.

##### Biology.

Collected by beating foliage in montane forests.

##### Etymology.

This epithet is based on the Latin adjective *agilis* (quick) and refers to the behavior of this species and its close relatives.

##### Notes.

*Trigonopterus agilis* Riedel, sp. n. was coded as “*Trigonopterus* sp. 14” by Riedel et al. (2010) and Tänzler et al. (2012), respectively “*Trigonopterus* spm” in the EMBL/GenBank/DDBJ databases.

#### 5. Trigonopterus amplipennis

Riedel sp. n.

urn:lsid:zoobank.org:act:7871593A-F435-4CFC-99C5-4A68F2BB589D

http://species-id.net/wiki/Trigonopterus_amplipennis

##### Diagnostic description.

**Figure 5.**
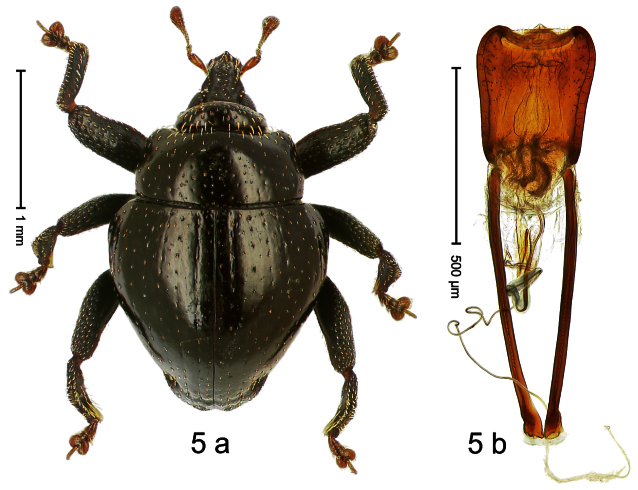
*Trigonopterus amplipennis* Riedel, sp. n., holotype; (**a**) Habitus (**b**) Aedeagus.

Holotype, male (Fig. 5a). Length 2.26 mm. Color black; tarsi and antenna ferruginous. Body subrhomboid; in dorsal aspect with marked constriction between pronotum and elytron; in profile with shallow constriction. Rostrum dorsally dull, with 3 irregular ridges, with rows of erect setae; epistome forming angulate ridge and median denticle. Pronotum with distinct subapical constriction, sparsely punctate with small setiferous punctures, behind subapical constriction scales larger, subclavate, yellowish. Elytra subglabrous, striae weakly impressed, with minute punctures, each puncture with minute seta; apex bordered by curved ridge, median suture incised. Femora edentate. Meso- and metatibia in basal half widened, subapically narrowed. Metafemur with denticulate dorsoposterior edge, subapically without stridulatory patch. Aedeagus (Fig. 5b) widening towards subtruncate, medially pointed apex; transfer apparatus flagelliform, curved, shorter than body; ductus ejaculatorius basally swollen, without bulbus. **Intraspecific variation**. Length 2.00–2.28 mm. Color of elytra and legs black or ferruginous. Female rostrum dorsally punctate-rugose, in apical half without setae; epistome simple.

##### Material examined.

Holotype (MZB): ARC0556 (EMBL # FN429262), WEST NEW GUINEA, Jayapura Reg., Cyclops Mts, Sentani, S02°31.182', E140°30.542', 1510 m, 30-XI-2007, sifted. Paratypes (ARC, SMNK, ZSM): WEST NEW GUINEA, Jayapura Reg., Cyclops Mts, Sentani: 34 exx, ARC0557 (EMBL # FN429263), ARC0558 (EMBL # FN429264), ARC0559 (EMBL # HE615318), same data as holotype; 14 exx, S02°31.281', E140°30.535', 1420 m, 30-XI-2007, sifted; 7 exx (1 marked ARC0094), 1320 m, 23-XII-2004, sifted; 5 exx, 300–1400 m, 10-VIII-1991.

##### Distribution.

Jayapura Reg. (Cyclops Mts). Elevation: 1320–1510 m.

##### Biology.

Sifted from leaf litter in montane forest.

##### Etymology.

This epithet is based on a combination of the Latin adjective *amplus* (wide) and the noun *penna* (wing, elytron) and refers to the basally widened elytra.

##### Notes.

*Trigonopterus amplipennis* Riedel, sp. n. was coded as “*Trigonopterus* sp. 40” by Riedel et al. (2010) and Tänzler et al. (2012), respectively “*Trigonopterus* span” in the EMBL/GenBank/DDBJ databases.

#### 6. Trigonopterus ancoruncus

Riedel sp. n.

urn:lsid:zoobank.org:act:09FC1006-E9A6-41F7-B345-C97D58ECE178

http://species-id.net/wiki/Trigonopterus_ancoruncus

##### Diagnostic description.

**Figure 6.**
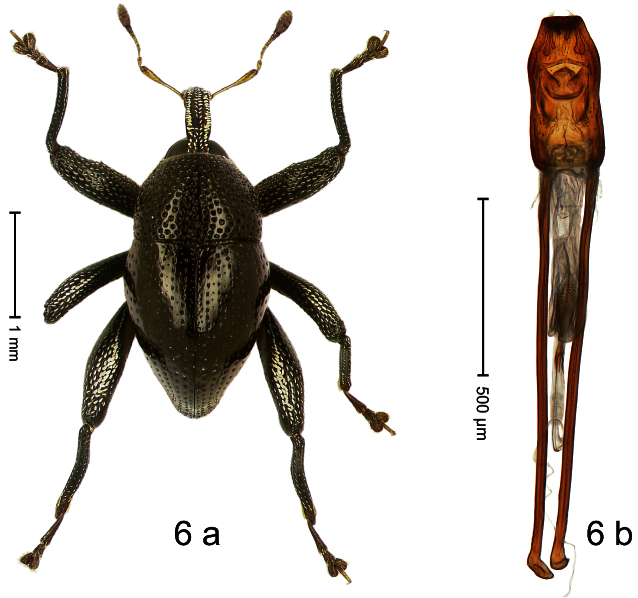
*Trigonopterus ancoruncus* Riedel, sp. n., holotype; (**a**) Habitus (**b**) Aedeagus.

Holotype, male (Fig. 6a). Length 2.49 mm. Color black; antenna and tarsi ferruginous. Body subovate; in dorsal aspect and in profile with distinct constriction between pronotum and elytron. Eyes large. Rostrum medially with two rows of course squamiferous punctures; pair of lateral furrows with row of larger overlapping almond-shaped white scales. Pronotum densely punctate; dorsally punctures containing inconspicuous setae, anterolaterally with scattered white scales. Elytra with striae distinct; intervals flat, subglabrous, each with one row of minute punctures. Femora ventrally with acute tooth. Mesofemur and metafemur dorsally sparsely squamose with white scales. Metafemur subapically with stridulatory patch. Metatibia at middle curved ventrad, subapically with brush of long setae; uncus hook-like extended, curved ventrobasad. Aedeagus (Fig. 6b) with apodemes 2.5 × as long as body; sides of body weakly bisinuate, converging; apex extended, pointed, markedly curved ventrad, sinuate in profile; transfer apparatus flagelliform, subequal to body of aedeagus; ductus ejaculatorius without bulbus.

##### Material examined.

Holotype (SMNK): ARC1115 (EMBL # HE615744), PAPUA NEW GUINEA, Simbu Prov., Karimui Dist., Haia, Supa, S06°40.078', E145°03.207' to S06°39.609', E145°03.012', 1220–1450 m, 02-X-2009.

##### Distribution.

Simbu Prov. (Haia). Elevation: ca. 1220–1450 m.

##### Biology.

Collected by beating foliage in montane forest.

##### Etymology.

This epithet is based on a combination of the Latin nouns *ancora* (anchor) and *uncus* (hook; tibial uncus) in apposition and refers to the species´ remarkable metatibia.

##### Notes.

*Trigonopterus ancoruncus* Riedel, sp. n. was coded as “*Trigonopterus* sp. 78” by Tänzler et al. (2012).

#### 7. Trigonopterus angulatus

Riedel sp. n.

urn:lsid:zoobank.org:act:08E94D6F-0D25-4ABD-AEF0-DA8726D4137E

http://species-id.net/wiki/Trigonopterus_angulatus

##### Diagnostic description.

**Figure 7.**
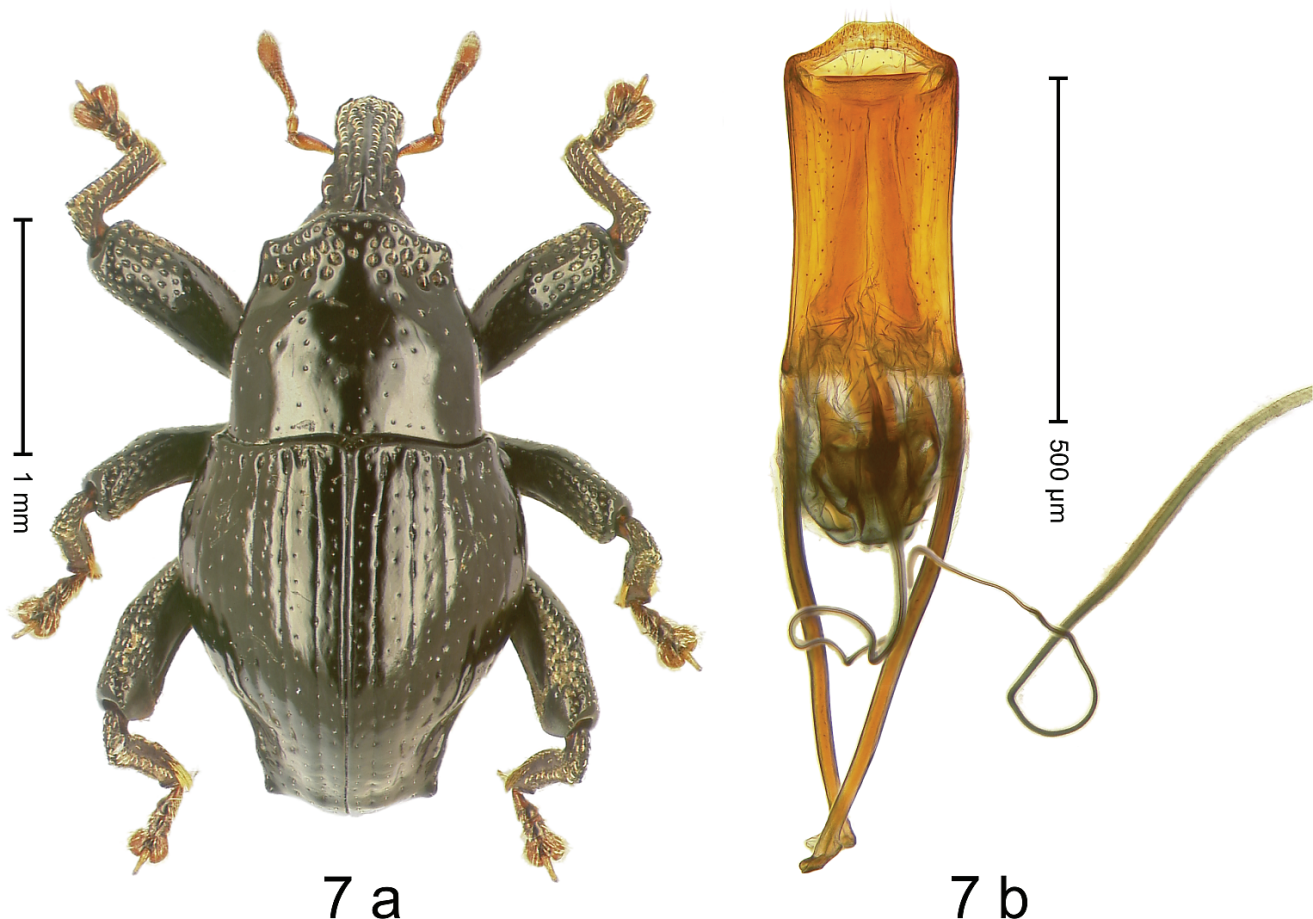
*Trigonopterus angulatus* Riedel, sp. n., holotype; (**a**) Habitus (**b**) Aedeagus.

Holotype, male (Fig. 7a). Length 2.63 mm. Color black; antenna light ferruginous; tarsi and tibiae deep ferruginous. Body subovate-hexa-goniform; in dorsal aspect with shallow constriction between pronotum and elytron; in profile evenly convex. Rostrum in basal half with median and pair of submedian carinae; laterally somewhat flattened; with sparse suberect scales; epistome with transverse, angulate ridge. Pronotum with anterior margin curved dorsad, forming lateral angles; disk subglabrous, anteriorly densely coarsely punctate, towards sides forming edges; laterally in front and behind procoxa with cavity. Elytra subglabrous; intervals flat, with minute punctures; striae weakly incised on disk, towards glabrous sides forming edges; interval 7 subapically forming edge, apex angulate. Femora edentate. Metafemur dorsally squamose with indistinct suberect scales; in apical third without transverse row of setae, subapically with stridulatory patch. Aedeagus (Fig. 7b) apically subangulate, median tip truncate; with complex, symmetrical transfer apparatus; ductus ejaculatorius with indistinct bulbus. **Intraspecific variation**. Length 2.60–2.63 mm. No female specimen available.

##### Material examined.

Holotype (SMNK): ARC1088 (EMBL # HE615718), PAPUA NEW GUINEA, Simbu Prov., Karimui Dist., Haia, S06°41.216', E145°00.945', 965 m, 27-IX-2009, sifted. Paratype (NAIC): 1 ex, ARC1098 (EMBL # HE615727): PAPUA NEW GUINEA, Simbu Prov., Karimui Dist., Haia, S06°40.976', E145°00.979', 1135 m, 27-IX-2009, sifted.

##### Distribution.

Simbu Prov. (Haia). Elevation: 965–1135 m.

##### Biology

. Sifted from leaf litter in primary forest.

##### Etymology.

This epithet is based on the Latin adjective *angulatus* (with angles) and refers to the outline of its body in dorsal aspect.

##### Notes.

*Trigonopterus angulatus* Riedel, sp. n. was coded as “*Trigonopterus* sp. 194” by Tänzler et al. (2012).

#### 8. Trigonopterus angustus

Riedel sp. n.

urn:lsid:zoobank.org:act:0235E0EC-2D2D-4857-9720-33CA745EFB42

http://species-id.net/wiki/Trigonopterus_angustus

##### Diagnostic description.

**Figure 8.**
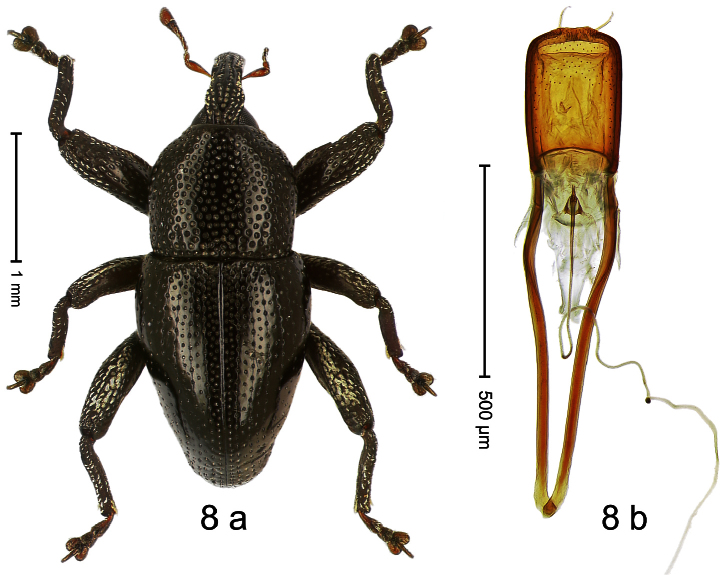
*Trigonopterus angustus* Riedel, sp. n., holotype; (**a**) Habitus (**b**) Aedeagus.

Holotype, male (Fig. 8a). Length 2.83 mm. Color black; legs and antenna ferruginous. Body elongate; in dorsal aspect and in profile with distinct constriction between pronotum and elytron. Rostrum basally with distinct median and pair of submedian carinae, in apical ¼ relatively smooth, basally sparsely squamose. Pronotum densely punctate with large punctures except small glabrous area at center; interspaces smaller than puncture´s diameter. Elytra dorsally punctate with deep punctures; near base and along suture more densely punctate, punctation confuse; basal margin near elytral suture somewhat swollen and glabrous; striae impressed as fine lines; laterally punctation relatively sparse, behind humerus with row of deep punctures. Femora edentate. Profemur in basal third posteriorly with callus. Metafemur subapically without stridulatory patch. Aedeagus (Fig. 8b) apically subangulate, medially truncate, with pair of stout setae; body flattened, sides subparallel; ductus ejaculatorius without bulbus. **Intraspecific variation**. Length 2.24–2.83 mm. Body of females more slender. Female rostrum dorsally subglabrous, punctate, basally sparsely squamose. Female abdominal ventrites 1–2 flat.

##### Material examined.

Holotype (MZB): ARC0626 (EMBL # FN429283), WEST NEW GUINEA, Jayapura Reg., Cyclops Mts, Sentani, S02°32.3', E140°30.4', 350–620 m, 19-XI-2007, beaten. Paratypes (ARC, SMNK, ZSM): WEST NEW GUINEA, Jayapura Reg., Cyclops Mts: 4 exx, Sentani, ARC0455 (EMBL # FN429166), ARC0456 (EMBL # FN429167), ARC0479 (EMBL # FN429186), ARC0631 (EMBL # FN429286), S02°31.3', E140°30.5', 1200–1420 m, 30.XI.2007; 2 exx, ARC0627 (EMBL # FN429284), ARC0630 (EMBL # FN429285), same data as holotype; 1 ex, Cyclops Mts, Angkasa indah ARC1693 (EMBL # HE615980), S02°30.355', E140°42.103' to S02°30.346', E140°42.087', 450–520 m, 28-VI-2010; 5 exx (1 marked as “ARC0046”), 950–1450 m, 03-X-1992; 3 exx, 400–800 m, 07-VIII-1992; 1 ex, 1100–1600 m, 05-X-1991; 2 exx, 300–1400 m, 10-VIII-1991; 6 exx, 1200–1400 m, 09-VIII-1992; 3 exx, Lereh, 500–1000 m, 26-I-1996.

##### Distribution.

Jayapura Reg. (Cyclops Mts; Lereh). Elevation: 520–1200 m.

##### Biology.

Collected by beating foliage in montane forests.

##### Etymology.

This epithet is based on the Latin adjective *angustus* (narrow) and refers to its habitus.

##### Notes.

*Trigonopterus angustus* Riedel, sp. n. was coded as “*Trigonopterus* sp. 12” by Riedel et al. (2010) and Tänzler et al. (2012), respectively “*Trigonopterus* spl” in the EMBL/GenBank/DDBJ databases.

#### 9. Trigonopterus apicalis

Riedel sp. n.

urn:lsid:zoobank.org:act:3071BA70-6556-4D6B-84D0-834C3CB54B99

http://species-id.net/wiki/Trigonopterus_apicalis

##### Diagnostic description.

**Figure 9.**
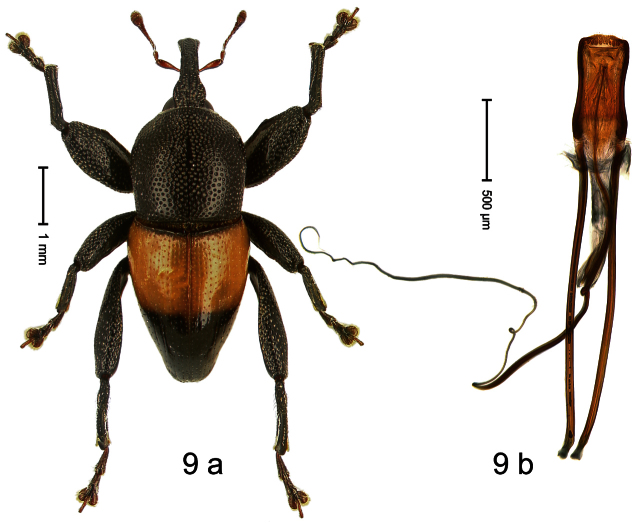
*Trigonopterus apicalis* Riedel, sp. n., holotype; (**a**) Habitus (**b**) Aedeagus.

Holotype, male (Fig. 9a). Length 4.90 mm. Color black except basal half of elytron bright orange; tarsi and antenna ferruginous. Body elongate; in dorsal aspect with distinct constriction between pronotum and elytron. Rostrum slender, basally scabrous with distinct median ridge and pair of irregular submedian ridges, in apical 1/3 punctate. Pronotum large, subquadrate, densely punctate. Elytra densely punctate with small irregular punctures; striae partly impressed as fine lines, partly indistinct. Femora dentate with acute tooth. Profemur enlarged, subovate, posteriorly concave and polished, in basal third with callus. Metafemur subapically with stridulatory patch. Thoracic and abdominal venter concave, densely punctate, sparsely setose with thin erect setae. Aedeagus (Fig. 9b) with apodemes 3.0 × as long as body; apex rounded, at middle subtruncate; transfer apparatus flagelliform, stout, more than 2 × as long as body; ductus ejaculatorius basally swollen, without bulbus. **Intraspecific variation**. Length 4.08–4.90 mm. Female rostrum dorsally subglabrous, sparsely punctate, at base coarsely punctate. Male pronotum larger, with sides posteriorly almost straight; female pronotum smaller, with sides rather convex. Male elytra narrower, converging apicad; female elytra wider, lateral contour convex. Female abdominal venter flat, subglabrous, punctate, with sparse short recumbent setae.

##### Material examined.

Holotype (SMNK): ARC1136 (EMBL # HE615765), PAPUA NEW GUINEA, Simbu Prov., Karimui Dist., Haia, Supa, S06°39.905', E145°03.880' to S06°39.796', E145°03.873', 1220–1320 m, 01-X-2009. Paratypes (NAIC, SMNK, ZSM): Simbu Prov., Karimui Dist., Haia, Supa: 2 exx, ARC1137 (EMBL # HE615766), ARC1138 (EMBL # HE615767), same data as holotype; 1 ex, Haia, Supa station, S06°40.047', E145°03.464' to S06°39.905', E145°03.880', 1075–1220 m, 01-X-2009, beaten; 1 ex, Haia, Supa station, S06°39.815', E145°03.169' to S06°39.609', E145°03.012', 1240–1450 m, 30-IX-2009, beaten.

##### Distribution.

Simbu Prov. (Haia). Elevation: 1220–1240 m.

##### Biology.

Collected by beating foliage in montane forests.

##### Etymology.

This epithet is based on the Latin adjective *apicalis* (pertaining to the apex) and refers to the species´ contrasting elytral coloration.

##### Notes.

*Trigonopterus apicalis* Riedel, sp. n. was coded as “*Trigonopterus* sp. 259” by Tänzler et al. (2012).

#### 10. Trigonopterus armatus

Riedel sp. n.

urn:lsid:zoobank.org:act:FF48AA13-61F6-40DA-A5E3-918590A8212B

http://species-id.net/wiki/Trigonopterus_armatus

##### Diagnostic description.

**Figure 10.**
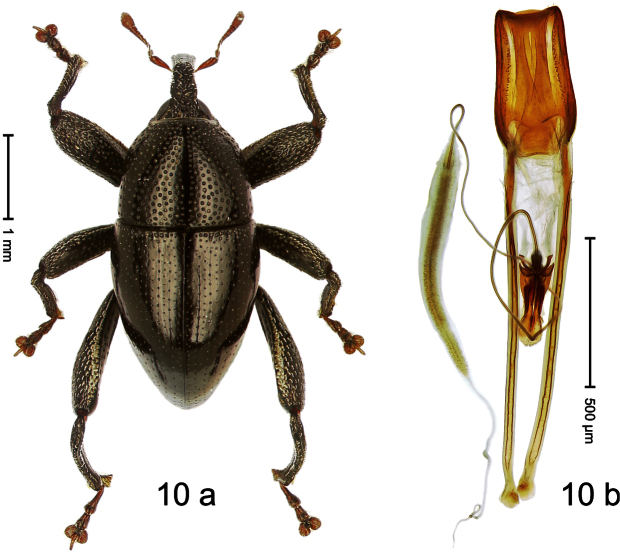
*Trigonopterus armatus* Riedel, sp. n., holotype; (**a**) Habitus (**b**) Aedeagus.

Holotype, male (Fig. 10a). Length 3.53 mm. Color black; tarsi and antenna ferruginous. Body ovate; almost without constriction between pronotum and elytron; in profile evenly convex. Rostrum in basal third swollen, dorsally coarsely punctate and with indistinct median carina; apically shining, punctures more shallow, with longitudinal furrows. Pronotum densely punctate except along impunctate midline. Elytra punctate with small punctures, along basal margin with transverse row of deeper and denser punctures; striae impressed as fine lines; lateral stria behind humeri simple, not deepened. Profemur and mesofemur with anteroventral ridge ending abruptly 1/3 before apex. Metafemur with anteroventral ridge ending with bluntly angled tooth 1/3 before apex; with denticulate dorsoposterior edge; subapically with stridulatory patch. Mesotibia ventrally with spine in subapical 1/3, apically with premucro. Metatibia apically with uncus and larger subtriangular premucro. Aedeagus (Fig. 10b) with distinct, symmetrical transfer-apparatus; ductus ejaculatorius with bulbus. **Intraspecific variation**. Length 2.63–3.59 mm. Female rostrum basally simple, not swollen. Female mesotibia in subapical 1/3 simple, apically without premucro. Female metatibia without premucro.

##### Material examined.

Holotype (MZB): ARC570 (EMBL#FN429273), WEST NEW GUINEA, Jayapura Reg., Cyclops Mts, Sentani, S02°32.0', E140°30.4', 700–900m, 02.XII.2007, beaten. Paratypes (ARC, SMNK, ZSM): WEST NEW GUINEA, Jayapura Reg., Cyclops Mts, Sentani: 9 exx, ARC0464 (EMBL # FN429174), ARC0496 (EMBL # FN429203), ARC0498 (EMBL # FN429205), S02°31.8', E140°30.5', 600–900 m, 28.XI.2007; 12 exx, S02°31.6', E140°30.4', 900–1100 m, 28-XI-2007, beaten; 2 exx, S02°31.6', E140°30.4', 1000–1200 m, 30-XI-2007, beaten; 1 ex, S02°31.7' E140°30.3', 860–1150 m, 21-XI-2007, beaten; 2 exx, S02°32.0', E140°30.4', 700–900 m, 02-XII-2007, beaten; 4 exx, 950–1450 m, 03-X-1992; 1 ex, S02°31.794', E140°30.190', 800–860 m, 21-XI-2007, “Mim2”, beaten; 7 exx, 1100–1600 m, 05-X-1991; 1 ex, 1200–1400 m, 09-VIII-1992; 3 exx, 300–1400 m, 10-VIII-1991; 5 exx, 800–1000 m, 07-VIII-1992; 4 exx, 950–1450 m, 03-X-1992.

##### Distribution.

Jayapura Reg. (Cyclops Mts). Elevation: 860–1200 m.

##### Biology.

Collected by beating foliage in montane forests.

##### Etymology.

This epithet is based on the Latin participle *armatus* (armed) and refers to the teeth of the male meso- and metatibia.

##### Notes.

*Trigonopterus armatus* Riedel, sp. n. was coded as “*Trigonopterus* sp. 7” by Riedel et al. (2010), respectively “*Trigonopterus* spg” in the EMBL/GenBank/DDBJ databases.

#### 11. Trigonopterus ascendens

Riedel sp. n.

urn:lsid:zoobank.org:act:9362791A-D087-4D72-80AE-2D076BAFEF02

http://species-id.net/wiki/Trigonopterus_ascendens

##### Diagnostic description.

**Figure 11.**
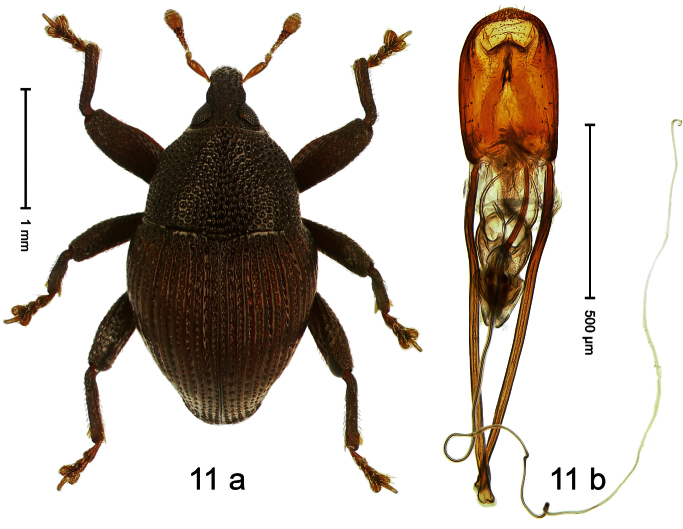
*Trigonopterus ascendens* Riedel, sp. n., holotype; (**a**) Habitus (**b**) Aedeagus.

Holotype, male (Fig. 11a). Length 2.55 mm. Color ferruginous; dorsal surface of head and pronotum black. Body subovate; in dorsal aspect with distinct constriction between pronotum and elytron; in profile with weak constriction. Rostrum densely punctate-reticulate, without longitudinal furrows or ridges. Eyes large, approximate. Pronotum coarsely punctate-reticulate. Elytra densely striate-punctate; striae deeply impressed; intervals each with dense row of deeply impressed punctures, similar to striae; interspaces costate, subglabrous. Femora edentate. Metafemur subapically with stridulatory patch. Aedeagus (Fig. 11b) apically subangulate, subglabrous; transfer apparatus spiniform, long, subequal to body; ductus ejaculatorius without bulbus. **Intraspecific variation**. Length 2.52–2.64 mm. Female rostrum dorsally in apical half with punctures usually isolated.

##### Material examined.

Holotype (MZB): ARC1767 (EMBL # HE616044), WEST NEW GUINEA, Jayawijaya Reg., Poga, S03°47.575', E138°33.155' to S03°47.473', E138°33.163', 2620–2715 m, 15-VII-2010. Paratypes (SMNK, ZSM): 21 exx, ARC1768 (EMBL # HE616045), ARC1769 (EMBL # HE616046), same data as holotype.

##### Distribution.

Jayawijaya Reg. (Poga). Elevation: ca. 2620–2715 m.

##### Biology.

Beaten from foliage of upper montane forests.

##### Etymology.

This epithet is based on the Latin participle *ascendens* (climbing up) and refers to its occurrence on higher elevations.

##### Notes.

*Trigonopterus ascendens* Riedel, sp. n. was coded as “*Trigonopterus* sp. 169” by Tänzler et al. (2012).

#### 12. Trigonopterus augur

Riedel sp. n.

urn:lsid:zoobank.org:act:215ECCF4-792A-4809-BC55-2E702B970739

http://species-id.net/wiki/Trigonopterus_augur

##### Diagnostic description.

**Figure 12.**
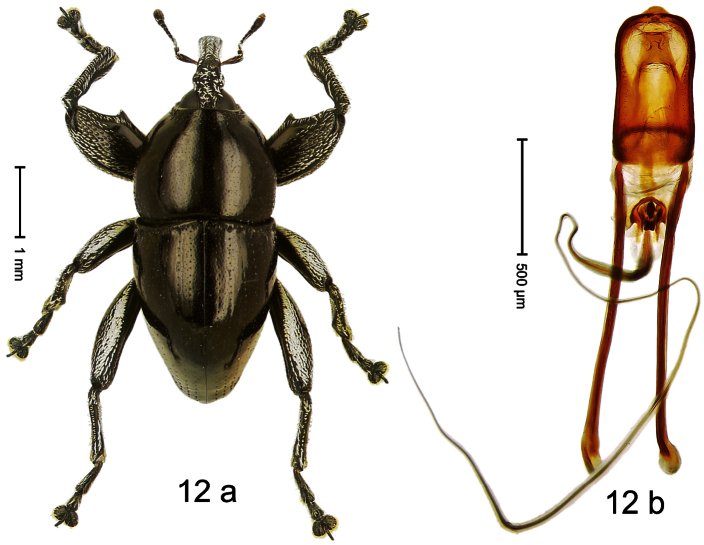
*Trigonopterus augur* Riedel, sp. n., holotype; (**a**) Habitus (**b**) Aedeagus.

Holotype, male (Fig. 12a). Length 4.24 mm. Color black; antenna partly ferruginous. Body ovate; in dorsal aspect and in profile with constriction between pronotum and elytron. Rostrum slender, dorsally with distinct median carina and sublateral ridges; furrows bordering ridges containing each one row of mesad directed white narrow scales; subapically shining, punctate, setose. Eyes large. Pronotum large, subglabrous, with minute punctures, in basal half sides separated by densely punctate edge. Elytra punctate with minute punctures; striae impressed as fine lines; basal margin bisinuate, bordered by row of large punctures continued behind humeri. Profemur large, anteriorly at middle with large tooth. Mesofemur and metafemur dorsally densely squamose with white scales; with anteroventral ridge at middle with inconspicuous tooth. Metafemur with smooth dorsoposterior edge; subapically without stridulatory patch. Aedeagus (Fig. 12b) apically weakly pointed; ductus ejaculatorius near insertion to transfer apparatus swollen, subapically with weak bulbus. **Intraspecific variation**. Length 3.28–4.16 mm. Female rostrum dorsally largely subglabrous, with submedian rows of minute punctures; in basal 1/5 punctate-rugose, with white recumbent scales.

##### Material examined.

Holotype (MZB): ARC0444 (EMBL # FN429155), WEST NEW GUINEA, Jayapura Reg., Cyclops Mts, Sentani, S02°31.685', E140°30.430', 1010m, 28-XI-2007, beaten. Paratypes (ARC, NHMB, SMNK, ZSM): WEST NEW GUINEA, Jayapura Reg., Cyclops Mts, Sentani: 1 ex, ARC0445 (EMBL # FN429156), same data as holotype; 1 ex, ARC0658 (EMBL # FN429305), S02°31.794', E140°30.190', 800–860 m, 21-XI-2007, “Mim2”, beaten; 16 exx, ARC0666 (EMBL # FN429313), ARC0667 (EMBL # FN429314), S02°31.6', E140°30.4', 900–1100 m, 28-XI-2007, beaten; 3 exx, 1100–1600 m, 05-X-1991; 1 ex, 300–1400 m, 10-VIII-1991; 1 ex, 800–1000 m, 07-VIII-1992; 6 exx (1 marked as “ARC0130”), 950–1450 m, 03-X-1992; 2 exx, Lake Sentani, III-1992, 300 m.

##### Distribution.

Jayapura Reg. (Cyclops Mts). Elevation: 300–1200 m.

##### Biology.

Collected by beating foliage in montane forest.

##### Etymology.

This epithet is based on the Latin noun *augur* in apposition and refers to the large eyes that help the species to see birds, presumably important predators.

##### Notes.

*Trigonopterus augur* Riedel, sp. n. was coded as “*Trigonopterus* sp. 21” by Riedel et al. (2010) and Tänzler et al. (2012), respectively “*Trigonopterus* spu” in the EMBL/GenBank/DDBJ databases.

#### 13. Trigonopterus balimensis

Riedel sp. n.

urn:lsid:zoobank.org:act:3700C962-57D1-4014-87E8-3C0F2FCD6E7D

http://species-id.net/wiki/Trigonopterus_balimensis

##### Diagnostic description.

**Figure 13.**
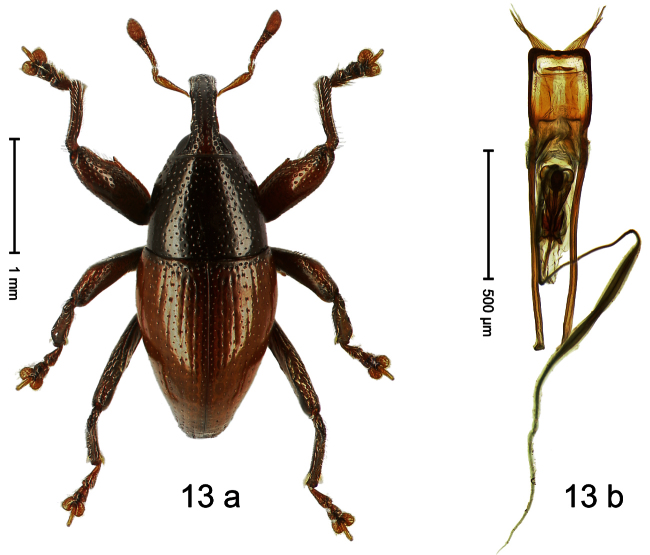
*Trigonopterus balimensis* Riedel, sp. n., holotype; (**a**) Habitus (**b**) Aedeagus.

Holotype, male (Fig. 13a). Length 2.58 mm. Color orange-ferruginous; pronotum and parts of head black. Body fusiform, almost without constriction between pronotum and elytron; in profile dorsally flat, towards apex convex. Rostrum with median costa flat, pair of submedian furrows containing sparse rows of mesad-directed setae. Pronotum subglabrous, punctate with small to minute punctures. Elytra subglabrous, punctation confused with small to minute punctures. Femora with anteroventral ridge terminating with tooth in apical third. Anteroventral ridge of mesofemur high, at middle with subangulate incision. Metafemur with crenulate dorsoposterior edge; subapically with stridulatory patch; posteroventral ridge at middle with knob. Mesotibia with dorsal edge pushed forward, apex slightly curved ventrad. Abdominal ventrite 1 besides metacoxa with brush of erect scales. Aedeagus (Fig. 13b) with body subrectangular; apex with pair of long pointed brushes; transfer-apparatus complex, symmetrical; ductus ejaculatorius with bulbus. **Intraspecific variation**. Length 2.11–2.96 mm. Female mesofemur with evenly denticulate anteroventral ridge, without incision at middle. Posteroventral ridge of female metafemur simple. Female mesotibia straight. Abdominal ventrite 1 besides metacoxa simple.

##### Material examined.

Holotype (MZB): ARC0752 (EMBL # HE615435), WEST NEW GUINEA, Jayawijaya Reg., Jiwika, Kurulu, S03°57.043', E138°57.410', 1920–1950 m, “Mim 3”, 26-XI-2007. Paratypes (ARC, SMNK, NHMB, ZSM): WEST NEW GUINEA, Jayawijaya Reg.: 45 exx, ARC0753 (EMBL # HE615436), ARC0754 (EMBL # HE615437), same data as holotype; 34 exx, Jiwika, Kurulu, S03°56.146', E138°57.710', 2245–2290 m, “Mim 4”, 26-XI-2007; 35 exx, Jiwika, Kurulu, S03°56.5', E138°57.1', 1900–2245 m, 26-XI-2007; 45 exx, Jiwika, Kurulu, S03°57.161', E138°57.357', 1875 m, 11-VII-2010, sifted; 135 exx, Jiwika, Kurulu, S03°57.161', E138°57.357' to S03°56.977', E138°57.441', 1875–1990 m, 12-VII-2010; 2 exx, ARC1711 (EMBL # HE615991), ARC1712 (EMBL # HE615992), Jiwika, Kurulu, S03°57.161', E138°57.357' to S03°56.977', E138°57.441', 1875–1990 m, 12-VII-2010; 73 exx (1 marked ARC0034), Jiwika, Kurulu, 1800–2300 m, 31-V-1998; 20 exx, Jiwika, Kurulu, 1700–2300 m, 02-IX-1991; 9 exx, Jiwika, Kurulu, 1700–2300 m, 06-IX-1991; 15 exx, Jiwika, 1750–2100 m, 05-VII-1994; 18 exx, Jiwika, Kurulu, trail to Wandanku, 2240–2420 m, 28-IX-1996; 17 exx, Jiwika, Kurulu, trail to Wandanku, 1900–2150 m, 28-29-IX-1996; 19 exx, Jiwika, Kurulu, 1900–2300 m, 29-IX-1992; 6 exx, Jiwika, 1700–2100 m, 05-XII-1995; 14 exx, Jiwika, 1700–2000 m, 11-IX-1991; 4 exx, Jiwika, trail to Wandanku, 2240–2420 m, 28-IX-1996; 1 ex, Jiwika, 2300 m, 1992; 69 exx, Baliem-vall., ca. 1700 m, III-1992.

##### Distribution.

Jayawijaya Reg. (Jiwika). Elevation: 1875–2240 m.

##### Biology.

Beaten from foliage of montane forests.

##### Etymology.

This epithet is based on the name of the Balim-river area which is close to the type locality.

##### Notes.

*Trigonopterus balimensis* Riedel, sp. n. was coded as “*Trigonopterus* sp. 172” by Tänzler et al. (2012). At the time, it was lumped with a closely related but distinct species from Poga and Lake Habbema.

#### 14. Trigonopterus basalis

Riedel sp. n.

urn:lsid:zoobank.org:act:72AD61FF-7493-4F5F-953F-A5A5F47C4A50

http://species-id.net/wiki/Trigonopterus_basalis

##### Diagnostic description.

**Figure 14.**
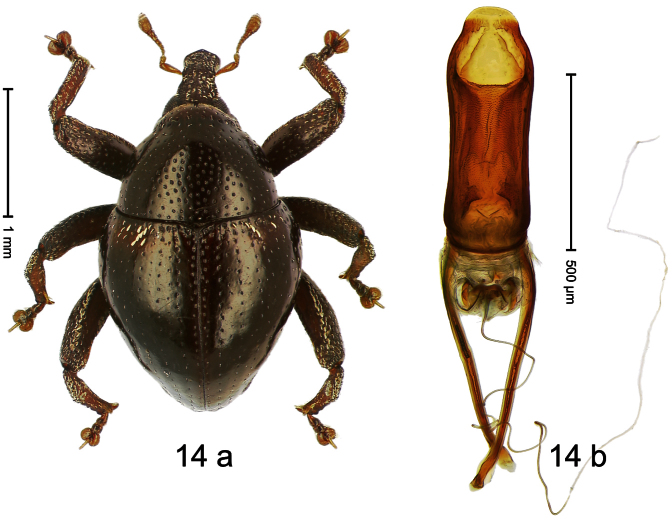
*Trigonopterus basalis* Riedel, sp. n., holotype; (**a**) Habitus (**b**) Aedeagus.

Holotype, male (Fig. 14a). Length 2.42 mm. Color black; base of elytra and legs ferruginous; antenna light ferruginous. Body subovate; in dorsal aspect with marked constriction between pronotum and elytron; in profile almost evenly convex. Rostrum dorsally scabrous, basally with indistinct median ridge; epistome forming angulate ridge. Pronotum moderately densely punctate. Elytra with striae marked by small punctures; interval 4 basally with cluster of few subovate, cream-colored, recumbent scales; interval 7 subapically forming indistinct ridge. Meso- and metafemur with anteroventral ridge weakly dentate. Meso- and metatibia in basal half widened, subapically narrowed; dorsal edge basally granulate; uncus large, peg-shaped. Metafemur with denticulate dorsoposterior edge, subapically without stridulatory patch. Aedeagus (Fig. 14b) with sides of body in apical third converging, apex rounded; orifice retracted; endophallus denticulate; transfer apparatus symmetrical; ductus ejaculatorius without bulbus. **Intraspecific variation**. Length 1.98–2.48 mm. Female rostrum dorsally subglabrous in apical half, with small punctures, epistome simple.

##### Material examined.

Holotype (MZB): ARC0523 (EMBL # FN429229), WEST NEW GUINEA, Jayapura Reg., Cyclops Mts, Sentani, S02°31.912', E140°30.416', 785 m, 02-XII-2007, sifted. Paratypes (SMNK, ZSM): WEST NEW GUINEA, Jayapura Reg., Cyclops Mts: 4 exx, ARC0524 (EMBL # FN429230), same data as holotype; 16 exx, ARC0532 (EMBL # FN429238), Doyo, S02°32.478', E140°28.835', 365 m, 27-XI-2007, sifted; 2 exx, Sentani, S02°32.031', E140°30.412', 710 m, 02-XII-2007, sifted; 5 exx (1 marked “ARC0105”), Sentani, 600 m, 22-XII-2004, sifted; 1 ex, Sentani, 700 m, 22-XII-2004, sifted; 17 exx, Sentani, S02°32.221', E140°30.526', 575 m, 19-XI-2007, sifted; 1 ex, Sentani, S02°32.166' E140°30.512', 620 m, 19-XI-2007, sifted; 3 exx, Sentani, S02°32.291', E140°30.505', 515 m, 19-XI-2007, sifted.

##### Distribution.

Jayapura Reg. (Cyclops Mts). Elevation: 365–785 m.

##### Biology.

Sifted from leaf litter in primary forest.

##### Etymology.

This epithet is based on the Latin adjective *basalis* (characterized by the base) and refers to the elytral base differing in color and vestiture from the remainder.

##### Notes.

*Trigonopterus basalis* Riedel, sp. n. was coded as “*Trigonopterus* sp. 42” by Riedel et al. (2010) and Tänzler et al. (2012), respectively “*Trigonopterus* spap” in the EMBL/GenBank/DDBJ databases.

#### 15. Trigonopterus conformis

Riedel sp. n.

urn:lsid:zoobank.org:act:D8E785F7-96B2-4427-A91B-DF21B3743994

http://species-id.net/wiki/Trigonopterus_conformis

##### Diagnostic description.

**Figure 15.**
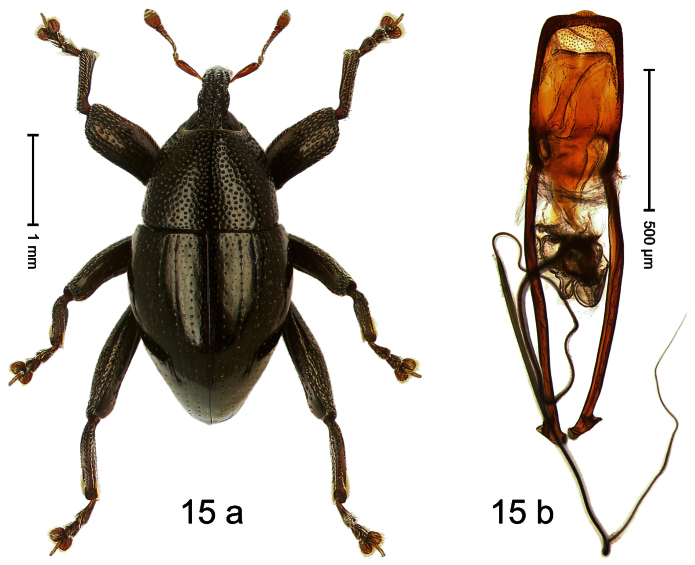
*Trigonopterus conformis* Riedel, sp. n., holotype; (**a**) Habitus (**b**) Aedeagus.

Holotype, male (Fig. 15a). Length 3.34 mm. Color black, legs deep ferruginous, antenna lighter ferruginous. Body ovate; almost without constriction between pronotum and elytron; in profile evenly convex. Rostrum dorsally tricarinate, with distinct median and pair of lateral carinae. Pronotum densely punctate. Elytra densely punctate; strial punctures slightly larger than minute punctures on intervals; striae impressed as fine lines; lateral stria behind humeri simple, not deepened. Femora edentate. Metafemur with denticulate dorsoposterior edge; subapically with stridulatory patch. Mesotibia simple, in basal half dorsal contour denticulate, but without distinct angulation. Metatibia with minute premucro; without suprauncal projection. Aedeagus (Fig. 15b) apically subangulate; dorsum sublaterally sparsely setose; transfer apparatus dentiform, short; ductus ejaculatorius with bulbus. **Intraspecific variation**. Length 3.31–3.34 mm. No female specimen available.

##### Material examined.

Holotype (MZB): ARC1794 (EMBL # HE616071), WEST NEW GUINEA, Jayawijaya Reg., Bokondini, S03°41.787', E138°40.229' to S03°41.778', E138°40.129', 1705–1710 m, 17-VII-2010. Paratype (SMNK): 3 exx, ARC1795 (EMBL # HE616072), ARC2347 (EMBL # HF548203), ARC2348 (EMBL # HF548204) same data as holotype.

##### Distribution.

Jayawijaya Reg. (Bokondini). Elevation: ca. 1705–1710 m.

##### Biology.

Beaten from foliage of montane forest.

##### Etymology.

This epithet is based on the Latin adjective *conformis* (like, similar) and refers to the similarity of this species, both to some closely related sibling species, and to others of only superficial resemblance.

##### Notes.

*Trigonopterus conformis* Riedel, sp. n. was coded as “*Trigonopterus* sp. 86” by Tänzler et al. (2012).

#### 16. Trigonopterus constrictus

Riedel sp. n.

urn:lsid:zoobank.org:act:DB8A37EA-E484-49ED-9D6A-4EE6AF3473C5

http://species-id.net/wiki/Trigonopterus_constrictus

##### Diagnostic description.

**Figure 16.**
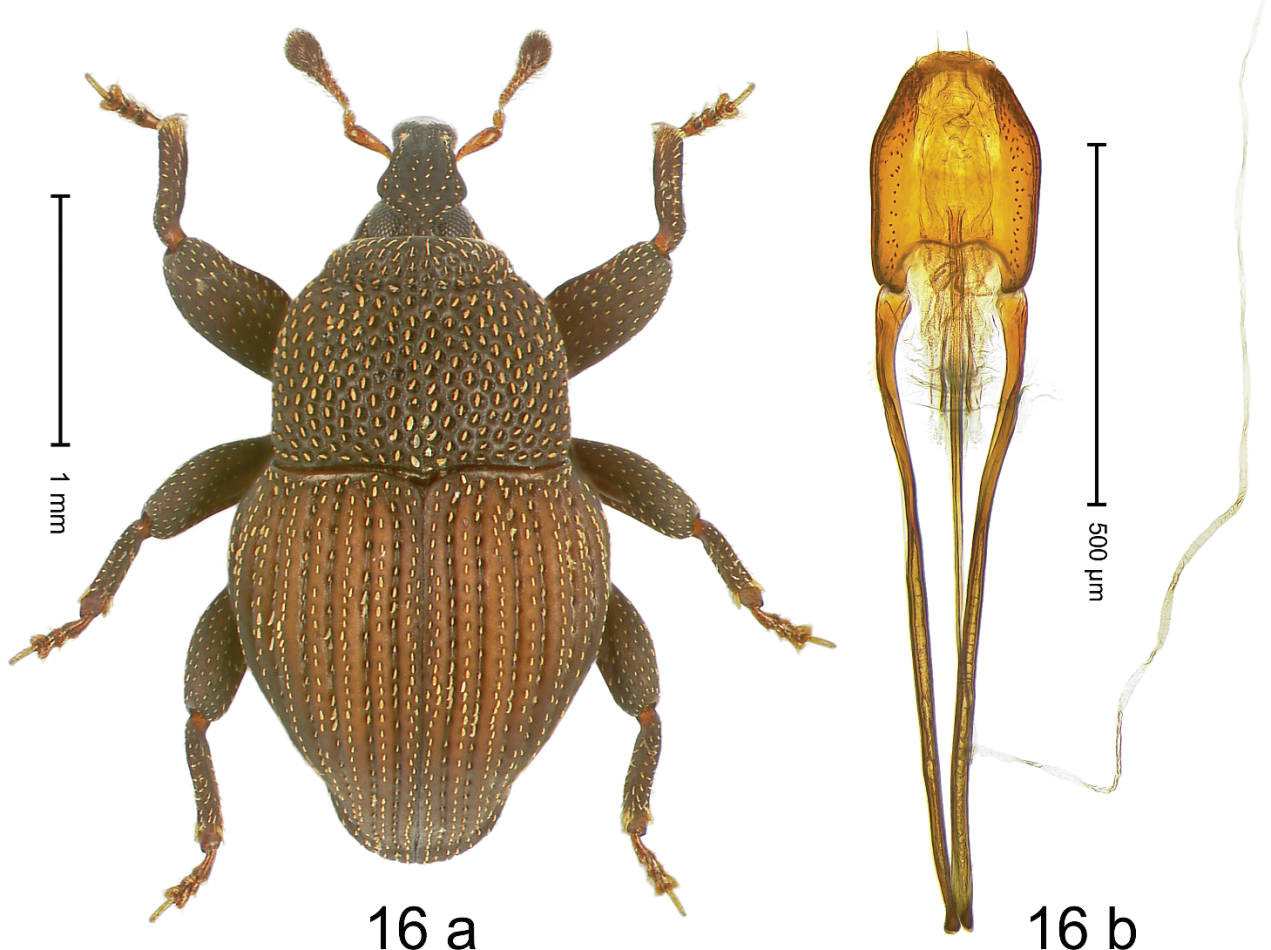
*Trigonopterus constrictus* Riedel, sp. n., holotype; (**a**) Habitus (**b**) Aedeagus.

Holotype, male (Fig. 16a). Length 2.55 mm. Color dark brown; antenna, tarsi, and elytra ferruginous. Body dull, microreticulate; subovate; in dorsal aspect with marked constriction between pronotum and elytron; in profile with shallow constriction. Rostrum sparsely punctate, with sublateral pair of furrows; epistome simple. Eyes large, divided into dorsal and ventral portions by marked incision of posterior margin. Pronotum with distinct subapical constriction; densely punctate with deep punctures; each puncture with one elongate-ovate, ochre scale. Elytra with striae deeply incised; intervals costate, each with one row of narrow scales. Metafemur subapically without stridulatory patch. Tibial apex with stout uncus and minute premucro. Onychium ca. 2.3 × longer than tarsomere 3. Aedeagus (Fig. 16b) with sides of apical half converging to apex, in profile markedly curved; transfer apparatus tubuliform; ductus ejaculatorius without bulbus. **Intraspecific variation**. Length 2.55–2.63 mm. Color with ferruginous elytra or entirely brown.

##### Material examined.

Holotype (MZB): ARC1731 (EMBL # HE616008), WEST NEW GUINEA, Jayawijaya Reg., Poga, S03°47.406', E138°35.507', 2410 m, 14-VII-2010, sifted. Paratypes (SMNK): WEST NEW GUINEA, Jayawijaya Reg., Poga: 1 ex, ARC1724 (EMBL # HE616004), S03°48.382', E138°34.780'; 2330 m, 13-VII-2010, sifted.

##### Distribution.

Jayawijaya Reg. (Poga). Elevation: 2330–2410 m.

##### Biology.

Sifted from leaf litter in montane forest.

##### Etymology.

This epithet is based on the Latin participle *constrictus* (constricted) and refers both to the constriction of the eye and the body between pronotum and elytron.

##### Notes.

*Trigonopterus constrictus* Riedel, sp. n. was coded as “*Trigonopterus* sp. 54” by Tänzler et al. (2012).

#### 17. Trigonopterus costatus

Riedel sp. n.

urn:lsid:zoobank.org:act:8640F855-706A-4462-9BB0-F50993518DCD

http://species-id.net/wiki/Trigonopterus_costatus

##### Diagnostic description.

**Figure 17.**
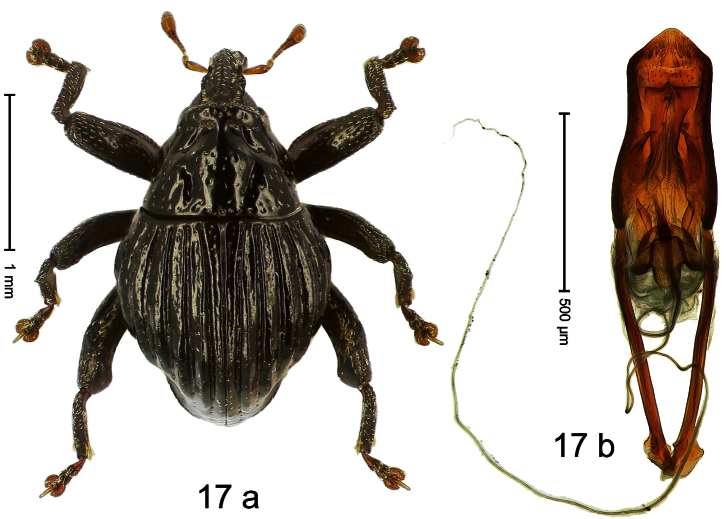
*Trigonopterus costatus* Riedel, sp. n., holotype; (**a**) Habitus (**b**) Aedeagus.

Holotype, male (Fig. 17a). Length 2.12 mm. Color black; antenna light ferruginous; legs deep ferruginous. Body subovate, with shallow constriction between pronotum and elytron; in profile evenly convex. Rostrum in basal half medially carinate, with pair of sublateral furrows posteriorly converging on forehead; weakly punctate, sparsely setose; epistome with transverse, angulate ridge. Pronotum with subapical constriction dorsally distinct, laterally indistinct; disk with longitudinal impressions, sparsely punctate, sparsely setose. Elytra with striae deeply incised, towards sides with deep interspersed punctures; intervals costate-carinate, subglabrous, with sparsely setose with minute recumbent setae; apex rounded. Femora edentate. Metafemur with simple dorsoposterior edge, in apical third with transverse row of small suberect setae, subapically with stridulatory patch. Abdominal ventrite 5 basally with pair of teeth. Aedeagus (Fig. 17b) apically subangulate, glabrous; with complex, symmetrical transfer apparatus; ductus ejaculatorius without bulbus. **Intraspecific variation**. Length 2.06–2.12 mm. Female rostrum in basal half with median carina less distinct.

##### Material examined.

Holotype (MZB): ARC0767 (EMBL # HE615450), WEST NEW GUINEA, Manokwari, Mt. Meja, S00°51.497', E134°04.949', 220 m, 05-XII-2007, sifted. Paratypes (SMNK, ZSM): WEST NEW GUINEA, Manokwari, Mt. Meja: 2 exx, ARC766 (EMBL # HE615449), ARC0768 (EMBL # HE615451), same data as holotype; 6 exx, S00°51.400', E134°04.918', 225 m, 06-XII-2007, sifted; 1 Ex, 200 m, 30-XII-2004.

##### Distribution.

Manokwari Reg. (Mt. Meja). Elevation: 220–225 m.

##### Biology.

Sifted from leaf litter in lowland forest.

##### Etymology.

This epithet is based on the Latin adjective *costatus* (ribbed) and refers to the elytral sculpture.

##### Notes.

*Trigonopterus costatus* Riedel, sp. n. was coded as “*Trigonopterus* sp. 202” by Tänzler et al. (2012).

#### 18. Trigonopterus costicollis

Riedel sp. n.

urn:lsid:zoobank.org:act:68D95F06-54A5-4B9E-90FD-9D0F12AE70D0

http://species-id.net/wiki/Trigonopterus_costicollis

##### Diagnostic description.

**Figure 18.**
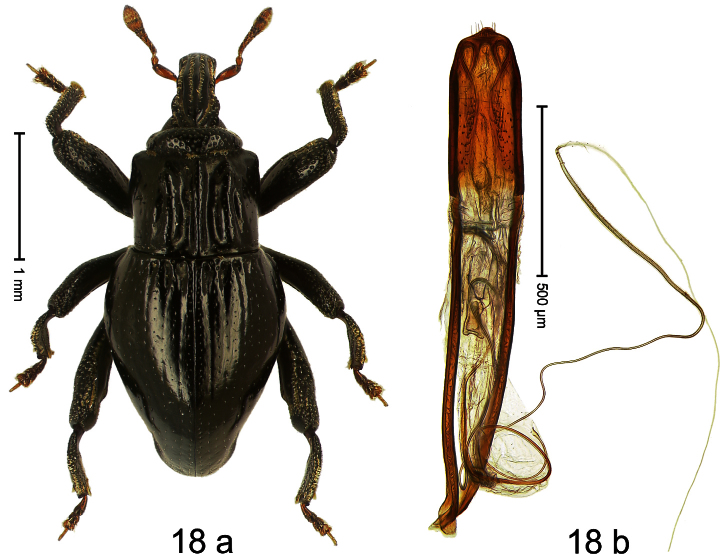
*Trigonopterus costicollis* Riedel, sp. n., holotype; (**a**) Habitus (**b**) Aedeagus.

Holotype, male (Fig. 18a). Length 2.80 mm. Color black; tarsi and antenna ferruginous. Body elongate; in dorsal aspect and in profile with distinct constriction between pronotum and elytron. Rostrum with median costa and pair of submedian costae, furrows between with sparse rows of suberect scales; epistome flat. Pronotum with marked subapical constriction, anteriorly densely punctate; disk sparsely punctate, deeply sculptured, with median ridge and pair of broad submedian ridges; behind constriction anteriorly with pair of lateral angular protrusions. Elytra subglabrous except basally striae 1–3 with each one fovea; remaining striae indistinct, marked by minute punctures; apex rounded. Femora edentate. Metafemur subapically with stridulatory patch. Tarsomere 3 small, hardly larger than preceding, onychium ca. 2.1 × longer than tarsomere 3. Aedeagus (Fig. 18b) apically angulate, sparsely setose; transfer-apparatus flagelliform; ductus ejaculatorius with indistinct bulbus.

##### Material examined.

Holotype (SMNK): ARC1094 (EMBL # HE615723), PAPUA NEW GUINEA, Simbu Prov., Karimui Dist., Haia, S06°43.948', E144°59.856', 915 m, 26-IX-2009, sifted.

##### Distribution.

Simbu Prov. (Haia). Elevation: 915 m.

##### Biology.

Sifted from leaf litter in primary forest.

##### Etymology.

This epithet is a combination of the Latin nouns *costa* (rib, ridge) and *collum* (neck; pronotum) refers to the sculpture of its pronotum.

##### Notes.

*Trigonopterus costicollis* Riedel, sp. n. was coded as “*Trigonopterus* sp. 166” by Tänzler et al. (2012).

#### 19. Trigonopterus crassicornis

Riedel sp. n.

urn:lsid:zoobank.org:act:C9B21DA1-F32C-4E44-8F9A-4632D30DEE1B

http://species-id.net/wiki/Trigonopterus_crassicornis

##### Diagnostic description.

**Figure 19.**
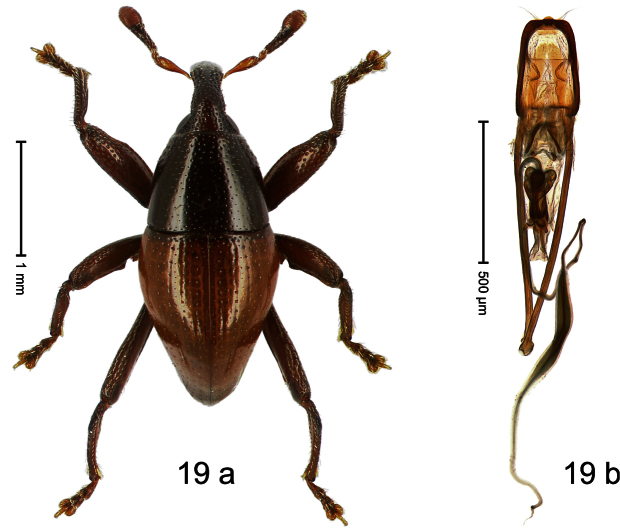
*Trigonopterus crassicornis* Riedel, sp. n., holotype; (**a**) Habitus (**b**) Aedeagus.

Holotype, male (Fig. 19a). Length 2.39 mm. Color orange-ferruginous; pronotum and parts of head black. Body subrhomboid, almost without constriction between pronotum and elytron; in profile dorsally flat, towards apex convex. Rostrum without median ridge, basally swollen and densely punctate, sparsely setose. Antenna with funicle swollen, continuous with club. Pronotum subglabrous, sparsely punctate with small to minute punctures. Elytra subglabrous, punctation confused with small to minute punctures. Profemur with anteroventral ridge terminating with tooth in apical third. Mesofemur with anteroventral ridge irregularly serrate; posteroventral ridge in apical half, terminating with rectangular protrusion. Metafemur laterally flattened; dorsally sparsely squamose with silvery scales; anteroventral ridge distinct; dorsoposterior edge simple; subapically with stridulatory patch. Uncus of metatibia small. Aedeagus (Fig. 19b). Body with sides converging; apex medially weakly extended, sublaterally with pair of sparse setose brushes; transfer-apparatus complex, symmetrical; ductus ejaculatorius with bulbus. **Intraspecific variation**. Length 2.22–2.85 mm. Female rostrum dorsally subglabrous with pair of submedian row of small punctures. Female antennal funicle less swollen. Female mesofemur along posteroventral edge simple, without rectangular protrusion.

##### Material examined.

Holotype (MZB): ARC0756 (EMBL # HE615439), WEST NEW GUINEA, Jayawijaya Reg., Jiwika, Kurulu, S03°56.146', E138°57.710', 2245–2290 m, “Mim 4”, 26-XI-2007. Paratypes (ARC, SMNK, ZSM): WEST NEW GUINEA, Jayawijaya Reg.: 3 exx, ARC0757 (EMBL # HE615440), ARC0758 (EMBL # HE615441), same data as holotype; 72 exx, ARC1785 (EMBL # HE616062), ARC1786 (EMBL # HE616063), Bokondini, S03°40.345', E138°42.386' to S03°40.255', E138°42.189', 1655–1700 m, 18-VII-2010; 22 exx, ARC1788 (EMBL # HE616065), ARC1789 (EMBL # HE616066), Bokondini, S03°41.787', E138°40.229' to S03°41.778', E138°40.129', 1705–1710 m, 17-VII-2010; 2 exx (1 marked ARC00611), Ilugwa, Melanggama, 1900–2200 m, 09-12-IX-1990; 2 exx, Ilugwa, Melanggama, trail to Pass Valley, 2100–2300 m, 09-10-IX-1990; 3 exx, Jiwika, Kurulu, ca. 1700–2300 m, 06-IX-1991; 1 ex, Jiwika, 1800–2300 m, 31-V-1998.

**Distribution**. Jayawijaya Reg. (Jiwika, Ilugwa, Bokondini). Elevation: 1700–2245 m.

**Biology**. Beaten from foliage of montane forests.

**Etymology**. This epithet is based on a combination of the Latin adjective *crassus* (thick) and *cornu* (horn, antenna) and refers to the thickened antennal funicle.

**Notes**. *Trigonopterus crassicornis* Riedel, sp. n. was coded as “*Trigonopterus* sp. 171” by Tänzler et al. (2012).

#### 20. Trigonopterus cuneipennis

Riedel sp. n.

urn:lsid:zoobank.org:act:422E2A48-0075-4C84-AC2B-080BD7EF0185

http://species-id.net/wiki/Trigonopterus_cuneipennis

##### Diagnostic description.

**Figure 20.**
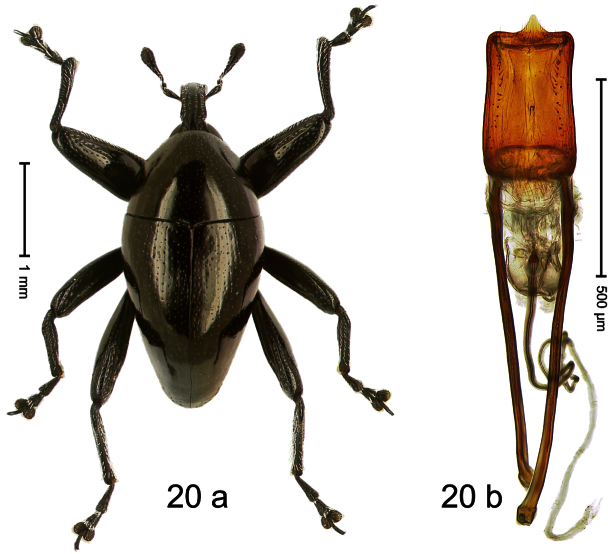
*Trigonopterus cuneipennis* Riedel, sp. n., holotype; (**a**) Habitus (**b**) Aedeagus.

Holotype, male (Fig. 20a). Length 3.00 mm. Color black. Body slender, subrhomboid; without constriction between pronotum and elytron; in profile evenly convex. Rostrum in apical third smooth; in basal 2/3 with broad median costa and pair of sublateral ridges; furrows containing sparse row of setae. Eyes with dorsal margin carinate. Head bordering eye with elongate impression. Pronotum subglabrous, sparsely punctate with minute punctures. Elytra subglabrous, punctation confused with minute punctures; striae hardly visible, impressed as very fine lines; lateral stria behind humeri with row of ca. 5 deep punctures. Femora subglabrous, including dorsum of metafemur without scales. Mesofemur on posterior surface with longitudinal ridge. Metafemur on posterior surface with two longitudinal furrows; dorsally with smooth ridge; subapically without stridulatory patch. Meso- and metatibia subapically simple, with uncus, without premucro. Aedeagus (Fig. 20b). Apex symmetrical, with median acute extension; body dorsally with two rows of sparse short setae; transfer apparatus small, dentiform; endophallus without distinct sclerites; ductus ejaculatorius without bulbus. **Intraspecific variation**. Length 1.95–3.00 mm.

##### Material examined.

Holotype (SMNK): ARC1839 (EMBL # HE616116), PAPUA NEW GUINEA, Eastern Highlands Prov., Aiyura, S06°21.033', E145°54.597', 2169 m, 06-II-2010. Paratypes (NAIC): PAPUA NEW GUINEA, Eastern Highlands Prov.: 1 ex, ARC1849 (EMBL # HE616126), Okapa, Kimiagomo village, Hamegoya, S06°25.727', E145°35.455', S06°25.117', E145°35.225', 1891–2131 m, 18-III-2010.

##### Distribution.

Eastern Highlands Prov. (Aiyura, Okapa). Elevation: 2131–2169 m.

##### Biology.

Beaten from foliage of montane forests.

##### Etymology.

This epithet is based on a combination of the Latin nouns *cuneus* (wedge) and *penna* (wing, elytron) and refers to the shape of elytra.

##### Notes. 

*Trigonopterus cuneipennis* Riedel, sp. n. was coded as “*Trigonopterus* sp. 96” by Tänzler et al. (2012).

#### 21. Trigonopterus cyclopensis

Riedel sp. n.

urn:lsid:zoobank.org:act:2801D93B-6053-415C-B6FB-726952E07B33

http://species-id.net/wiki/Trigonopterus_cyclopensis

##### Diagnostic description.

**Figure 21.**
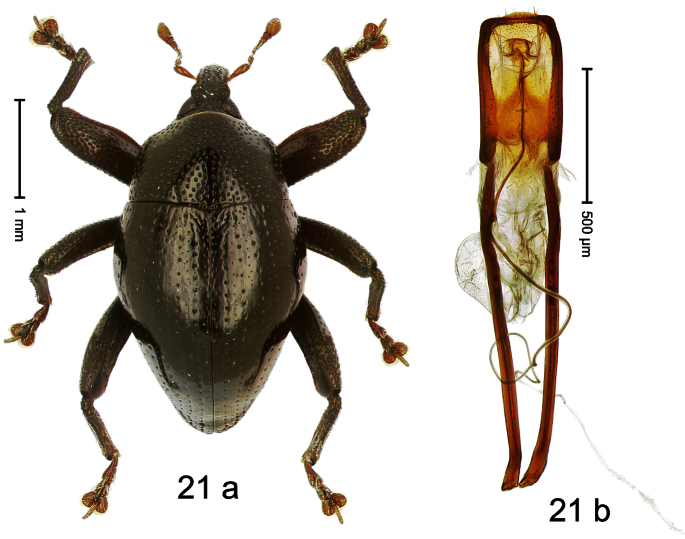
*Trigonopterus cyclopensis* Riedel, sp. n., holotype; (**a**) Habitus (**b**) Aedeagus.

Holotype, male (Fig. 21a). Length 3.27 mm. Color black; legs deep ferruginous, antenna light ferruginous. Body ovate; with weak constriction between pronotum and elytron; in profile evenly convex. Rostrum dorsally densely rugose-punctate, in basal half with brown erect scales, in apical half sparsely setose. Eyes large. Pronotum densely punctate with subtriangular, setiferous punctures. Elytral striae distinct, marked by regular rows of small punctures; intervals with row of minute punctures; laterally behind humeri with ridge bordered by row of deep punctures of stria 8. Legs squamose with inconspicuous brownish scales. Femora with anteroventral ridge terminating in apical third. Metafemur with weakly denticulate dorsoposterior edge; subapically with stridulatory patch. Abdominal ventrite 5 at middle with shallow, subquadrate impression. Aedeagus (Fig. 21b) with apex subangulate; transfer-apparatus flagelliform, longer than body of aedeagus; ductus ejaculatorius without bulbus. **Intraspecific variation**. Length 2.67–3.27 mm. Female rostrum in apical half with relatively small, sparse punctures. Abdominal ventrite 5 simple.

##### Material examined.

Holotype (MZB): ARC478 (EMBL # FN429185), WEST NEW GUINEA, Jayapura Reg., Cyclops Mts, Sentani, S02°31.2', E140°30.5', 1420–1520 m, 30.XI.2007, beaten. Paratypes (ARC, SMNK, ZSM): WEST NEW GUINEA, Jayapura Reg., Cyclops Mts, Sentani: 5 exx, same data as holotype; 4 exx, ARC0423 (EMBL # FN429134), ARC0483 (EMBL # FN429190), ARC0674 (EMBL # FN429319), ARC0675 (EMBL # FN429320), S02°31.3', E140°30.5', 1200–1420 m, 30.XI.2007, beaten; 2 exx (1 marked as “ARC0409”), 1100–1600 m, 05-X-1991.

##### Distribution.

Jayapura Reg. (Cyclops Mts). Elevation: ca. 1420 m.

##### Biology.

Collected by beating foliage in montane forests.

##### Etymology.

This epithet is based on the type locality, the Cyclops Mountains.

##### Notes.

*Trigonopterus cyclopensis* Riedel, sp. n. was coded as “*Trigonopterus* sp. 5” by Riedel et al. (2010) and Tänzler et al. (2012), respectively “*Trigonopterus* spe” in the EMBL/GenBank/DDBJ databases.

#### 22. Trigonopterus dentirostris

Riedel sp. n.

urn:lsid:zoobank.org:act:0026240A-0B72-4B1C-8DB9-7757D2C10530

http://species-id.net/wiki/Trigonopterus_dentirostris

##### Diagnostic description.

**Figure 22.**
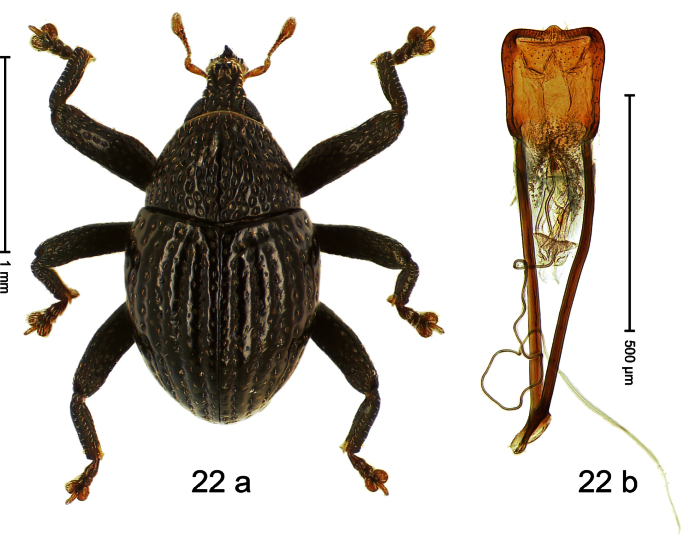
*Trigonopterus dentirostris* Riedel, sp. n., holotype; (**a**) Habitus (**b**) Aedeagus.

Holotype, male (Fig. 22a). Length 1.65 mm. Color black; antenna and tarsi ferruginous. Body subovate; in dorsal aspect without constriction between pronotum and elytron; in profile with distinct constriction. Rostrum at base with median ridge and pair of submedian ridges; at middle with anteriorly hollowed protuberance dorsally bearing pair of denticles; between protuberance and epistome relatively flat, with sparse erect scales; epistome at middle with dorsoposteriad directed horn. Pronotum punctate-rugose, each puncture containing one narrow transparent scale; medially with indistinct ridge. Elytra with striae deeply impressed; punctures large, each containing one downcurved seta; intervals weakly costate, subglabrous. Metafemur dorsoposteriorly simple, subapically without stridulatory patch. Aedeagus (Fig. 22b). Body widening to shortly before apex; medially weakly extended; endophallus denticulate; transfer apparatus spiniform, curved; ductus ejaculatorius with weak bulbus. **Intraspecific variation**. Length 1.65–1.92 mm. Female rostrum dorsally even, without teeth or cavities, medially subglabrous, sublaterally punctate.

##### Material examined.

Holotype (MZB): ARC0538 (EMBL # FN429244), WEST NEW GUINEA, Jayapura Reg., Cyclops Mts, S02°31.594', E140°30.407', 1065 m, 21-XI-2007, sifted. Paratypes (ARC, SMNK, ZSM): WEST NEW GUINEA, Jayapura Reg., Cyclops Mts, Sentani: 2 exx, ARC0544 (EMBL # FN429250), S02°31.683', E140°30.281', 960 m, 21-XI-2007, sifted; 6 exx, ARC0545 (EMBL # FN429251), Sentani, S02°31.776', E140°30.215', 945 m, 21-XI-2007, sifted; 1 ex, Sentani, S02°31.603', E140°30.434', 1095 m, 28-XI-2007, sifted; 2 exx, Sentani, 950–1450 m, 03-X-1992; 6 exx (1 marked ARC0026), Sentani, 1000 m, 23-XII-2004, sifted; 1 ex, Sentani, 1100 m, 23-XII-2004, sifted; 3 exx, ARC1690 (EMBL # HE615977), ARC1691 (EMBL # HE615978), Angkasa indah, S02°30.346', E140°42.087', 490 m, 28-VI-2010, sifted.

##### Distribution.

Jayapura Reg. (Cyclops Mts). Elevation: 490–1095 m.

##### Biology.

Sifted from leaf litter in montane forest.

##### Etymology.

This epithet is based on a combination of the Latin nouns *dens* (tooth) and *rostrum* (snout) and refers to the dorsal protrusions of the rostrum.

##### Notes.

*Trigonopterus dentirostris* Riedel, sp. n. was coded as “*Trigonopterus* sp. 49” by Riedel et al. (2010) and Tänzler et al. (2012), respectively “*Trigonopterus* spaw” in the EMBL/GenBank/DDBJ databases.

#### 23. Trigonopterus discoidalis

Riedel sp. n.

urn:lsid:zoobank.org:act:17DF68F9-C2DC-43E8-A0EC-03FB104DFD37

http://species-id.net/wiki/Trigonopterus_discoidalis

##### Diagnostic description.

**Figure 23.**
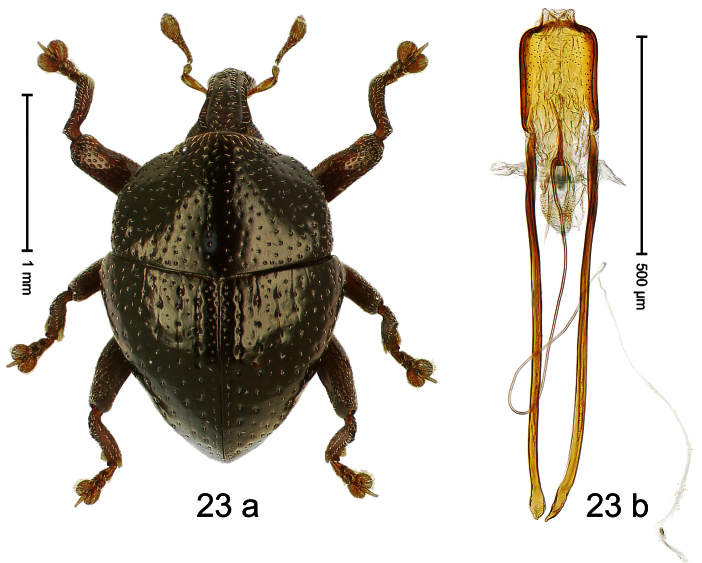
*Trigonopterus discoidalis* Riedel, sp. n., holotype; (**a**) Habitus (**b**) Aedeagus.

Holotype, male (Fig. 23a). Length 2.20 mm. Color black; legs ferruginous, antenna light ferruginous. Body subglobose; in dorsal aspect with weak constriction between pronotum and elytron; in profile almost evenly convex. Rostrum with pair of sublateral furrows and pair of submedian row of punctures, each containing row of mesally directed setae; epistome simple. Pronotum with large disk and distinct lateral edges; moderately densely punctate with small setiferous punctures. Elytra converging to subangulate apex; striae distinct, marked by small punctures; intervals subglabrous, with row of minute punctures; some punctures with one minute recumbent seta. Metafemur with weakly denticulate dorsoposterior edge; subapically with stridulatory patch. Abdominal ventrites 1–2 deeply excavated. Aedeagus (Fig. 23b) with body flattened, sides subparallel, apex subangulate; apodemes ca. 3 X as long as body; transfer-apparatus spiniform; ductus ejaculatorius, without bulbus. **Intraspecific variation**. Length 1.68–2.43 mm. Female rostrum more slender than in males, dorsally punctures smaller.

##### Material examined.

Holotype (SMNK): ARC1175 (EMBL # HE615803), PAPUA NEW GUINEA, Morobe Prov., Huon peninsula, Mindik, S06°27.311', E147°24.073', 1570 m, 10-X-2009. Paratypes (ARC, NAIC, SMNK, ZSM): Morobe Prov., Huon peninsula, Mindik: 5 exx, same data as holotype; 12 exx, ARC1179 (EMBL # HE615807), ARC1180 (EMBL # HE615808), S06°27.221', E147°24.185', 1670 m, 10-X-2009; 5 exx, 1450 m, 26-IV-1998; 1 ex, 1400–1550 m, 27-IV-1998.

##### Distribution.

Morobe Prov. (Mindik). Elevation: 1450–1670 m.

##### Biology.

Sifted from leaf litter in montane forest.

##### Etymology.

This epithet is based on the Latin adjective *discoidalis* (shaped like a disk) and refers to the species´ outline when viewed from above.

##### Notes.

*Trigonopterus discoidalis* Riedel, sp. n. was coded as “*Trigonopterus* sp. 88” by Tänzler et al. (2012).

#### 24. Trigonopterus dromedarius

Riedel sp. n.

urn:lsid:zoobank.org:act:1B816F75-55CA-4F53-87F4-DA832747626D

http://species-id.net/wiki/Trigonopterus_dromedarius

##### Diagnostic description.

**Figure 24.**
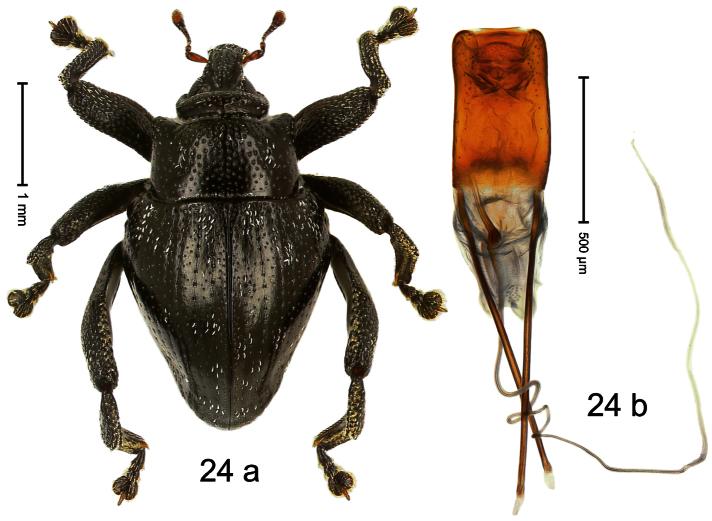
*Trigonopterus dromedarius* Riedel, sp. n., holotype; (**a**) Habitus (**b**) Aedeagus.

Holotype, male (Fig. 24a). Length 3.31 mm. Color black; tarsi and tibiae dark ferruginous; antenna light ferruginous. Body subrhomboid; in dorsal aspect with marked constriction between pronotum and elytron; in profile with moderate constriction. Rostrum medially punctate, with pair of sublateral furrows converging posteriorly on forehead; epistome forming indistinct, angulate ridge. Pronotum with marked subapical constriction, sides subparallel, behind subapical constriction projecting with marked angular protrusions; center of disk densely punctate, laterally with sparse punctures. Elytra of subtriangular shape; median suture carinate; striae moderately impressed; intervals weakly punctate; surface weakly microreticulate, with sparse clusters of white recumbent scales; interval 7 subapically forming indistinct ridge. Femora edentate. Meso- and metatibia in basal half markedly widened, dorsal edge curved, denticulate. Metafemur with denticulate dorsoposterior edge, subapically without stridulatory patch. Metathoracic and abdominal venter forming common concavity, subglabrous. Aedeagus (Fig. 24b) with body parallel-sided, markedly curved ventrad; apex medially extended; transfer apparatus spiniform; ductus ejaculatorius without bulbus. **Intraspecific variation**. Length 3.22–3.69 mm. Integument relatively smooth and with scattered white scales in specimens from Sentani, dull-coriaceous and almost nude in specimens from Angkasa indah. Aedeagus with median tip acute in specimens from Sentani, median tip more rounded in specimens from Angkasa indah.

##### Material examined.

Holotype (MZB): ARC0512 (EMBL # FN429218), WEST NEW GUINEA, Jayapura Reg., Cyclops Mts, Sentani, S02°32.031', E140°30.412', 710 m, 02-XII-2007, sifted. Paratypes (SMNK, ZSM): WEST NEW GUINEA, Jayapura Reg., Cyclops Mts: 1 ex, ARC0534 (EMBL # FN429240), Sentani, S02°31.516', E140°30.436', 1150 m, 21-XI-2007, sifted; 2 exx, ARC0549 (EMBL # FN429255), Sentani, S02°31.776', E140°30.215', 945 m, 21-XI-2007, sifted; 2 exx, Sentani, S02°31.603', E140°30.434', 1095 m, 28-XI-07, sifted; 1 ex, Sentani, S02°31.912', E140°30.416', 785 m, 02-XII-2007, sifted; 1 ex, ARC0092, Sentani,1000 m, 23-XII-2004, sifted; 1 ex, S02°31.425', E140°30.474', 1265 m, 30-XI-2007, sifted; 2 exx, ARC1684 (EMBL # HE615971), ARC1685 (EMBL # HE615972), Angkasa indah, S02°30.346', E140°42.087', 490 m, 28-VI-2010, sifted.

##### Distribution.

Jayapura Reg. (Cyclops Mts). Elevation: 490–1265 m.

##### Biology.

Sifted from leaf litter in primary forest.

##### Etymology.

This epithet is based on the name of the dromedary camel (*Camelus dromedaries* L.) and refers to the body shape.

##### Notes.

*Trigonopterus dromedarius* Riedel, sp. n. was coded as “*Trigonopterus* sp. 41” by Riedel et al. (2010) and Tänzler et al. (2012), respectively “*Trigonopterus* spao” in the EMBL/GenBank/DDBJ databases. Specimens from Angkasa indah and Sentani exhibit minor morphological differences (e.g. tip of aedeagus; surface of integument) and a high *cox1* p-distance of 7.79%. These allopatric populations may be regarded “subspecies”.

#### 25. Trigonopterus durus

Riedel sp. n.

urn:lsid:zoobank.org:act:4D45A783-70C2-4DE5-B061-6B227AF90B5C

http://species-id.net/wiki/Trigonopterus_durus

##### Diagnostic description.

**Figure 25.**
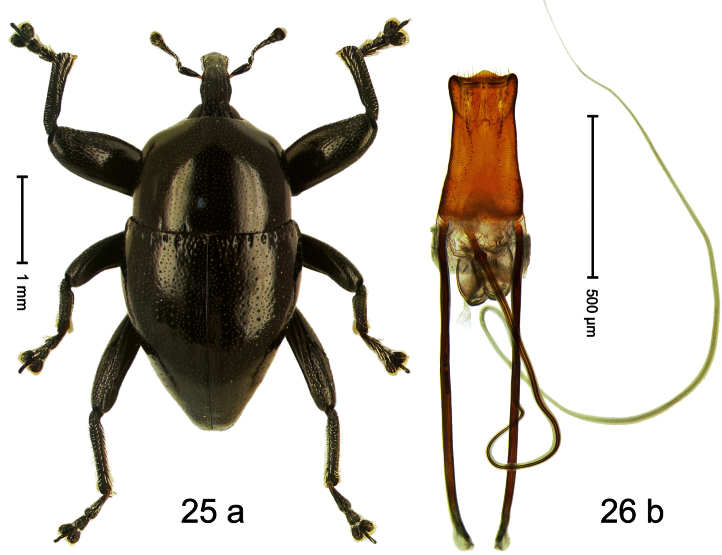
*Trigonopterus durus* Riedel, sp. n., holotype; (**a**) Habitus (**b**) Aedeagus.

Holotype, male (Fig. 25a). Length 3.69 mm. Color black; base of antennal scape ferruginous. Body subovate; with weak constriction between pronotum and elytron; in profile evenly convex. Rostrum punctate-rugose, with pair of sublateral furrows. Eyes dorsally bordered by furrow. Pronotum subglabrous, densely punctate with minute punctures; disk subquadrate, rounded towards sides. Elytra tapering apicad; striae indistinct, marked by rows of small punctures; intervals flat, with confused minute punctures; elytral base with row of foveae continued laterally behind humeri bordering ridge. Femora subglabrous, edentate. Metafemur dorsally partly covered with silvery scales; with weakly denticulate dorsoposterior edge; subapically with stridulatory patch. Mesotibia and metatibia with inconspicuous straight premucro below tarsal insertion. Aedeagus (Fig. 25b) with sides of body basally converging, in apical 1/3 with weak constriction; apex with median, broad angular extension, sparsely setose; transfer apparatus spiniform; ductus ejaculatorius without bulbus. **Intraspecific variation**. Length 3.38–4.64 mm. Female rostrum slender, dorsally subglabrous, sparsely punctate with small punctures. Pronotum of males anteriorly subangulate, in females sides curving more evenly towards apex. Elytra of females shorter and dorsally rather convex; elytra of males apically slightly extended and dorsally slightly flattened.

##### Material examined.

Holotype (MZB): ARC1790 (EMBL # HE616067), WEST NEW GUINEA, Jayawijaya Reg., Bokondini, S03°41.787', E138°40.229' to S03°41.778', E138°40.129', 1705–1710 m, 17-VII-2010. Paratypes (ARC, SMNK, ZSM): 91 exx, ARC1791 (EMBL # HE616068), ARC1792 (EMBL # HE616069), same data as holotype; 6 exx, ARC1793 (EMBL # HE616070), Bokondini, S03°40.345', E138°42.386' to S03°40.255', E138°42.189', 1655–1700 m, 18-VII-2010; 85 exx, Angguruk, 1600–1700 m, 21-IX-1991; 1 ex, Angguruk, 1200–1500 m, 23-IX-1992; 2 exx, Angguruk – Tanggeam, 1500–1800 m, 28-29-IX-1991.

##### Distribution.

Jayawijaya Reg. (Bokondini, Angguruk). Elevation: 1700–1710 m.

##### Biology.

Beaten from foliage of montane forests.

##### Etymology.

This epithet is based on the Latin adjective *durus* (hard, tough) and refers to the physical properties of the species. The name would be equally fitting many other species of this genus and should not be seen as diagnostic.

##### Notes.

*Trigonopterus durus* Riedel, sp. n. was coded as “*Trigonopterus* sp. 102” by Tänzler et al. (2012).

#### 26. Trigonopterus echinus

Riedel sp. n.

urn:lsid:zoobank.org:act:A54F0EBB-1A80-4AFC-9550-3844181CD2F7

http://species-id.net/wiki/Trigonopterus_echinus

##### Diagnostic description.

**Figure 26.**
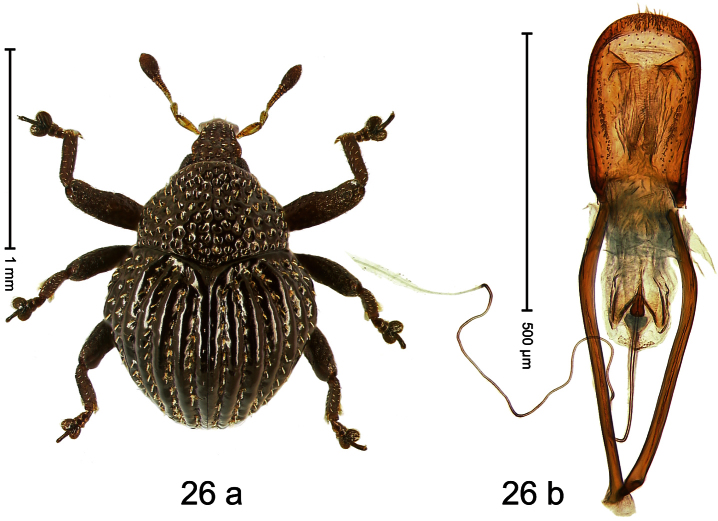
*Trigonopterus echinus* Riedel, sp. n., holotype; (**a**) Habitus (**b**) Aedeagus.

Holotype, male (Fig. 26a). Length 1.41 mm. Color black; antenna light ferruginous, club dark; legs and head dark ferruginous. Body subglobose; with distinct constriction between pronotum and elytron; in profile almost evenly rounded, with weak constriction between pronotum and elytron. Rostrum basally with indistinct, median ridge and pair of submedian ridges, sparsely setose; apical 1/3 subglabrous; epistome simple. Eyes small. Pronotum with weak subapical constriction; disk densely, coarsely punctate; each puncture with short, yellowish scale. Elytra with striae deeply incised; with suberect, yellowish scales; intervals subglabrous, markedly costate. Femora subapically ventrally constricted. Metafemur subapically with stridulatory patch. Protibia with long, hook-shaped uncus. Aedeagus (Fig. 26b) with apex rounded; transfer apparatus symmetrical, spiniform; ductus ejaculatorius with indistinct bulbus. **Intraspecific variation**. Length 1.28–1.54 mm. Female rostrum basally without ridges; apical half subglabrous. Suberect scales may be partly abraded.

##### Material examined.

Holotype (MZB): ARC0781 (EMBL # HE615464), WEST NEW GUINEA, Manokwari, Arfak Mts, S01°04.087', E133°54.268', 1520 m, 08-XII-2007, sifted. Paratypes (SMNK, ZSM): 21 exx, ARC0782 (EMBL # HE615465), ARC0783 (EMBL # HE615466), same data as holotype; Mokwam, Siyoubrig, S01°06.107', E133°54.888', 1530 m, 10–XII-2007.

##### Distribution.

Manokwari Reg. (Arfak Mts). Elevation: 1520 m.

##### Biology.

Sifted from leaf litter in montane forest dominated by *Nothofagus*.

##### Etymology.

This epithet is based on the Latin noun *echinus* (hedgehog) in apposition and refers to the species´ general habitus and its suberect scales resembling spines.

##### Notes.

*Trigonopterus echinus* Riedel, sp. n. was coded as “*Trigonopterus* sp. 230” by Tänzler et al. (2012).

#### 27. Trigonopterus edaphus

Riedel sp. n.

urn:lsid:zoobank.org:act:ACC1DBCF-2E5E-486D-A61F-0F6F6A6301FE

http://species-id.net/wiki/Trigonopterus_edaphus

##### Diagnostic description.

**Figure 27.**
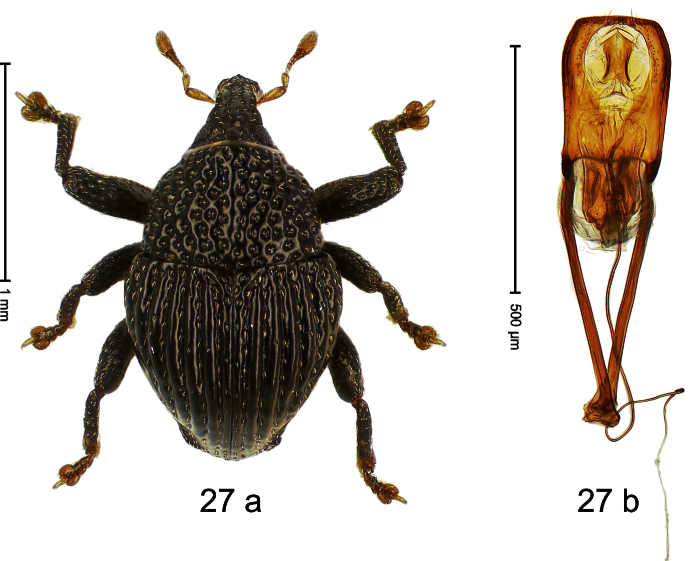
*Trigonopterus edaphus* Riedel, sp. n., holotype; (**a**) Habitus (**b**) Aedeagus.

Holotype, male (Fig. 27a). Length 1.48 mm. Color black; antenna light ferruginous; legs deep ferruginous. Body subglobose, in dorsal aspect with marked constriction between pronotum and elytron; dorsally flattened; anteriorly profile almost straight, convex at apex. Rostrum coarsely rugose-punctate; epistome forming distinct, transverse ridge. Pronotum coarsely punctate-reticulate. Elytra with striae deeply incised, with sparse rows of setae; intervals costate, subglabrous, with few minute punctures; interval 7 subapically forming ridge, projecting dentiform; apex subangulate. Legs. Anteroventral ridge of meso- and metafemur ending in apical third, forming indistinct tooth. Metafemur dorsoposteriorly simple, subapically with stridulatory patch. Aedeagus (Fig. 27b) with apex subtruncate, slightly angulate; transfer apparatus spiniform, shorter than body of aedeagus; ductus ejaculatorius without bulbus. **Intraspecific variation**. Length 1.32–2.02 mm. Body of females subovate. Female rostrum dorsally subglabrous, coarsely punctate; epistome simple. Female elytral apex laterally not dentiform.

##### Material examined.

Holotype (MZB): ARC0737 (EMBL # HE615420), WEST NEW GUINEA, Jayawijaya Reg., Jiwika, Kurulu, S03°57.161', E138°57.357', 1875 m, 24-XI-2007, sifted. Paratypes (ARC, SMNK, ZSM): WEST NEW GUINEA, Jayawijaya Reg., Jiwika, Kurulu: 27 exx, ARC0738 (EMBL # HE615421), ARC0739 (EMBL # HE615422), ARC0740 (EMBL # HE615423), ARC0741 (EMBL # HE615424), ARC0742 (EMBL # HE615425), same data as holotype; 64 exx, S03°57.161', E138°57.357', 1875 m, 11-VII-2010, sifted; 8 exx (1 marked ARC0074), 1900–2050 m, 24-X.1993, sifted;12 exx, 1900–2000 m, 23-IX-1992, sifted; 3 exx, 1900–2050 m, 24-IX-1992, sifted; 2 exx, ca. 1700–2300 m, 02-IX-1991, sifted; 7 exx, 1700–2000 m, 11-IX-1991, sifted.

##### Distribution.

Jayawijaya Reg. (Jiwika). Elevation: 1875–1900 m.

##### Biology.

Sifted from leaf litter in montane forest.

##### Etymology.

This epithet is the latinized form of the Greek word *edaphos* (soil, ground) and treated as an adjective. It refers to the habit of this litter-dwelling species.

##### Notes.

*Trigonopterus edaphus* Riedel, sp. n. was coded as “*Trigonopterus* sp. 224” by Tänzler et al. (2012).

#### 28. Trigonopterus eremitus

Riedel sp. n.

urn:lsid:zoobank.org:act:A26056D8-6DDE-462D-973C-CB428A9B389F

http://species-id.net/wiki/Trigonopterus_eremitus

##### Diagnostic description.

**Figure 28.**
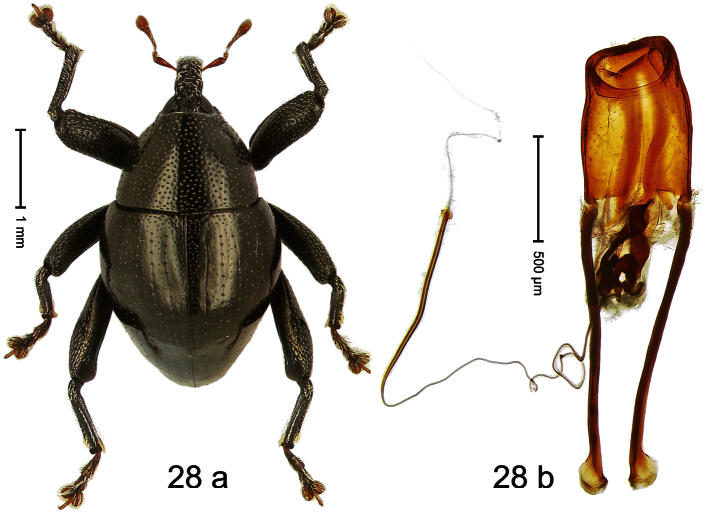
*Trigonopterus eremitus* Riedel, sp. n., holotype; (**a**) Habitus (**b**) Aedeagus.

Holotype, male (Fig. 28a). Length 3.66 mm. Color black; tarsi and antenna ferruginous. Body ovate; with weak constriction between pronotum and elytron; in profile evenly convex. Rostrum dorsally with distinct median carina and sparse rows of upcurved scales; submedian ridges indistinct, irregular. Pronotum densely punctate except along impunctate midline. Elytra punctate with minute punctures, especially on intervals; strial punctures slightly larger; striae impressed as fine lines; lateral stria behind humeri simple, not deepened. Femora edentate. Metafemur with denticulate dorsoposterior edge; subapically with fine stridulatory patch. Metatibia apically with uncus, without premucro. Abdominal ventrite 5 flat, densely setose with suberect setae. Aedeagus (Fig. 28b) slightly asymmetrical; with distinct, asymmetrical transfer-apparatus; ductus ejaculatorius with bulbus. **Intraspecific variation**. Length 3.38–3.72 mm. Female rostrum dorsally in basal half with median and pair of submedian ridges, apical half punctate. Female abdominal ventrite 5 flat, sparsely setose with recumbent setae.

##### Material examined.

Holotype (MZB): ARC426 (EMBL # FN429137), WEST NEW GUINEA, Jayapura Reg., Cyclops Mts, Sentani, S02°31.2', E140°30.5', 1420–1520 m, 30-XI-2007, beaten. Paratypes (ARC, SMNK, ZSM): WEST NEW GUI-NEA, Jayapura Reg., Cyclops Mts, Sentani: 9 exx, ARC0425 (EMBL # FN429136), ARC0427 (EMBL # FN429138), same data as holotype; 1 ex (marked as “ARC0411”), 1200–1400 m, 9-VIII-1992; 1 ex, 1100–1600 m, 5.X.1991.

##### Distribution.

Jayapura Reg. (Cyclops Mts). Elevation: 1400–1420 m.

##### Biology.

Collected by beating foliage in montane forests.

##### Etymology.

This epithet is the latinized form of the Greek noun *eremites* (hermit) and refers to the species´ restricted occurrence in the montane forests of the Cyclops Mountains.

##### Notes.

*Trigonopterus eremitus* Riedel, sp. n. was coded as “*Trigonopterus* sp. 6” by Riedel et al. (2010) and Tänzler et al. (2012), respectively “*Trigonopterus* spf” in the EMBL/GenBank/DDBJ databases.

#### 29. Trigonopterus euops

Riedel sp. n.

urn:lsid:zoobank.org:act:1F045C52-B021-418F-9CB5-6C2EBA36352F

http://species-id.net/wiki/Trigonopterus_euops

##### Diagnostic description.

**Figure 29.**
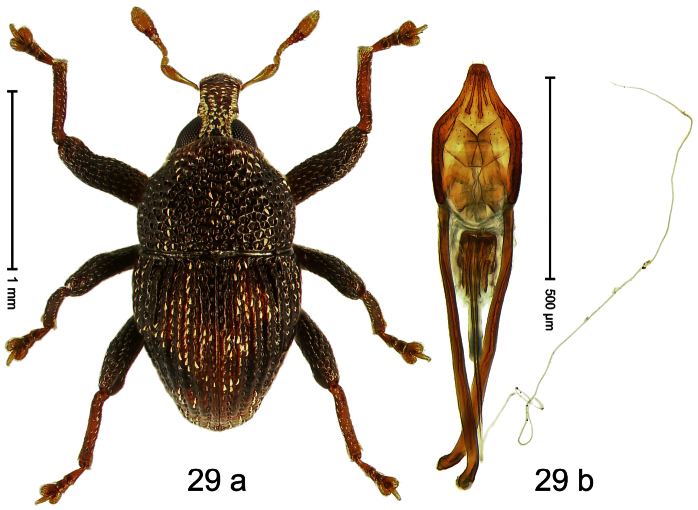
*Trigonopterus euops* Riedel, sp. n., holotype; (**a**) Habitus (**b**) Aedeagus.

Holotype, male (Fig. 29a). Length 1.70 mm. Color of antenna, tarsi, tibiae, and rostrum ferruginous; head and pronotum black; elytra black, with ferruginous patches near base of intervals 2–4, continued to apex with irregular patches on intervals 1–4 to apex. Body subovate; in dorsal aspect and in profile with distinct constriction between pronotum and elytron. Eyes large. Rostrum punctate, without distinct longitudinal ridges; laterally with rows of cream-colored scales. Pronotum densely punctate-reticulate; with few scattered cream-colored scales; each puncture containing one brownish seta. Elytra with striae deeply impressed, intervals costate, with scattered white scales. Femora ventrally edentate, subapically constricted. Tibial uncus simple, curved. Metafemur subapi- cally with stridulatory patch. Aedeagus (Fig. 29b) with sides of body sinuate, converging; apex extended, pointed, curved ventrad; transfer apparatus compact, symmetrical; ductus ejaculatorius basally swollen, without bulbus. **Intraspecific variation**. Length 1.63–1.86 mm. Ferruginous color of elytra more or less extensive. Female rostrum dorsally subglabrous, laterally punctate. Body of males rather slender, females slightly stouter.

**Material examined**. Holotype (MZB): ARC0784 (EMBL # HE615467), WEST NEW GUINEA, Manokwari Reg., Manokwari, Arfak Mts, S01°04.087', E133°54.268', 1520 m, 08-XII-2007. Paratypes (SMNK, ZSM): WEST NEW GUINEA, Manokwari Reg., Manokwari, Arfak Mts: 6 exx, same data as holotype; 4 exx, ARC0775 (EMBL # HE615458), ARC0776 (EMBL # HE615459), S01°03.723', E133°54.145', 1385 m, 08-XII-2007; 2 exx, Mokwam, Siyoubrig, S01°06.107', E133°54.888', 1530 m, 10-XII-2007.

##### Distribution.

Manokwari Reg. (Arfak Mts). Elevation: 1385–1530 m.

##### Biology.

Sifted from leaf litter in montane forest.

##### Etymology.

This epithet is based on the Greek *euops* (well-sighted) and refers to the species´ relatively large eyes, at least by comparison with other edaphic species.

##### Notes.

*Trigonopterus euops* Riedel, sp. n. was coded as “*Trigonopterus* sp. 53” by Tänzler et al. (2012).

#### 30. Trigonopterus ferrugineus

Riedel sp. n.

urn:lsid:zoobank.org:act:64E0AF8A-8988-484C-AA0D-1B66A9C0CD10

http://species-id.net/wiki/Trigonopterus_ferrugineus

##### Diagnostic description.

**Figure 30.**
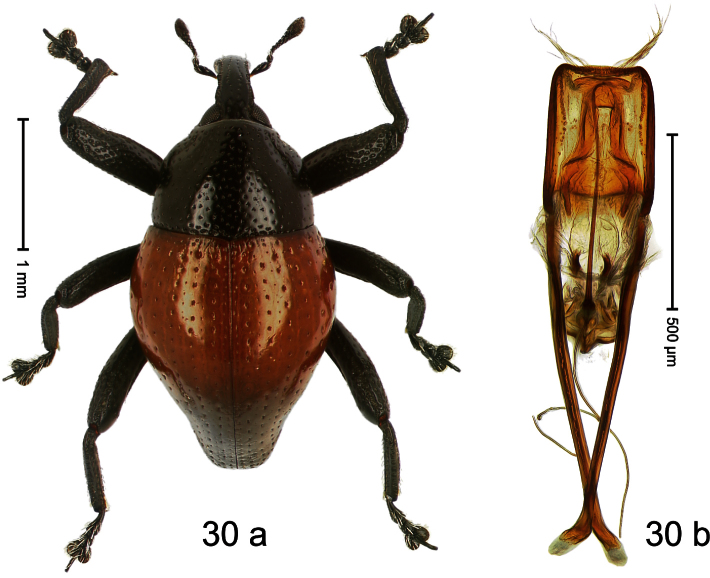
*Trigonopterus ferrugineus* Riedel, sp. n., holotype; (**a**) Habitus (**b**) Aedeagus.

Holotype, male (Fig. 30a). Length 2.73 mm. Color black; elytra orange-red, apically changing to black. Body with distinct constriction between pronotum and elytron; in profile almost evenly convex. Rostrum dorsally relatively flat, sparsely punctate, dorsolaterally with pair of furrows continuing along eye, furrows containing row of mesad directed setae. Pronotum densely punctate with punctures of subtriangular shape, interspaces larger than puncture´s width. Elytra with striae distinct, dorsally punctures small, along base and laterally punctures large; interspaces subglabrous. Femora edentate. Metafemur with indistinct, simple dorsoposterior edge; subapically with stridulatory patch. Thoracic and abdominal venter partly with dense erect setae. Aedeagus (Fig. 30b) apically subangulate, with pair of sublateral setose brushes; transfer apparatus flagelliform, longer than body; ductus ejaculatorius without bulbus. **Intraspecific variation**. Length 2.73–2.85 mm. Female venter subglabrous, sparsely setose with short recumbent setae.

##### Material examined.

Holotype (MZB): ARC0477 (EMBL # FN429184), WEST NEW GUINEA, Jayapura Reg., Cyclops Mts, Sentani, S02°31.2', E140°30.5', 1420–1520 m, 30-XI-2007, beaten. Paratypes (SMNK, ZSM): WEST NEW GUINEA, Jayapura Reg., Cyclops Mts, Sentani: 9 exx, ARC0475 (EMBL # FN429182), ARC0476 (EMBL # FN429183), same data as holotype; 1 ex, S02°31.3', E140°30.5', 1200–1420 m, 30-XI-2007; 1 ex, S02°31.182', E140°30.542', 1510 m, 30-XI-2007, sifted.

##### Distribution.

Jayapura Reg. (Cyclops Mts). Elevation: 1420–1510 m.

##### Biology.

Collected by beating foliage in montane crippled forests.

##### Etymology.

This epithet is based on the Latin adjective *ferrugineus* (rusty, red-brown) and refers to the species´ elytra.

##### Notes.

*Trigonopterus ferrugineus* Riedel, sp. n. was coded as “*Trigonopterus* sp. 29” by Riedel et al. (2010) and Tänzler et al. (2012), respectively “*Trigonopterus* spac” in the EMBL/GenBank/DDBJ databases.

#### 31. Trigonopterus fusiformis

Riedel sp. n.

urn:lsid:zoobank.org:act:863A0812-1FB3-4C7D-906B-B701FEB19B4A

http://species-id.net/wiki/Trigonopterus_fusiformis

##### Diagnostic description.

**Figure 31.**
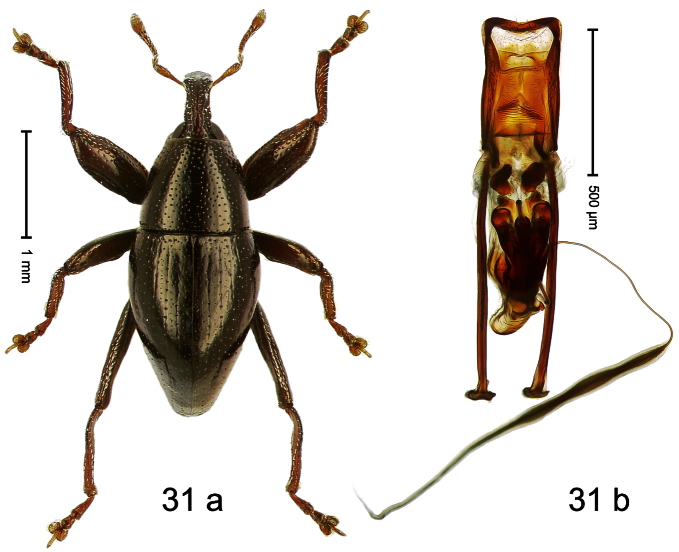
*Trigonopterus fusiformis* Riedel, sp. n., holotype; (**a**) Habitus (**b**) Aedeagus.

Holotype, male (Fig. 31a). Length 2.63 mm. Color black, legs and antenna ferruginous. Body subrhomboid; almost without constriction between pronotum and elytron; in profile dorsally flat, towards apex convex. Rostrum in apical half dorsally flattened, slightly widened, rugose-punctate; basal half markedly swollen in profile, narrow in dorsal aspect; with indistinct median ridge, densely punctate; dorsolaterally with pair of wide furrows containing erect narrow scales. Pronotum subglabrous, punctate, punctures becoming larger and denser towards apex. Elytra densely punctate; strial punctures slightly larger than minute punctures on intervals; striae impressed as fine lines. Anteroventral ridge of femora terminating with tooth in apical third; tooth largest in metafemur. Metafemur laterally markedly flattened; dorsally with row of silvery scales; posteroventral ridge indistinct, at middle with denticle; dorsoposterior edge weakly denticulate; subapically with stridulatory patch. Aedeagus (Fig. 31b). Body with sides subparallel, extended dorsad; profile of body subtriangular; apex subtruncate, subglabrous; transfer-apparatus complex, symmetrical; ductus ejaculatorius with bulbus. **Intraspecific variation**. No female specimen available.

##### Material examined.

Holotype (MZB): ARC1807 (EMBL # HE616084), WEST NEW GUINEA, Jayawijaya Reg., Bokondini, S03°41.787', E138°40.229' to S03°41.778', E138°40.129', 1705–1710 m, 17-VII-2010. Paratype (SMNK): WEST NEW GUINEA, Jayawijaya Reg.: 1 ex, ARC1805 (EMBL # HE616082), Bokondini, S03°40.345', E138°42.386' to S03°40.255', E138°42.189´, 1655–1700 m, 18-VII-2010.

##### Distribution.

Jayawijaya Reg. (Bokondini). Elevation: 1700–1705 m.

##### Biology.

Beaten from foliage of montane forests.

##### Etymology.

This epithet is a combination of the Latin noun *fusus* (spindle) and the suffix *-formis* (-shaped) and refers to the habitus of this species.

##### Notes.

*Trigonopterus fusiformis* Riedel, sp. n. was coded as “*Trigonopterus* sp. 107” by Tänzler et al. (2012).

#### 32. Trigonopterus glaber

Riedel sp. n.

urn:lsid:zoobank.org:act:7FBAF1AD-D9B6-447F-B0D7-F4A2F83F8A43

http://species-id.net/wiki/Trigonopterus_glaber

##### Diagnostic description.

**Figure 32.**
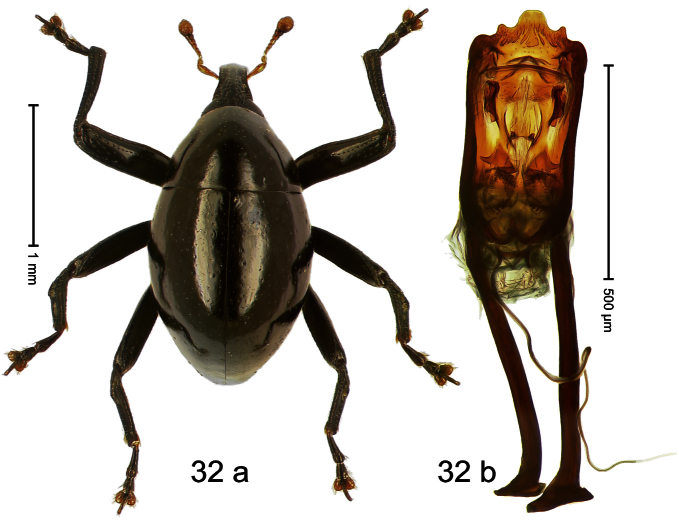
*Trigonopterus glaber* Riedel, sp. n., holotype; (**a**) Habitus (**b**) Aedeagus.

Holotype, male (Fig. 32a). Length 1.98 mm. Color black, antenna and tarsi ferruginous. Body laterally somewhat compressed; ovate; without constriction between pronotum and elytron; in profile almost evenly convex. Rostrum dorsally in basal half with pair of sublateral furrows; medially even, sparsely punctate. Eyes large. Pronotum subglabrous. Elytra subglabrous, laterally sparsely punctate with small punctures, striae indistinct. Femora with anteroventral ridge. Profemur subparallel. Meso- and metafemur with dorsoposterior edge subapically worn; metafemur subapically without stridulatory patch. Tibiae simple, without rows or brushes of long setae. Metaventrite laterally forming acute process over metacoxa, reaching tibial insertion. Metaventrite and abdominal ventrite 1 subglabrous, with sparse recumbent setae. Abdominal ventrite 2 fused to and forming common cavity with ventrite 1. Abdominal ventrite 5 anteriorly with pair of distinct longitudinal protrusions, indistinct median cavity posteriorly open. Aedeagus (Fig. 32b) apically with median extension and with pair of submedian teeth; body with two conspicuous pairs of endophallic sclerites; ductus ejaculatorius without bulbus. **Intraspecific variation**. Length 1.98–2.18 mm. Female rostrum subglabrous except in basal ¼ with ridges. Female abdominal ventrite 5 flat.

##### Material examined.

Holotype (SMNK): ARC0960 (EMBL # HE615593), PAPUA NEW GUINEA, Central Prov., Moroka area, Kailaki, Mt. Berogoro, S09°24.213', E147°33.870' to S09°23.647', E147°34.244', 500–600 m, 20-IX-2009. Paratypes (NAIC, SMNK, ZSM): PAPUA NEW GUINEA, Central Prov.: 1 ex, ARC0961 (EMBL # HE615594), same data as holotype; 8 exx, Moroka area, Kailaki, Mt. Berogoro, S09°24.213', E147°33.870' to S09°23.647', E147°34.244', 500–565 m, 26-X-2009; 4 exx, ARC0942 (EMBL # HE615575), ARC0943 (EMBL # HE615576), Varirata N.P., S09°26.150', E147°21.520' to S09°26.148', E147°21.361', 700–800 m, 19-IX-2009; 16 Ex, ARC0991 (EMBL # HE615624), Moroka area, Kailaki, Wariaga, S09°25.350', E147°31.047' to S09°25.403', E147°31.315', 650–820 m, 27-X-2009; 9 exx, Moroka area, Kailaki, Wariaga, S09°25.350', E147°31.047' to S09°25.683', E147°31.707', 650–920 m, 27-X-2009; 18 exx, Moroka area, Kailaki, Wariaga, S09°25.403', E147°31.315' to S09°25.683', E147°31.707', 820–920 m, 27-X-2009; 5 exx, Moroka area, Kailaki, Beremutana ridge, S09°25.515', E147°33.136' to S09°25.754', E147°33.485', 535–700 m, 28-X-2009, beaten; 5 exx, Moroka area, Kailaki, Beremutana ridge, S09°25.754', E147°33.485' to S09°25.940', E147°33.703', 700–845 m, 28-X-2009, beaten; 2 exx, Moroka area, Kailaki, Beremutana ridge, S09°25.515', E147°33.136' to S09°25.754', E147°33.485', 535–650 m, 28-X-2009, beaten.

##### Distribution.

Central Prov. (Varirata, Moroka). Elevation: 565–820 m.

##### Biology.

Collected by beating foliage in primary forests.

##### Etymology.

This epithet is based on the Latin adjective *glaber* (hairless, bald) and refers to its smooth body.

##### Notes.

*Trigonopterus glaber* Riedel, sp. n. was coded as “*Trigonopterus* sp. 164” by Tänzler et al. (2012).

#### 33. Trigonopterus gonatoceros

Riedel sp. n.

urn:lsid:zoobank.org:act:6B7F6087-236C-4763-9BEF-032FCB98C073

http://species-id.net/wiki/Trigonopterus_gonatoceros

##### Diagnostic description.

**Figure 33.**
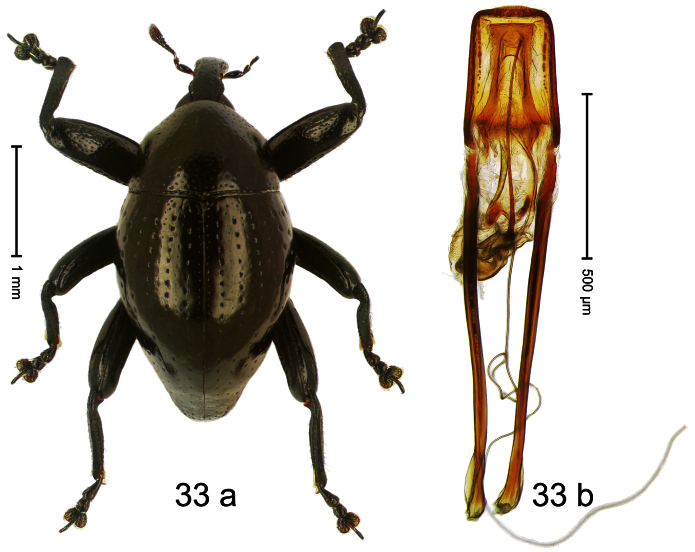
*Trigonopterus gonatoceros* Riedel, sp. n., holotype; (**a**) Habitus (**b**) Aedeagus.

Holotype, male (Fig. 33a). Length 2.90 mm. Color black. Body subovate; almost without constriction between pronotum and elytron; in profile evenly convex. Rostrum dorsally relatively flat, with two indistinct submedian rows of punctures, dorsolaterally with pair of furrows continuing along eye; surface weakly microreticulate. Pronotum sparsely punctate with double-punctures each consisting of two minute approximate punctures. Elytra with striae distinct, dorsally punctures small, laterally large; intervals flat, with row of minute punctures, subglabrous. Femora edentate. Metafemur with simple dorsoposterior edge; subapically with stridulatory patch. Tibial base dentiform, when leg extended tibial tooth overlapping femoral apex dorsally. Thoracic and abdominal venter with dense erect setae. Aedeagus (Fig. 33b) apically subangulate, subglabrous; transfer apparatus flagelliform, 1.5 X longer than body; endophallus with two pairs of sclerites; ductus ejaculatorius without bulbus. **Intraspecific variation**. Length 2.75–2.88 mm. Female venter subglabrous.

##### Material examined.

Holotype (MZB): ARC1776 (EMBL # HE616053), WEST NEW GUINEA, Jayawijaya Reg., Bokondini, S03°40.345', E138°42.386' to S03°40.255', E138°42.189', 1655–1700 m, 18-VII-2010. Paratypes (SMNK, ZSM): 81 exx, ARC1777 (EMBL # HE616054), ARC1778 (EMBL # HE616055), same data as holotype.

##### Distribution.

Jayawijaya Reg. (Bokondini). Elevation: ca. 1655–1700 m.

##### Biology.

Beaten from foliage of montane forests.

##### Etymology.

This epithet is based on a combination of the Greek nouns *gonatos* (knee) and *ceros* (horn) in apposition and refers to the peculiar extensions of the tibial base.

##### Notes.

*Trigonopterus gonatoceros* Riedel, sp. n. was coded as “*Trigonopterus* sp. 121” by Tänzler et al. (2012).

#### 34. Trigonopterus granum

Riedel sp. n.

urn:lsid:zoobank.org:act:08FF1F00-3CCB-4E95-997B-4B170BFF6069

http://species-id.net/wiki/Trigonopterus_granum

##### Diagnostic description.

**Figure 34.**
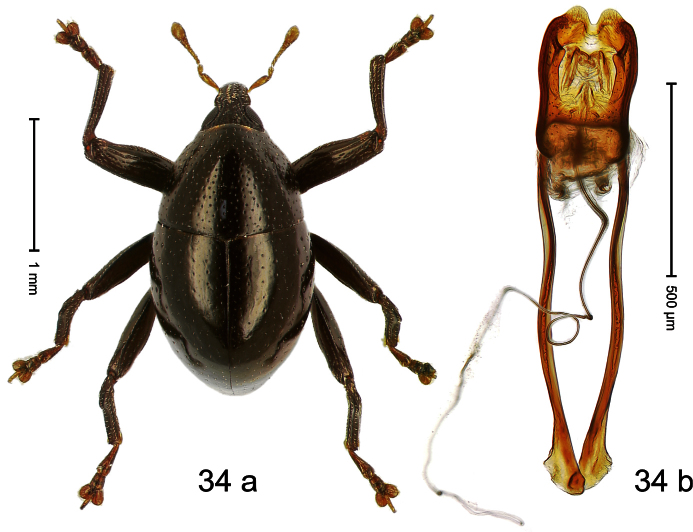
*Trigonopterus granum* Riedel, sp. n., holotype; (**a**) Habitus (**b**) Aedeagus.

Holotype, male (Fig. 34a). Length 2.15 mm. Color black; legs and rostrum deep ferruginous; antenna light ferruginous. Body laterally somewhat compressed, ovate; without constriction between pronotum and elytron; in profile evenly convex. Rostrum dorsally in basal third with low median ridge and pair of submedian ridges; apically subglabrous. Eyes large. Pronotum densely punctate; dorsally punctures small, laterally larger, each with one minute seta; without scales. Elytra dorsally subglabrous, stria 1–2 hardly visible; laterally strial punctures large, relatively shallow. Femora with anteroventral ridge. Profemur converging from base to apex. Meso- and metafemur with dorsoposterior edge in apical third shortened; metafemur subapically without stridulatory patch. Tibiae simple, without rows or brushes of long setae; metatibia subapically with small suprauncal projection. Metaventrite laterally forming acute process over metacoxa, reaching tibial insertion. Metaventrite and abdominal ventrite 1 subglabrous, with sparse recumbent setae. Abdominal ventrite 2 similar to ventrites 3–4. Abdominal ventrite 5 with transverse depression, without distinct cavity; subapically with median denticle. Aedeagus (Fig. 34b) apically sinuate, with deep median incision; ductus ejaculatorius without bulbus. **Intraspecific variation**. Length 1.98–2.48 mm. Female abdominal ventrite 5 almost flat, densely punctate.

##### Material examined.

Holotype (MZB): ARC0441 (EMBL # FN429152), WEST NEW GUINEA, Jayapura Reg., Cyclops Mts, Sentani, S02°32.2', E140°30.4', 545–700 m, 02-XII-2007, beaten. Paratypes (ARC, SMNK, ZSM): WEST NEW GUI-NEA, Jayapura Reg., Cyclops Mts, Sentani: 38 exx, ARC0442 (EMBL # FN429153), ARC0443 (EMBL # FN429154), same data as holotype; 1 ex, ARC0653, S02°32.3', E140°30.4', 350–620 m, 19-XI-2007, beaten; 2 exx, S02°31.8', E140°30.5', 600–900 m, 28-XI-2007; 2 exx, S02°32.0', E140°30.4', 700–900 m, 02-XII-2007; 4 exx, S02°31.6', E140°30.4', 900–1100 m, 28-XI-2007, beaten; 11 exx, 950–1450 m, 03-X-1992; 21 exx, 600–1000 m, 05-X-1991; 2 exx, 800–1000 m, 07-VIII-1992; 6 exx, 300–1400 m, 10-VIII-1991; 5 exx, 400–800 m, 07-VIII-1992; 4 exx, 350–850 m, 16-X-1996; 1 ex, 300–550 m, 02-X-1992.

##### Distribution.

Jayapura Reg. (Cyclops Mts). Elevation: 620–950 m.

##### Biology.

Collected by beating foliage in primary forests.

##### Etymology.

This epithet is based on the Latin noun *granum* (small kernel, seed) in apposition and refers to the general habitus.

##### Notes.

*Trigonopterus granum* Riedel, sp. n. was coded as “*Trigonopterus* sp. 15” by Riedel et al. (2010) and Tänzler et al. (2012), respectively “*Trigonopterus* spo” in the EMBL/GenBank/DDBJ databases. It is closely related to *Trigonopterus pseudogranum* sp. n., *Trigonopterus velaris* sp. n., and *Trigonopterus imitatus* sp. n.; from the latter two it can be distinguished by its sparsely punctate body and the structure of its male abdominal ventrite 5. The externally very similar *Trigonopterus pseudogranum* sp. n. is best separated by the *cox1*-sequence which diverges 12.1 %.

#### 35. Trigonopterus helios

Riedel sp. n.

urn:lsid:zoobank.org:act:A600DE45-0F31-4B37-BDD5-8ADBC6859E05

http://species-id.net/wiki/Trigonopterus_helios

##### Diagnostic description.

**Figure 35.**
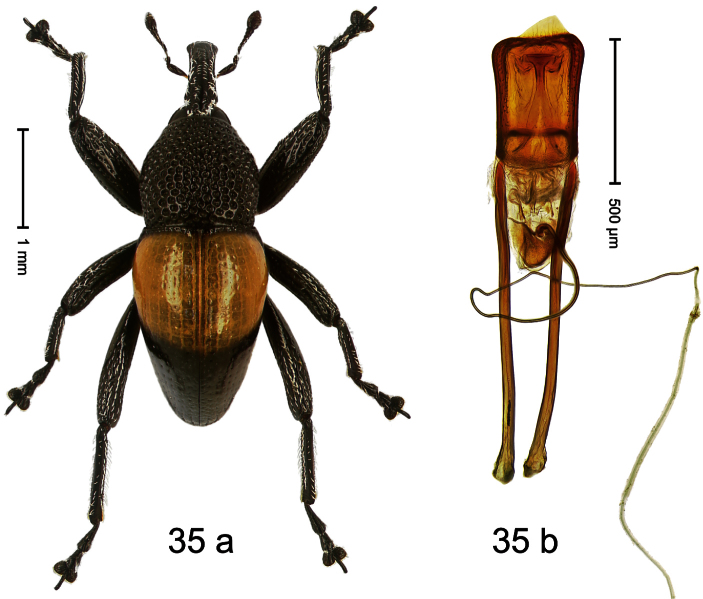
*Trigonopterus helios* Riedel, sp. n., holotype; (**a**) Habitus (**b**) Aedeagus.

Holotype, male (Fig. 35a). Length 3.14 mm. Color black; basal half of elytra with large orange spot. Body elongate-subovate; in dorsal aspect and in profile with distinct constriction between pronotum and elytron. Rostrum slender; dorsally with distinct median costa and pair of narrow submedian ridges; at base with erect white clavate scales, apically replaced by bristles. Eyes large, medially approximate. Pronotum densely punctate-reticulate; sides anteriorly with punctures containing small white scales. Elytra subglabrous, irregularly punctate with minute punctures; striae obsolete; basal margin with ridge extending behind humeri, bordered by row of small indistinct punctures. Femora with tooth in apical half. Mesofemur and metafemur dorsally sparsely squamose with white scales. Metafemur with smooth dorsoposterior edge; subapically without stridulatory patch. Abdominal ventrites 1–2 laterally swollen, medially concave; ventrite 2 posteriorly truncate, posterior face markedly projecting over ventrite 3. Aedeagus (Fig. 35b) subapically slightly widened; apex subangulate, asymmetrical, shifted to left; transfer apparatus dentiform, basally supported by crescent-shaped sclerite; ductus ejaculatorius subapically without bulbus. **Intraspecific variation**. Length 3.27–3.53 mm. Female rostrum dorsally subglabrous, with submedian rows of minute punctures, with lateral furrow containing row of sparse setae. Female abdominal ventrites 1–2 convex.

##### Material examined.

Holotype (SMNK): ARC1831 (EMBL # HE616108), PAPUA NEW GUINEA, Eastern Highlands Prov., Okapa, Kimiagomo village, Afiyaleto, S06°25.593', E145°34.862', S06°25.212', E145°35.498', 1911 m, 18-III-2010. Paratypes (NAIC, SMNK, ZSM): PAPUA NEW GUINEA, Eastern Highlands Prov.: 4 exx, ARC1830 (EMBL # HE616107), ARC1832 (EMBL # HE616109), same data as holotype; 1 ex, Okapa, Afiyaleto village, S06°25.593', E145°34.862', 1940 m, 18-III-2010, beaten.

##### Distribution.

Eastern Highlands Prov. (Okapa). Elevation: 1911–1940 m.

##### Biology.

Beaten from foliage of montane forest.

##### Etymology.

This epithet is based on the Greek noun *helios* (sun) in apposition and refers to the bright orange spot on the elytra.

##### Notes.

*Trigonopterus helios* Riedel, sp. n. was coded as “*Trigonopterus* sp. 136” by Tänzler et al. (2012).

#### 36. Trigonopterus hitoloorum

Riedel sp. n.

urn:lsid:zoobank.org:act:23025BDD-B5CD-4FF7-A6B3-04B8233ADA4A

http://species-id.net/wiki/Trigonopterus_hitoloorum

##### Diagnostic description.

**Figure 36.**
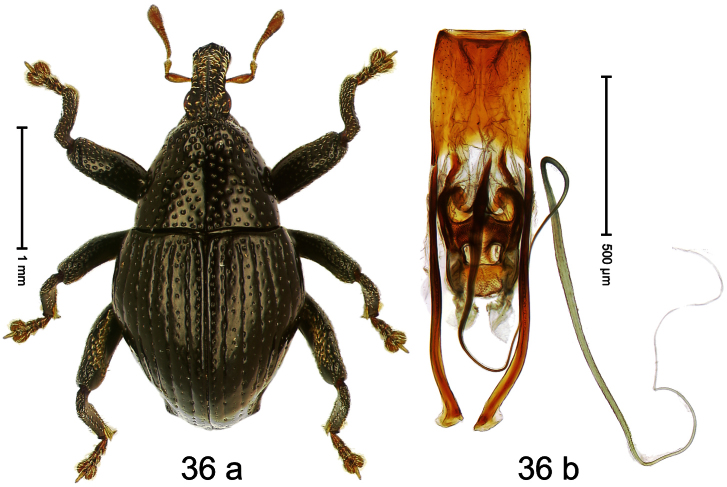
*Trigonopterus hitoloorum* Riedel, sp. n., holotype; (**a**) Habitus (**b**) Aedeagus.

Holotype, male (Fig. 36a). Length 2.64 mm. Color black; tarsi and antenna ferruginous. Body subovate; with weak constriction between pronotum and elytron; in profile evenly convex. Rostrum sparsely squamose with suberect scales; in basal half with median carina; in front of eyes with flat lateral extensions; subapically weakly scabrous; epistome smooth, posteriorly forming transverse, angulate ridge, with weak median denticle. Pronotum without subapical constriction, densely coarsely punctate; laterally above procoxa with fovea. Elytra with striae moderately incised on disk; intervals flat, with rows of small punctures; laterally subglabrous; apex subangulate, with pair of sublateral knobs; extended ventrad, slightly beak-shaped. Femora edentate. Metafemur dorsally sparsely squamose, with weakly denticulate dorsoposterior edge; subapically with stridulatory patch. Abdominal ventrite 2 projecting dentiform over elytral edge in profile. Aedeagus (Fig. 36b) apically rounded, sparsely setose; with complex, symmetrical transfer apparatus; ductus ejaculatorius with bulbus. **Intraspecific variation**. Length 2.28–2.64 mm. Female rostrum with median carina and transverse ridge of epistome less distinct.

##### Material examined.

Holotype (SMNK): ARC1172 (EMBL # HE615800), PAPUA NEW GUINEA, Morobe Prov., Huon peninsula, Mindik, S06°27.311', E147°24.073', 1570 m, 10-X-2009. Paratypes (NAIC): Morobe Prov., Huon peninsula, Mindik: 1 ex, ARC1176 (EMBL # HE615804), S06°27.221', E147°24.185', 1670 m, 10-X-2009.

##### Distribution.

Morobe Prov. (Mindik). Elevation: 1570–1670 m.

##### Biology.

Sifted from leaf litter in montane forest.

##### Etymology.

This species is dedicated to the people of Papua New Guinea. The epithet is based on the family name Hitolo, found on page 221 of the Papua New Guinea Telephone Directory of 2010 and treated in genitive plural.

##### Notes.

*Trigonopterus hitoloorum* Riedel, sp. n. was coded as “*Trigonopterus* sp. 195” by Tänzler et al. (2012).

#### 37. Trigonopterus imitatus

Riedel sp. n.

urn:lsid:zoobank.org:act:F0B9CC05-E2E0-4B7F-8A82-8246B16F69FE

http://species-id.net/wiki/Trigonopterus_imitatus

##### Diagnostic description.

**Figure 37.**
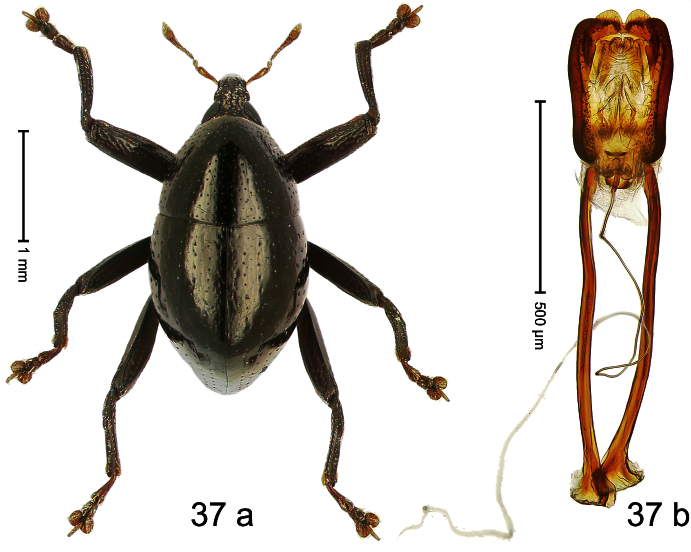
*Trigonopterus imitatus* Riedel, sp. n., holotype; (**a**) Habitus (**b**) Aedeagus.

Holotype, male (Fig. 37a). Length 2.58 mm. Color black; legs and rostrum deep ferruginous; antenna light ferruginous. Body laterally somewhat compressed, ovate, without constriction between pronotum and elytron; in profile evenly convex. Rostrum dorsally in basal third punctate-rugose; apically subglabrous. Eyes large. Pronotum densely punctate, punctures dorsally small, laterally becoming larger, bearing each one minute seta; without scales. Elytra dorsally subglabrous, stria 1–2 hardly visible; laterally strial punctures large, relatively shallow. Profemur converging from base to apex. Meso- and metafemur with in apical 1/3 with anteroventral ridge terminating as tooth. Metafemur subapically without stridulatory patch. Tibiae simple, without rows or brushes of long setae. Metaventrite laterally forming acute process over metacoxa, reaching tibial insertion. Metaventrite and abdominal ventrite 1 with long erect setae, especially near mesocoxa. Abdominal ventrite 2 similar to ventrites 3–4. Abdominal ventrite 5 with deep, transversely ovate cavity; basal third simple, swollen; laterally setose. Aedeagus (Fig. 37b) apically sinuate, with deep narrow median incision; ductus ejaculatorius without bulbus. **Intraspecific variation**. Length 1.98–2.58 mm. Female rostrum subglabrous except in basal ¼ with ridges. Female venter sparsely setose, abdominal ventrite 5 flat.

##### Material examined.

Holotype (MZB): ARC1697 (EMBL # HE615984), WEST NEW GUINEA, Biak Reg., Supiori Isl., Korido, S00°49.715', E135°35.055', 50–100 m, 9-VII-2010, beaten. Paratypes (SMNK, ZSM): 4 exx, ARC1698 (EMBL # HE615985), ARC1699 (EMBL # HE615986), same data as holotype.

##### Distribution.

Biak Reg. (Supiori Isl.). Elevation: ca. 50–100 m.

##### Biology.

Collected by beating foliage in primary forests.

##### Etymology.

This epithet is based on the Latin participle *imitatus* (imitated) and refers to its morphological similarity with sibling species.

##### Notes.

*Trigonopterus imitatus* Riedel, sp. n. was coded as “*Trigonopterus* sp. 273” by Tänzler et al. (2012). It is closely related to *Trigonopterus granum* sp. n., *Trigonopterus pseudogranum* sp. n., and *Trigonopterus velaris* sp. n. from which it can be distinguished by the male venter with long setae. Despite its close morphological similarity its *cox1*-sequence diverges 9.9–12.3 % from the other species.

#### 38. Trigonopterus inflatus

Riedel sp. n.

urn:lsid:zoobank.org:act:8AE401E3-76B5-4768-8267-377F1BFA5695

http://species-id.net/wiki/Trigonopterus_inflatus

##### Diagnostic description.

**Figure 38.**
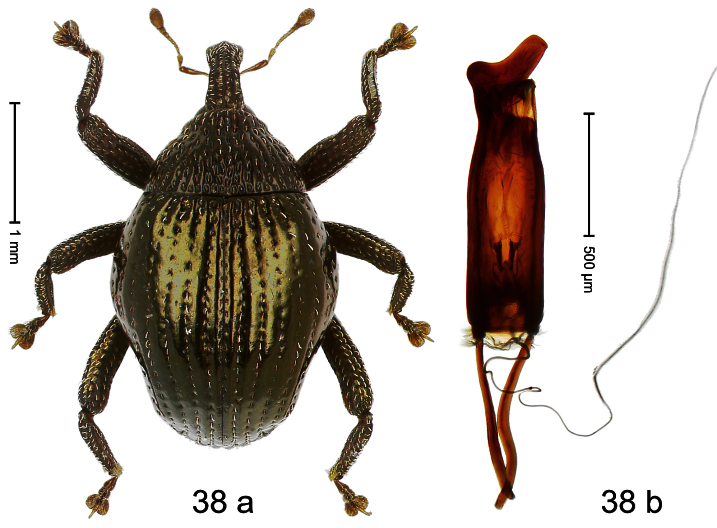
*Trigonopterus inflatus* Riedel, sp. n., holotype; (**a**) Habitus (**b**) Aedeagus.

Holotype, male (Fig. 38a). Length 2.90 mm. Color black, elytra with greenish-bronze lustre; antenna light ferruginous, legs dark ferruginous. Body subovate; in dorsal aspect and in profile almost without constriction between pronotum and elytron. Rostrum with distinct median and pair of submedian carinae, in apical third scabrous; epistome simple. Pronotum relatively small, with weak subapical constriction; disk densely punctate, longitudinally rugose; each puncture with seta, a few with scale. Elytra with striae deeply impressed; with sparse, yellowish scales; intervals subglabrous. Meso- and metafemur edentate. Metafemur with dorsoposterior edge serrate, subapically without stridulatory patch. Metatibia apically with blunt premucro. Onychium ca. 1.1× longer than tarsomere 3. Aedeagus (Fig. 38b) with apex asymmetrical, right side forming biramose extension; basal orifice ventrally with rim; transfer apparatus spiniform, curved, directed basad; ductus ejaculatorius without bulbus. **Intraspecific variation**. Length 2.45–2.90 mm. Female rostrum dorsally subglabrous, with dense small punctures, in basal third with longitudinal ridges. Female metatibia apically without premucro.

##### Material examined.

Holotype (MZB): ARC0858 (EMBL # HE615540), WEST NEW GUINEA, Biak Isl., Korim, Nernu, S00°55.784', E136°01.530', 165 m, 15-XII-2007, sifted. Paratypes (ARC, SMNK, ZSM): WEST NEW GUINEA, Biak Isl.: 28 exx, ARC0859 (EMBL # HE615541), same data as holotype; 1 ex, ARC0883 (EMBL # HE615565), Korim, Nernu, S00°55.888', E136°01.671', 180 m, 15-XII-2007, sifted; 3 exx (1 marked ARC0093), Korim, Nernu, 150 m, 20-I-2005, sifted.

##### Distribution.

Biak Isl.. Elevation: 150–180 m.

##### Biology.

Sifted from leaf litter in lowland forest.

##### Etymology.

This epithet is based on the Latin participle *inflatus* (swollen) and refers to the shape of the elytra that appear somewhat disproportionate in relation to the pronotum.

##### Notes.

*Trigonopterus inflatus* Riedel, sp. n. was coded as “*Trigonopterus* sp. 63” by Tänzler et al. (2012).

#### 39. Trigonopterus insularis

Riedel sp. n.

urn:lsid:zoobank.org:act:493867A3-E874-4AC7-9BFD-CB42BB34EFC2

http://species-id.net/wiki/Trigonopterus_insularis

##### Diagnostic description.

**Figure 39.**
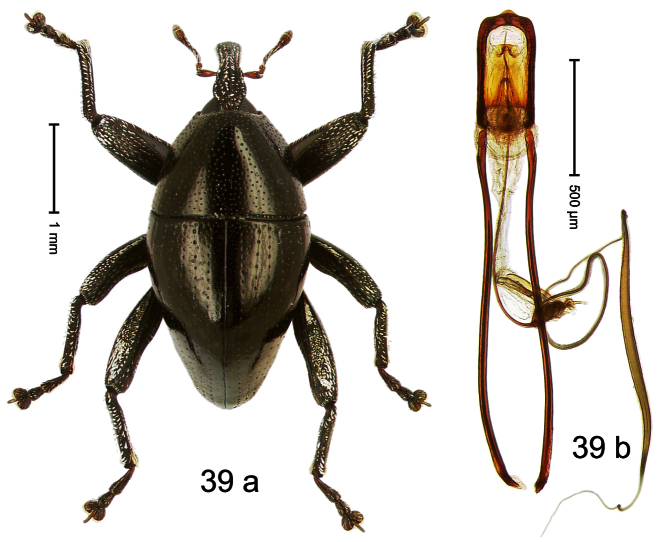
*Trigonopterus insularis* Riedel, sp. n., holotype; (**a**) Habitus (**b**) Aedeagus.

Holotype, male (Fig. 39a). Length 3.41 mm. Color black; antenna and tarsi ferruginous. Body subovate; without constriction between pronotum and elytron; in profile evenly convex. Rostrum in basal half with distinct median carina and pair of indistinct sublateral ridges; with sparse rows of white scales; subapical third subglabrous, weakly punctate; in front of antennal insertion with weak constriction. Eyes large, medially approximate. Pronotum densely punctate with small punctures; sides separated by indistinct edge bearing dense row of punctures; laterally above coxa sparsely squamose with white scales. Elytral striae distinct with small punctures; intervals flat, subglabrous, sparsely punctate with minute punctures; basal margin straight, simple. Femora with anteroventral ridge distinct, at base abruptly ending and forming markedly projecting blunt angle; edentate. Mesofemur and metafemur dorsally densely squamose with white scales but partly abraded. Metafemur with smooth dorsoposterior edge; subapically without stridulatory patch. Aedeagus (Fig. 39b) with apodemes ca. 3× as long as body; sides of body in basal half subparallel, markedly sclerotized, mid-portion of body weakly sclerotized; apex subangulate; transfer apparatus flagelliform, longer than body; ductus ejaculatorius subapically with bulbus. **Intraspecific variation**. Length 3.38–3.41 mm. No female specimen available.

##### Material examined.

Holotype (MZB): ARC1694 (EMBL # HE615981), WEST NEW GUINEA, Biak Reg., Supiori Isl., Korido, S00°49.715´, E135°35.055´, 50–100 m, 09-VII-2010. Paratype (SMNK): 1 ex, ARC1695 (EMBL # HE615982), same data as holotype.

##### Distribution.

Biak Reg. (Supiori Isl.). Elevation: ca. 50–100 m.

##### Biology.

Beaten from foliage of lowland forest.

##### Etymology.

This epithet is based on the Latin adjective *insularis* (of an island) and refers to the type locality, Supiori Island.

##### Notes.

*Trigonopterus insularis* Riedel, sp. n. was coded as “*Trigonopterus* sp. 57” by Tänzler et al. (2012).

#### 40. Trigonopterus irregularis

Riedel sp. n.

urn:lsid:zoobank.org:act:475C5E3F-9BD7-4BAA-A617-C25E7A754C92

http://species-id.net/wiki/Trigonopterus_irregularis

##### Diagnostic description.

**Figure 40.**
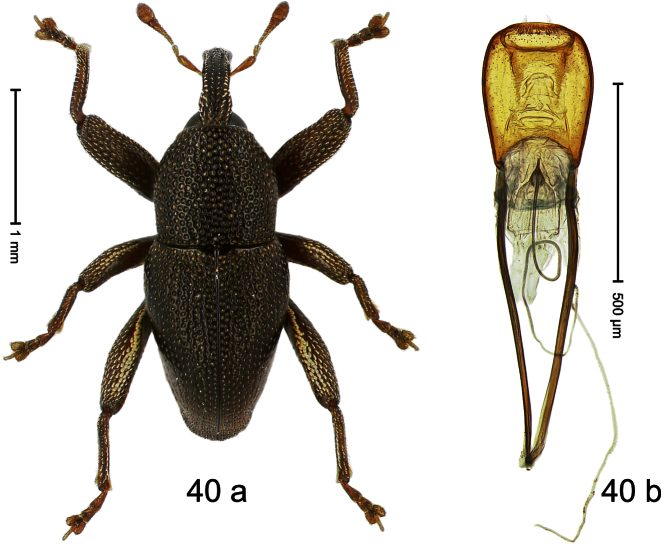
*Trigonopterus irregularis* Riedel, sp. n., holotype; (**a**) Habitus (**b**) Aedeagus.

Holotype, male (Fig. 40a). Length 2.36 mm. Legs ferruginous, antenna light ferruginous, remainder black except elytral apex deep ferruginous, especially along sides. Body elongate; with distinct constriction between pronotum and elytron; in profile dorsally flat. Rostrum with distinct median and pair of submedian carinae, in apical ¼ relatively smooth. Pronotum densely punctate-reticulate, interspaces smaller than puncture´s diameter. Elytra dorsally with dense punctation confused, partly reticulate; striae laterally impressed as fine lines; basal margin medially bordered by narrow glabrous band. Femora edentate. Profemur in basal third posteriorly with callus. Meso- and metafemur on dorsal edge densely squamose with cream-colored scales. Metafemur subapically with indistinct stridulatory patch. Aedeagus (Fig. 40b) with sides curved, apex rounded; body flattened, transfer apparatus spiniform; ductus ejaculatorius without bulbus. **Intraspecific variation**. Length 2.34–2.59 mm. Female rostrum dorsally subglabrous, with pair of submedian rows of large punctures.

##### Material examined.

Holotype (MZB): ARC0780 (EMBL # HE615463), WEST NEW GUINEA, Manokwari, Arfak Mts, S01°03.723', E133°54.145', 1385 m, 08-XII-2007. Paratypes (ARC, SMNK, ZSM): WEST NEW GUINEA, Manokwari, Mokwam, Siyoubrig: 1 ex, ARC0804 (EMBL # HE615487), S01°06.107', E133°54.888', 1530 m, 10-XII-2007, beaten; 1 ex, ARC0851 (EMBL # HE615533), S01°06.7', E133°54.6', 1580–1750 m, 11-XII-2007; 1 ex, Minyambou – Mokwam, 1300–1900 m, 16-IV-1993; 2 exx, Mokwam, Kwau, 1300–1650 m, 17-IV-1993.

##### Distribution.

Manokwari Reg. (Arfak Mts). Elevation: 1385–1580 m.

##### Biology.

Collected by beating foliage in montane forests.

##### Etymology.

This epithet is based on the Latin adjective *irregularis* (irregular) and refers to the elytral punctation.

##### Notes.

*Trigonopterus irregularis* Riedel, sp. n. was coded as “*Trigonopterus* sp. 247” by Tänzler et al. (2012).

#### 41. Trigonopterus ixodiformis

Riedel sp. n.

urn:lsid:zoobank.org:act:53B538F7-D7F3-47C9-9A95-5BC50A01AC31

http://species-id.net/wiki/Trigonopterus_ixodiformis

##### Diagnostic description.

**Figure 41.**
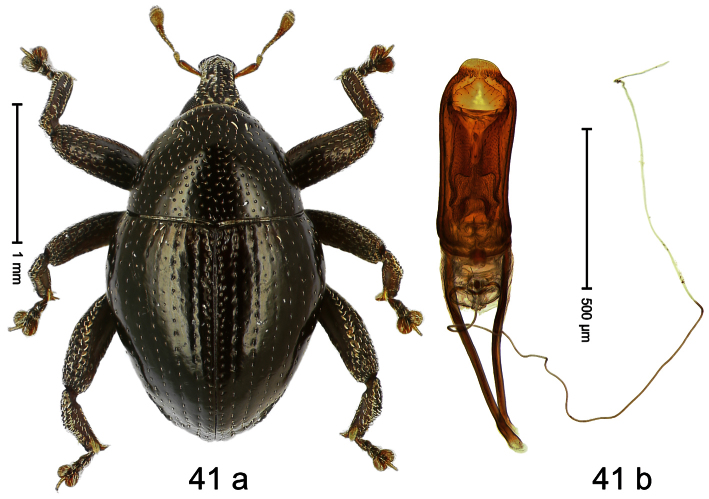
*Trigonopterus ixodiformis* Riedel, sp. n., holotype; (**a**) Habitus (**b**) Aedeagus.

Holotype, male (Fig. 41a). Length 2.39 mm. Color black; elytral base and legs dark ferruginous, antenna light ferruginous. Body subovate, with shallow constriction between pronotum and elytron; in profile almost evenly convex. Rostrum scabrous, basally with indistinct median ridge; epistome forming angulate ridge. Pronotum moderately densely punctate. Elytra with striae marked by small punctures; interval 4 basally with cluster of few narrow, cream-colored, recumbent scales; interval 7 subapically forming indistinct ridge. Meso- and metafemur with anteroventral ridge distinctly dentate. Meso- and metatibia in basal half widened; dorsal edge basally denticulate; subapically narrowed; uncus large, peg-shaped. Metafemur with serrate dorsoposterior edge, subapically without stridulatory patch. Venter subglabrous, concave. Aedeagus (Fig. 41b) with sides of body in apical third converging, apex rounded; orifice retracted; endophallus weakly granulate; transfer apparatus symmetrical, heart-shaped frame relatively thin; ductus ejaculatorius without bulbus. **Intraspecific variation**. Length 1.75–2.39 mm. Female rostrum dorsally subglabrous, sparsely punctate; epistome simple.

##### Material examined.

Holotype (MZB): ARC0762 (EMBL # HE615445), WEST NEW GUINEA, Manokwari, Mt. Meja, S00°51.497', E134°04.949', 220 m, 05-XII-2007, sifted. Paratypes (ARC, SMNK, ZSM): WEST NEW GUINEA, Manokwari: 23 exx, ARC0763 (EMBL # HE615446), ARC0764 (EMBL # HE615447), same data as holotype; 4 exx, Mt. Meja, S00°51.400', E134°04.918', 225 m, 06-XII-2007, sifted; 16 exx, Mt. Meja, 200 m, 30-XII-2004, sifted; 6 exx, Mt. Meja, 200 m, 30-XII-2000, sifted; 9 exx, Mt. Meja, 22-23-IX-1990, sifted; 6 exx, Mt. Meja, 200 m, 19-IV-1993; 1 ex, ARC0772 (EMBL # HE615455), Arfak Mts, S01°01.465', E133°54.243', 685 m, 08-XII-2007, sifted.

##### Distribution.

Manokwari Reg. (Mt. Meja, Arfak Mts). Elevation: 200–685 m.

##### Biology.

Sifted from leaf litter in lowland forest.

##### Etymology.

This epithet is a combination of the name Ixodidae and the Latin suffix *-formis* (having the form of) and refers to the species´ superficial resemblance with ticks.

##### Notes.

*Trigonopterus ixodiformis* Riedel, sp. n. was coded as “*Trigonopterus* sp. 222” by Tänzler et al. (2012).

#### 42. Trigonopterus kanawiorum

Riedel sp. n.

urn:lsid:zoobank.org:act:AF3FBA36-231F-4BF0-AE98-F2D28A70D967

http://species-id.net/wiki/Trigonopterus_kanawiorum

##### Diagnostic description.

**Figure 42.**
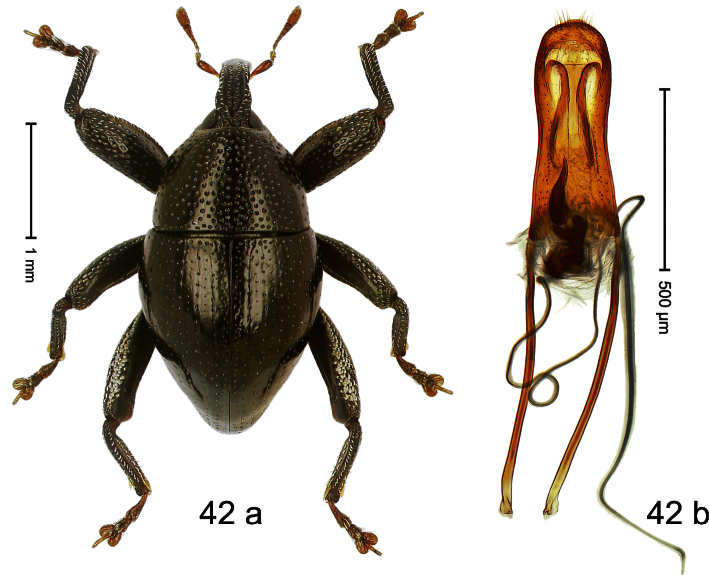
*Trigonopterus kanawiorum* Riedel, sp. n., holotype; (**a**) Habitus (**b**) Aedeagus.

Holotype, male (Fig. 42a). Length 2.68 mm. Color black; antenna and tarsi ferruginous. Body ovate; in dorsal aspect with weak constriction between pronotum and elytron; in profile evenly convex. Rostrum dorsally with median ridge and pair of submedian ridges, furrows with sparse rows of yellowish scales; in apical 1/3 weakly punctate-rugose. Pronotum densely punctate. Elytra densely punctate with minute punctures, especially on intervals; strial punctures slightly larger; striae impressed as fine lines; lateral stria behind humeri simple, not deepened. Femora edentate. Mesofemur and metafemur dorsally densely squamose with silvery scales. Metafemur with denticulate dorsoposterior edge; subapically with stridulatory patch. Metatibia apically with uncus, without premucro. Abdominal ventrite 5 tomentose. Aedeagus (Fig. 42b) symmetrical; apex subangulate, sparsely setose; body at middle slightly constricted; transfer-apparatus asymmetrical, spiniform; ductus ejaculatorius with bulbus. **Intraspecific variation**. Length 2.45–2.76 mm. Female rostrum dorsally in apical half flattened, subglabrous, with submedian rows of small punctures. Female abdominal ventrite 5 with sparse recumbent scales.

##### Material examined.

Holotype (SMNK): ARC1133 (EMBL # HE615762), PAPUA NEW GUINEA, Simbu Prov., Karimui Dist., Haia, Supa, S06°40.078', E145°03.207' to S06°39.609', E145°03.012', 1220–1450 m, 02-X-2009. Paratypes (NAIC, SMNK, ZSM): PAPUA NEW GUINEA, Simbu Prov.: 8 exx, ARC1134 (EMBL # HE615763), same data as holotype; 2 exx, ARC1149 (EMBL # HE615777), ARC1150 (EMBL # HE615778), Haia, Supa, S06°40.047', E145°03.464' to S06°40.078', E145°03.207', 1075–1220 m, 02-X-2009; 7 exx, Haia, Supa station, S06°39.905', E145°03.880' to S06°39.796', E145°03.873', 1220–1320 m, 01-X-2009, beaten; 1 ex, ARC1142 (EMBL # HE615770), Haia, Supa, S06°40.047', E145°03.464' to S06°39.815', E145°03.169', 1075–1240 m, 30-IX-2009; 24 exx, ARC1168 (EMBL # HE615796), Haia, S06°41.216', E145°00.945' to S06°40.976', E145°00.979', 970–1135 m, 04-X-2009; 1 ex, ARC1083 (EMBL # HE615714), Haia, S06°41.553', E145°00.355' to S06°41.624', E145°00.728', 800–960 m, 25-IX-2009; 9 exx, Haia, S06°41.259', E145°00.822' to S06°41.102', E145°00.979', 900–1005 m, 27-IX-2009; 4 exx, Haia, S06°41.102', E145°00.979', 1005–1020 m, 27-IX-2009, beaten, “Mimikry-sample”; 10 exx, Haia, S06°41.102', E145°00.979' to S06°40.976', E145°00.979', 1020–1135m, 27-IX-2009, beaten.

##### Distribution.

Simbu Prov. (Haia). Elevation: 960–1220 m.

##### Biology.

Collected by beating foliage in primary forest.

##### Etymology.

This species is dedicated to the people of Papua New Guinea. The epithet is based on the family name Kanawi, found on page 236 of the Papua New Guinea Telephone Directory of 2010 and treated in genitive plural.

##### Notes.

*Trigonopterus kanawiorum* Riedel, sp. n. was coded as “*Trigonopterus* sp. 209” by Tänzler et al. (2012).

#### 43. Trigonopterus katayoi

Riedel sp. n.

urn:lsid:zoobank.org:act:3C9EA32F-2234-49F8-8D1A-F3CA5336C632

http://species-id.net/wiki/Trigonopterus_katayoi

##### Diagnostic description.

**Figure 43.**
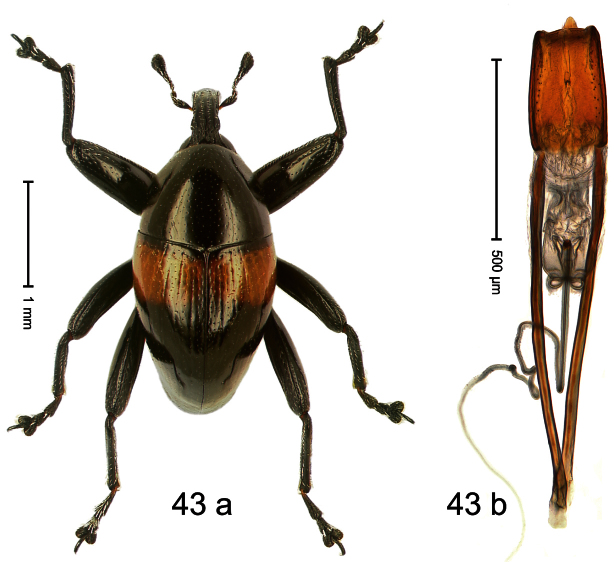
*Trigonopterus katayoi* Riedel, sp. n., holotype; (**a**) Habitus (**b**) Aedeagus.

Holotype, male (Fig. 43a). Length 2.68 mm. Color black, elytra in basal third orange. Body slender, ovate; without constriction between pronotum and elytron; in profile evenly convex. Rostrum in apical third smooth; in basal 2/3 with median ridge and pair of sublateral ridges; furrows containing sparse row of setae. Eyes with dorsal margin carinate. Head bordering eye with bean-shaped impression. Pronotum subglabrous, sparsely punctate with minute punctures. Elytra subglabrous, punctation confused with minute punctures; striae hardly visible, impressed as very fine lines; lateral stria behind humeri with row of ca. 5 deep punctures. Femora subglabrous, including dorsum of metafemur without scales. Mesofemur on posterior surface with longitudinal ridge. Metafemur on posterior surface with indistinct longitudinal impression; dorsoposteriorly simple, subapically without stridulatory patch. Meso- and metatibia subapically simple, with uncus, without premucro. Aedeagus (Fig. 43b). Apex symmetrical, with median acute extension; transfer apparatus small, dentiform; endophallus without distinct sclerites; ductus ejaculatorius without bulbus. **Intraspecific variation**. Length 2.50–2.93 mm. Color of elytral base orange in specimens from Goroka, dark ferruginous in specimens from Okapa.

##### Material examined.

Holotype (SMNK): ARC1052 (EMBL # HE615683), PAPUA NEW GUINEA, Eastern Highlands Prov., Goroka, Mt. Gahavisuka, S06°00.864', E145°24.779', 2150–2250 m, 22-IX-2009. Paratypes (NAIC, SMNK, ZSM): PAPUA NEW GUINEA, Eastern Highlands Prov.: 2 exx, ARC1051 (EMBL # HE615682), ARC1053 (EMBL # HE615684), same data as holotype; 3 exx, Goroka, Mt. Gahavisuka, S06°00.864', E145°24.779', 2150–2280 m, 24-X-2009; 2 exx, ARC1836 (EMBL # HE616113), ARC1837 (EMBL # HE616114), Okapa, Konafi to Isimomo, S06°25.593', E145°34.862', 1911–2131 m, 18-III-2010; 2 exx, ARC1838 (EMBL # HE616115), Okapa, Kimiagomo village, Afiyaleto, S06°25.593', E145°34.862' to S06°25.212', E145°35.498', 1911 m, 18-III-2010.

##### Distribution.

Eastern Highlands Prov. (Goroka, Okapa). Elevation: 1911–2150 m.

##### Biology.

Beaten from foliage of montane forests.

##### Etymology.

This species is named in honour of our colleague Katayo Sagata (Goroka) who greatly supported our field-work in PNG and who collected some of the specimens.

##### Notes.

*Trigonopterus katayoi* Riedel, sp. n. was coded as “*Trigonopterus* sp. 97” by Tänzler et al. (2012).

#### 44. Trigonopterus koveorum

Riedel sp. n.

urn:lsid:zoobank.org:act:0C660759-395A-4536-92ED-7BBD31110121

http://species-id.net/wiki/Trigonopterus_koveorum

##### Diagnostic description.

**Figure 44.**
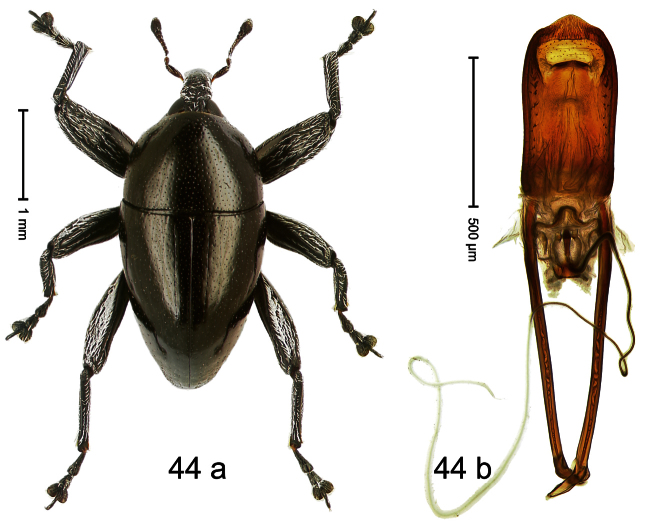
*Trigonopterus koveorum* Riedel, sp. n., holotype; (**a**) Habitus (**b**) Aedeagus.

Holotype, male (Fig. 44a). Length 3.13 mm. Color black; antennal scape and funicle ferruginous. Body ovate; with weak constriction between pronotum and elytron; in profile evenly convex. Rostrum dorsally densely punctate, without ridges or furrows, towards apex punctures becoming minute; laterally and at base with white scales. Pronotum densely punctate; dorsally punctures minute, laterally larger, anterolaterally with scattered white scales. Elytra dorsally with minute, confused punctures, subglabrous; laterally strial punctures sparse but deeply impressed. Femora edentate. Meso- femur and metafemur dorsally densely squamose with white scales. Metafemur with smooth dorsoposterior edge; subapically without stridulatory patch. Metatibia apically with uncus, without premucro. Abdominal ventrites 1–2 weakly concave. Aedeagus (Fig. 44b) symmetrical; apex subangulate, subglabrous; transfer-apparatus simple, dentiform; ductus ejaculatorius without bulbus. **Intraspecific variation**. Length 2.95–3.41 mm. Female rostrum in apical 2/3 dorsally subglabrous. Female abdominal ventrites 1–2 flat.

##### Material examined.

Holotype (SMNK): ARC1846 (EMBL # HE616123), PAPUA NEW GUINEA, Eastern Highlands Prov., Okapa, Kimiagomo village, Hamegoya, S06°25.727', E145°35.455', S06°25.117', E145°35.225', 1891–2131 m, 18-III-2010. Paratypes (NAIC, SMNK, ZSM): 4 exx, ARC1847 (EMBL # HE616124), ARC1848 (EMBL # HE616125), same data as holotype.

##### Distribution.

Eastern Highlands Prov. (Okapa). Elevation: ca. 1891–2131 m.

##### Biology.

Beaten from foliage of montane forests.

##### Etymology.

This species is dedicated to the people of Papua New Guinea. The epithet is based on the family name Kove, found on page 248 of the Papua New Guinea Telephone Directory of 2010 and treated in genitive plural.

##### Notes.

*Trigonopterus koveorum* Riedel, sp. n. was coded as “*Trigonopterus* sp. 149” by Tänzler et al. (2012).

#### 45. Trigonopterus kurulu

Riedel sp. n.

urn:lsid:zoobank.org:act:E6CF5ADF-BFC3-4B89-8AFF-16FF8DC9153A

http://species-id.net/wiki/Trigonopterus_kurulu

##### Diagnostic description.

**Figure 45.**
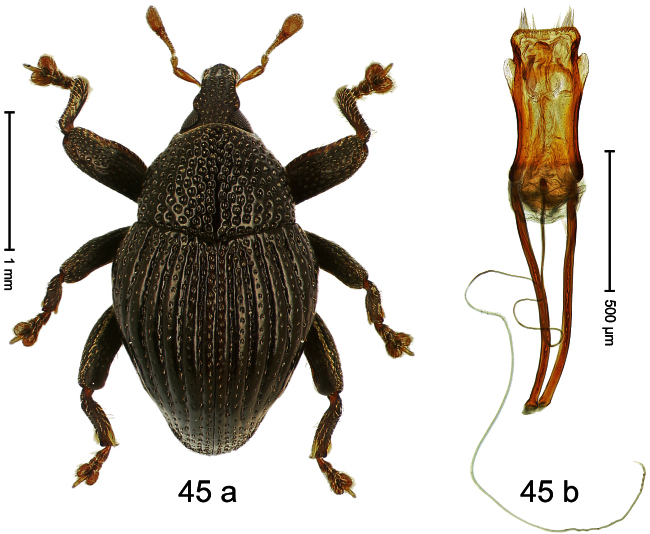
*Trigonopterus kurulu* Riedel, sp. n., holotype; (**a**) Habitus (**b**) Aedeagus.

Holotype, male (Fig. 45a). Length 2.43 mm. Color black; antenna and tarsi light ferruginous; tibiae and femora deep ferruginous. Body subrhomboid; in dorsal aspect with marked constriction between pronotum and elytron; in profile almost evenly convex, with shallow constriction. Rostrum rugose-punctate; epistome forming indistinct, angulate ridge. Pronotum basally angulate, densely punctate with deep punctures; each with one small, upcurved seta. Elytra with striae deeply incised; intervals costate, each with one row of small punctures. Metafemur with simple dorsoposterior edge, subapically with stridulatory patch. Aedeagus (Fig. 45b) with apex subtruncate, setose; subapically sides with pair of membranous protrusions; transfer apparatus minute; ductus ejaculatorius without bulbus. **Intraspecific variation**. Length 2.26–2.73 mm. Female rostrum with epistome simple.

##### Material examined.

Holotype (MZB): ARC0733 (EMBL # HE615416), WEST NEW GUINEA, Jayawijaya Reg., Jiwika, Kurulu, S03°57.161', E138°57.357', 1875 m, 24-XI-2007, sifted. Paratypes (ARC, SMNK, ZSM): WEST NEW GUINEA, Jayawijaya Reg.: 11 exx, ARC0734 (EMBL # HE615417), ARC0735 (EMBL # HE615418), same data as holotype; 6 exx, Jiwika, Kurulu, S03°56.481', E138°57.073', 2070 m, 26-XI-2007, sifted; 2 exx, Jiwika, Kurulu, S03°56.289', E138°57.622', 2200 m, 26-XI-2007, sifted; 25 exx, S03°57.161', E138°57.357', 1875 m, 11-VII-2010, sifted; 4 exx, ca. 1700–2300 m, 02-IX-1991, sifted; 3 exx (1 marked ARC0002), ca. 1700–2300 m, 06-IX-1991, sifted; 2 exx, 2300 m, 06-IX-1991, sifted; 1 ex, 2300 m, 1992, sifted; 6 exx, 1900–2000 m, 23-IX-1992, sifted; 7 exx, 1900–2050 m, 24-X.1993; 2 exx, ARC1716 (EMBL # HE615996), ARC1717 (EMBL # HE615997), Poga, S03°48.382', E138°34.780', 2330 m, 13-VII-2010.

##### Distribution.

Jayawijaya Reg. (Jiwika, Poga). Elevation: 1875–2330 m.

##### Biology.

Sifted from leaf litter in montane forest.

##### Etymology.

This epithet is a noun in apposition. Kurulu, who lived near Jiwika, was a famous Dani leader in the 1960´s. The district around Jiwika bears his name today. The species´ name refers both to the type locality and is a reference to the disappearing culture of the indigenous people of this area.

##### Notes.

*Trigonopterus kurulu* Riedel, sp. n. was coded as “*Trigonopterus* sp. 226” by Tänzler et al. (2012).

#### 46. Trigonopterus lekiorum

Riedel sp. n.

urn:lsid:zoobank.org:act:461E47B8-1A15-4A4E-BDF8-AD6461576CC6

http://species-id.net/wiki/Trigonopterus_lekiorum

##### Diagnostic description.

**Figure 46.**
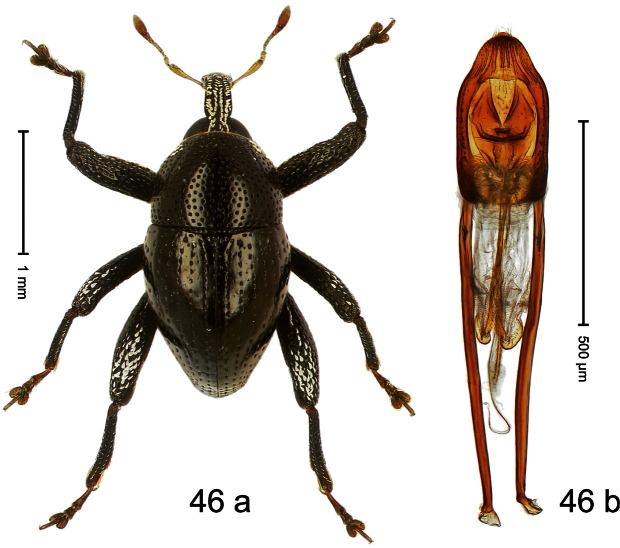
*Trigonopterus lekiorum* Riedel, sp. n., holotype; (**a**) Habitus (**b**) Aedeagus.

Holotype, male (Fig. 46a). Length 2.19 mm. Color black; antenna and tarsi ferruginous. Body subovate; in dorsal aspect and in profile with distinct constriction between pronotum and elytron. Eyes large. Rostrum basally with median and pair of submedian ridges; median and lateral pairs of longitudinal furrows each with row of overlapping almond-shaped white scales. Pronotum densely punctate; anterolaterally with scattered white scales. Elytra with striae distinct; intervals flat, subglabrous. Profemur edentate, mesofemur with small blunt tooth, metafemur with small acute tooth. Mesofemur and metafemur dorsally sparsely squamose with white scales. Metafemur subapically with stridulatory patch. Metatibia subapically ventrally concave, without brush of long setae; uncus hook-like extended, curved ventrobasad. Aedeagus (Fig. 46b) with apodemes 2.0 X as long as body; sides of body in basal half subparallel; apex extended, pointed, markedly curved ventrad; transfer apparatus flagelliform, longer than body of aedeagus; ductus ejaculatorius without bulbus. **Intraspecific variation**. Length 2.18–2.30 mm. Female rostrum dorsally in apical half subglabrous, sparsely punctate, basally with pair of lateral furrows containing row of scales. Female metatibia apically simple, with simple uncus and minute premucro.

##### Material examined.

Holotype (SMNK): ARC1116 (EMBL # HE615745), PAPUA NEW GUINEA, Simbu Prov., Karimui Dist., Haia, Supa, S06°40.078', E145°03.207' to S06°39.609', E145°03.012', 1220–1450 m, 02-X-2009. Paratypes (NAIC, SMNK, ZSM): PAPUA NEW GUINEA, Simbu Prov.: 3 exx, ARC1117 (EMBL # HE615746), same data as holotype; 1 ex, Haia, Supa station, S06°39.815', E145°03.169' to S06°39.609', E145°03.012', 1240–1450m, 30-IX-2009, beaten; 1 ex, ARC1108 (EMBL # HE615737), Haia, S06°43.515', E145°00.128' to S06°43.948', E144°59.856', 750–915 m, 26-IX-2009; 1 ex, Haia, Supa station, S06°40.047', E145°03.464' to S06°39.905', E145°03.880', 1075–1220 m, 01-X-2009, beaten; 1 ex, Haia, Supa station, S06°39.905', E145°03.880' to S06°39.796', E145°03.873', 1220–1320 m, 01-X-2009, beaten.

##### Distribution.

Simbu Prov. (Haia). Elevation: 915–1240 m.

##### Biology.

Collected by beating foliage in primary forest.

##### Etymology.

This species is dedicated to the people of Papua New Guinea. The epithet is based on the family name Leki, found on page 256 of the Papua New Guinea Telephone Directory of 2010 and treated in genitive plural.

##### Notes.

*Trigonopterus lekiorum* Riedel, sp. n. was coded as “*Trigonopterus* sp. 79” by Tänzler et al. (2012).

#### 47. Trigonopterus lineatus

Riedel sp. n.

urn:lsid:zoobank.org:act:5247DE6B-FC3D-4DD9-80AC-BB9628E3406B

http://species-id.net/wiki/Trigonopterus_lineatus

##### Diagnostic description.

**Figure 47.**
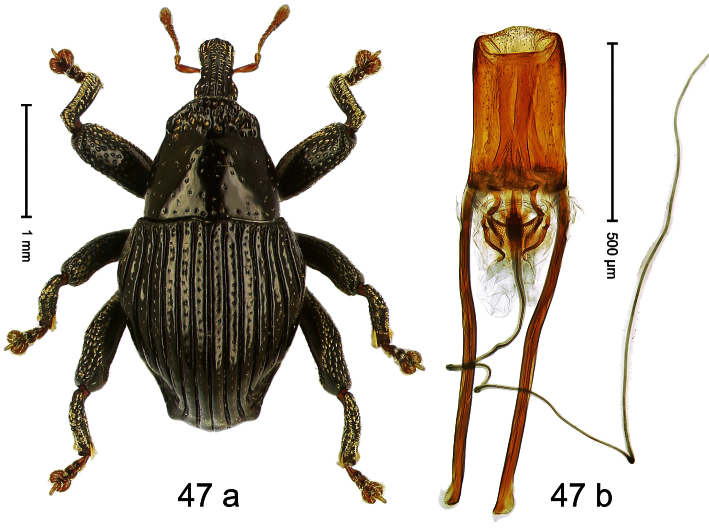
*Trigonopterus lineatus* Riedel, sp. n., holotype; (**a**) Habitus (**b**) Aedeagus.

Holotype, male (Fig. 47a). Length 3.00 mm. Color black; tarsi and antenna ferruginous. Body subrhomboid; in dorsal aspect and in profile with distinct constriction between pronotum and elytron. Rostrum in basal half with median costa bordered by furrows; in front of eyes with flat lateral extensions; subapically scabrous, with suberect scales; epistome smooth, posteriorly forming transverse, angulate ridge. Pronotum with subapical constriction, anteriorly densely coarsely punctate, with sparse lanceolate scales; disk subglabrous, with sparse punctures. Elytra with striae 1–6 deeply incised; intervals flat, with rows of small punctures and minute recumbent setae; laterally subglabrous; apex subangulate, with pair of sublateral knobs; apex extended ventrad, slightly beak-shaped. Femora edentate. Metafemur with weakly denticulate dorsoposterior edge, subapically with stridulatory patch. Aedeagus (Fig. 47b) apically simple, weakly rounded, without setae; with complex, symmetrical transfer apparatus; ductus ejaculatorius without bulbus. **Intraspecific variation**. Length 2.78–3.13 mm. Female rostrum only basally with median ridge; in apical third punctate, epistome without distinct transverse ridge.

##### Material examined.

Holotype (MZB): ARC0520 (EMBL # FN429226), WEST NEW GUINEA, Jayapura Reg., Cyclops Mts, Sentani, S02°31.912', E140°30.416', 785 m, 02-XII-2007, sifted. Paratypes (SMNK, ZSM): WEST NEW GUINEA, Jayapura Reg., Cyclops Mts, Sentani: 1 ex, ARC0519 (EMBL # FN429225), same data as holotype; 1 ex, ARC0510 (EMBL # FN429216), S02°32.031', E140°30.412', 710 m, 02-XII-2007, sifted; 1 ex, ARC0656 (EMBL # FN429303), S02°31.683', E140°30.281', 960 m, 21-XI-2007, sifted.

##### Distribution.

Jayapura Reg. (Cyclops Mts). Elevation: 710–960 m.

##### Biology.

Sifted from leaf litter in primary forest.

##### Etymology.

This epithet is based on the Latin participle *lineatus* (marked with parallel lines) and refers to the elytral sculpture.

##### Notes.

*Trigonopterus lineatus* Riedel, sp. n. was coded as “*Trigonopterus* sp. 35” by Riedel et al. (2010) and Tänzler et al. (2012), respectively “*Trigonopterus* spai” in the EMBL/GenBank/DDBJ databases.

#### 48. Trigonopterus lineellus

Riedel sp. n.

urn:lsid:zoobank.org:act:91B66959-0B3C-473D-9640-DAF4EB7A71F7

http://species-id.net/wiki/Trigonopterus_lineellus

##### Diagnostic description.

**Figure 48.**
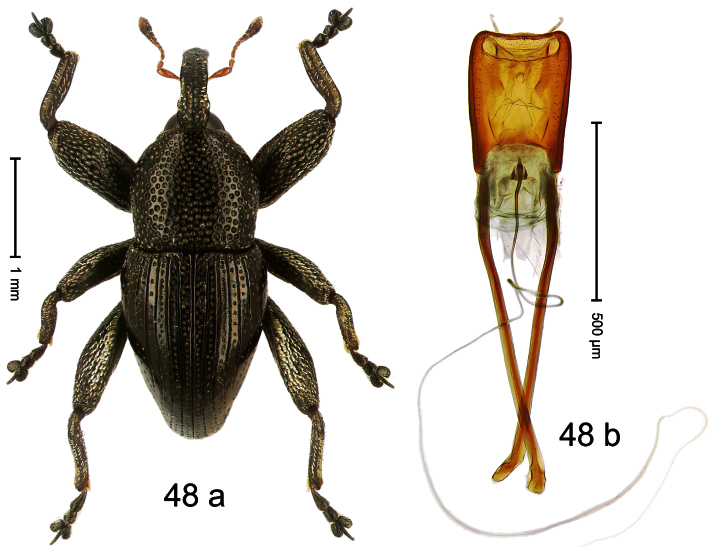
*Trigonopterus lineellus* Riedel, sp. n., holotype; (**a**) Habitus (**b**) Aedeagus.

Holotype, male (Fig. 48a). Length 3.23 mm. Color black; tibiae and antenna ferruginous. Body elongate; in dorsal aspect and in profile with distinct constriction between pronotum and elytron. Rostrum basally with distinct median and pair of submedian carinae, in apical ¼ relatively smooth; in front of eye sparsely squamose. Pronotum densely punctate with large punctures, interspaces smaller than puncture´s diameter. Elytra dorsally with striae deeply incised, intervals with 1–2 rows of punctures; laterally striae impressed as fine lines, strial punctures deep. Femora edentate. Profemur in basal third posteriorly with callus. Metafemur subapically without stridulatory patch. Abdominal ventrites 1–2 concave. Aedeagus (Fig. 48b) apically bluntly angulate, with pair of stout setae; body flattened, sides diverging to shortly before apex; ductus ejaculatorius without bulbus. **Intraspecific variation**. Length 2.83–3.63 mm. Body of females more slender. Female rostrum dorsally subglabrous, punctate, in front of eye sparsely squamose. Female abdominal ventrites 1–2 flat.

##### Material examined.

Holotype (MZB): ARC0625 (EMBL # FN429282), WEST NEW GUINEA, Jayapura Reg., Cyclops Mts, Sentani, S02°31.6', E140°30.4', 1000–1200 m, 30-XI-2007, beaten. Paratypes (ARC, SMNK, ZSM): WEST NEW GUI-NEA, Jayapura Reg., Cyclops Mts, Sentani: 1 ex, ARC0422 (EMBL # FN429133), S02°31.3', E140°30.5', 1200–1420 m, 30.XI.2007; 4 exx, ARC0465 (EMBL # FN429175), ARC0466 (EMBL # FN429176), ARC0621 (EMBL # FN429278), ARC0622 (EMBL # FN429279), S02°31.8', E140°30.5', 600–900 m, 28.XI.2007; 1 ex, ARC0623 (EMBL # FN429280), S02°31.6', E140°30.4', 900–1100 m, 28.XI.2007; 1 ex, ARC0624 (EMBL # FN429281), S02°31.6', E140°30.4', 1000–1200 m, 30.XI.2007; 1 ex (marked as ARC0043”), 950–1450 m, 03-X-1992; 2 exx, 800–1000 m, 07-VIII-1992; 3 exx, 300–1400 m, 10-VIII-1991; 1 ex, 400–800 m, 07-VIII-1992; 1 ex, 1200–1400 m, 09-VIII-1992; 1 ex, 600–1100 m, 05-X-1991; 1 ex, 850–950 m, 16-X-1996.

##### Distribution.

Jayapura Reg. (Cyclops Mts). Elevation: 800–1200 m.

##### Biology.

Collected by beating foliage in montane forests.

##### Etymology.

This epithet is based on the Latin participle *lineellus* (marked with weak parallel lines) and refers to the elytral sculpture.

##### Notes.

*Trigonopterus lineellus* Riedel, sp. n. was coded as “*Trigonopterus* sp. 11” by Riedel et al. (2010) and Tänzler et al. (2012), respectively “*Trigonopterus* spk” in the EMBL/GenBank/DDBJ databases.

#### 49. Trigonopterus maculatus

Riedel sp. n.

urn:lsid:zoobank.org:act:28208496-2A6D-4941-9D7A-F7EA09FF0105

http://species-id.net/wiki/Trigonopterus_maculatus

##### Diagnostic description.

**Figure 49.**
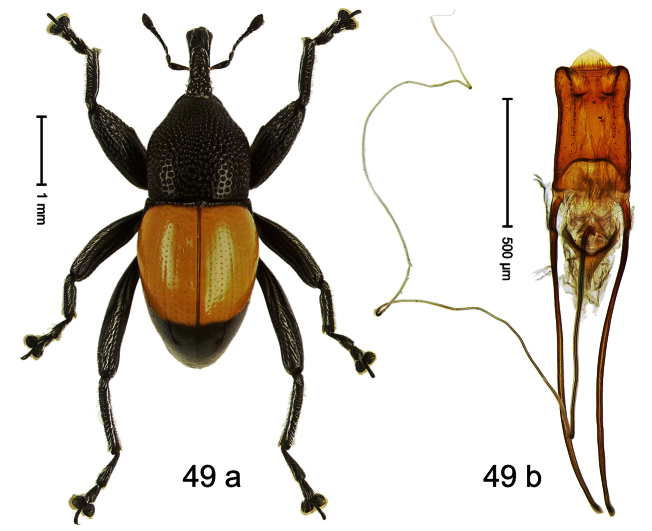
*Trigonopterus maculatus* Riedel, sp. n., holotype; (**a**) Habitus (**b**) Aedeagus.

Holotype, male (Fig. 49a). Length 3.92 mm. Color black; basal half of elytra with large orange spot. Body elongate-subovate; in dorsal aspect and in profile with distinct constriction between pronotum and elytron. Rostrum slender; dorsally punctate-rugose, with indistinct median ridge and pair of submedian ridges; apical 1/3 sparsely punctate; at base with erect white elongate scales, apically replaced by bristles. Eyes large, medially approximate. Pronotum densely punctate-reticulate; disk basally at middle with punctures containing each one long erect seta; basal contours of disk subparallel, slightly converging in straight line; laterally in front of procoxa with acute process, squamose with white scales. Elytra subglabrous; striae distinct, marked by small to minute punctures; intervals with row of minute punctures; basal margin with ridge extending behind humeri, medially somewhat swollen, bordered by indistinct row of small punctures. Femora with acute tooth at middle. Mesofemur and metafemur dorsally sparsely squamose with narrow silvery scales. Metafemur with smooth dorsoposterior edge; subapically without stridulatory patch. Thoracic and abdominal venter concave, medially densely setose. Abdominal ventrite 5 sublaterally with pair of knobs, medially densely punctate and setose. Aedeagus (Fig. 49b) with apex subangulate; ventral surface of body subapically with pair of denticles; transfer apparatus dentiform, basally supported by Y-shaped sclerite; ductus ejaculatorius subapically without bulbus. **Intraspecific variation**. Length 3.74–3.92 mm. Female rostrum dorsally subglabrous, sparsely punctate, at base with erect white scales. Female venter subglabrous, abdominal ventrites 1–2 weakly convex.

##### Material examined.

Holotype (SMNK): ARC1840 (EMBL # HE616117), PAPUA NEW GUINEA, Eastern Highlands Prov., Aiyura, S06°21.033', E145°54.597', 2169 m, 06-II-2010. Paratypes (NAIC, SMNK, ZSM): PAPUA NEW GUINEA, Eastern Highlands Prov.: 3 exx, ARC1841 (EMBL # HE616118), same data as holotype; 2 exx, ARC1842 (EMBL # HE616119), Okapa, Kimiagomo village, Hamegoya, S06°25.727', E145°35.455', S06°25.117', E145°35.225', 1891–2131 m, 18-III-2010.

##### Distribution.

Eastern Highlands Prov. (Aiyura, Okapa). Elevation: 2131–2169 m.

##### Biology.

Beaten from foliage of montane forests.

##### Etymology.

This epithet is based on a combination of the Latin noun *macula* (mark, spot) and refers to the conspicuous orange spot on the elytra.

##### Notes.

*Trigonopterus maculatus* Riedel, sp. n. was coded as “*Trigonopterus* sp. 132” by Tänzler et al. (2012).

#### 50. Trigonopterus mimicus

Riedel sp. n.

urn:lsid:zoobank.org:act:89EC9D74-0A8A-4E90-87F2-FBB8F47DA265

http://species-id.net/wiki/Trigonopterus_mimicus

##### Diagnostic description.

**Figure 50.**
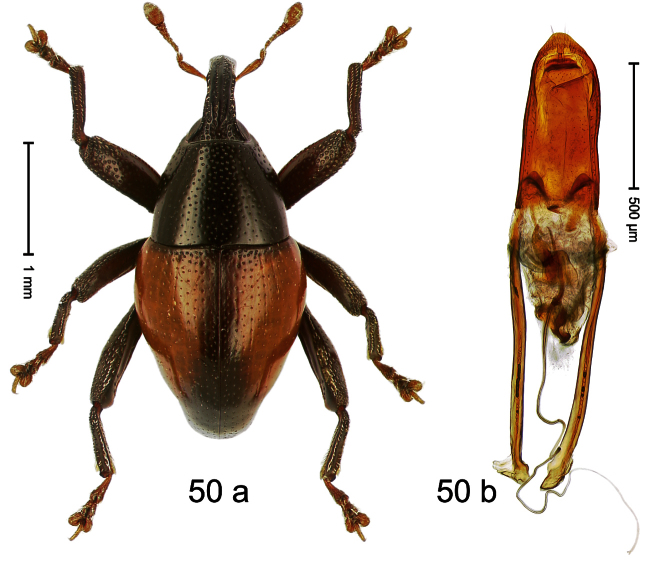
*Trigonopterus mimicus* Riedel, sp. n., holotype; (**a**) Habitus (**b**) Aedeagus.

Holotype, male (Fig. 50a). Length 2.73 mm. Color orange-ferruginous; head and pronotum black; elytra orange-ferruginous, much darker along suture and apex. Body subrhomboid; with weak constriction between pronotum and elytron; in profile evenly convex. Rostrum with distinct median and pair of submedian costae, furrows with rows of coarse punctures; in apical 1/3 weakly rugose-punctate. Pronotum moderately densely punctate with small punctures. Elytra with striae indistinct, marked by small to minute punctures, intervals with row of minute punctures. Femora edentate, with distinct anteroventral ridge. Metafemur dorsally with 1–2 rows of silvery scales; subapically with stridulatory patch. Abdominal ventrite 5 densely coarsely punctate, densely setose. Aedeagus (Fig. 50b) with apex subangulate; ostium with rectangular sclerite extending far basad; ventral surface of body in basal 1/3 with constriction and pair of angulate carinae in front of insertion of apodemes; endophallus with asymmetrical sclerites; transfer-apparatus spiniform; ductus ejaculatorius without bulbus. **Intraspecific variation**. Length 2.65–3.06 mm. Elytral color usually as in holotype, in some specimens from Tiom and Yohosim – Kiroma dark brownish. Female rostrum dorsally in apical half subglabrous, punctate. Female abdominal ventrite 5 subglabrous, sparsely punctate.

##### Material examined.

Holotype (MZB): ARC1743 (EMBL # HE616020), WEST NEW GUINEA, Jayawijaya Reg., Poga, S03°47.575', E138°33.155' to S03°47.473', E138°33.163', 2620–2715 m, 15-VII-2010. Paratypes (ARC, SMNK, ZSM): WEST NEW GUINEA, Jayawijaya Reg.: 1 ex, ARC0755 (EMBL # HE615438), Jiwika, Kurulu, S03°56.146', E138°57.710', 2245–2290 m, 26-XI-2007; 1 ex, Jiwika, trail to Wandanku, 2240–2420 m, 28-IX-1996; 2 exx (1 marked as “ARC0032”), Jiwika, 1800–2300 m, 31-V-1998; 1 ex, Jiwika, 2300 m, 1992, sifted; 2 exx, Ilugwa, Melanggama, trail to Pass-valley, 2100–2300 m, 9–10-IX-1990; 69 exx, ARC1744 (EMBL # HE616021), same data as holotype; 32 exx, ARC1808 (EMBL # HE616085), ARC1809 (EMBL # HE616086), W Wamena, road to Lake Habbema, S04°07.625', E138°49.992', 2520 m, 20-VII-2010; 2 exx, ARC1810 (EMBL # HE616087), ARC1811 (EMBL # HE616088), W Wamena, road to Lake Habbema, S04°08.256', E138°49.049', 2770 m, 20-VII-2010; 2 exx, Moss forest between Theila and L. Habbema, 2800–2950 m, 22-X-1993, beaten at night; 19 exx, Moss forest between Theila and L. Habbema, 2800–2950 m, 22-X-1993; 18 exx, Tiom, Wanuga, 2750–2900 m, 08-XII-1995; 1 ex, Poga, 2100–2500 m, 06-07-IV-1999; 7 exx, Kwiyawagi, 2750 m, 09-10-XII-1995; 3 exx, Yohosim – Kiroma, 2500–2700 m, 13-IX-1991.

##### Distribution.

Jayawijaya Reg. (Jiwika, Ilugwa, Poga, L. Habbema, Tiom). Elevation: 2290–2800 m.

##### Biology.

Beaten from foliage of montane forests.

##### Etymology.

This epithet is based on the Latin adjective *mimicus* (acting, imitating) and refers to the resemblance to other species with ferruginous elytra.

##### Notes.

*Trigonopterus mimicus* Riedel, sp. n. was coded as “*Trigonopterus* sp. 213” by Tänzler et al. (2012).

#### 51. Trigonopterus monticola

Riedel sp. n.

urn:lsid:zoobank.org:act:322A13D0-EEC1-43A9-A205-2414585A0D53

http://species-id.net/wiki/Trigonopterus_monticola

##### Diagnostic description.

**Figure 51.**
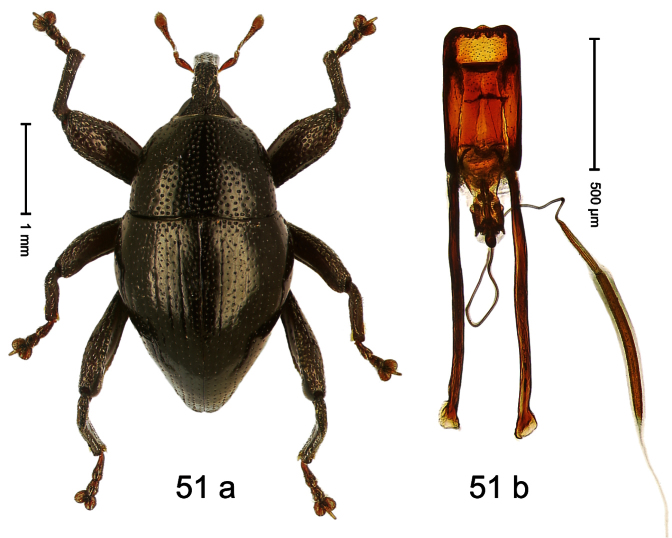
*Trigonopterus monticola* Riedel, sp. n., holotype; (**a**) Habitus (**b**) Aedeagus.

Holotype, male (Fig. 51a). Length 3.50 mm. Color black, legs and antenna deep ferruginous. Body ovate; with weak constriction between pronotum and elytron; in profile almost evenly convex, elytral base medially weakly swollen and slightly projecting. Rostrum dorsally rugose-punctate in basal half, punctate in apical half. Pronotum densely punctate except along impunctate midline. Elytra densely punctate; strial punctures slightly larger than minute punctures on intervals; striae impressed as fine lines; lateral stria behind humeri with dense row of deep punctures. Profemur and metafemur in basal half with anteroventral ridge terminating as tooth; tooth of profemur small. Metafemur with denticulate dorsoposterior edge; with crenulate anteroventral ridge, terminating in apical 1/3 as acute tooth; subapically with stridulatory patch. Abdominal ventrite 5 with shallow impression, densely punctate, sublaterally setose. Aedeagus (Fig. 51b) with ostium somewhat retracted; with distinct, symmetrical transfer-apparatus; ductus ejaculatorius with bulbus. **Intraspecific variation**. Length 2.63–3.53 mm. Female rostrum basally punctate, towards apex dorsally subglabrous, with rows of small punctures. Female abdominal ventrite 5 flat, sparsely punctate.

##### Material examined.

Holotype (MZB): ARC0420 (EMBL # FN429131), WEST NEW GUINEA, Jayapura Reg., Cyclops Mts, Sentani, S02°31.7', E140°30.3', 900–1150 m, 21.XI.2007, beaten, marked “stridul. 3”. Paratypes (ARC, SMNK, ZSM): WEST NEW GUINEA, Jayapura Reg., Cyclops Mts, Sentani: 23 exx, ARC0431 (EMBL # FN429142), S02°31.2', E140°30.5', 1420–1520 m, 30-XI-2007; 2 exx, ARC0450 (EMBL # FN429161), ARC0454 (EMBL # FN429165), S02°31.7', E140°30.3', 850–1000 m, 30.XI.2007; 8 exx, S02°31.6', E140°30.4', 1000–1200 m, 30-XI-2007, beaten; 38 exx, ARC0484 (EMBL # FN429191), ARC0487 (EMBL # FN429194), S02°31.3', E140°30.5', 1200–1420 m, 30-XI-2007; 1 ex, S02°31.425', E140°30.474', 1265 m, 30-XI-2007, sifted; 1 ex, ARC0686 (EMBL # FN429331), S02°31.182', E140°30.542', 1510 m, 30-XI-2007; 17 exx [1 marked “ARC 042”], 950–1450 m, 03-X-1992; 4 exx, 1100–1600 m, 05-X-1991; 17 exx, 1200–1400 m, 09-VIII-1992; 17 exx, 1100–1600 m, 05-X-1991; 13 exx, 1400 m, 10-VIII-1991; 4 exx, 950–1450 m, 03-X-1992.

##### Distribution.

Jayapura Reg. (Cyclops Mts). Elevation: 1000–1510 m.

##### Biology.

Collected by beating foliage in lowland forests.

##### Etymology.

This epithet is based on the Latin noun *monticola* (mountain dweller) and refers to the species´ restriction to the upper elevations of the Cyclops Mountains.

**Notes**. *Trigonopterus monticola* Riedel, sp. n. was coded as “*Trigonopterus* sp. 3” by Riedel et al. (2010) and Tänzler et al. (2012), respectively “*Trigonopterus* spc” in the EMBL/GenBank/DDBJ databases.

#### 52. Trigonopterus montivagus

Riedel sp. n.

urn:lsid:zoobank.org:act:1A9FE62A-E6EF-43B3-8F2C-43A56E5F7771

http://species-id.net/wiki/Trigonopterus_montivagus

##### Diagnostic description.

**Figure 52.**
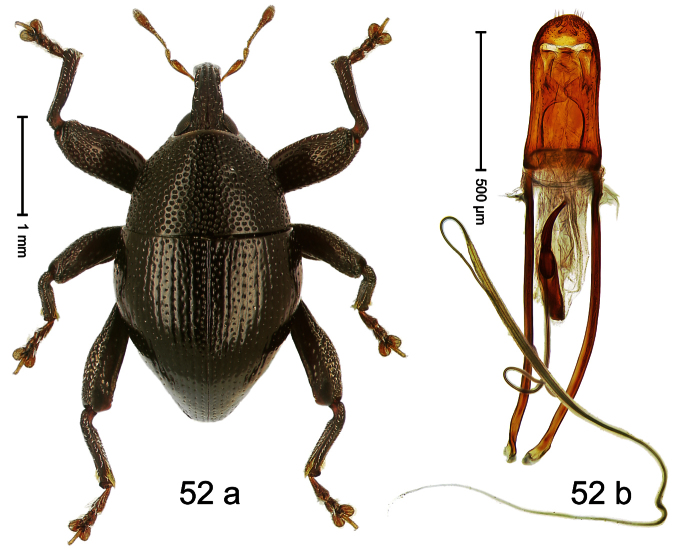
*Trigonopterus montivagus* Riedel, sp. n., holotype; (**a**) Habitus (**b**) Aedeagus.

Holotype, male (Fig. 52a). Length 2.94 mm. Color black; antennae light ferruginous, legs deep ferruginous. Body subovate; with weak constriction between pronotum and elytron; in profile evenly convex. Rostrum in basal half with distinct median ridge and pair of submedian ridges, furrows with sparse rows of yellowish scales; apically weakly punctate, sparsely setose. Pronotum coarsely punctate-reticulate. Elytra with dense, somewhat confused punctation; striae distinct, impressed as very fine lines, strial punctures small; intervals with slightly smaller punctures; laterally behind humeri simple. Femora edentate. Metafemur with denticulate dorsoposterior edge, with sparse row of suberect silvery scales; subapically with stridulatory patch. Metatibia apically with uncus and minute premucro. Abdominal ventrite 5 with shallow impression, densely punctate, with dense suberect scales. Aedeagus (Fig. 52b) with sides in apical 1/3 converging, weakly rounded, sparsely setose; transfer-apparatus spiniform; ductus ejaculatorius with bulbus. **Intraspecific variation**. Length 2.53–3.20 mm. Female rostrum dorsally subglabrous, sparsely punctate, in basal ¼ with longitudinal ridges and furrows. Elytral sculpture differing markedly among populations: striae weakly or hardly impressed in specimens from Eastern Highlands; distinctly impressed in specimens from Pindiu; deeply incised forming well-defined furrows in specimens from Mindik.

##### Material examined.

Holotype (SMNK): ARC1873 (EMBL # HE616150), PAPUA NEW GUINEA, Eastern Highlands Prov., Aiyura, S06°21.033', E145°54.597', 2169 m, 06-II-2010. Paratypes (NAIC, SMNK, ZSM): PAPUA NEW GUINEA: 2 exx, ARC1874 (EMBL # HE616151), ARC1875 (EMBL # HE616152), same data as holotype; 4 exx, Okapa, Afiyaleto village, S06°25.593', E145°34.862' to S06°25.212', E145°35.498', 1911 m, 18-III-2010, beaten; 1 ex, Okapa, Isimomo, S06°25.003', E145°34.527', 2131 m, 22-XII-2010; 2 exx, Okapa, Konafi to Isimomo, S06°25.593', E145°34.862', S06°25.003', E145°34.527', 1911–2131 m, 18-III-2010, 3 exx, Okapa, Kimiagomo village, Hamegoya, S06°25.727', E145°35.455', S06°25.117', E145°35.225', 1891–2131 m, 18-III-2010; 3 exx, ARC1054 (EMBL # HE615685), ARC1055 (EMBL # HE615686), ARC1056 (EMBL # HE615687), Eastern Highlands Prov., Goroka, Mt. Gahavisuka, S06°00.864', E145°24.779', 2150–2250 m, 24-X-2009; 6 exx, ARC1191 (EMBL # HE615819), ARC1192 (EMBL # HE615820), ARC1193 (EMBL # HE615821), Morobe Prov., Huon peninsula, Mindik, S06°27.380', E147°25.099' to S06°27.267', E147°25.049', 1500–1650 m, 09-X-2009; 2 exx, Mindik, 1200–1500 m, 26-IV-1994; 11 exx, Mindik, 1670–1710 m, S06°27.221', E147°24.185' to S06°27.196', E147°24.276', 10-X-2009, beaten; 22 exx, ARC1243 (EMBL # HE615871), ARC1244 (EMBL # HE615872), ARC1245 (EMBL # HE615873), Morobe Prov., Huon peninsula, mountain SW Pindiu, S06°27.437', E147°30.512' to S06°27.435', E147°30.310', 1170–1225 m, 14-X-2009; 1 ex, mountain SW Pindiu, 1225–1340 m, S06°27.435', E147°30.310' to S06°27.307', E147°30.168', 14-X-2009, beaten; 8 exx, Boana, Saruwaged-Mts, 1000–1500 m, 21–22-X-1992; 4 exx, E Pindiu, Kobau, 1250–1400 m, 24-IV-1998; 1 ex, W Pindiu, 1000–1400 m, 1200–1500 m, 26-IV-1998.

##### Distribution.

Eastern Highlands Prov. (Aiyura, Goroka, Okapa); Morobe Prov. (Mindik, Pindiu). Elevation: 1340–2169 m.

##### Biology.

Beaten from foliage of montane forests.

##### Etymology.

This epithet is based on a combination of the Latin noun *mons* (mountain) and the participle *vagus* (wandering) and refers to the relatively wide distribution in the highlands of Papua New Guinea.

##### Notes.

*Trigonopterus montivagus* Riedel, sp. n. was coded as “*Trigonopterus* sp. 205” by Tänzler et al. (2012).

#### 53. Trigonopterus moreaorum

Riedel sp. n.

urn:lsid:zoobank.org:act:B28315B8-C173-4FEC-8BDE-5AB9733790D7

http://species-id.net/wiki/Trigonopterus_moreaorum

##### Diagnostic description.

**Figure 53.**
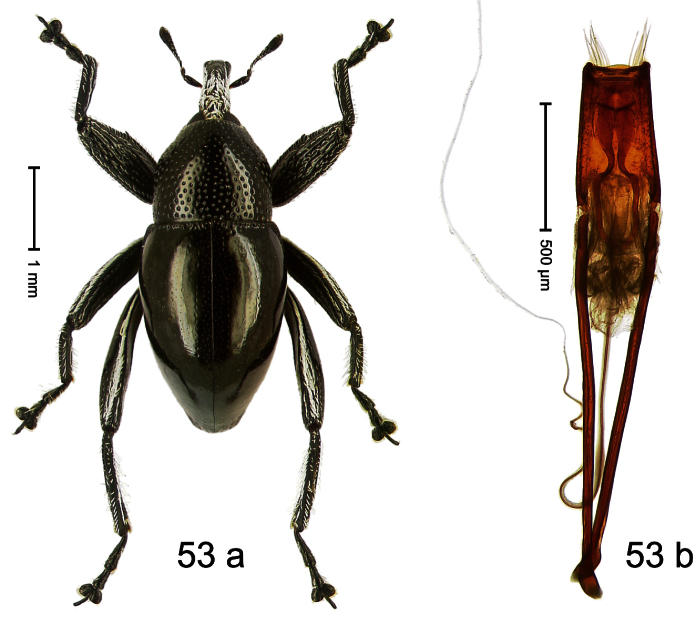
*Trigonopterus moreaorum* Riedel, sp. n., holotype; (**a**) Habitus (**b**) Aedeagus.

Holotype, male (Fig. 53a). Length 3.76 mm. Color black. Body elongate-subovate; in dorsal aspect and in profile with distinct constriction between pronotum and elytron. Rostrum slender; dorsally punctate-rugose, with distinct median ridge and pair of submedian ridges; apical 1/3 sparsely punctate; at base with erect white elongate scales, apically replaced by bristles. Eyes large, medially approximate. Pronotum densely punctate, each puncture containing one short seta; basally with indistinct lateral edge, in basal half contours of disk subparallel; sides anteriorly with few scattered white scales. Elytra subglabrous; irregularly punctate with minute punctures; basal margin with distinct ridge extending behind humeri, bordered by row of coarse punctures. Femora with acute tooth at middle. Mesofemur and metafemur dorsally densely squamose with narrow white scales. Metafemur with smooth dorsoposterior edge; subapically without stridulatory patch. Meso- and metatibia with rows of long erect setae. Venter with sparse pubescence of long suberect setae. Metaventrite and abdominal ventrites 1–2 medially forming common concavity. Aedeagus (Fig. 53b) with apex subangulate, with long setae; body containing somewhat X-shaped sclerite subequal to length of body; transfer apparatus dentiform, ductus ejaculatorius subapically without bulbus. **Intraspecific variation**. Length 3.31–3.76 mm. Female rostrum in apical 2/3 dorsally flattened, subglabrous, sparsely punctate; at base with erect, white, elongate scales. Female venter subglabrous, with scattered long suberect scales; female abdominal ventrites 1–2 flat.

##### Material examined.

Holotype (SMNK): ARC1862 (EMBL # HE616139), PAPUA NEW GUINEA, Eastern Highlands Prov., Okapa, Kimiagomo village, Verefare, S06°24.760', E145°35.575', 1940 m, 18-III-2010. Paratypes (NAIC, SMNK): PAPUA NEW GUINEA, Eastern Highlands Prov.: 2 exx, ARC1863 (EMBL # HE616140), ARC1864 (EMBL # HE616141), same data as holotype.

##### Distribution.

Eastern Highlands Prov. (Okapa). Elevation: 1940 m.

##### Biology.

Beaten from foliage of montane forests.

##### Etymology.

This species is dedicated to the people of Papua New Guinea. The epithet is based on the family name Morea, found on page 275 of the Papua New Guinea Telephone Directory of 2010 and treated in genitive plural.

##### Notes.

*Trigonopterus moreaorum* Riedel, sp. n. was coded as “*Trigonopterus* sp. 145” by Tänzler et al. (2012).

#### 54. Trigonopterus myops

Riedel sp. n.

urn:lsid:zoobank.org:act:B3936427-7F5C-4541-844E-E3DECD929C3D

http://species-id.net/wiki/Trigonopterus_myops

##### Diagnostic description.

**Figure 54.**
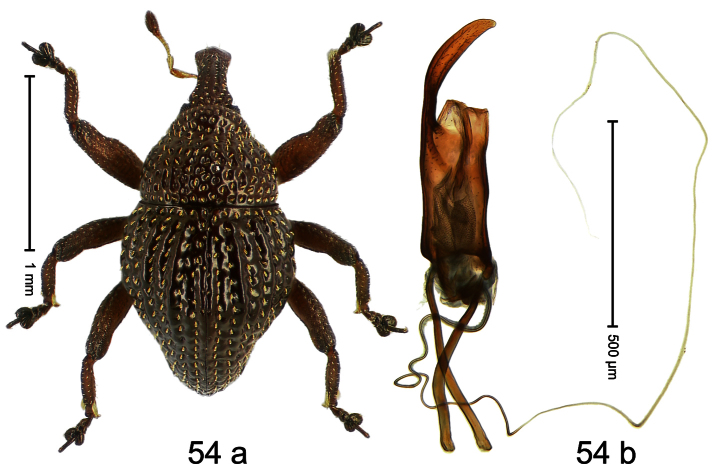
*Trigonopterus myops* Riedel, sp. n., holotype; (**a**) Habitus (**b**) Aedeagus.

Holotype, male (Fig. 54a). Length 1.66 mm. Color dark ferruginous, elytra almost black; antenna light ferruginous. Body subovate, in dorsal aspect and in profile with distinct constriction between pronotum and elytron. Rostrum with indistinct, median ridge, bordered by row of coarse punctures, sparsely setose; epistome simple. Eyes small, anteriorly angularly projecting. Pronotum with weak subapical constriction; disk densely, coarsely punctate; each puncture with short, yellowish scale. Elytra with striae deeply impressed; each puncture with suberect, yellowish scale; intervals subglabrous, uneven, intervals 3, 5 and 7 distinctly costate; apex extended ventrad, slightly beak-shaped. Meso- and metafemur ventrally dentate. Metafemur subapically without stridulatory patch. Aedeagus (Fig. 54b) with apex asymmetrical, right side forming long curved extension; basal orifice ventrally with rim; transfer apparatus spiniform; ductus ejaculatorius without bulbus. **Intraspecific variation**. Length 1.35–1.76 mm. Female rostrum dorsally subglabrous, sparsely punctate, in basal third coarsely punctate. Abdominal ventrite 3 of females with flattened subtriangular process projecting over ventrite 4 with pair of submedian spines. Female abdominal ventrites 4–5 weakly sclerotized.

##### Material examined.

Holotype (MZB): ARC0777 (EMBL # HE615460), WEST NEW GUINEA, Manokwari Reg., Manokwari, Arfak Mts, S01°03.723', E133°54.145', 1385 m, 08-XII-2007. Paratypes (SMNK, ZSM): WEST NEW GUINEA, Manokwari Reg., Manokwari, Arfak Mts: 14 exx, ARC0778 (EMBL # HE615461), same data as holotype; 14 exx, Arfak Mts, S01°04.087', E133°54.268', 1520 m, 08-XII-2007; 51 exx, ARC0799 (EMBL # HE615482), Mokwam, Siyoubrig, S01°06.668', E133°54.594', 1535 m, 08-XII-2007; 5 exx, Mokwam, Siyoubrig, S01°06.107', E133°54.888', 1530 m, 10-XII-2007; 21 exx, Mokwam, Siyoubrig, S01°06.086', E133°55.027', 1500 m, 10-XII-2007, sifted.

##### Distribution.

Manokwari Reg. (Arfak Mts). Elevation: 1385–1535 m.

##### Biology.

Sifted from leaf litter in montane forest.

##### Etymology.

This epithet is based on the Greek *myops* (short-sighted) and refers to its peculiar eyes of a few ommatidia.

##### Notes.

*Trigonopterus myops* Riedel, sp. n. was coded as “*Trigonopterus* sp. 232” by Tänzler et al. (2012).

#### 55. Trigonopterus nangiorum

Riedel sp. n.

urn:lsid:zoobank.org:act:E10159AB-092C-4CDF-A3F8-D5FC52AB99D7

http://species-id.net/wiki/Trigonopterus_nangiorum

##### Diagnostic description.

**Figure 55.**
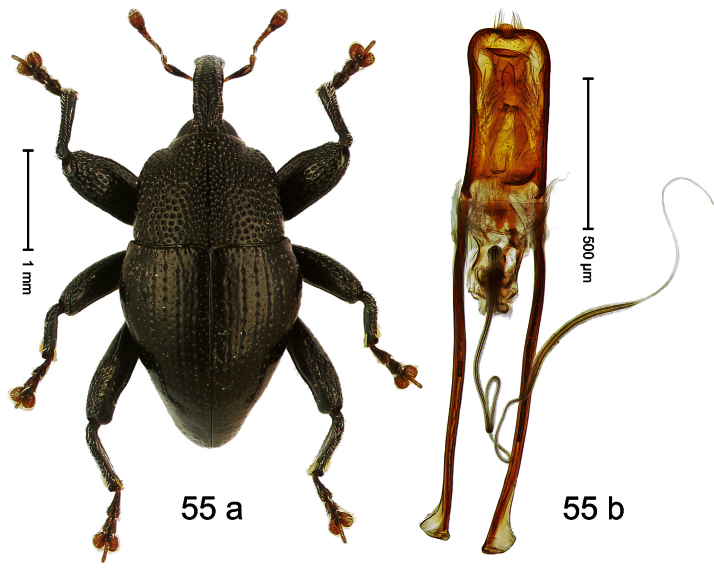
*Trigonopterus nangiorum* Riedel, sp. n., holotype; (**a**) Habitus (**b**) Aedeagus.

Holotype, male (Fig. 55a). Length 3.17 mm. Color black; antenna and tarsi ferruginous. Body subovate; with distinct constriction between pronotum and elytron; in profile evenly convex. Rostrum dorsally with median and pair of submedian ridges, furrows with sparse rows of setae; subapically sparsely punctate. Pronotum densely punctate; interspaces dull, microgranulate; with indistinct lateral edges converging in straight line to shortly before apex. Elytra densely punctate; striae distinct, marked by fine lines and rows of small punctures; intervals with row of minute punctures; laterally behind humeri with dense row of deep punctures of stria 9. Femora edentate. Mesofemur and metafemur dorsally with sparse yellowish lanceolate scales. Metafemur with denticulate dorsoposterior edge; subapically with stridulatory patch. Metatibia apically with uncus and distinct premucro. Procoxa ventrally with dense patch of long erect setae. Posteroventral rim of mesoventral receptacle densely setose with long erect setae. Abdominal ventrite 5 with distinct impression, densely punctate. Aedeagus (Fig. 55b) with sides subparallel, apically rounded with median nipple-shaped extension, sparsely setose; body dorsally with patches of long setae; transfer-apparatus flagelliform; ductus ejaculatorius with bulbus. **Intraspecific variation**. Length 2.69–3.17 mm. Female rostrum in apical half dorsally with sculpture shallower than in males. Male pronotum anteriorly subangularly projecting, in females rather rounded. Female mesoventral receptacle with posteroventral rim subglabrous. Female abdominal ventrite 5 flat, subglabrous, sparsely punctate.

##### Material examined.

Holotype (SMNK): ARC1865 (EMBL # HE616142), PAPUA NEW GUINEA, Eastern Highlands Prov., Aiyura, S06°21.033', E145°54.597', 2169 m, 06-II-2010. Paratypes (NAIC, SMNK, ZSM): PAPUA NEW GUINEA, Eastern Highlands Prov.: 10 exx, ARC1866 (EMBL # HE616143), ARC1867 (EMBL # HE616144), same data as holotype; 2 exx, ARC0351 (EMBL # HE615164), ARC0352 (EMBL # HE615165), Aiyura, S06°21.131', E145°54.398', 1670 m, 05-IV-2006.

##### Distribution.

Eastern Highlands Prov. (Aiyura). Elevation: 1670–2169 m.

##### Biology.

Beaten from foliage of montane forests.

##### Etymology.

This species is dedicated to the people of Papua New Guinea. The epithet is based on the family name Nagi, found on page 280 of the Papua New Guinea Telephone Directory of 2010 and treated in genitive plural.

##### Notes.

*Trigonopterus nangiorum* Riedel, sp. n. was coded as “*Trigonopterus* sp. 175” by Tänzler et al. (2012).

#### 56. Trigonopterus nothofagorum

Riedel sp. n.

urn:lsid:zoobank.org:act:19817145-716A-41D7-A77E-296475D433A6

http://species-id.net/wiki/Trigonopterus_nothofagorum

##### Diagnostic description.

**Figure 56.**
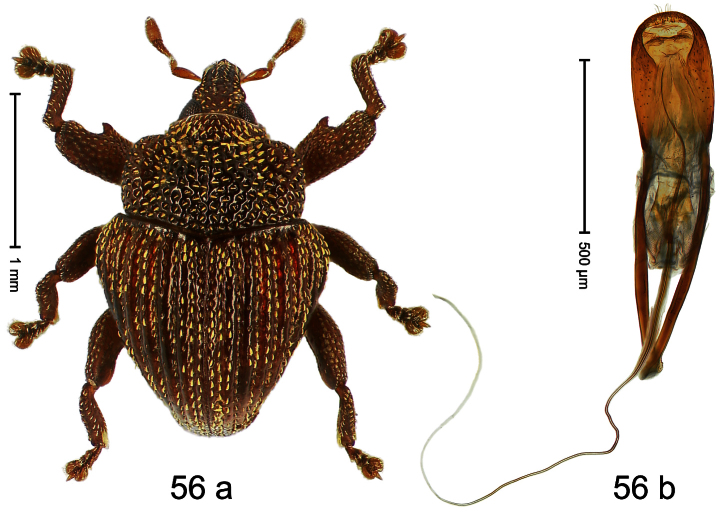
*Trigonopterus nothofagorum* Riedel, sp. n., holotype; (**a**) Habitus (**b**) Aedeagus.

Holotype, male (Fig. 56a). Length 2.10 mm. Color ferruginous, center of pronotum black. Body subrhomboid; in dorsal aspect with marked constriction between pronotum and elytron; in profile with shallow constriction. Rostrum with indistinct median wrinkle and pair of submedian wrinkles, coarsely punctate; epistome forming angulate ridge, medially with weak denticle. Pronotum with subapical constriction; with indistinct lateral flanges; coarsely punctate-reticulate; punctures each with one ochre scale. Elytra heart-shaped, humeri prominent; striae deeply incised; intervals costate; sparsely squamose with ochre scales. Femora ventrally markedly dentate. Metafemur subapically with stridulatory patch. Onychium subequal to tarsomere 3, fusiform. Aedeagus (Fig. 56b) with apex rounded; transfer apparatus flagelliform; ductus ejaculatorius without bulbus. **Intraspecific variation**. Length 1.78–2.17 mm. Color ranging from ferruginous with black spots to largely black with few ferruginous spots. Body of females subovate. Female rostrum with rows of coarse punctures; epistome simple. Female elytra with humeri simple, not prominent as in males. Femoral tooth smaller or larger.

##### Material examined.

Holotype (MZB): ARC0786 (EMBL # HE615469), WEST NEW GUINEA, Manokwari, Arfak Mts, S01°04.087', E133°54.268', 1520 m, 08-XII-2007, sifted. Paratypes (SMNK, ZSM): 25 exx, ARC0787 (EMBL # HE615470), ARC0788 (EMBL # HE615471), ARC0789 (EMBL # HE615472), same data as holotype; 10 exx, Mokwam, Siyoubrig, S01°06.107', E133°54.888', 1530 m, 10-XII-2007, sifted.

##### Distribution.

Manokwari Reg. (Arfak Mts). Elevation: 1520–1530 m.

##### Biology.

Sifted from leaf litter in montane forest dominated by *Nothofagus*.

##### Etymology.

This epithet is based on the plant genus *Nothofagus*.

##### Notes.

*Trigonopterus nothofagorum* Riedel, sp. n. was coded as “*Trigonopterus* sp. 235” by Tänzler et al. (2012).

#### 57. Trigonopterus ovatus

Riedel sp. n.

urn:lsid:zoobank.org:act:AF215921-4907-479E-BA54-D519D9124B48

http://species-id.net/wiki/Trigonopterus_ovatus

##### Diagnostic description.

**Figure 57.**
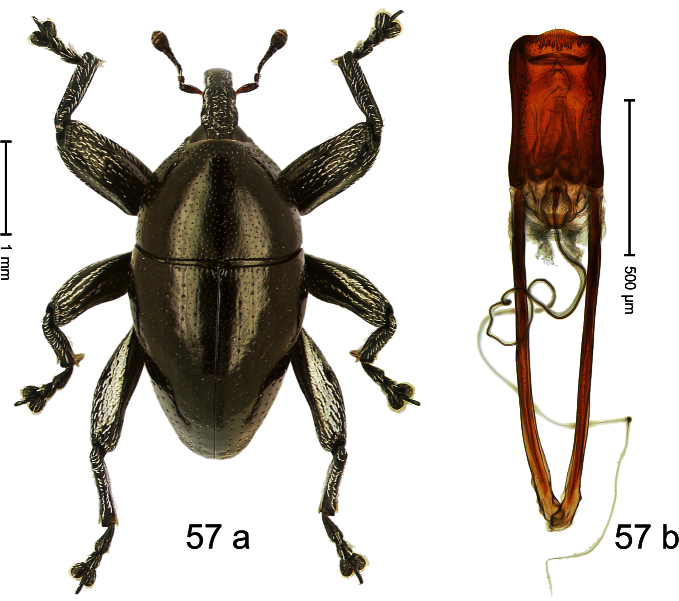
*Trigonopterus ovatus* Riedel, sp. n., holotype; (**a**) Habitus (**b**) Aedeagus.

Holotype, male (Fig. 57a). Length 3.56 mm. Color black; antennal scape and funicle ferruginous. Body ovate; with weak constriction between pronotum and elytron; in profile evenly convex. Rostrum dorsally densely punctate, without ridges or furrows. Pronotum densely punctate; dorsally punctures minute, laterally larger, anterolaterally with scattered white scales. Elytra punctate with minute punctures, especially on intervals; strial punctures slightly larger; striae impressed as fine lines; lateral stria behind humeri simple, not deepened. Femora edentate. Mesofemur and metafemur dorsally densely squamose with white scales. Metafemur with smooth dorsoposterior edge; subapically without stridulatory patch. Metatibia apically with uncus, without premucro. Aedeagus (Fig. 57b) symmetrical; apex subangulate, subglabrous; transfer-apparatus simple; ductus ejaculatorius without bulbus. **Intraspecific variation**. Length 2.97–3.94 mm. Female rostrum in apical half slightly narrower than in male, dorsally with punctures slightly sparser and smaller.

##### Material examined.

Holotype (SMNK): ARC1127 (EMBL # HE615756), PAPUA NEW GUINEA, Simbu Prov., Karimui Dist., Haia, Supa, S06°40.078', E145°03.207' to S06°39.609', E145°03.012', 1220–1450 m, 02-X-2009. Paratypes (NAIC, SMNK, ZSM): Simbu Prov., Karimui Dist., Haia: 143 exx, ARC1154 (EMBL # HE615782), ARC1155 (EMBL # HE615783), ARC1156 (EMBL # HE615784), S06°41.216', E145°00.945' to S06°40.976', E145°00.979', 970–1135 m, 04-X-2009; 2 exx, S06°43.515', E145°00.128' to S06°43.948', E144°59.856', 750–915 m, 26-IX-2009; 15 exx, Haia, S06°41.259', E145°00.822' to S06°41.102', E145°00.979', 900–1005 m, 27-IX-2009; 27 exx, Simbu Prov., Karimui Dist., Haia, S06°41.102', E145°00.979', 1005–1020 m, 27-IX-2009, beaten, “Mimikry-sample”; 52 exx, Haia, S06°41.102', E145°00.979' to S06°40.976', E145°00.979', 1020–1135 m, 27-IX-2009, beaten; 1 ex, S06°41.553', E145°00.355' to S06°41.624', E145°00.728', 800–960 m, 25-IX-2009; 7 exx, ARC1128 (EMBL # HE615757), same data as holotype; 3 exx, Haia, Supa station, S06°39.815', E145°03.169' to S06°39.609', E145°03.012', 1240–1450 m, 30-IX-2009, beaten; 12 exx, Haia, Supa station, S06°40.047', E145°03.464' to S06°39.905', E145°03.880', 1075–1220 m, 01-X-2009, beaten; 17 exx, Haia, Supa station, S06°39.905', E145°03.880' to S06°39.796', E145°03.873', 1220–1320 m, 01-X-2009, beaten.

##### Distribution.

Simbu Prov. (Haia). Elevation: 915–1240 m.

##### Biology.

Collected by beating foliage in primary forests.

##### Etymology.

This epithet is based on the Latin adjective *ovatus* (egg-shaped) and refers to the species´ body form.

##### Notes.

*Trigonopterus ovatus* Riedel, sp. n. was coded as “*Trigonopterus* sp. 147” by Tänzler et al. (2012).

#### 58. Trigonopterus oviformis

Riedel sp. n.

urn:lsid:zoobank.org:act:E10C6E76-3AAE-40A5-9B83-322703ED5182

http://species-id.net/wiki/Trigonopterus_oviformis

##### Diagnostic description.

**Figure 58.**
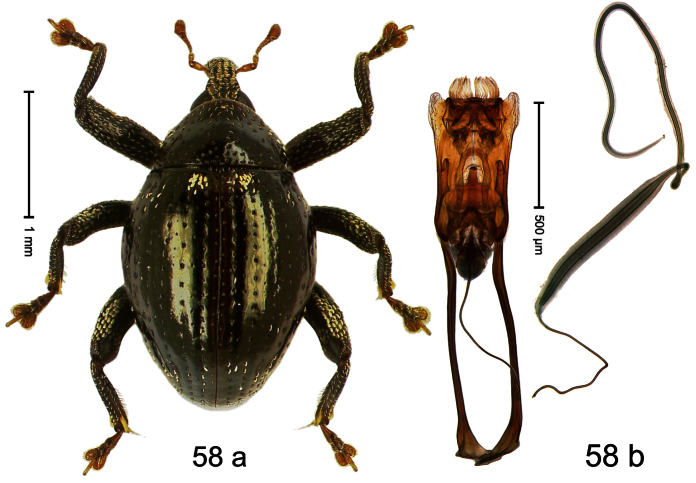
*Trigonopterus oviformis* Riedel, sp. n., holotype; (**a**) Habitus (**b**) Aedeagus.

Holotype, male (Fig. 58a). Length 2.45 mm. Color black with bronze lustre, antenna and tarsi ferruginous. Body subovate; in dorsal aspect and in profile with weak constriction between pronotum and elytron. Rostrum dorsally with median and pair of submedian ridges; sparsely squamose with slender subrecumbent scales. Pronotum relatively small; disk sparsely punctate; laterally with sparse, almond-shaped, cream-colored scales. Elytra with striae distinct; intervals flat, subglabrous; interval 2 basally with patch of almond-shaped, cream-colored scales. Femora ventrally with minute denticle. Meso- and metafemur especially dorsally squamose with cream-colored scales; metafemur subapically with stridulatory patch. Meso- and metatibia in apical half with regular row of setae, near uncus with small brush of long setae. Aedeagus (Fig. 58b) apically subtruncate, with dense fringe of setae; subapically sides with pair of membranous protrusions; orifice and body with complex sclerites; transfer apparatus symmetrical; ductus ejaculatorius with bulbus. **Intraspecific variation**. Length 2.16–2.45 mm. Female rostrum dorsally punctate and sparsely setose, in basal 1/3 coarsely punctate-rugose.

##### Material examined.

Holotype (SMNK): ARC1086 (EMBL # HE615716), PAPUA NEW GUINEA, Simbu Prov., Karimui Dist., Haia, S06°41.624', E145°00.728', 960 m, 25-IX-2009. Paratypes (NAIC): 1 ex, ARC1092 (EMBL # HE615722), PAPUA NEW GUINEA, Simbu Prov., Karimui Dist., Haia S06°43.948', E144°59.856', 915 m, 26-IX-2009.

##### Distribution.

Simbu Prov. (Haia). Elevation: 915–960 m.

##### Biology.

Sifted from leaf litter in primary forest.

##### Etymology.

This epithet is based on a combination of the Latin noun *ovum* (egg) and the suffix *-formis* (-shaped) and refers to the habitus of this species.

##### Notes.

*Trigonopterus oviformis* Riedel, sp. n. was coded as “*Trigonopterus* sp. 254” by Tänzler et al. (2012).

#### 59. Trigonopterus parumsquamosus

Riedel sp. n.

urn:lsid:zoobank.org:act:78AA5E83-38E8-4898-94A2-4C938342C306

http://species-id.net/wiki/Trigonopterus_parumsquamosus

##### Diagnostic description.

**Figure 59.**
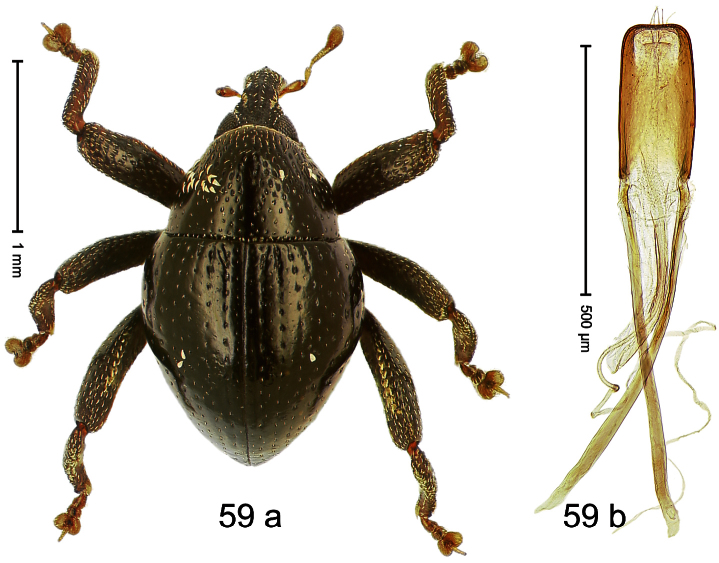
*Trigonopterus parumsquamosus* Riedel, sp. n., holotype; (**a**) Habitus (**b**) Aedeagus.

Holotype, male (Fig. 59a). Length 2.03 mm. Color black; legs ferruginous; antenna light ferruginous. Body subovate, in dorsal aspect with marked constriction between pronotum and elytron; in profile almost evenly convex. Rostrum dorsally scabrous, basally with indistinct median ridge and pair of sublateral ridges; epistome forming angulate ridge and small median denticle. Pronotum densely punctate with ovate punctures, medially subglabrous; at middle with pair of transverse squamose patches; scales cream-colored, subtriangular. Elytra with striae marked by small punctures; interval 4 at middle with cluster of few cream-colored recumbent scales. Meso- and metafemur with anteroventral ridge simple. Meso- and metatibia in basal half widened, subapically narrowed. Metafemur with denticulate dorsoposterior edge, subapically without stridulatory patch. Aedeagus (Fig. 59b) with sides of body subparallel, apex rounded; transfer apparatus flagelliform; ductus ejaculatorius without bulbus. **Intraspecific variation**. Length 1.60–2.03 mm. Female rostrum in apical half medially subglabrous, sublaterally punctate, without ridges; epistome simple. Cream-colored scales of pronotum and elytron in some specimens largely abraded.

##### Material examined.

Holotype (MZB): ARC0527 (EMBL # FN429233), WEST NEW GUINEA, Jayapura Reg., Cyclops Mts, Sentani, S02°31.912', E140°30.416', 785 m, 02-XII-2007, sifted. Paratypes (ARC, SMNK, ZSM): WEST NEW GUINEA, Jayapura Reg., Cyclops Mts, Sentani: 6 exx, ARC0528 (EMBL # FN429234), ARC0529 (EMBL # FN429235), same data as holotype; 2 exx, ARC0654 (EMBL # FN FN429301), ARC0655 (EMBL # FN429302), S02°31.594', E140°30.407', 1065 m, 21-XI-2007, sifted; 1 ex, ARC0657 (EMBL # FN429304), S02°31.776', E140°30.215', 945 m, 21-XI-2007, sifted; 2 exx, S02°31.683', E140°30.281', 960 m, 21-XI-2007, sifted; 3 exx (1 marked ARC0412), 700 m, 22-XII-2004, sifted; 3 exx, 1000 m, 23-XII-2004, sifted.

##### Distribution.

Jayapura Reg. (Cyclops Mts). Elevation: 700–1065 m.

##### Biology.

Sifted from leaf litter in primary forest.

##### Etymology.

This epithet is based on a combination of the Latin words *parum* (sparse) and *squamosus* (scaled) and refers to the few scattered scales.

##### Notes.

*Trigonopterus parumsquamosus* Riedel, sp. n. was coded as “*Trigonopterus* sp. 43” by Riedel et al. (2010) and Tänzler et al. (2012), respectively “*Trigonopterus* spaq” in the EMBL/GenBank/DDBJ databases.

#### 60. Trigonopterus parvulus

Riedel sp. n.

urn:lsid:zoobank.org:act:1F696E2A-E177-460B-B948-9CB293658E17

http://species-id.net/wiki/Trigonopterus_parvulus

##### Diagnostic description.

**Figure 60.**
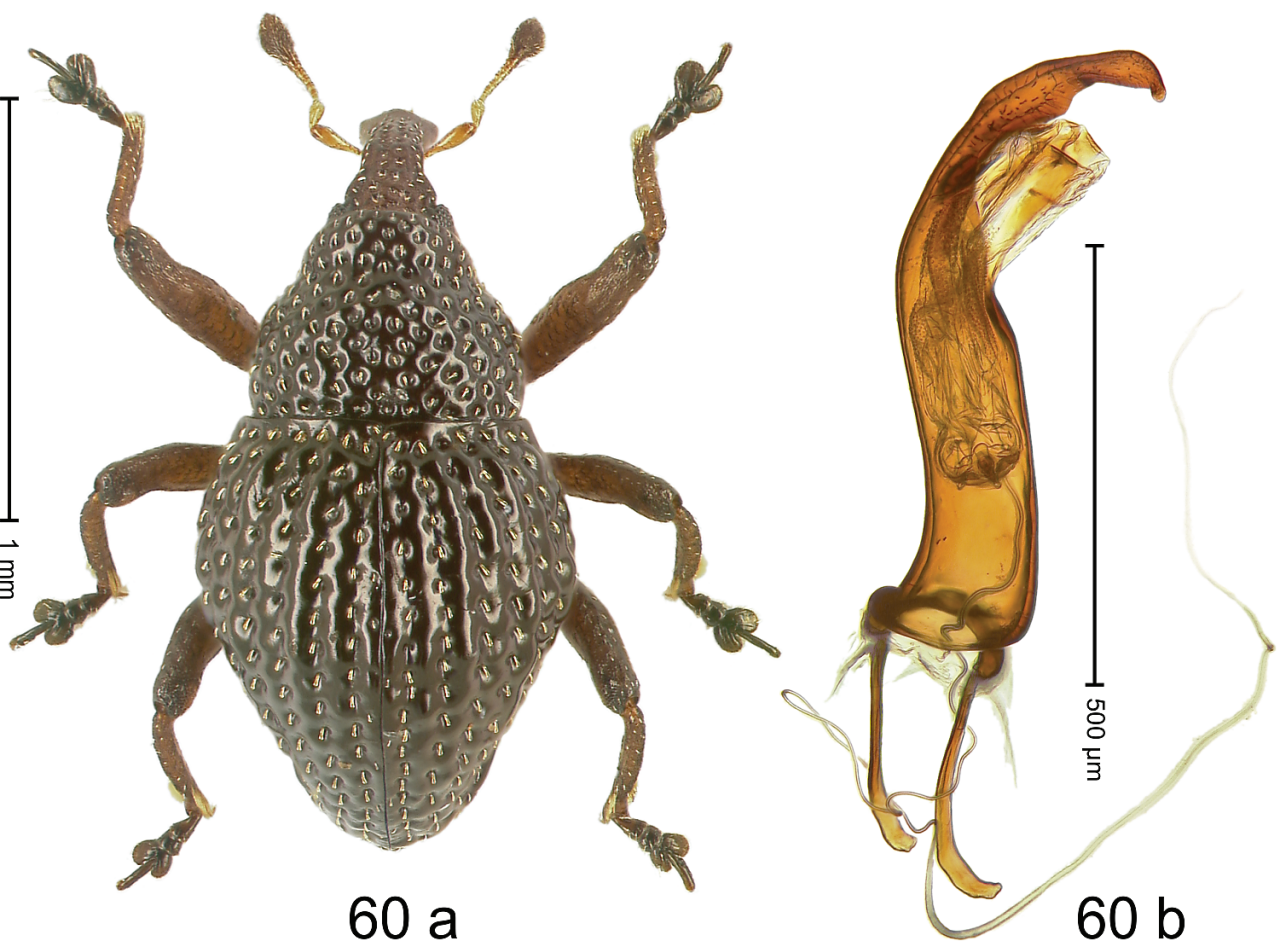
*Trigonopterus parvulus* Riedel, sp. n., holotype; (**a**) Habitus (**b**) Aedeagus.

Holotype, male (Fig. 60a). Length 1.54 mm. Antenna, legs, and head ferruginous; pronotum, elytra, and tarsi black. Body subovate; in dorsal aspect and in profile with distinct constriction between pronotum and elytron. Rostrum punctate; epistome simple. Eyes small, anterior margin angularly projecting. Pronotum with weak subapical constriction; disk densely, coarsely punctate; each puncture with short, yellowish scale. Elytra with striae deeply impressed; each puncture with suberect, yellowish scale; intervals subglabrous, costate, partly transversely confluent. Meso- and metafemur ventrally dentate. Metafemur subapically without stridulatory patch. Onychium ca. 1.6× longer than tarsomere 3. Aedeagus (Fig. 60b) with apex asymmetrical, right side forming long curved extension; basal orifice ventrally with rim; transfer apparatus dentiform; ductus ejaculatorius without bulbus. **Intraspecific variation**. Length 1.46–1.54 mm. Female rostrum slightly longer, dorsally subglabrous, sparsely punctate, in basal third coarsely punctate. Female elytral apex markedly extended ventrad, beak-shaped. Abdominal ventrite 3 of females with flattened process projecting over ventrite 4, medially with bifid extension reaching base of ventrite 5, sublaterally with pair of shorter spines. Female abdominal ventrites 4–5 weakly sclerotized.

##### Material examined.

Holotype (MZB): ARC0832 (EMBL # HE983631), WEST NEW GUINEA, Manokwari, Arfak Mts, Mokwam, Siyoubrig, S01°07.066', E133°54.710', 1870 m, 11-XII-2007, sifted. Paratypes (SMNK, ZSM): 23 exx, ARC0833 (EMBL # HE615515), ARC0834 (EMBL # HE615516), same data as holotype.

##### Distribution.

Manokwari Reg. (Arfak Mts). Elevation: 1870 m.

##### Biology.

Sifted from leaf litter in montane forest.

##### Etymology.

This epithet is based on the Latin adjective *parvulus* (little) and refers to the species´ small body size.

##### Notes.

*Trigonopterus parvulus* Riedel, sp. n. was coded as “*Trigonopterus* sp. 233” by Tänzler et al. (2012).

#### 61. Trigonopterus phoenix

Riedel sp. n.

urn:lsid:zoobank.org:act:76A1163B-D718-47F4-9466-2773F1EEDFB9

http://species-id.net/wiki/Trigonopterus_phoenix

##### Diagnostic description.

**Figure 61.**
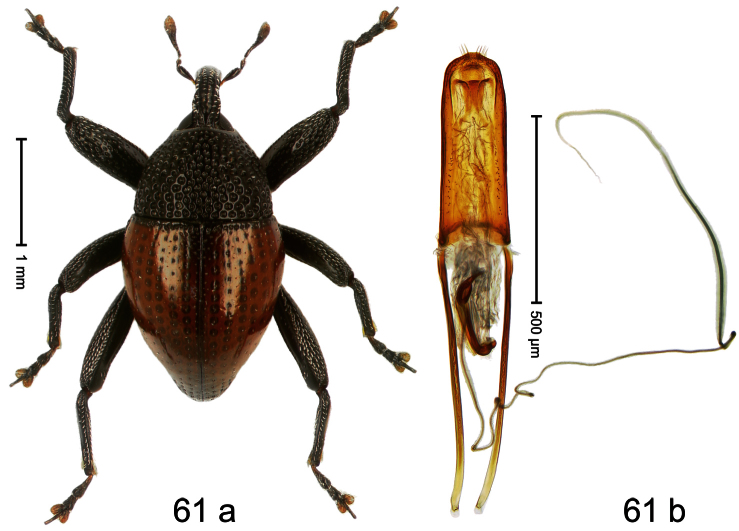
*Trigonopterus phoenix* Riedel, sp. n., holotype; (**a**) Habitus (**b**) Aedeagus.

Holotype, male (Fig. 61a). Length 2.63 mm. Color black; antennae, tarsi and elytra ferruginous. Body subovate; with weak constriction between pronotum and elytron; in profile evenly convex. Rostrum in basal half with distinct median ridge and pair of submedian ridges, furrows with sparse rows of yellowish scales; apically weakly punctate, sparsely setose. Pronotum coarsely punctate-reticulate. Elytra with distinct striae of small punctures; intervals with row of minute punctures; laterally behind humeri with ridge bordered by 4 deep punctures of stria 9. Femora edentate. Mesofemur and metafemur dorsally squamose with silvery scales. Metafemur with weakly denticulate dorsoposterior edge; subapically with stridulatory patch. Metatibia apically with uncus and minute premucro. Abdominal ventrite 5 coarsely punctate, in apical half with round depression fringed with dense erect scales. Aedeagus (Fig. 61b) apically weakly pointed, sparsely setose; transfer-apparatus spiniform; ductus ejaculatorius with bulbus. **Intraspecific variation**. Length 2.53–2.63 mm. Female rostrum in apical half slender, dorsally subglabrous, with sublateral furrows. Female abdominal ventrite 5 densely punctate, with suberect scales, with median ridge.

##### Material examined.

Holotype (SMNK): ARC1153 (EMBL # HE615781), PAPUA NEW GUINEA, Simbu Prov., Karimui Dist., Haia, Supa, S06°39.815', E145°03.169' to S06°39.609', E145°03.012', 1240–1450 m, 30-IX-2009. Paratypes (NAIC): PAPUA NEW GUINEA, Simbu Prov.: 1 ex, ARC1132 (EMBL # HE615761), S06°40.078', E145°03.207' to S06°39.609', E145°03.012', 1220–1450 m, 02-X-2009.

##### Distribution.

Simbu Prov. (Haia). Elevation: ca. 1240–1450 m.

##### Biology.

Collected by beating foliage in primary forest.

##### Notes.

*Trigonopterus phoenix* Riedel was coded as “*Trigonopterus* sp. 207” by Tänzler et al. (2012).

#### 62. Trigonopterus plicicollis

Riedel sp. n.

urn:lsid:zoobank.org:act:C92E1947-7151-4164-A216-68C190AD848E

http://species-id.net/wiki/Trigonopterus_plicicollis

##### Diagnostic description.

**Figure 62.**
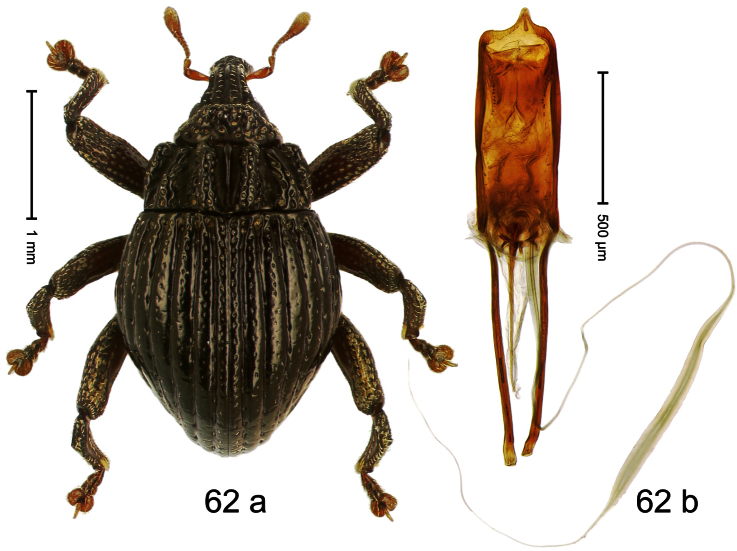
*Trigonopterus plicicollis* Riedel, sp. n., holotype; (**a**) Habitus (**b**) Aedeagus.

Holotype, male (Fig. 62a). Length 2.75 mm. Color black; legs deep ferruginous, antenna light ferruginous. Body in dorsal aspect and in profile with distinct constriction between pronotum and elytron. Rostrum in basal half with 3 ridges posteriorly continued to forehead; furrows each with 1 row of mesad directed setae; epistome weakly swollen, with transverse ridge. Pronotum deeply sculptured; with marked subapical constriction, apical collar with coarse punctures; disk with glabrous median ridge, on each side with additional three highly elevated coarsely punctate ridges; sides with oblique wrinkles and deep punctures. Elytra with striae deeply incised, towards sides with interspersed punctures; intervals costate, subglabrous, with sparse small punctures and wrinkles; apex extended ventrad, slightly beak-shaped. Femora edentate. Metafemur with denticulate dorsoposterior edge, subapically without stridulatory patch. Aedeagus (Fig. 62b) apically with submedian tooth shifted to the left; body at middle with broad depression visible in lateral aspect; transfer apparatus flagelliform, shorter than body, pointing basad; ductus ejaculatorius with bulbus. **Intraspecific variation**. Length 2.10–2.75 mm. Female rostrum in apical half dorsally flattened, subglabrous, with sparse punctures and lateral furrows; epistome simple.

##### Material examined.

Holotype (MZB): ARC0543 (EMBL # FN429249), WEST NEW GUINEA, Jayapura Reg., Cyclops Mts, Sentani, S02°31.683', E140°30.281', 960 m, 21-XI-2007, sifted. Paratypes (ARC, SMNK, ZSM): WEST NEW GUINEA, Jayapura Reg., Cyclops Mts, Sentani: 6 exx, same data as holotype; 2 exx, ARC0539 (EMBL # FN429245), ARC0540 (EMBL # FN429246), S02°31.594', E140°30.407', 1065 m, 21-XI-2007, sifted; 1 ex, 700 m, 22-XII-2004; 3 exx, 1000 m, 23-XII-2004, sifted; 1 ex, 1320 m, 23-XII-2004, sifted; 4 exx, 1100 m, 23-XII-2004, sifted.

##### Distribution.

Jayapura Reg. (Cyclops Mts). Elevation: 960–1320 m.

##### Biology.

Sifted from leaf litter in primary forest.

##### Etymology.

This epithet is based on a combination of the Latin nouns *plica* (fold) and *collum* (neck, pronotum) and refers to the surface of the pronotum.

##### Notes.

*Trigonopterus plicicollis* Riedel, sp. n. was coded as “*Trigonopterus* sp. 32” by Riedel et al. (2010) and Tänzler et al. (2012), respectively “*Trigonopterus* spaf” in the EMBL/GenBank/DDBJ databases.

#### 63. Trigonopterus politoides

Riedel sp. n.

urn:lsid:zoobank.org:act:336C5055-B4B6-4CAA-A6FE-332300F35FD0

http://species-id.net/wiki/Trigonopterus_politoides

##### Diagnostic description.

**Figure 63.**
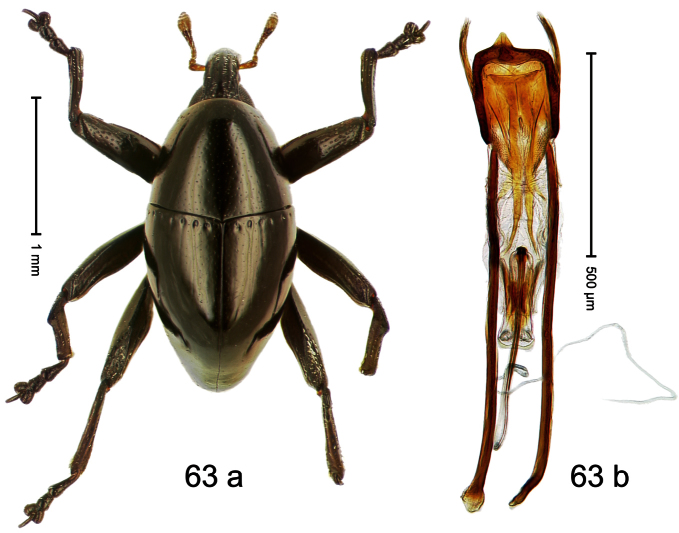
*Trigonopterus politoides* Riedel, sp. n., holotype; (**a**) Habitus (**b**) Aedeagus.

Holotype, male (Fig. 63a). Length 2.18 mm. Color black; antenna ferruginous. Body slender, ovate; without constriction between pronotum and elytron; in profile evenly convex. Rostrum in apical half smooth; in basal half with two pairs of longitudinal furrows containing sparse scales; above eye simple. Eyes with dorsal margin continuous with head, not carinate. Pronotum subglabrous, sparsely punctate with minute punctures. Elytra subglabrous, striae hardly visible but each with a deep pit along basal margin. Femora subglabrous; metafemur dorsally with silvery scales (partly abraded). Metafemur on posterior surface with longitudinal impression and row of scales; with smooth dorsoposterior edge; subapically without stridulatory patch. Mesotibia basally with external angulation, subapically simple, with uncus. Metatibia subapically simple, with uncus and few recumbent setae. Abdominal ventrite 5 with median impression. Aedeagus (Fig. 63b). Apex asymmetrical, with angular extension shifted to the right; laterally with dense brushes of weakly curved setae; proximal part of ductus ejaculatorius parallel to axis of aedeagus, enclosed by sclerites of transfer apparatus; ductus ejaculatorius without bulbus. **Intraspecific variation**. Length 2.18–2.51 mm. Female abdominal ventrite 5 flat.

##### Material examined.

Holotype (MZB): ARC0500 (EMBL # FN429207), WEST NEW GUINEA, Jayapura Reg., Cyclops Mts, Sentani, S02°32.2', E140°30.4', 545–700 m, 02-XII-2007, beaten. Paratypes (SMNK, ZSM): WEST NEW GUINEA, Jayapura Reg., Cyclops Mts, Sentani: 1 ex, ARC0501 (EMBL # FN429208), same data as holotype; 1 ex, ARC0503 (EMBL # FN429210), S02°32.0', E140°30.4', 700–900 m, 02-XII-2007, beaten.

##### Distribution.

Jayapura Reg. (Cyclops Mts). Elevation: 700 m.

##### Biology.

Collected by beating foliage.

##### Etymology.

This epithet is based on a combination of the name of *Trigonopterus politus* (Faust) and the Greek suffix *eides* (similar) and refers to their superficial similarity.

##### Notes.

*Trigonopterus politoides* Riedel, sp. n. was coded as “*Trigonopterus* sp. 25” by Riedel et al. (2010) and Tänzler et al. (2012), respectively “*Trigonopterus* spy” in the EMBL/GenBank/DDBJ databases.

#### 64. Trigonopterus pseudogranum

Riedel sp. n.

urn:lsid:zoobank.org:act:ECC19B3C-AC8F-4B57-A3EC-C29C95118BC6

http://species-id.net/wiki/Trigonopterus_pseudogranum

##### Diagnostic description.

**Figure 64.**
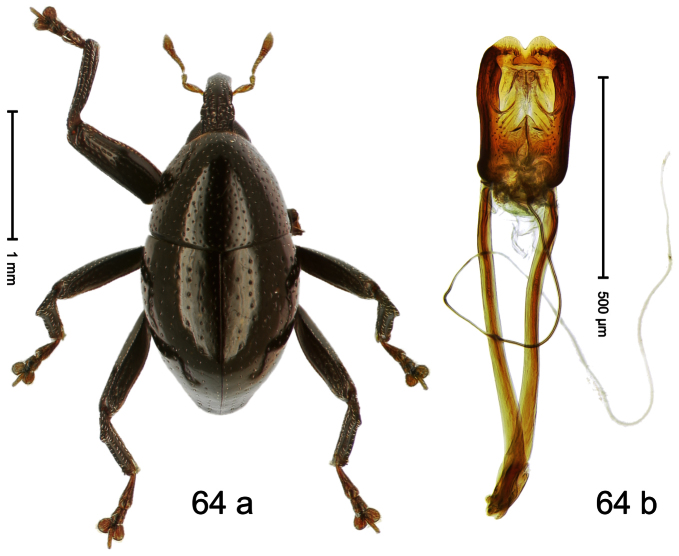
*Trigonopterus pseudogranum* Riedel, sp. n., holotype; (**a**) Habitus (**b**) Aedeagus.

Holotype, male (Fig. 64a). Length 2.26 mm. Color black; legs and rostrum dark ferruginous; antenna light ferruginous. Body laterally somewhat compressed, elongate-ovate, without constriction between pronotum and elytron; in profile evenly convex. Rostrum dorsally in basal third with low median ridge and pair of submedian ridges; apically subglabrous. Eyes large. Pronotum densely punctate, punctures dorsally small, laterally becoming larger, bearing each one minute seta; without scales. Elytra with striae distinct, punctures of stria 1–2 small, laterad strial punctures becoming larger, relatively shallow. Femora with anteroventral ridge. Profemur converging from base to apex. Meso- and metafemur with dorsoposterior edge subapically worn; metafemur subapically without stridulatory patch. Tibiae simple, without rows or brushes of long setae. Metatibia subapically with small suprauncal projection. Metaventrite laterally forming acute process over metacoxa, reaching tibial insertion. Metaventrite and abdominal ventrite 1 subglabrous, with sparse recumbent setae. Abdominal ventrite 2 similar to ventrites 3-4. Abdominal ventrite 5 with deep, subrotund cavity almost filling complete ventrite. Aedeagus (Fig. 64b) apically sinuate, with deep median incision; ductus ejaculatorius without bulbus. **Intraspecific variation**. Female rostrum subglabrous except in basal ¼ with ridges. Female abdominal ventrite 5 flat.

##### Material examined.

Holotype (MZB): ARC0774 (EMBL # HE615457), WEST NEW GUINEA, Manokwari, Arfak Mts, S01°01.465', E133°54.243', 685 m, 08-XII-2007, beaten. Paratype (ARC): 1 ex, WEST NEW GUINEA, Manokwari, Mt. Meja, 200 m, 18-III-1993, beaten.

##### Distribution.

Manokwari Reg. (Arfak Mts, Mt. Meja). Elevation: 200–685 m.

##### Biology.

Collected by beating foliage in primary forests.

##### Etymology.

This epithet is based on the Greek prefix *pseudo* (false) and the name of the sibling species *Trigonopterus granum*.

##### Notes.

*Trigonopterus pseudogranum* Riedel, sp. n. was coded as “*Trigonopterus* sp. 271” by Tänzler et al. (2012). It is closely related to *Trigonopterus granum* sp. n., *Trigonopterus pseudogranum* sp. n., and *Trigonopterus imitatus* sp. n. from which it can be distinguished by the denser punctation of the pronotum and the structure of the male abdominal ventrite 5. Despite its close morphological similarity its *cox1*-sequence diverges 10.4–13.1 % from the other species.

#### 65. Trigonopterus pseudonasutus

Riedel sp. n.

urn:lsid:zoobank.org:act:67C45350-3ABD-4AB6-AAD7-245B6C202360

http://species-id.net/wiki/Trigonopterus_pseudonasutus

##### Diagnostic description.

**Figure 65.**
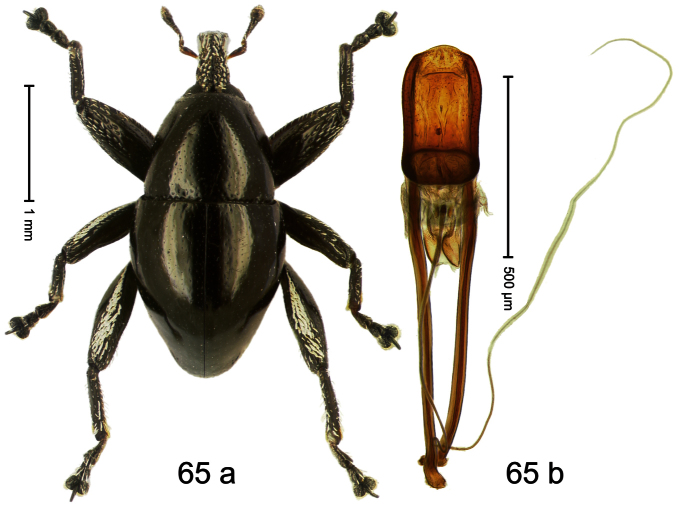
*Trigonopterus pseudonasutus* Riedel, sp. n., holotype; (**a**) Habitus (**b**) Aedeagus.

Holotype, male (Fig. 65a). Length 2.48 mm. Color black; legs deep ferruginous to black; antenna lighter ferruginous. Body subovate; in dorsal aspect and in profile with constriction between pronotum and elytron. Rostrum dorsally swollen, with distinct median carina, remainder coarsely punctate with erect white scales, subapical third subglabrous, weakly punctate. Eyes medially approximate. Pronotum subglabrous, with minute punctures, in front of elytral humeri with row of deep punctures, evenly rounded towards sides. Elytra subglabrous with minute punctures; striae obsolete; basal margin straight, simple. Femora with anteroventral ridge distinct, at base abruptly ending and forming markedly projecting blunt angle; at middle with inconspicuous tooth. Mesofemur and metafemur dorsally densely squamose with white scales. Metafemur with smooth dorsoposterior edge; subapically without stridulatory patch. Aedeagus (Fig. 65b) apically subangulate; ductus ejaculatorius subapically with weak bulbus. **Intraspecific variation**. Length 2.30–2.88 mm. Female rostrum in apical 2/3 dorsally flattened, subglabrous, sparsely punctate; basally swollen, with erect white scales.

##### Material examined.

Holotype (MZB): ARC0700 (EMBL # FN429344), WEST NEW GUINEA, Jayapura Reg., Cyclops Mts, Doyo, S02°32.5', E140°28.8', 300–400 m, 27-XI-2007, beaten. Paratypes (ARC, SMNK, ZSM): WEST NEW GUINEA, Jayapura Reg., Cyclops Mts: 1 ex, ARC0701 (EMBL # FN429345), same data as holotype; 9 exx, ARC0457 (EMBL # FN429168), Sentani, S02°32.2', E140°30.4', 545–700 m, 02-XII-2007; 1 ex, ARC0492 (EMBL # FN429199), Sentani, S02°31.7', E140°30.3', 850–1000 m, 30-XI-2007; 3 exx, ARC0495 (EMBL # FN429202), Sentani, S02°31.8', E140°30.5', 600–900 m, 28-XI-2007; 3 exx, S02°31.6', E140°30.4', 900–1100 m, 28-XI-2007, beaten; 2 exx, ARC0690 (EMBL # FN429334), ARC0691 (EMBL # FN429335), Sentani, S02°32.2', E140°30.4', 545–700 m, 02-XII-2007; 2 exx, S02°32.0', E140°30.4', 700–900 m, 02-XII-2007; 3 exx, Sentani, S02°32.2', E140°30.5', 500–600 m, 28-XI-07; 3 exx, Sentani, “Mim 1”, S02°32.166', E140°30.512', 600–620 m, 19-XI-07; 2 exx, Sentani, S02°31.794', E140°30.190', 800–860 m, 21-XI-07; 8 exx, Sentani, S02°32.3', E140°30.4', 350–620 m, 19-XI-07; 1 ex, Sentani, S02°31.8', E140°30.2', 630–800 m, 21-XI-2007; 1 ex, Sentani, 300–1400 m, 10-VIII-1991; 5 exx, Sentani, 600–1100 m, 05-X-1991; 3 exx, Sentani, 400–500 m, 10-VIII-1992; 4 exx, Sentani, 300–450 m, 07-10-VIII-1992; 4 exx, Sentani, 300–550 m, 02-X-1992; 1 ex (“marked as ARC0415”), 950–1450 m, 03-X-1992; 5 exx, Sentani, 300–500 m, 31-X-1992; 12 exx, Sentani, III-1992; 1 ex, Sentani, 350–850 m, 16-X-1996; 9 exx, Sentani, S02°32.535', E140°30.728', 250–385 m, 30-VI-2010.

##### Distribution.

Jayapura Reg. (Cyclops Mts). Elevation: 385–950 m.

##### Biology.

Collected by beating foliage in primary forests.

##### Etymology.

This epithet is based on the Greek prefix *pseudo* (false) and the name of *Trigonopterus nasutus* (Pascoe) which is superficially very similar and occurs sympatrically.

##### Notes.

*Trigonopterus pseudonasutus* Riedel, sp. n. was coded as “*Trigonopterus* sp. 22” by Riedel et al. (2010) and Tänzler et al. (2012), respectively “*Trigonopterus* spv” in the EMBL/GenBank/DDBJ databases.

#### 66. Trigonopterus ptolycoides

Riedel sp. n.

urn:lsid:zoobank.org:act:37E45438-EBB4-4FD0-BC0E-B72E4D9979F8

http://species-id.net/wiki/Trigonopterus_ptolycoides

##### Diagnostic description.

**Figure 66.**
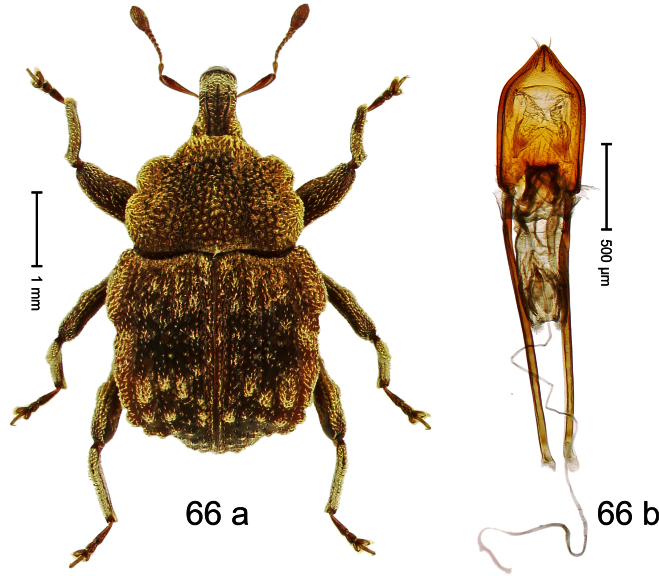
*Trigonopterus ptolycoides* Riedel, sp. n., holotype; (**a**) Habitus (**b**) Aedeagus.

Holotype, male (Fig. 66a). Length 4.28 mm. Color ferruginous, elytral disk with some irregular darker spots; integument partly covered with yellowish-brownish tomentum. Body dorsally flattened, with irregular lateral ridge; with constrictions at middle of pronotum, between pronotum and elytron, and in basal third of elytron. Rostrum in basal half with distinct median ridge and pair of submedian ridges; basally above eyes with pair of protrusions; apical ¼ subglabrous, punctulate. Pronotum with subapical constriction; with marked lateral flanges; coarsely rugose-punctate; punctures containing each one upcurved scale and much finer tomentum. Elytra with distinct striae; intervals with irregular tomentose tubercles; with marked lateral flanges; base bisinuate; apex extended ventrad, slightly beak-shaped. Femora narrow, parallel-sided, edentate. Meso- and metafemur dorsally with fringe of erect scales; metafemur subapically with stridulatory patch. Meso- and metatibia tapering from base to apex. Tarsi asymmetrical; tarsomere 3 with anterior lobe much larger than posterior lobe. Abdominal ventrite 5 apically deeply emarginate. Aedeagus (Fig. 66b) with body flattened, sides subparallel, apex pointed; transfer-apparatus short, spiniform; ductus ejaculatorius without bulbus. **Intraspecific variation**. Length 3.92–4.28 mm. Female abdominal ventrite 5 apically rounded.

##### Material examined.

Holotype (SMNK): ARC1415 (EMBL # HE615964), PAPUA NEW GUINEA, Eastern Highlands Prov., 37 km S Goroka, Hogave vill., Mt. Michael, S06°22.798', E145°15.427' to S06°22.925', E145°16.645', 2179–2800 m, 09-15-VII-2009, sifted. Paratypes (NAIC, SMNK, ZSM): 6 exx, ARC1416 (EMBL # HE615965), ARC1417 (EMBL # HE615966), same data as holotype.

##### Distribution.

Eastern Highlands Prov. (Mt. Michael). Elevation: ca. 2179–2800 m.

##### Biology.

Sifted from leaf litter in primary forest.

##### Etymology.

This epithet is a combination of the genus name *Ptolycus* and the Latin suffix *-oides* (having the form of) and refers to the species´ resemblance in habitus.

##### Notes.

*Trigonopterus ptolycoides* Riedel, sp. n. was coded as “*Trigonopterus* sp. 68” by Tänzler et al. (2012).

#### 67. Trigonopterus punctulatus

Riedel sp. n.

urn:lsid:zoobank.org:act:76389482-C7E2-49BA-9A72-900EAE695906

http://species-id.net/wiki/Trigonopterus_punctulatus

##### Diagnostic description.

**Figure 67.**
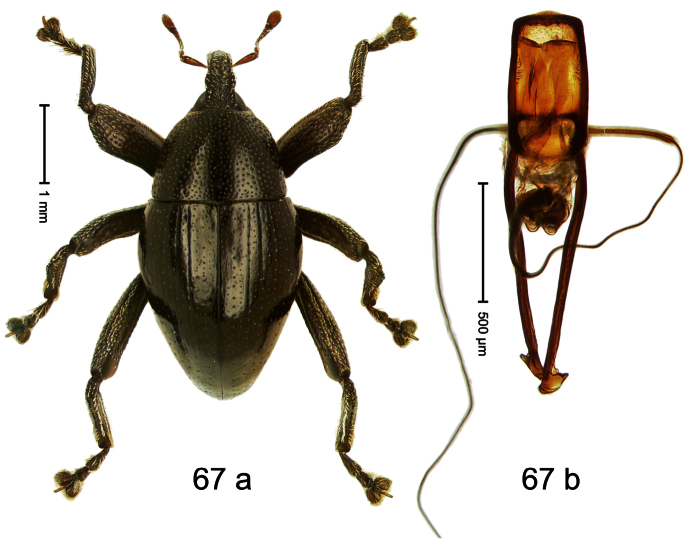
*Trigonopterus punctulatus* Riedel, sp. n., holotype; (**a**) Habitus (**b**) Aedeagus.

Holotype, male (Fig. 67a). Length 3.72 mm. Color black, legs and antenna deep ferruginous. Body ovate; almost without constriction between pronotum and elytron; in profile evenly convex. Rostrum dorsally tricarinate, with distinct median and pair of lateral carinae to shortly before apex. Pronotum densely punctate. Elytra densely punctate; strial punctures slightly larger than minute punctures on intervals; striae impressed as fine lines; lateral stria behind humeri simple, not deepened. Femora edentate. Metafemur with denticulate dorsoposterior edge; subapically with stridulatory patch. Mesotibia subapically narrow, subbasally widened, dorsal contour with angulation. Metatibia without premucro; with small suprauncal projection. Aedeagus (Fig. 67b) apically subangulate; dorsum sublaterally sparsely setose; transfer apparatus spiniform, curved; ductus ejaculatorius with bulbus. **Intraspecific variation**. Length 2.92–3.92 mm. Female rostrum basally punctate, towards apex rugose-punctate.

##### Material examined.

Holotype (MZB): ARC419 (EMBL # FN429130), WEST NEW GUINEA, Jayapura Reg., Cyclops Mts, Sentani, S02°31.7', E140°30.3', 900–1150 m, 21-XI-2007, beaten, marked “stridul. 2”. Paratypes (ARC, NHMB, SMNK, ZSM): WEST NEW GUINEA, Jayapura Reg., Cyclops Mts, Sentani: 10 exx, ARC0424 (EMBL # FN429135), ARC0482 (EMBL # FN429189), S02°31.3', E140°30.5', 1200–1420 m, 30.XI.2007; 1 ex, ARC0664 (EMBL # FN429311), S02°31.8', E140°30.2', 630–800 m, 21-XI-2007; 8 exx, S02°31.794', E140°30.190', 800–860 m, 21-XI-2007, “Mim2”, beaten; 8 exx, S02°31.7', E140°30.3', 860–1150 m, 21-XI-2007, beaten; 7 exx, S02°31.6', E140°30.4', 900–1100 m, 28-XI-2007, beaten; 3 exx, ARC0680 (EMBL # FN429325), ARC0681 (EMBL # FN429326), S02°31.2', E140°30.5', 1420–1520 m, 30-XI-2007; 1 ex, ARC0696 (EMBL # FN429340), S02°32.2', E140°30.4', 545–700 m, 02-XII-2007; 5 exx, S02°31.7', E140°30.3', 850–1000 m, 30-XI-2007, beaten; 5 exx, S02°31.6', E140°30.4', 1000–1200 m, 30-XI-2007, beaten; 1 ex, S02°31.603', E140°30.434', 1095 m, 28-XI-2007, sifted; 14 exx [1 marked “ARC 041”], 950–1450 m, 03-X-1992; 4 exx, 1100–1600 m, 05-X1991; 7 exx, 1200–1400 m, 09-VIII-1992; 10 exx, 1100–1600 m, 05-X-1991; 5 exx, 1400 m, 10-VIII-1991; 5 exx, 950–1450 m, 03-X-1992; 5 exx, Lake Sentani, 300 m, III-1992.

##### Distribution.

Jayapura Reg. (Cyclops Mts). Elevation: 300–1420 m.

##### Biology.

Collected by beating foliage in primary forests.

##### Etymology.

This epithet is based on the Latin participle *punctulatus* (provided with little punctures) and refers to the species´ surface scattered with small punctures.

##### Notes.

*Trigonopterus punctulatus* Riedel, sp. n. was coded as “*Trigonopterus* sp. 2” by Riedel et al. (2010) and Tänzler et al. (2012), respectively “*Trigonopterus* spb” in the EMBL/GenBank/DDBJ databases.

#### 68. Trigonopterus ragaorum

Riedel sp. n.

urn:lsid:zoobank.org:act:FE9AA1AF-43EF-4A40-9EB5-54D2AF0702CF

http://species-id.net/wiki/Trigonopterus_ragaorum

##### Diagnostic description.

**Figure 68.**
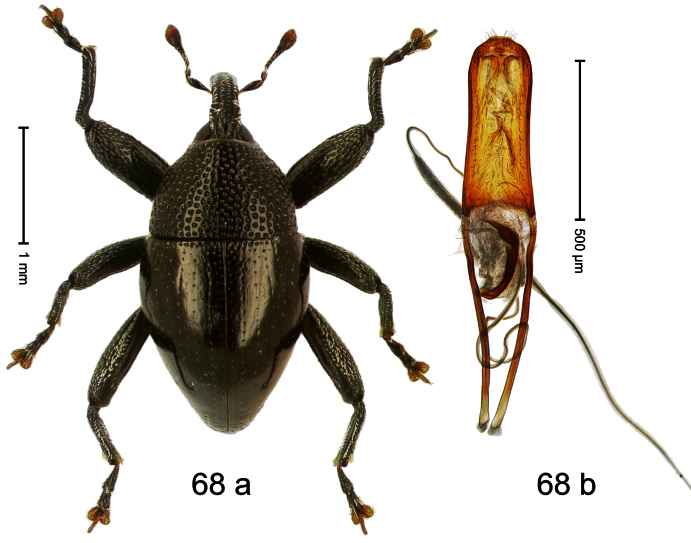
*Trigonopterus ragaorum* Riedel, sp. n., holotype; (**a**) Habitus (**b**) Aedeagus.

Holotype, male (Fig. 68a). Length 2.57 mm. Color black; antennae and tarsi ferruginous. Body subovate; with weak constriction between pronotum and elytron; in profile evenly convex. Rostrum in basal half with distinct median ridge and pair of submedian ridges, furrows with sparse rows scales and setae; apically weakly punctate, sparsely setose. Pronotum punctate-reticulate. Elytra with striae impressed as very fine lines; strial punctures very small; intervals flat, subglabrous; basal margin bordered by row of small punctures; laterally behind humeri with ridge bordered by 4 deep punctures of stria 9. Femora edentate. Mesofemur and metafemur dorsally squamose with silvery scales. Metafemur with weakly denticulate dorsoposterior edge; subapically with stridulatory patch. Metatibia apically with uncus and minute premucro. Abdominal ventrite 5 densely setose with erect setae. Aedeagus (Fig. 68b) apically weakly pointed, sparsely setose; body dorsally with 2 rows of sparse setae; in profile apical 2/3 of body markedly curved ventrad; transfer-apparatus spiniform; ductus ejaculatorius with bulbus. **Intraspecific variation**. Length 2.30–2.65 mm. Female rostrum in apical half dorsally subglabrous, sparsely punctate, with shallow furrows. Female abdominal ventrite 5 with subrecumbent setae.

##### Material examined.

Holotype (SMNK): ARC1876 (EMBL # HE616153), PAPUA NEW GUINEA, Eastern Highlands Prov., Aiyura, S06°21.033', E145°54.597', 2169 m, 06-II-2010. Paratypes (NAIC, SMNK, ZSM): PAPUA NEW GUINEA: 29 exx, ARC1877 (EMBL # HE616154), ARC1878 (EMBL # HE616155), ARC1879 (EMBL # HE616156), ARC1880 (EMBL # HE616157), ARC1881 (EMBL # HE616158), same data as holotype; 1 ex, ARC0354 (EMBL # HE615167), Aiyura, S06°21.131', E145°54.398', 1670 m, 05-IV-2006.

##### Distribution.

Eastern Highlands Prov. (Aiyura). Elevation: 1670–2169 m.

##### Biology.

Beaten from foliage of montane forests.

##### Etymology.

This species is dedicated to the people of Papua New Guinea. The epithet is based on the family name Raga, found on page 315 of the Papua New Guinea Telephone Directory of 2010 and treated in genitive plural.

##### Notes.

*Trigonopterus ragaorum* Riedel, sp. n. was coded as “*Trigonopterus* sp. 208” by Tänzler et al. (2012).

#### 69. Trigonopterus rhinoceros

Riedel sp. n.

urn:lsid:zoobank.org:act:E52A2105-B7CB-4350-A8F3-9E8B78A8C25E

http://species-id.net/wiki/Trigonopterus_rhinoceros

##### Diagnostic description.

**Figure 69.**
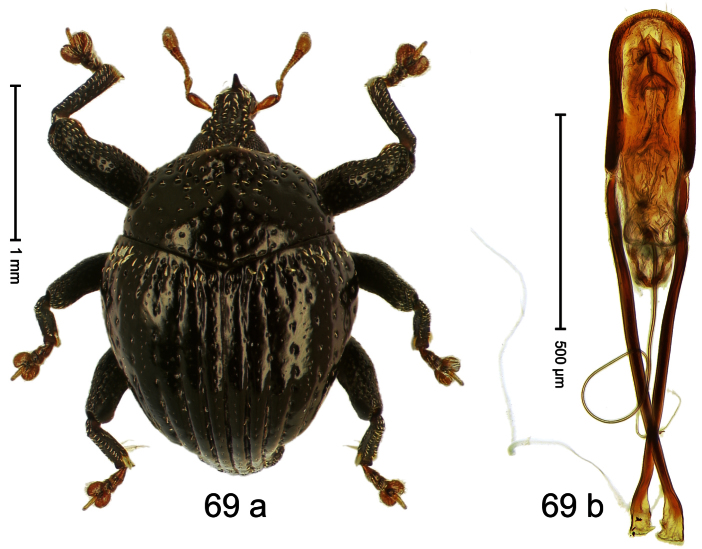
*Trigonopterus rhinoceros* Riedel, sp. n., holotype; (**a**) Habitus (**b**) Aedeagus.

Holotype, male (Fig. 69a). Length 2.14 mm. Color black; tarsi ferruginous; antenna light ferruginous. Body subglobose; with weak constriction between pronotum and elytron; in profile evenly convex. Rostrum in basal half with median ridge and pair of submedian ridges, at middle with tubercle; apical half laterally bordered by ridges; epistome at middle with dorsoposteriad directed horn. Pronotum basally angulate; apex, base, and sides densely punctate, disk medially and sublaterally subglabrous. Elytra with suture and striae 1–3 incised; all striae marked by small punctures; intervals subglabrous, with row of minute punctures; base with yellowish, posteriad directed scales; interval 7 subapically swollen. Meso- and metafemur with anteroventral ridge weakly dentate. Metatibia ventrally in apical half with row of long setae. Metafemur with stridulatory patch. Aedeagus (Fig. 69b) with apex rounded; endophallus distally with brace-shaped sclerite; ductus ejaculatorius, without bulbus. **Intraspecific variation**. Length 1.76–2.26 mm. Female rostrum dorsally flattened, medially subglabrous, with rows of punctures and lateral furrows; epistome simple. Female metatibia subapically simple, without long setae.

##### Material examined.

Holotype (MZB): ARC0554 (EMBL # FN429260), WEST NEW GUINEA, Jayapura Reg., Cyclops Mts, Sentani, S02°31.182', E140°30.542', 1510 m, 30-XI-2007, sifted. Paratypes (ARC, SMNK, ZSM): WEST NEW GUINEA, Jayapura Reg., Cyclops Mts, Senani: 7 exx, ARC0555 (EMBL # FN429261), ARC0562 (EMBL # FN429268), same data as holotype; 1 ex, S02°31.383', E140°30.490', 1275 m, 30-XI-2007, sifted; 1 ex, S02°31.281', E140°30.535', 1420 m, 30-XI-2007, sifted; 4 exx (1 marked ARC0095), 1320 m, 23-XII-2004, sifted; 2 exx, 300–1400 m, 10-VIII-1991; 1 ex, 1200–1400 m, 09-VIII-1992, sifted.

##### Distribution.

Jayapura Reg. (Cyclops Mts). Elevation: 1275–1510 m.

##### Biology.

Sifted from leaf litter in montane crippled forest.

##### Etymology.

This epithet is a noun in apposition and refers to the horn on the rostral apex, resembling the one of a mammal rhinoceros.

##### Notes.

*Trigonopterus rhinoceros* Riedel, sp. n. was coded as “*Trigonopterus* sp. 44” by Riedel et al. (2010) and Tänzler et al. (2012), respectively “*Trigonopterus* spar” in the EMBL/GenBank/DDBJ databases.

#### 70. Trigonopterus rhomboidalis

Riedel sp. n.

urn:lsid:zoobank.org:act:E072A3FE-B753-4634-ACAC-4548BD13EDB8

http://species-id.net/wiki/Trigonopterus_rhomboidalis

##### Diagnostic description.

**Figure 70.**
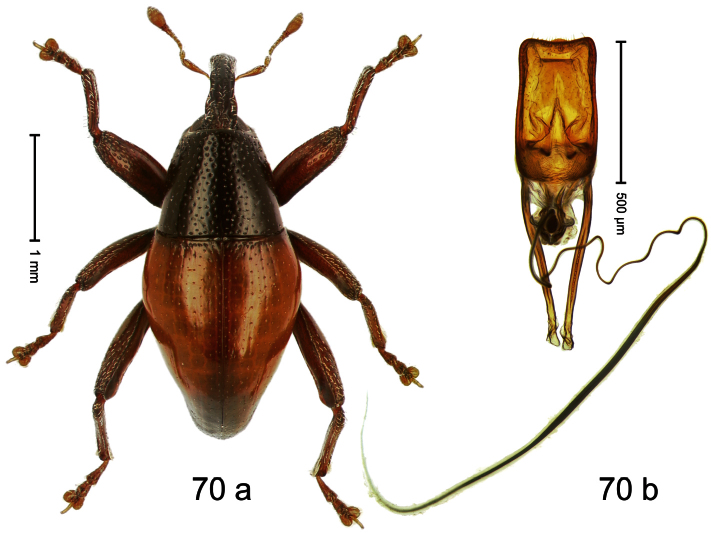
*Trigonopterus rhomboidalis* Riedel, sp. n., holotype; (**a**) Habitus (**b**) Aedeagus.

Holotype, male (Fig. 70a). Length 2.98 mm. Color orange-ferruginous, head and pronotum black. Body subrhomboid; almost without constriction between pronotum and elytron; in profile evenly convex. Rostrum with weak median and pair of submedian costae, basally with few distinct punctures; in apical 1/3 weakly rugose-punctate; sparsely setose. Pronotum punctate with small punctures becoming larger from base to apex; sides anteriorly with large punctures, posteriorly subglabrous. Elytra with striae indistinct, marked by small to minute punctures, intervals with row of minute punctures. Femora edentate, with distinct anteroventral ridge. Metafemur posteriorly subglabrous except subapically with stridulatory patch. Aedeagus (Fig. 70b) with sides of body subparallel, apex subtruncate, with small median tip; endophallus with two pairs of narrow sclerites; complex transfer-apparatus symmetrical; ductus ejaculatorius with bulbus. **Intraspecific variation**. Length 2.43–3.03 mm. Punctures of pronotum relatively small in specimens from Poga, especially basally; in specimens from Bokondini and Lake Habbema pronotum evenly coarsely punctate except along subglabrous midline.

##### Material examined.

Holotype (MZB): ARC1753 (EMBL # HE616030), WEST NEW GUINEA, Jayawijaya Reg., Poga, S03°47.631', E138°35.459' to S03°47.406', E138°35.507', 2260–2410 m, 14-VII-2010. Paratypes (ARC, NHMB, SMNK, ZSM): WEST NEW GUINEA, Jayawijaya Reg.: 3 exx, ARC1755 (EMBL # HE616032), same data as holotype; 1 ex, ARC1752 (EMBL # HE616029), Poga, S03°47.575', E138°33.155' to S03°47.473', E138°33.163', 2620–2715 m, 15-VII-2010; 1 ex, ARC1714 (EMBL # HE615994), Jiwika, Kurulu, S03°57.161', E138°57.357' to S03°56.977', E138°57.441', 1875–1990 m, 12-VII-2010; 2 exx, Jiwika, 1800–2300 m, 31-V-1998; 2 exx, Ilugwa, trail to Pass valley, 1900–2500 m, 14-IX-1990; 1 ex, ARC1806 (EMBL # HE616083), Bokondini, S03°40.345', E138°42.386' to S03°40.255', E138°42.189', 1655–1700 m, 18-VII-2010; 8 exx, ARC1815 (EMBL # HE616092), ARC1816 (EMBL # HE616093), W Wamena, road to Lake Habbema, S04°07.625', E138°49.992', 2520 m, 20-VII-2010; 62 exx, W Wamena, road to Lake Habbema, S04°08.256', E138°49.049', 2770 m, 20-VII-2010; 21 exx, Baliem-vall., ca. 1700 m, III-1992.

##### Distribution.

Jayawijaya Reg. (Bokondini, Poga, Ilugwa, Jiwika, L. Habbema). Elevation: 1700–2770 m.

##### Biology.

Beaten from foliage of montane forests.

##### Etymology.

This epithet is based on the Latin adjective *rhomboidalis* (shaped like a rhomboid) and refers to the body-shape.

##### Notes.

*Trigonopterus rhomboidalis* Riedel, sp. n. was coded as “*Trigonopterus* sp. 81” by Tänzler et al. (2012).

#### 71. Trigonopterus rubiginosus

Riedel sp. n.

urn:lsid:zoobank.org:act:B5E826C2-162D-4CF8-B781-6DCFFC180673

http://species-id.net/wiki/Trigonopterus_rubiginosus

##### Diagnostic description.

**Figure 71.**
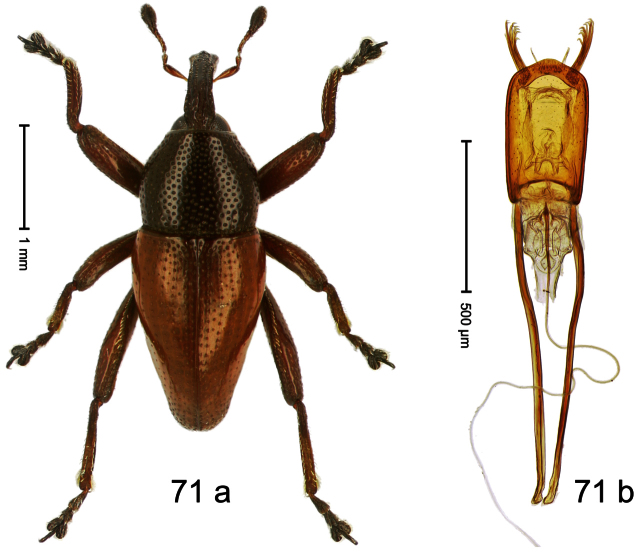
*Trigonopterus rubiginosus* Riedel, sp. n., holotype; (**a**) Habitus (**b**) Aedeagus.

Holotype, male (Fig. 71a). Length 2.93 mm. Color orange-ferruginous; pronotum, tarsi and antennal club black. Body elongate; with distinct constriction between pronotum and elytron; in profile dorsally flat. Rostrum with distinct median and pair of submedian costae, in apical 1/3 scabrous. Pronotum densely punctate; interspaces subequal to puncture´s diameter. Elytra with striae marked by small punctures and hardly visible fine lines, intervals with smaller punctures interspersed; intervals 1–3 near base with larger and denser punctures. Mesocoxa with densely setose patch. Femora edentate. Profemur in basal third posteriorly with callus. Metafemur dorsally with row of white scales; subapically with indistinct stridulatory patch interspersed with coarse punctures. Metatibia ventrally with sparse row of long setae. Aedeagus (Fig. 71b) apically subangulate, with brushes of stout, apically hooked setae; body flattened, sides subparallel; transfer apparatus simple, spiniform; ductus ejaculatorius without bulbus. **Intraspecific variation**. Length 2.81–3.09 mm. No female specimen available.

##### Material examined.

Holotype (MZB): ARC1756 (EMBL # HE616033), WEST NEW GUINEA, Jayawijaya Reg., Poga, S03°47.631', E138°35.459' to S03°47.406', E138°35.507', 2260–2410 m, 14-VII-2010. Paratypes (ARC, SMNK, ZSM): WEST NEW GUINEA, Jayawijaya Reg.: 13 exx, ARC1736 (EMBL # HE616013), ARC1737 (EMBL # HE616014), ARC1738 (EMBL # HE616015), Poga, S03°48.382', E138°34.780', 2285–2345 m, 13-VII-2010; 1 ex (marked as “ARC0033”), Jiwika, 1800–2300 m, 31-V-1998; 1 ex, Ilugwa, Melanggama, 1900–2200 m, 09-12-IX-1990.

##### Distribution.

Jayawijaya Reg. (Jiwika, Ilugwa, Poga). Elevation: 2200–2285 m.

##### Biology.

Beaten from foliage of montane forests.

##### Etymology.

This epithet is based on the Latin adjective *rubiginosus* (rusty-red)

##### Notes.

*Trigonopterus rubiginosus* Riedel, sp. n. was coded as “*Trigonopterus* sp. 248” by Tänzler et al. (2012).

#### 72. Trigonopterus rubripennis

Riedel sp. n.

urn:lsid:zoobank.org:act:EB5923DD-599C-4CEA-9EA2-EA61BA28D9F1

http://species-id.net/wiki/Trigonopterus_rubripennis

##### Diagnostic description.

**Figure 72.**
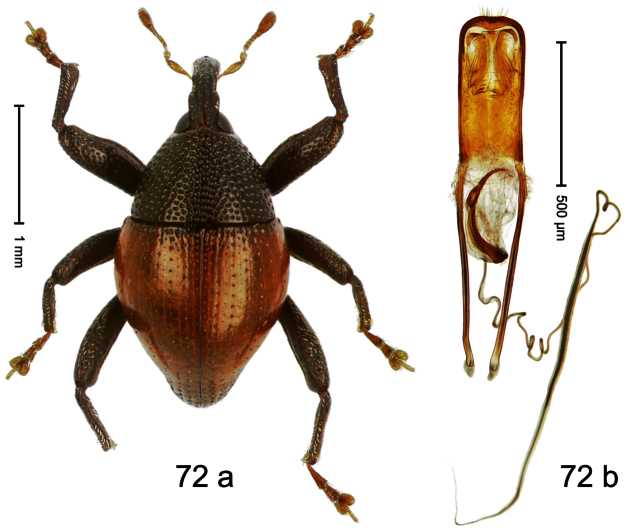
*Trigonopterus rubripennis* Riedel, sp. n., holotype; (**a**) Habitus (**b**) Aedeagus.

Holotype, male (Fig. 72a). Length 2.43 mm. Color black; antennae, legs and elytra ferruginous. Body subrhomboid; with weak constriction between pronotum and elytron; in profile evenly convex. Rostrum in basal half with distinct median ridge and pair of submedian ridges; with lateral constriction; sparsely setose. Pronotum densely punctate-reticulate. Elytra with striae distinct; strial punctures small; intervals with row of minute punctures; laterally behind humeri with ridge bordered by 4 deeper punctures of stria 9. Femora edentate. Anteroventral ridge of profemur in apical 1/3 shortened, forming weak angulation. Mesofemur and metafemur dorsally sparsely squamose. Metafemur subapically with stridulatory patch. Metatibia apically with uncus and minute premucro. Abdominal ventrite 5 with shallow depression and patch of dense erect setae. Aedeagus (Fig. 72b) apically weakly pointed, sparsely setose; body dorsally with two combs of setae; transfer-apparatus spiniform, curved; ductus ejaculatorius with bulbus. **Intraspecific variation**. Length 2.14–2.43 mm. Female rostrum in apical half subglabrous, sparsely punctate. Female abdominal ventrite 5 flat, with subrecumbent setae.

##### Material examined.

Holotype (SMNK): ARC1834 (EMBL # HE616111), PAPUA NEW GUINEA, Eastern Highlands Prov., Okapa, Konafi to Isimomo, S06°25.593', E145°34.862', S06°25.003', E145°34.527', 1911–2131 m, 18-III-2010. Paratypes (NAIC, SMNK, ZSM): PAPUA NEW GUINEA, Eastern Highlands Prov.: 4 exx, ARC1835 (EMBL # HE616112), same data as holotype; 1 ex, Okapa, Isimomo, S06°25.003', E145°34.527', 2131 m, 22-XII-2010; 7 exx, ARC1833 (EMBL # HE616110), Okapa, Kimiagomo village, Hamegoya, S06°25.727', E145°35.455', S06°25.117', E145°35.225', 1891–2131 m, 18-III-2010; 1 ex, Okapa, Afiyaleto village, S06°25.593', E145°34.862' to S06°25.212', E145°35.498', 1911 m, 18-III-2010, beaten.

##### Distribution.

Eastern Highlands Prov. (Okapa). Elevation: ca. 1911–2131 m.

##### Biology.

Beaten from foliage of montane forests.

##### Etymology.

This epithet is based on a combination of the Latin adjective *ruber* (red) and the noun *penna* (wing, elytron).

##### Notes.

*Trigonopterus rubripennis* Riedel, sp. n. was coded as “*Trigonopterus* sp. 206” by Tänzler et al. (2012).

#### 73. Trigonopterus rufibasis

Riedel sp. n.

urn:lsid:zoobank.org:act:A01D3C2B-6BB0-451E-B8CB-077A16A49BD3

http://species-id.net/wiki/Trigonopterus_rufibasis

##### Diagnostic description.

**Figure 73.**
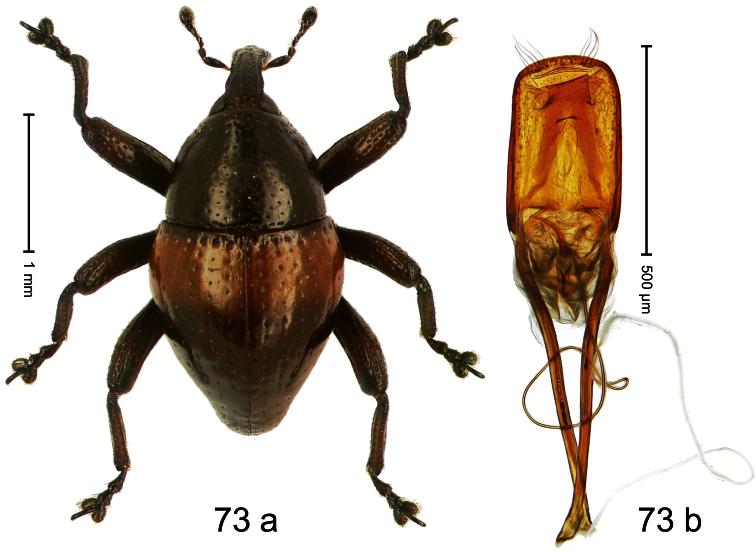
*Trigonopterus rufibasis* Riedel, sp. n., holotype; (**a**) Habitus (**b**) Aedeagus.

Holotype, male (Fig. 73a). Length 2.38 mm. Color black; basal 1/3 of elytra ferruginous; legs dark ferruginous except tarsi, knees and base of femora black. Body subovate; with weak constriction between pronotum and elytron; in profile almost evenly convex. Rostrum dorsally relatively flat, with two submedian rows of punctures, dorsolaterally with pair of furrows continuing along eye. Pronotum sparsely punctate with minute punctures, subapically punctures larger and denser. Elytra with striae distinct, dorsally punctures small, along base and laterally behind humeri punctures large; intervals subglabrous. Femora edentate. Metafemur with simple dorsoposterior edge; subapically with stridulatory patch. Aedeagus (Fig. 73b) apically rounded, sparsely setose; transfer apparatus spiniform, curved, much shorter than body; ductus ejaculatorius without bulbus. **Intraspecific variation**. Length 1.91–2.55 mm. Color of legs ferruginous or black. Female rostrum dorsally with punctures smaller and sparser than in male.

##### Material examined.

Holotype (MZB): ARC1764 (EMBL # HE616041), WEST NEW GUINEA, Jayawijaya Reg., Poga, S03°47.575', E138°33.155' to S03°47.473', E138°33.163', 2620–2715 m, 15-VII-2010. Paratypes (SMNK, ZSM): WEST NEW GUINEA, Jayawijaya Reg., Poga: 12 exx, ARC1765 (EMBL # HE616042), ARC1766 (EMBL # HE616043), same data as holotype.

##### Distribution.

Jayawijaya Reg. (Poga). Elevation: ca. 2620–2715 m.

##### Biology.

Beaten from foliage of upper montane forests.

##### Etymology.

This epithet is based on the combination of the Latin adjective *rufus* (reddish) and the noun *basis* (base) and refers to the elytral coloration.

##### Notes.

*Trigonopterus rufibasis* Riedel, sp. n. was coded as “*Trigonopterus* sp. 124” by Tänzler et al. (2012).

#### 74. Trigonopterus scabrosus

Riedel sp. n.

urn:lsid:zoobank.org:act:41D1ED4D-256D-40EA-959C-7E2978F22AF5

http://species-id.net/wiki/Trigonopterus_scabrosus

##### Diagnostic description.

**Figure 74.**
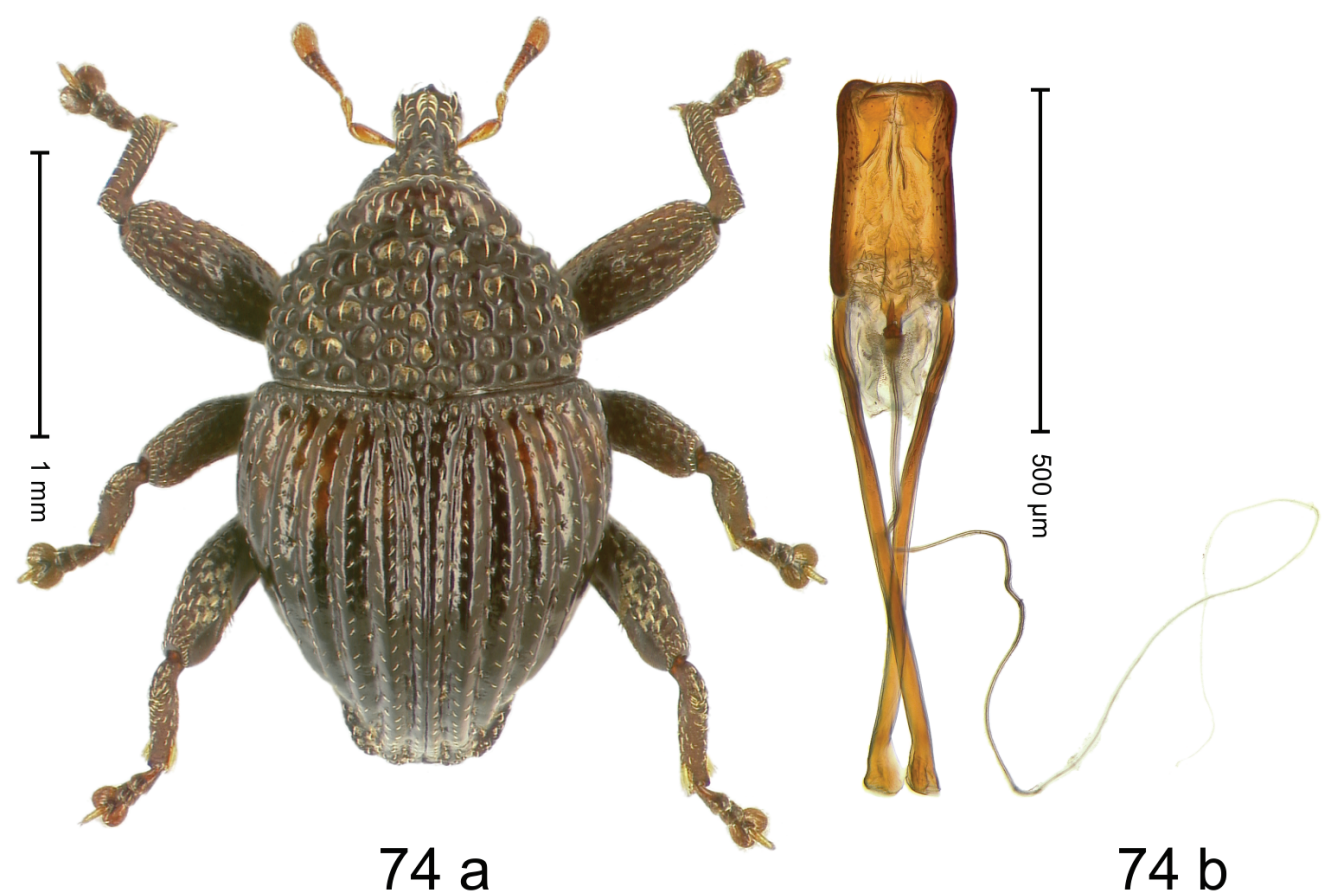
*Trigonopterus scabrosus* Riedel, sp. n., holotype; (**a**) Habitus (**b**) Aedeagus.

Holotype, male (Fig. 74a). Length 2.08 mm. Color of legs and elytral base dark ferruginous; antenna light ferruginous; remainder black. Body subovate, in dorsal aspect and in profile with distinct constriction between pronotum and elytron. Rostrum with median carina bordered by pair of sublateral furrows, posteriorly continued to hind-level of eyes; at level of antennal insertion median carina high, denticulate; with sparse, erect scales; epistome with transverse, angulate ridge provided with three teeth, medially with horn and laterally with pair of teeth. Pronotum with subapical constriction; disk coarsely punctate, areolate. Elytra with striae deeply incised, intervals costate, sparsely setose with minute recumbent setae; interval 7 subapically forming ridge; apex subangulate. Femora edentate. Metafemur with weakly denticulate dorsoposterior edge, in apical third with transverse row of small suberect setae, subapically with stridulatory patch. Abdominal ventrites 1–2 cavernous. Abdominal ventrite 2 projecting dentiform over elytral edge in profile. Aedeagus (Fig. 74b) apically subtruncate; transfer apparatus small, tridentate; ductus ejaculatorius without bulbus. **Intraspecific variation**. Length 1.90–2.45 mm. Color of elytra ranging from almost black (except base of intervals 4–5 ferruginous) to ferruginous in basal half. Female rostrum dorsally with low median costa bearing two rows of punctures, bordered by pair of lateral furrows, without protrusions; epistome simple.

##### Material examined.

Holotype (MZB): ARC0759 (EMBL # HE615442), WEST NEW GUINEA, Manokwari, Mt. Meja, S00°51.497’, E134°04.949’, 220 m, 05-XII-2007, sifted. Paratypes (ARC, SMNK, ZSM): WEST NEW GUINEA, Manokwari, Mt. Meja: 3 exx, ARC0760 (EMBL # HE615443), ARC0761 (EMBL # HE615444), same data as holotype; 1 ex, 22-23-IX-1990, sifted; 4 exx, 200 m, 30-XII-2004, sifted.

##### Distribution.

Manokwari Reg. (Mt. Meja). Elevation: 200–220 m.

##### Biology.

Sifted from leaf litter in lowland forest.

##### Etymology.

This epithet is based on the Latin adjective *scabrosus* (rough) and refers to sculpture, especially the one of the pronotum.

##### Notes.

*Trigonopterus scabrosus* Riedel, sp. n. was coded as “*Trigonopterus* sp. 204” by Tänzler et al. (2012).

#### 75. Trigonopterus scissops

Riedel sp. n.

urn:lsid:zoobank.org:act:5E783DAA-30CE-41D6-A8DE-0FEE8E94B143

http://species-id.net/wiki/Trigonopterus_scissops

##### Diagnostic description.

**Figure 75.**
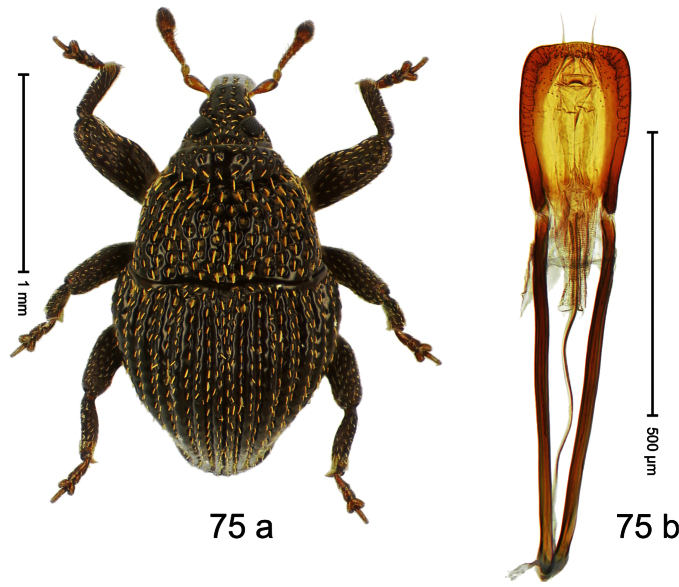
*Trigonopterus scissops* Riedel, sp. n., holotype; (**a**) Habitus (**b**) Aedeagus.

Holotype, male (Fig. 75a). Length 1.78 mm. Color black; antenna and tarsi ferruginous; tibiae and femora dark ferruginous. Body subovate; in dorsal aspect and in profile with marked constriction between pronotum and elytron. Rostrum with submedian and sublateral pair of furrows; epistome simple. Eyes large, divided into dorsal and ventral portions by marked incision of posterior margin. Pronotum with marked subapical constriction; densely punctate with deep punctures; each puncture with one narrow, elongate, ochre scale. Elytra with striae deeply incised; intervals costate, each with one row of narrow scales. Metafemur subapically without stridulatory patch. Tibia apically confluent with stout uncus. Onychium ca. 1.9× longer than tarsomere 3. Aedeagus (Fig. 75b) dorsoventrally flattened, widened to subangulate apex; transfer apparatus short, tubuliform. **Intraspecific variation**. Length 1.78–2.10 mm.

##### Material examined.

Holotype (MZB): ARC0836 (EMBL # HE615518), WEST NEW GUINEA, Manokwari Reg., Arfak Mts, Mokwam, Siyoubrig, S01°07.066', E133°54.710', 1870 m, 11-XII-2007, sifted. Paratypes (SMNK, ZSM): WEST NEW GUINEA, Manokwari Reg., Arfak Mts: 7 exx, ARC0835 (EMBL # HE615517), ARC0837 (EMBL # HE615519), same data as holotype.

##### Distribution.

Manokwari Reg. (Arfak Mts). Elevation: 1870 m.

##### Biology.

Sifted from leaf litter in montane forest.

##### Etymology.

This epithet is composed of the Latin participle *scissus* (split) and the Greek noun *ops* (eye) and refers to the remarkable morphology of the eye of this species.

##### Notes.

*Trigonopterus scissops* Riedel, sp. n. was coded as “*Trigonopterus* sp. 55” by Tänzler et al. (2012).

#### 76. Trigonopterus scharfi

Riedel sp. n.

urn:lsid:zoobank.org:act:8D7C7A25-B2D6-416F-813B-2F9186048CB2

http://species-id.net/wiki/Trigonopterus_scharfi

##### Diagnostic description.

**Figure 76.**
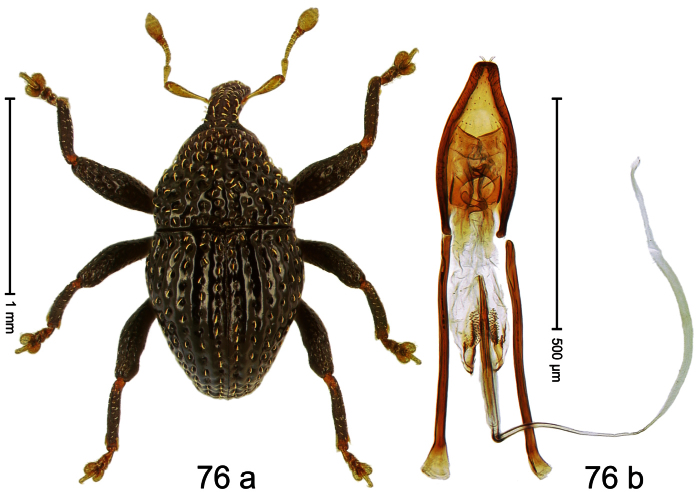
*Trigonopterus scharfi* Riedel, sp. n., holotype; (**a**) Habitus (**b**) Aedeagus.

Holotype, male (Fig. 76a). Length 1.40 mm. Color black; head, antenna, tarsi and tibiae ferruginous. Body subovate; in dorsal aspect and in profile with distinct constriction between pronotum and elytron. Rostrum with indistinct, median ridge, sparsely setose. Eyes in subdorsal position, approximate. Pronotum with weak subapical constriction; disk with median ridge; densely punctate with large punctures; each puncture with curved, suberect, yellowish scale. Elytra with striae deeply impressed; each puncture with curved, suberect, yellowish scale; intervals weakly costate, subglabrous. Meso- and metafemur ventrally dentate. Metafemur subapically with stridulatory patch. Aedeagus (Fig. 76b) with apex extended, pointed, curved ventrad; transfer apparatus spiniform; ductus ejaculatorius with bulbus. **Intraspecific variation**. Length 1.40–1.60 mm. Female rostrum dorsally slightly flattened, in apical half subglabrous, weakly punctate.

##### Material examined.

Holotype (MZB): ARC0536 (EMBL # FN429242), WEST NEW GUINEA, Jayapura Reg., Cyclops Mts, S02°31.594', E140°30.407', 1065 m, 21-XI-2007, sifted. Paratypes (ARC, SMNK, ZSM): WEST NEW GUINEA, Jayapura Reg., Cyclops Mts, Sentani: 2 exx, ARC0535 (EMBL # FN429241), ARC0537 (EMBL # FN429243), same data as holotype; 17 exx, S02°31.383', E140°30.490', 1275 m, 30-XI-2007, sifted; 20 exx, ARC0678 (EMBL # FN429323), ARC0679 (EMBL # FN429324), S02°31.281', E140°30.535', 1420 m, 30-XI-2007, sifted; 67 exx, ARC0684 (EMBL # FN429329), ARC0685 (EMBL # FN429330), S02°31.182', E140°30.542', 1510 m, 30-XI-2007, sifted; 6 exx, S02°31.425', E140°30.474', 1265 m, 30-XI-2007, sifted; 1 ex, S02°31.683', E140°30.281', 960 m, 21-XI-2007, sifted; 14 exx, 1000 m, 23-XII-2004, sifted; 5 exx, S02°31.594', E140°30.407', 1065 m, 21-XI-2007, sifted; 2 exx, Sentani, S02°31.603', E140°30.434', 1095 m, 28-XI-07, sifted; 5 exx, 1100 m, 23-XII-2004, sifted; 6 exx, 1200–1400 m, 09-VIII-1991, sifted; 44 exx, 300–1400 m, 10-VIII-1991, sifted; 2 exx, S02°31.516', E140°30.436', 1150 m, 21-XI-2007, sifted.

##### Distribution.

Jayapura Reg. (Cyclops Mts). Elevation: 960–1510 m.

##### Biology.

Sifted from leaf litter in montane forest.

##### Etymology.

This species is named in honor of Stefan Scharf (Karlsruhe), who has spent months creating a 3D computer model of one of the paratypes.

##### Notes.

*Trigonopterus scharfi* Riedel, sp. n. was coded as “*Trigonopterus* sp. 51” by Riedel et al. (2010) and Tänzler et al. (2012), respectively “*Trigonopterus* spay” in the EMBL/GenBank/DDBJ databases.

#### 77. Trigonopterus signicollis

Riedel sp. n.

urn:lsid:zoobank.org:act:1F97F68E-A68D-40AB-832E-AFB33A98CAE3

http://species-id.net/wiki/Trigonopterus_signicollis

##### Diagnostic description.

**Figure 77.**
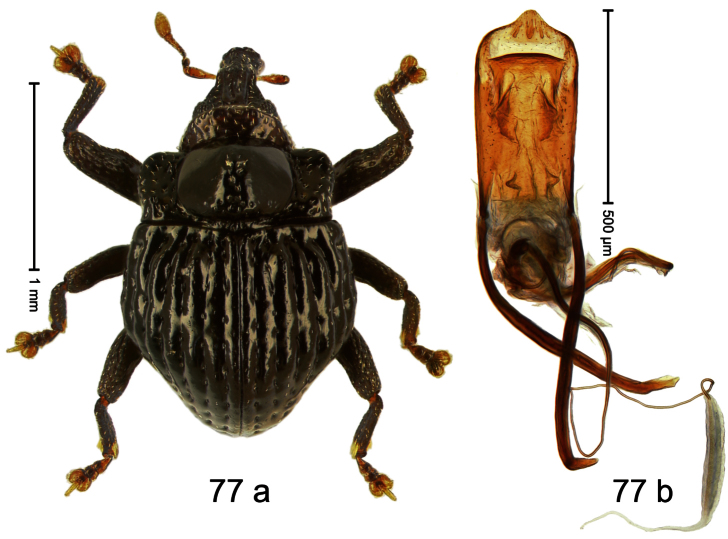
*Trigonopterus signicollis* Riedel, sp. n., holotype; (**a**) Habitus (**b**) Aedeagus.

Holotype, male (Fig. 77a). Length 1.79 mm. Color black; legs and apex of pronotum deep ferruginous; antenna and tarsi light ferruginous. Body subglobose; in dorsal aspect and in profile with distinct constriction between pronotum and elytron. Rostrum in basal half with 3 ridges posteriorly continued to and uniting on forehead; subapically scabrous; epistome forming curved, transverse ridge. Pronotum deeply sculptured; with marked subapical constriction, apical collar and lateral costate flanges punctate, sparsely setose; transversely ovate disk swollen, glabrous except medially with few punctures. Elytra subglabrous, on disk striae deeply incised, intervals costate, towards sides and near apex with deep punctures; intervals 3, 5, and 6 more prominent; apex extended ventrad, beak-shaped; lateral interval subapically with row of setae. Meso- and metafemur weakly dentate. Metafemur with weakly denticulate dorsoposterior edge, subapically without stridulatory patch. Aedeagus (Fig. 77b) with apex medially pointed; body in apical half with broad depression visible in lateral aspect; transfer apparatus markedly asymmetrical, flagelliform, curled, pointing laterad; ductus ejaculatorius with bulbus. **Intraspecific variation**. Length 1.71–2.03 mm. Female rostrum in apical half dorsally flattened, subglabrous, with sparse punctures; epistome simple.

##### Material examined.

Holotype (MZB): ARC0553 (EMBL # FN429259), WEST NEW GUINEA, Jayapura Reg., Cyclops Mts, Sentani, S02°31.383', E140°30.490', 1275 m, 30-XI-2007, sifted. Paratypes (ARC, SMNK, ZSM): WEST NEW GUINEA, Jayapura Reg., Cyclops Mts, Sentani: 2 exx, same data as holotype; 2 exx, ARC0560 (EMBL # FN429266), ARC0561 (EMBL # FN429267), S02°31.425', E140°30.474', 1265 m, 30-XI-2007, sifted; 2 exx, S02°31.281', E140°30.535', 1420 m, 30-XI-2007, sifted; 3 exx, ARC0682 (EMBL # FN429327), ARC0683 (EMBL # FN429328), S02°31.182', E140°30.542', 1510 m, 30-XI-2007, sifted; 1 ex, 1320 m, 23-XII-2004.

##### Distribution.

Jayapura Reg. (Cyclops Mts). Elevation: 1265–1510 m.

##### Biology.

Sifted from leaf litter in montane forest.

##### Etymology.

This epithet is based on a combination of the Latin nouns *signum* (mark, seal) and *collum* (neck, pronotum) and refers to the distinct, central portion of the pronotal disk.

##### Notes.

*Trigonopterus signicollis* Riedel, sp. n. was coded as “*Trigonopterus* sp. 38” by Riedel et al. (2010) and Tänzler et al. (2012), respectively “*Trigonopterus* spal” in the EMBL/GenBank/DDBJ databases.

#### 78. Trigonopterus simulans

Riedel sp. n.

urn:lsid:zoobank.org:act:7F9C0CC2-B228-4134-8548-19243C03D182

http://species-id.net/wiki/Trigonopterus_simulans

##### Diagnostic description.

**Figure 78.**
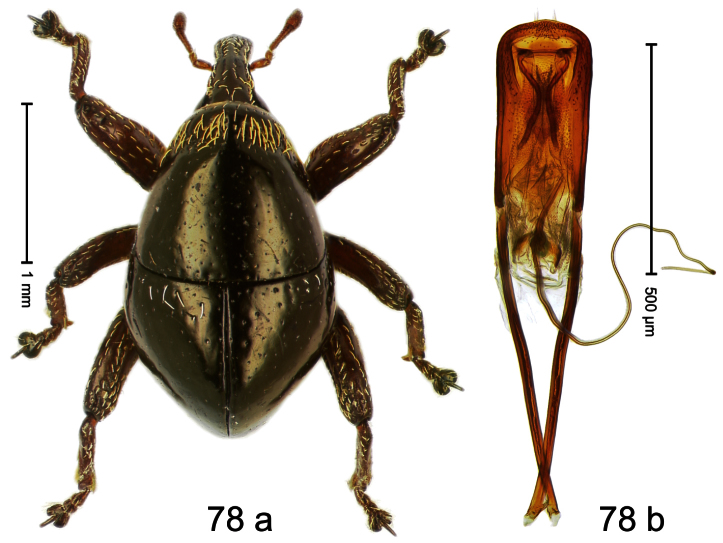
*Trigonopterus simulans* Riedel, sp. n., holotype; (**a**) Habitus (**b**) Aedeagus.

Holotype, male (Fig. 78a). Length 2.15 mm. Color black, with bronze lustre; legs and antenna ferruginous. Body subovate; in dorsal aspect without constriction between pronotum and elytron; in profile with distinct constriction. Rostrum with weak median ridge and pair of submedian furrows. Pronotum anteriorly densely punctate, with yellowish elongate apicad directed scales; disk subglabrous, with sparse minute punctures. Elytra near base and before apex with sparse, yellowish, elongate scales; remainder subglabrous, with sparse minute punctures; striae obsolete. Anteroventral ridges of femora simple. Metafemur with weakly denticulate dorsoposterior edge, subapically without stridulatory patch. Abdominal ventrite 5 swollen, at middle with depression. Aedeagus (Fig. 78b) with apex rounded; behind orifice containing X-shaped pair of sclerites; in profile dorsal contour convex; transfer apparatus spiniform, 0.3× as long as body of aedeagus. **Intraspecific variation**. Length 2.15–2.30 mm. Female rostrum slender, dorsally subglabrous, sparsely punctate.

##### Material examined.

Holotype (MZB): ARC0518 (EMBL # FN429224), WEST NEW GUINEA, Jayapura Reg., Cyclops Mts, Sentani, S02°31.912', E140°30.416', 785 m, 02-XII-2007, sifted. Paratypes (ARC, SMNK, ZSM): WEST NEW GUINEA, Jayapura Reg., Cyclops Mts, Sentani: 2 exx, ARC0511 (EMBL # HE615270), ARC0513 (EMBL # FN429219), S02°32.031', E140°30.412', 710 m, 02-XII-2007, sifted; 1 ex, S02°31.912', E140°30.416', 785 m, 02-XII-2007, sifted; 2 exx, ARC0542 (EMBL # FN HE615301), S02°31.683', E140°30.281', 960 m, 21-XI-2007, sifted.

##### Distribution.

Jayapura Reg. (Cyclops Mts). Elevation: 710–960 m.

##### Biology.

Sifted from leaf litter in primary forest.

##### Etymology.

This epithet is based on the Latin adjective *simulans* (imitating). It refers to the close resemblance of this species with other closely related ones.

##### Notes.

*Trigonopterus simulans* Riedel, sp. n. was coded as “*Trigonopterus* sp. 45” by Riedel et al. (2010) and Tänzler et al. (2012), respectively “*Trigonopterus* spas” in the EMBL/GenBank/DDBJ databases.

#### 79. Trigonopterus soiorum

Riedel sp. n.

urn:lsid:zoobank.org:act:74800A1A-4341-4D75-9D36-3700E71A7DE3

http://species-id.net/wiki/Trigonopterus_soiorum

##### Diagnostic description.

**Figure 79.**
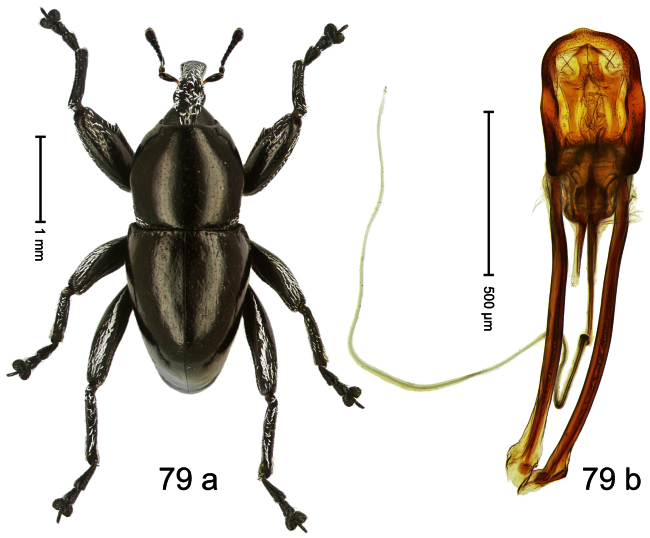
*Trigonopterus soiorum* Riedel, sp. n., holotype; (**a**) Habitus (**b**) Aedeagus.

Holotype, male (Fig. 79a). Length 3.06 mm. Color black; base of antennal scape ferruginous. Body elongate; in dorsal aspect with distinct constriction between pronotum and elytron; in profile with weak constriction. Rostrum slender; dorsally with carina and pair of short submedian ridges; at base with erect white elongate scales, apically replaced by bristles. Eyes large, medially approximate. Pronotum with disk subglabrous, with minute punctures; with distinct edge lateral edge except in apical 1/4 evenly rounded; posterior angles with coarse punctures and sparse white scales; sides along anterior margin with few white scales; above procoxa coarsely punctate. Elytra subglabrous, irregularly punctate with minute punctures; striae obsolete; basal margin straight; laterally behind humeri with ridge bordered by row of shallow punctures. Femora with anteroventral ridge distinct, terminating at base; profemur at middle with tooth. Mesofemur and metafemur dorsally densely squamose with white scales. Metafemur with smooth dorsoposterior edge; subapically without stridulatory patch. Metaventrite and abdominal ventrites 1–2 forming common, subglabrous concavity. Abdominal ventrite 5 with subrotund densely setose impression, laterally bordered by distinct ridges. Aedeagus (Fig. 79b) with body markedly curved in profile; apex subangulate, subglabrous; transfer apparatus short, spiniform; ductus ejaculatorius subapically without bulbus. **Intraspecific variation**. Length 3.06–3.38 mm. Female rostrum in apical 2/3 dorsally subglabrous, sparsely punctate, sublaterally with furrow containing row of setae; at base bordering eyes with white elongate scales. Female abdominal ventrite 5 flat, subglabrous.

##### Material examined.

Holotype (SMNK): ARC1858 (EMBL # HE616135), PAPUA NEW GUINEA, Eastern Highlands Prov., Aiyura, S06°21.033', E145°54.597', 2169 m, 06-II-2010. Paratypes (NAIC, SMNK, ZSM): PAPUA NEW GUINEA, Eastern Highlands Prov.: 1 ex, ARC1857 (EMBL # HE616134), same data as holotype; 5 exx, ARC1859 (EMBL # HE616136), ARC1860 (EMBL # HE616137), Okapa, Kimiagomo village, Verefare, S06°24.760', E145°35.575', 1940 m, 18-III-2010; 2 exx, Okapa, Kofare village, S06°25.212', E145°35.498', 2140 m, 18-III-2010, beaten; 2 exx, Okapa, Anurite village, S06°24.760', E145°35.575', 1940 m, 18-III-2010, beaten; 3 exx, Okapa, Nakaloyate village, S06°24.760', E145°35.575', 1940 m, 18-III-2010.

##### Distribution.

Eastern Highlands Prov. (Aiyura, Okapa). Elevation: 1940–2169 m.

##### Biology.

Beaten from foliage of montane forests.

##### Etymology.

This species is dedicated to the people of Papua New Guinea. The epithet is based on the family name Soi, found on page 330 of the Papua New Guinea Telephone Directory of 2010 and treated in genitive plural.

##### Notes.

*Trigonopterus soiorum* Riedel, sp. n. was coded as “*Trigonopterus* sp. 58” by Tänzler et al. (2012).

#### 80. Trigonopterus sordidus

Riedel sp. n.

urn:lsid:zoobank.org:act:4FC14699-33FD-4559-A77E-4838ED9272D5

http://species-id.net/wiki/Trigonopterus_sordidus

##### Diagnostic description.

**Figure 80.**
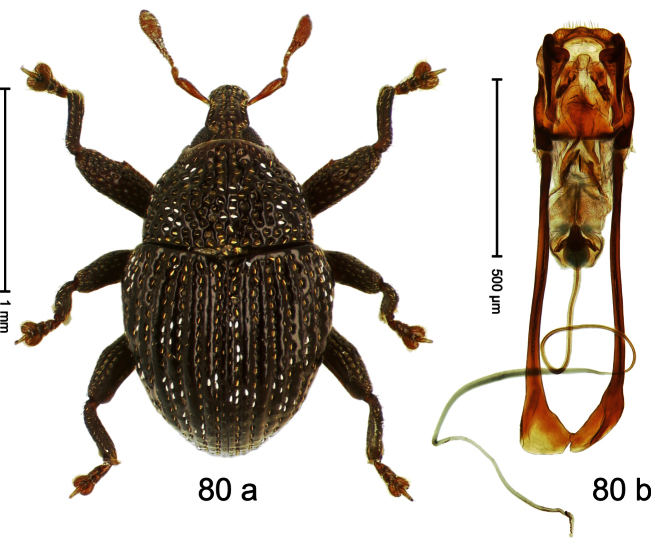
*Trigonopterus sordidus* Riedel, sp. n., holotype; (**a**) Habitus (**b**) Aedeagus.

Holotype, male (Fig. 80a). Length 1.98 mm. Color black; antenna and tarsi light ferruginous; tibiae, femora, and head deep ferruginous. Body subovate; in dorsal aspect with distinct constriction between pronotum and elytron; in profile evenly convex. Rostrum rugose-punctate; epistome weakly, transversely swollen. Pronotum deeply rugose-punctate, each puncture containing one scale; majority of scales narrow, ochre; few scales almond-shaped, white. Elytra with striae deeply impressed; strial punctures with same set of scales as pronotum; intervals costate, largely subglabrous, but with scattered scales. Femora dentate. Metafemur with simple dorsoposterior edge, subapically with stridulatory patch. Aedeagus (Fig. 80b) complex, laterally incised, with dorsolateral well-sclerotized flanges; apex subtruncate, sparsely setose. Endophallus with various sclerites. transfer apparatus drop-shaped, apically pointed; ductus ejaculatorius with weak bulbus. **Intraspecific variation**. Length 1.86–2.28 mm. Color ferruginous in some possibly teneral specimens. Scattered scales on elytra more or less numerous.

##### Material examined.

Holotype (MZB): ARC0736 (EMBL # HE615419), WEST NEW GUINEA, Jayawijaya Reg., Jiwika, Kurulu, S03°57.161', E138°57.357', 1875 m, 24-XI-2007, sifted. Paratypes (ARC, SMNK, ZSM): WEST NEW GUINEA, Jayawijaya Reg., Jiwika, Kurulu: 1 ex, ARC1715 (EMBL # HE615995), S03°57.161', E138°57.357', 1875 m, 12-VII-2010, sifted; 2 exx (1 marked ARC0080), ca. 1700–2300 m, 02-IX-1991, sifted; 9 exx, 1900–2000 m, 23-IX-1992, sifted; 2 exx, 1900–2050 m, 24-X.1993.

##### Distribution.

Jayawijaya Reg. (Jiwika). Elevation: 1875–1900 m.

##### Biology.

Sifted from leaf litter in montane forest.

##### Etymology.

This epithet is based on the Latin adjective *sordidus* (dirty) and refers both to the occurrence of incrustations and the species´ general appearance making it hard to distinguish from a grain of dirt.

##### Notes.

*Trigonopterus sordidus* Riedel, sp. n. was coded as “*Trigonopterus* sp. 176” by Tänzler et al. (2012).

#### 81. Trigonopterus squamirostris

Riedel sp. n.

urn:lsid:zoobank.org:act:909E35FA-AA5F-479E-AF66-B5A9E59317E6

http://species-id.net/wiki/Trigonopterus_squamirostris

##### Diagnostic description.

**Figure 81.**
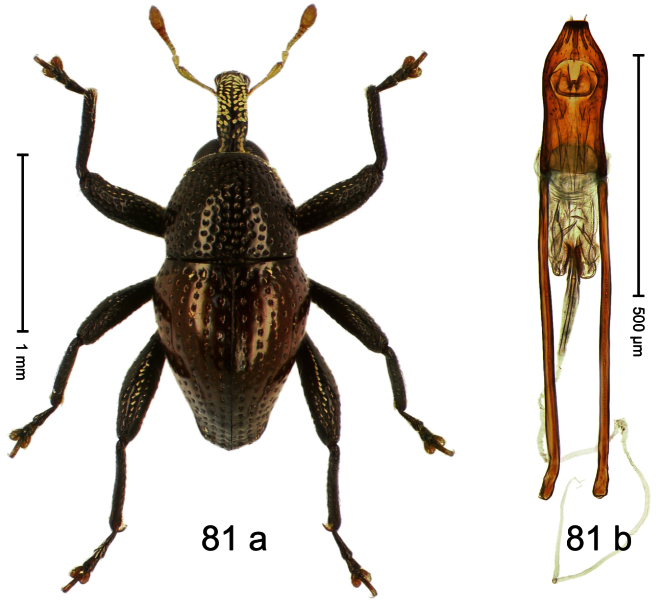
*Trigonopterus squamirostris* Riedel, sp. n., holotype; (**a**) Habitus (**b**) Aedeagus.

Holotype, male (Fig. 81a). Length 1.70 mm. Color black; tarsi and elytra dark ferruginous, antenna light ferruginous. Body subovate; in dorsal aspect and in profile with distinct constriction between pronotum and elytron. Eyes large. Rostrum punctate, surface microreticulate, densely squamose with cream-colored almond-shaped scales, without distinct furrows or ridges. Pronotum densely punctate; anterolaterally with scattered cream-colored scales. Elytra with striae distinct; intervals flat, subglabrous; stria 3 near base and apex with few scales. Femora edentate, dorsally with few scattered scales. Metafemur subapically with stridulatory patch. Metatibia simple; uncus hook-like extended, curved ventroposteriad. Aedeagus (Fig. 81b) with apodemes 2.0 X as long as body; sides of body weakly bisinuate; apex extended, pointed, markedly curved ventrad; transfer apparatus small, symmetrical; ductus ejaculatorius basally swollen, without bulbus. **Intraspecific variation**. Length 1.38–1.88 mm. Color of elytra ferruginous or black. Female rostrum dorsally subglabrous, submedially with row of punctures, sublateral furrow containing sparse row of setae. Female metatibia with uncus simple, not hook-like.

##### Material examined.

Holotype (SMNK): ARC1119 (EMBL # HE615748), PAPUA NEW GUINEA, Simbu Prov., Karimui Dist., Haia, Supa, S06°40.078', E145°03.207' to S06°39.609', E145°03.012', 1220–1450 m, 02-X-2009. Paratypes (NAIC, SMNK, ZSM): Simbu Prov., Karimui Dist., Haia: 1 ex, ARC1104 (EMBL # HE615733), S06°41.018', E145°00.995',1090 m, 04-X-2009, sifted; 28 exx, ARC1113 (EMBL # HE615742), ARC1114 (EMBL # HE615743), ARC1118 (EMBL # HE615747), ARC1120 (EMBL # HE615749), ARC1121 (EMBL # HE615750), same data as holotype; 1 ex, Haia, S06°41.259', E145°00.822' to S06°41.102', E145°00.979', 900–1005 m, 27-IX-2009; 3 exx, Haia, S06°41.216', E145°00.945' to S06°40.976', E145°00.979', 970–1135 m, 04-X-2009, beaten; 3 exx, Haia, Supa station, S06°40.047', E145°03.464' to S06°39.815', E145°03.169', 1075–1240m, 30-IX-2009, beaten; 1 ex, Haia, Supa station, S06°40.047', E145°03.464' to S06°39.905', E145°03.880', 1075–1220 m, 01-X-2009, beaten; 1 ex, Haia, Supa station, S06°39.905', E145°03.880' to S06°39.796', E145°03.873', 1220–1320 m, 01-X-2009, beaten; 3 exx, Haia, Supa station, S06°40.047', E145°03.464' to S06°40.078', E145°03.207', 1075–1220 m, 02-X-2009, beaten.

##### Distribution.

Simbu Prov. (Haia). Elevation: 1005–1220 m.

##### Biology.

Mostly collected by beating foliage in montane forest.

##### Etymology.

This epithet is based on a combination of the Latin nouns *squama* (scale) and rostrum (snout) and refers to the clothed rostrum of the males.

##### Notes.

*Trigonopterus squamirostris* Riedel, sp. n. was coded as “*Trigonopterus* sp. 225” by Tänzler et al. (2012).

#### 82. Trigonopterus striatus

Riedel sp. n.

urn:lsid:zoobank.org:act:BF617D19-62C4-4847-9CFD-99F747C7564D

http://species-id.net/wiki/Trigonopterus_striatus

##### Diagnostic description.

**Figure 82.**
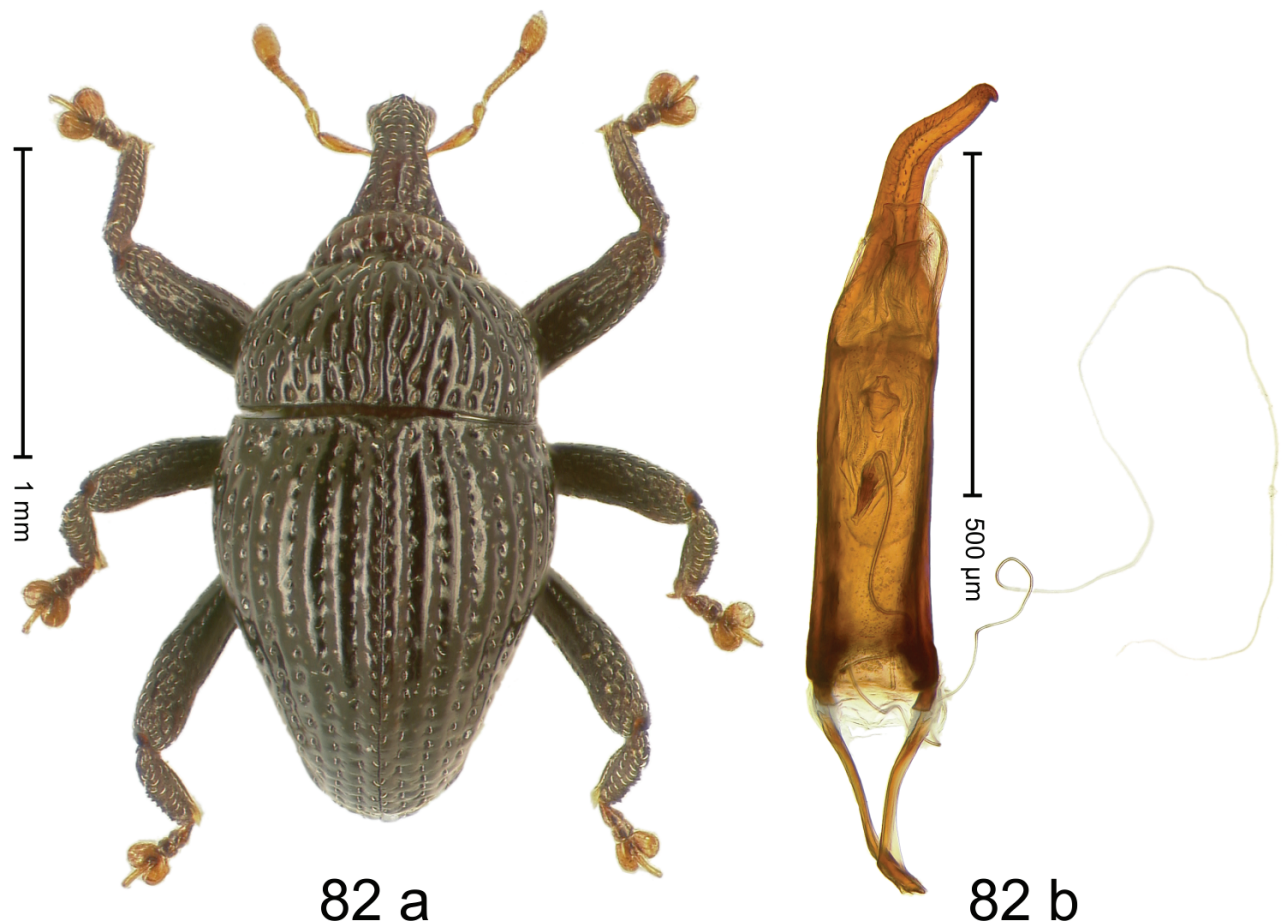
*Trigonopterus striatus* Riedel, sp. n., holotype; (**a**) Habitus (**b**) Aedeagus.

Holotype, male (Fig. 82a). Length 1.92 mm. Color of antenna and tarsi light ferruginous; tibiae, head, and anterior part of pronotum deep ferruginous; remainder black. Body subovate; in dorsal aspect and in profile with distinct constriction between pronotum and elytron. Rostrum rugose-punctate, sparsely setose with recumbent mesad directed setae. Pronotum with distinct subapical constriction; disk longitudinally rugose-punctate; sparsely setose. Elytra with striae deeply impressed; each puncture with inconspicuous seta; intervals weakly costate, subglabrous; sutural interval more distinctly raised; apex extended ventrad, slightly beak-shaped. Meso- and metafemur ventrally weakly dentate. Metafemur with denticulate dorsoposterior edge, subapically without stridulatory patch. Abdominal venter excavated. Abdominal ventrites 4 simple; ventrite 5 flat, ferruginous. Aedeagus (Fig. 82b) with apex extended, bent to the left; transfer apparatus short, spiniform; basal orifice ventrally with rim; apodemes short; ductus ejaculatorius without bulbus. **Intraspecific variation**. Length 1.43–1.96 mm. Female rostrum dorsally subglabrous, sparsely punctate. Abdominal ventrite 3 of females with flattened process projecting over retracted, subglabrous ventrites 4–5; process of ventrite 3 shaped like a flattened thistle-leaf with one long median spine and two shorter spines on each side.

##### Material examined.

Holotype (MZB): ARC0603 (EMBL # HE615329), WEST NEW GUINEA, Manokwari, Mt. Meja, S00°51.497', E134°04.949', 220 m, 05-XII-2007, sifted. Paratypes (ARC, SMNK, ZSM): WEST NEW GUINEA, Manokwari, Mt. Meja: 2 exx, ARC0604 (EMBL # HE615330), ARC0605 (EMBL # HE615331), same data as holotype; 6 exx, S00°51.400', E134°04.918', 225 m, 06-XII-2007, sifted; 13 exx (1 marked as “ARC00141”), 200 m, 30-XII-2004, sifted; 3 exx, 22-23-IX-1990, sifted; 5 exx, 200 m, 19-IV-1993, sifted; 19 exx, 200 m, 30-XII-2000, sifted.

##### Distribution.

Manokwari Reg. (Mt. Meja). Elevation: 200–225 m.

##### Biology.

Sifted from leaf litter in lowland forest.

##### Etymology.

This epithet is based on the Latin participle *striatus* (provided with furrows) and refers to the species´ body-sculpture.

##### Notes.

*Trigonopterus striatus* Riedel, sp. n. was coded as “*Trigonopterus* sp. 256” by Tänzler et al. (2012).

#### 83. Trigonopterus strigatus

Riedel sp. n.

urn:lsid:zoobank.org:act:AA765190-0387-4301-A5EA-4806700C0D1F

http://species-id.net/wiki/Trigonopterus_strigatus

##### Diagnostic description.

**Figure 83.**
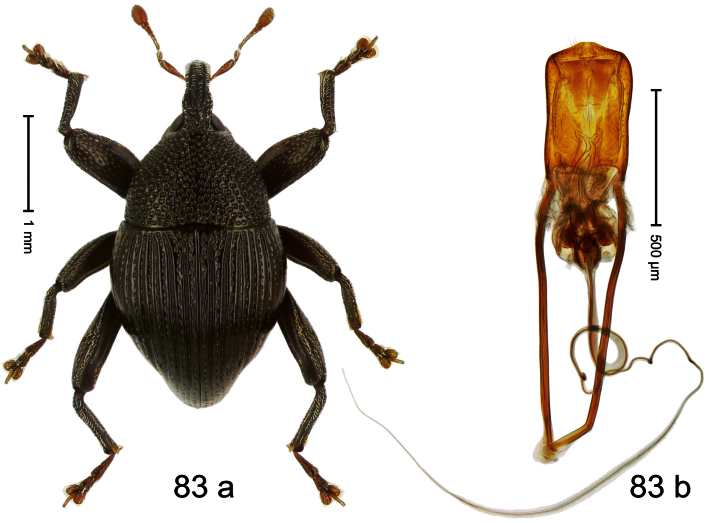
*Trigonopterus strigatus* Riedel, sp. n., holotype; (**a**) Habitus (**b**) Aedeagus.

Holotype, male (Fig. 83a). Length 2.95 mm. Color black; antenna and tarsi ferruginous. Body ovate; in dorsal aspect almost without constriction between pronotum and elytron; in profile evenly convex. Rostrum in basal half with distinct median ridge and pair of submedian ridges, furrows with sparse rows of yellowish scales; apically weakly punctate, sparsely setose. Pronotum densely punctate-reticulate. Elytra dorsally with striae deeply incised forming well-defined furrows; intervals each with one secondary furrow of fused elongate punctures; laterally subglabrous, sparsely punctate. Femora somewhat widened, with distinct anteroventral ridge, edentate. Metafemur dorsally sparsely squamose with silvery scales; with weakly denticulate dorsoposterior edge; subapically with stridulatory patch. Metatibia apically with uncus, without premucro. Abdominal ventrite 5 with shallow depression, densely punctate, with dense suberect setae. Aedeagus (Fig. 83b) apically angulate, subglabrous; complex transfer-apparatus symmetrical; ductus ejaculatorius basally swollen, with indistinct bulbus. **Intraspecific variation**. Length 2.94–3.06 mm. Female rostrum in apical half slender, dorsally subglabrous, punctate, posteriorly with furrows. Female abdominal ventrite 5 densely punctate, with sparse recumbent scales, medially with weak swelling.

##### Material examined.

Holotype (SMNK): ARC1188 (EMBL # HE615816), PAPUA NEW GUINEA, Morobe Prov., Huon peninsula, Mindik, S06°27.380', E147°25.099' to S06°27.267', E147°25.049', 1500–1650 m, 09-X-2009. Paratypes (ARC, NAIC, SMNK, ZSM): Morobe Prov., Huon peninsula, Mindik: 7 exx, ARC1189 (EMBL # HE615817), ARC1190 (EMBL # HE615818), same data as holotype; 4 exx, 1500–1670 m, S06°27.311', E147°24.073' to S06°27.221', E147°24.185', beaten, 10-X-2009; 1 ex (marked as ARC0060), 1200–1500 m, 26-IV-1998; 1 ex, 1400–1550 m, 27-IV-1998.

##### Distribution.

Morobe Prov. (Mindik). Elevation: 1500 m.

##### Biology.

Beaten from foliage of montane forests.

##### Etymology.

This epithet is based on the Latin participle *strigatus* (striated) and refers to the species´ elytral sculpture.

##### Notes.

*Trigonopterus strigatus* Riedel, sp. n. was coded as “*Trigonopterus* sp. 216” by Tänzler et al. (2012).

#### 84. Trigonopterus strombosceroides

Riedel sp. n.

urn:lsid:zoobank.org:act:482BE1A0-47AB-4F78-83B8-ACCFB436F5EA

http://species-id.net/wiki/Trigonopterus_strombosceroides

##### Diagnostic description.

**Figure 84.**
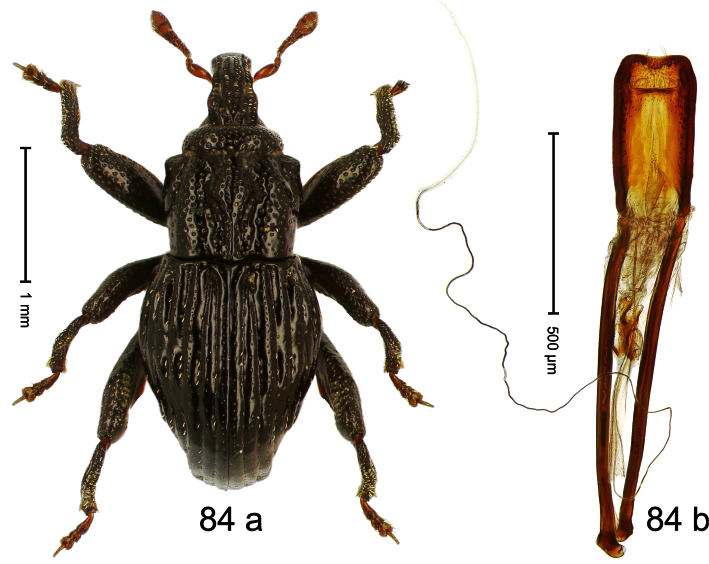
*Trigonopterus strombosceroides* Riedel, sp. n., holotype; (**a**) Habitus (**b**) Aedeagus.

Holotype, male (Fig. 84a). Length 2.75 mm. Color black; tarsi and antenna ferruginous. Body elongate; in dorsal aspect and in profile with distinct constriction between pronotum and elytron. Rostrum with longitudinal ridges and rows of coarse punctures, with sparse suberect scales; epistome flat, with small punctures. Pronotum with marked subapical constriction, anteriorly densely punctate; disk deeply sculptured, with median ridge and pair of broad submedian ridges; ridges densely punctate; behind constriction anteriorly with pair of lateral angular protrusions. Elytra with striae deeply incised; with sparse narrow cream-colored scales; intervals irregularly costate, with rows of small punctures, along two transverse bands depressed; apex rounded. Femora edentate. Metafemur with weakly denticulate dorsoposterior edge, subapically with stridulatory patch. Tarsomere 3 small, hardly larger than preceding, onychium ca. 1.5 X longer than tarsomere 3. Abdominal ventrites 1–2 with common impression; abdominal ventrite 5 concave, tomentose. Aedeagus (Fig. 84b) apically subtruncate, without setae; with asymmetrical transfer apparatus; ductus ejaculatorius without bulbus. Intraspecific variation. Length 2.75–2.93 mm.

##### Material examined.

Holotype (MZB): ARC0546 (EMBL # FN429252), WEST NEW GUINEA, Jayapura Reg., Cyclops Mts, Sentani, S02°31.776', E140°30.215', 945 m, 21-XI-2007, sifted. Paratypes (ARC, SMNK, ZSM): WEST NEW GUINEA, Jayapura Reg., Cyclops Mts, Sentani: 4 exx, ARC0547 (EMBL # FN429253), ARC0548 (EMBL # FN429254), same data as holotype; 1 ex (marked as ARC0090), 1100 m, 23-XII-2004, sifted; 2 exx, S02°31.425', E140°30.474', 1265 m, 30-XI-2007, sifted.

##### Distribution.

Jayapura Reg. (Cyclops Mts). Elevation: 945–1265 m.

##### Biology.

Sifted from leaf litter in primary forest.

##### Etymology.

This epithet is a combination of the name Stromboscerinae and the Latin ending *-oides* (having the form of) and refers to the species´ resemblance in habitus with members of this subfamily of Dryophthoridae.

##### Notes.

*Trigonopterus strombosceroides* Riedel, sp. n. was coded as “*Trigonopterus* sp. 37” by Riedel et al. (2010) and Tänzler et al. (2012), respectively “*Trigonopterus* spak” in the EMBL/GenBank/DDBJ databases.

#### 85. Trigonopterus subglabratus

Riedel sp. n.

urn:lsid:zoobank.org:act:44E2D57D-14C1-4E38-84E8-B8D39449ED0A

http://species-id.net/wiki/Trigonopterus_subglabratus

##### Diagnostic description.

**Figure 85.**
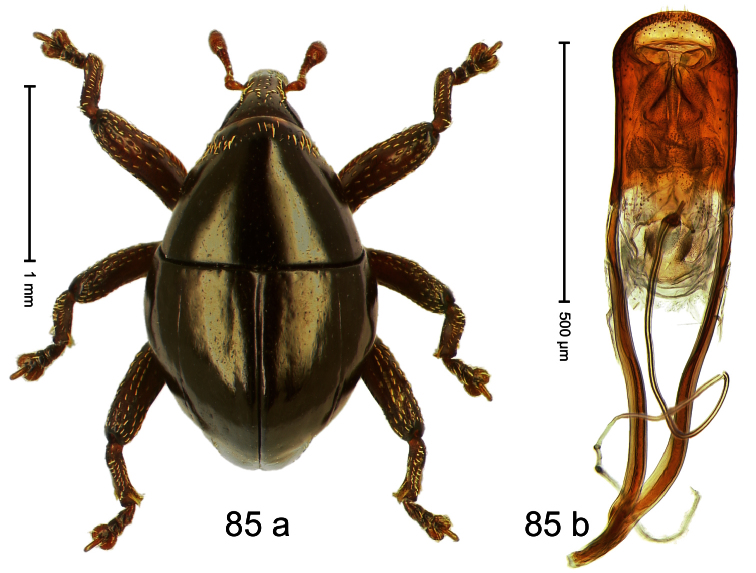
*Trigonopterus subglabratus* Riedel, sp. n., holotype; (**a**) Habitus (**b**) Aedeagus.

Holotype, male (Fig. 85a). Length 2.09 mm. Color ferruginous, pronotum and elytra black with bronze lustre. Body subovate; without constriction between pronotum and elytron; in profile evenly convex. Rostrum with weak median ridge, pair of submedian furrows, sparsely squamose. Pronotum anteriorly sparsely punctate with small punctures, with yellowish ovate to elongate scales; disk subglabrous. Elytra subglabrous, striae obsolete except stria 4 and suture weakly incised; subapically laterally sparsely squamose. Anteroventral ridges of femora simple. Metafemur with dorsoposterior edge simple, subapically without stridulatory patch. Metaventrite and abdominal ventrites 1–2 forming common concavity. Abdominal ventrite 5 markedly swollen, subapically with shallow impression. Aedeagus (Fig. 85b) with apex rounded; in profile dorsal contour forming right angle; transfer apparatus very short, spiniform. **Intraspecific variation**. Length 2.09–2.48 mm. Female rostrum in apical half sparsely punctate with minute punctures. Female metaventrite and abdominal ventrites 1–2 less markedly concave.

##### Material examined.

Holotype (MZB): ARC0638 (EMBL # FN429291), WEST NEW GUINEA, Jayapura Reg., Cyclops Mts, Doyo, S02°32.478', E140°28.835', 365 m, 27-XI-2007, sifted. Paratypes (ARC, SMNK, ZSM): WEST NEW GUINEA, Jayapura Reg., Cyclops Mts, Sentani: 1 ex, ARC0514 (EMBL # FN429220), S02°32.031', E140°30.412', 710 m, 02-XII-2007, sifted; 2 exx, ARC0633 (EMBL # FN429288), ARC0634 (EMBL # FN429289), Sentani, S02°32.221', E140°30.526', 575 m, 19-XI-2007, sifted.

##### Distribution.

Jayapura Reg. (Cyclops Mts). Elevation: 365–710 m.

##### Biology.

Sifted from leaf litter in primary forest.

##### Etymology.

This epithet is based on a combination of the Latin prefix *sub*- (less than; almost) and the participle *glabratus* (smoothened). It refers to its smooth body surface.

##### Notes.

*Trigonopterus subglabratus* Riedel, sp. n. was coded as “*Trigonopterus* sp. 47” by Riedel et al. (2010) and Tänzler et al. (2012), respectively “*Trigonopterus* spat” in the EMBL/GenBank/DDBJ databases.

#### 86. Trigonopterus sulcatus

Riedel sp. n.

urn:lsid:zoobank.org:act:E795D8B6-C12D-4300-A91F-1D0977604471

http://species-id.net/wiki/Trigonopterus_sulcatus

##### Diagnostic description.

**Figure 86.**
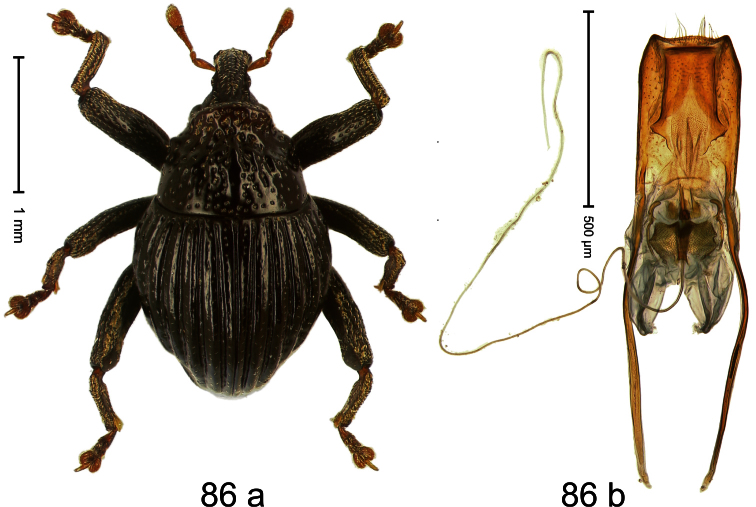
*Trigonopterus sulcatus* Riedel, sp. n., holotype; (**a**) Habitus (**b**) Aedeagus.

Holotype, male (Fig. 86a). Length 2.24 mm. Color black; legs and apex of pronotum deep ferruginous; antenna light ferruginous. Body subovate-globose; with distinct constriction between pronotum and elytron; in profile evenly convex. Rostrum in basal half medially weakly carinate, coarsely punctate, sparsely setose; epistome with transverse, angulate ridge. Pronotum with subapical constriction, laterally with pair of blunt denticles; disk coarsely punctate, weakly rugose. Elytra with striae deeply incised, towards sides with deep interspersed punctures; intervals costate, subglabrous, with sparse small punctures and minute recumbent setae; apex rounded. Femora edentate. Metafemur with weakly denticulate dorsoposterior edge, subapically with stridulatory patch. Abdominal ventrite 2 laterally projecting dentiform over elytral edge in profile; abdominal ventrites 1–2 with common cavity; abdominal ventrite 5 sublaterally with pair of denticles. Aedeagus (Fig. 86b) apically sparsely setose; with complex, symmetrical transfer apparatus; ductus ejaculatorius without bulbus. **Intraspecific variation**. Length 2.23–2.64 mm.

##### Material examined.

Holotype (MZB): ARC0632 (EMBL # FN429287), WEST NEW GUINEA, Jayapura Reg., Cyclops Mts, Sentani, S02°32.221', E140°30.526', 575 m, 19-XI-2007, sifted. Paratypes (SMNK, ZSM): WEST NEW GUINEA, Jayapura Reg., Cyclops Mts, Sentani: 2 exx, ARC0508 (EMBL # FN429214), ARC0509 (EMBL # FN429215), S02°32.031', E140°30.412', 710 m, 02-XII-2007, sifted.

##### Distribution.

Jayapura Reg. (Cyclops Mts). Elevation: 575–710 m.

##### Biology.

Sifted from leaf litter in lowland forest.

##### Etymology.

This epithet is based on the Latin participle *sulcatus* (furrowed, grooved) and refers to the elytral sculpture.

##### Notes.

*Trigonopterus sulcatus* Riedel, sp. n. was coded as “*Trigonopterus* sp. 33” by Riedel et al. (2010) and Tänzler et al. (2012), respectively “*Trigonopterus* spag” in the EMBL/GenBank/DDBJ databases.

#### 87. Trigonopterus taenzleri

Riedel sp. n.

urn:lsid:zoobank.org:act:C31E3CFE-9696-480E-ABF1-6AD099FEBC87

http://species-id.net/wiki/Trigonopterus_taenzleri

##### Diagnostic description.

**Figure 87.**
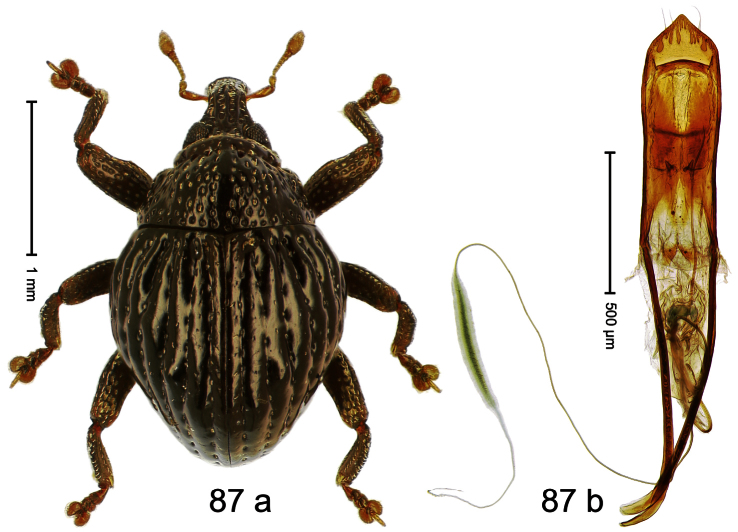
*Trigonopterus taenzleri* Riedel, sp. n., holotype; (**a**) Habitus (**b**) Aedeagus.

Holotype, male (Fig. 87a). Length 2.16 mm. Color black; head and legs deep ferruginous; antenna light ferruginous. Body subglobose; in dorsal aspect and in profile with distinct constriction between pronotum and elytron. Rostrum in basal half with 3 ridges posteriorly continued to and uniting on forehead; subapically scabrous; epistome forming indistinct transverse ridge. Pronotum deeply punctate except medially glabrous; with distinct subapical constriction. Elytra subglabrous, striae deeply impressed, intervals with irregular knobs and costae; apex extended ventrad, beak-shaped. Femora weakly dentate. Metafemur with denticulate dorsoposterior edge, subapically without stridulatory patch. Aedeagus (Fig. 87b) with apex medially pointed; body in apical half with broad depression visible in lateral aspect; laterally with rows of setae; transfer apparatus markedly flagelliform, longer than body, curled, pointing apicad; ductus ejaculatorius with bulbus. **Intraspecific variation**. Length 2.16–2.43 mm. Female rostrum in apical half dorsally flattened, subglabrous, with sparse punctures and lateral furrows; epistome simple.

##### Material examined.

Holotype (MZB): ARC0551 (EMBL # FN429257), WEST NEW GUINEA, Jayapura Reg., Cyclops Mts, Sentani, S02°31.603', E140°30.434', 1095 m, 28-XI-2007, sifted. Paratypes (SMNK, ZSM): WEST NEW GUINEA, Jayapura Reg., Cyclops Mts, Sentani: 1 ex, S02°31.383', E140°30.490', 1275 m, 30-XI-2007, sifted; 2 exx, ARC0552 (EMBL # FN429258), same data as holotype; 1 ex, ARC0541 (EMBL # FN429247), S02°31.594', E140°30.407', 1065 m, 21-XI-2007, sifted; 1 ex, 1000 m, 23-XII-2004, sifted.

##### Distribution.

Jayapura Reg. (Cyclops Mts). Elevation: 1000–1275 m.

##### Biology.

Sifted from leaf litter in montane forest.

##### Etymology.

This species is named in honor of Rene Tänzler (Munich), who has spent years working on *Trigonopterus* weevils.

##### Notes.

*Trigonopterus taenzleri* Riedel, sp. n. was coded as “*Trigonopterus* sp. 39” by Riedel et al. (2010) and Tänzler et al. (2012), respectively “*Trigonopterus* spam” in the EMBL/GenBank/DDBJ databases.

#### 88. Trigonopterus talpa

Riedel sp. n.

urn:lsid:zoobank.org:act:6CDBE834-7B01-4548-B9B9-277B74341B30

http://species-id.net/wiki/Trigonopterus_talpa

##### Diagnostic description.

**Figure 88.**
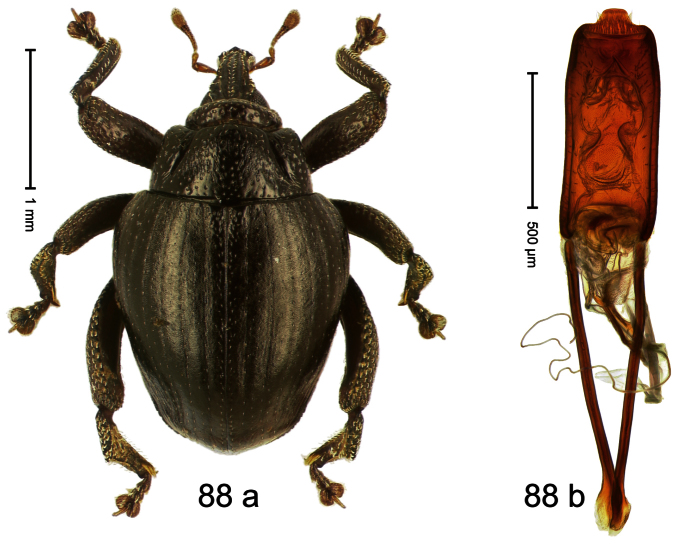
*Trigonopterus talpa* Riedel, sp. n., holotype; (**a**) Habitus (**b**) Aedeagus.

Holotype, male (Fig. 88a). Length 2.63 mm. Color black; legs dark ferruginous; antenna light ferruginous. Body subrhomboid; with marked constriction between pronotum and elytron; in profile evenly convex to subapical constriction of pronotum. Rostrum with pair of sublateral furrows converging posteriorly on forehead; pair of submedian furrows shallow; epistome forming angulate ridge and median denticle. Pronotum with marked subapical constriction, sides weakly converging, behind subapical constriction with angular protrusions; disk with pair of deep furrows curving anteriad joining subapical constriction; center of disk coriaceous, sparsely punctate; laterally rugose. Elytra subovate, apically subangulate; striae weakly impressed; surface dull, microgranulate, almost nude, with sparse minute setae; interval 7 subapically forming distinct, weakly denticulate ridge; apex extended ventrad, slightly beak-shaped in profile. Femora edentate. Meso- and metatibia in basal half widened, subapically narrowed. Metafemur with denticulate dorsoposterior edge, subapically without stridulatory patch. Aedeagus (Fig. 88b) with body parallel-sided; apex medially extended into subtruncate tip; transfer apparatus thick spiniform; ductus ejaculatorius without bulbus. **Intraspecific variation**. Length 2.30–2.63 mm. Female rostrum in apical half medially subglabrous, with two rows of punctures; epistome simple.

##### Material examined.

Holotype (MZB): ARC1686 (EMBL # HE615973), WEST NEW GUINEA, Jayapura Reg., Cyclops Mts, Angkasa indah, S02°30.346', E140°42.087', 490 m, 28-VI-2010, sifted. Paratype (SMNK): 1 ex, ARC1687 (EMBL # HE615974), same data as holotype.

##### Distribution.

Jayapura Reg. (Cyclops Mts). Elevation: 490 m.

##### Biology.

Sifted from leaf litter in primary forest.

##### Etymology.

This epithet is based on the Latin noun *talpa* (mole) in apposition and refers both to the species´ habitus and its edaphic habits.

##### Notes.

*Trigonopterus talpa* Riedel, sp. n. was coded as “*Trigonopterus* sp. 77” by Tänzler et al. (2012).

#### 89. Trigonopterus taurekaorum

Riedel sp. n.

urn:lsid:zoobank.org:act:AAE8DBE8-E025-4A63-9B6A-26D78755ACEF

http://species-id.net/wiki/Trigonopterus_taurekaorum

##### Diagnostic description.

**Figure 89.**
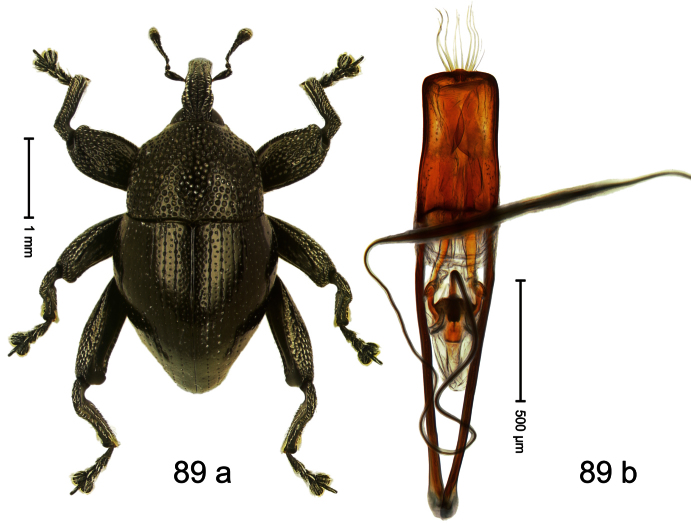
*Trigonopterus taurekaorum* Riedel, sp. n., holotype; (**a**) Habitus (**b**) Aedeagus.

Holotype, male (Fig. 89a). Length 3.44 mm. Color black. Body subovate; with distinct constriction between pronotum and elytron; in profile evenly convex. Rostrum dorsally rugose-punctate, basally with median ridge and indistinct pair of submedian ridges, furrows with sparse rows of setae and yellowish scales. Pronotum densely punctate. Elytra densely punctate; striae distinct, consisting of small punctures; intervals with row of minute punctures; laterally behind humeri with ridge bordered by dense row of deep punctures of stria 9. Femora edentate. Mesofemur and metafemur dorsally densely squamose with silvery scales. Metafemur with denticulate dorsoposterior edge; subapically with stridulatory patch. Metatibia apically with uncus and small premucro. Abdominal ventrite 5 with shallow impression, densely punctate, with sparse erect setae. Aedeagus (Fig. 89b) apically pointed, with tuft of long setae; body containing pair of elongate sclerites; transfer-apparatus symmetrical, with lyriform sclerite; ductus ejaculatorius with bulbus. **Intraspecific variation**. Length 2.91–3.58 mm. Body of females more slender than males. Female rostrum in apical 2/3 slender, dorsally subglabrous, with submedian rows of small punctures and lateral furrows. Pronotum of females basally at middle with punctures smaller and sparser, in males punctures evenly deep and dense. Female abdominal ventrite 5 flat, without erect setae.

##### Material examined.

Holotype (SMNK): ARC1151 (EMBL # HE615779), PAPUA NEW GUINEA, Simbu Prov., Karimui Dist., Haia, Supa, S06°39.905', E145°03.880' to S06°39.796', E145°03.873', 1220–1320 m, 01-X-2009. Paratypes (NAIC, SMNK, ZSM): PAPUA NEW GUINEA, Simbu Prov.: 6 exx, ARC1152 (EMBL # HE615780), same data as holotype; 4 exx, Haia, Supa station, S06°40.047', E145°03.464' to S06°39.815', E145°03.169', 1075–1240 m, 30-IX-2009, beaten; 2 exx, Haia, Supa station, S06°39.815', E145°03.169' to S06°39.609', E145°03.012', 1240–1450 m, 30-IX-2009, beaten; 16 exx, ARC1129 (EMBL # HE615758), ARC1130 (EMBL # HE615759), Haia, Supa, S06°40.078', E145°03.207' to S06°39.609', E145°03.012', 1220–1450 m, 02-X-2009; 20 exx, ARC1167 (EMBL # HE615795), Haia, S06°41.216', E145°00.945' to S06°40.976', E145°00.979', 970–1135 m, 04-X-2009; 14 exx, Haia, S06°43.515', E145°00.128' to S06°43.948', E144°59.856', 750–915 m, 26-IX-2009; 7 exx, ARC1169 (EMBL # HE615797), Haia, S06°41.259', E145°00.822' to S06°41.102', E145°00.979', 900–1005 m, 27-IX-2009; 1 ex, Simbu Prov., Karimui Dist., Haia, S06°41.102', E145°00.979', 1005–1020 m, 27-IX-2009, beaten, “Mimikry-sample”; 2 exx, Haia, S06°41.216', E145°00.945', 965 m, 27-IX-2009, beaten; 3 exx, ARC1082 (EMBL # HE615713), Haia, S06°41.553', E145°00.355' to S06°41.624', E145°00.728', 800–960 m, 25-IX-2009.

##### Distribution.

Simbu Prov. (Haia). Elevation: 915–1240 m.

##### Biology.

Collected by beating foliage in primary forest.

##### Etymology.

This species is dedicated to the people of Papua New Guinea. The epithet is based on the family name Taureka, found on page 338 of the Papua New Guinea Telephone Directory of 2010 and treated in genitive plural.

##### Notes.

*Trigonopterus taurekaorum* Riedel, sp. n. was coded as “*Trigonopterus* sp. 76” by Tänzler et al. (2012).

#### 90. Trigonopterus tialeorum

Riedel sp. n.

urn:lsid:zoobank.org:act:C3792D70-1DA1-4462-8739-AA4888E64087

http://species-id.net/wiki/Trigonopterus_tialeorum

##### Diagnostic description.

**Figure 90.**
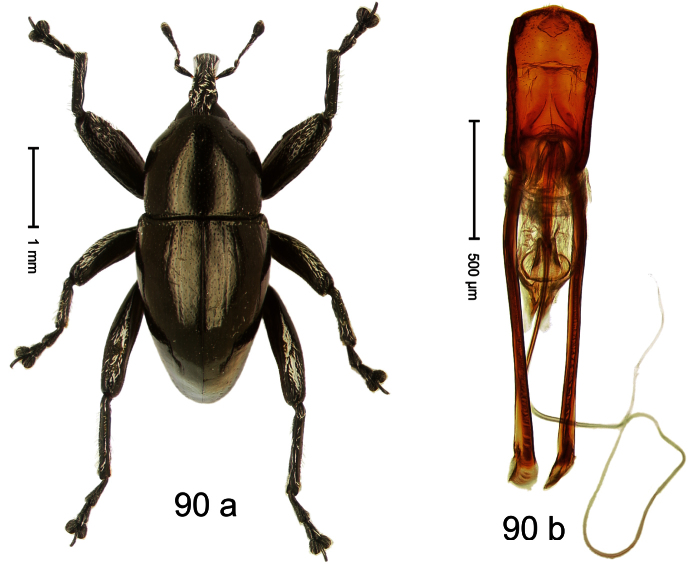
*Trigonopterus tialeorum* Riedel, sp. n., holotype; (**a**) Habitus (**b**) Aedeagus.

Holotype, male (Fig. 90a). Length 3.59 mm. Color black; base of antennal scape ferruginous. Body subovate; in dorsal aspect with weak constriction between pronotum and elytron; in profile with distinct constriction. Rostrum slender; dorsally with carina and pair of submedian ridges; at base with erect white elongate scales, apically replaced by bristles. Eyes large, medially approximate. Pronotum with disk subglabrous, with minute punctures; basal ¼ in front of elytral humeri with indistinct edge bordered by row of deep punctures; anteriorly evenly rounded towards sides; sides anteriorly with white recumbent scales. Elytra subglabrous, irregularly punctate with minute punctures; striae obsolete; basal margin straight; laterally behind humeri with ridge bordered by row of deep punctures. Femora with anteroventral ridge distinct, terminating at base; profemur at middle with tooth. Mesofemur and metafemur dorsally densely squamose with white scales. Metafemur with smooth dorsoposterior edge; subapically without stridulatory patch. Abdominal ventrite 5 hardly impressed at middle, sparsely setose with suberect setae, laterally sparsely squamose. Aedeagus (Fig. 90b) with sides of body dorsally carinate; apex flattened, subangulate; transfer apparatus flagelliform, subequal to body, relatively thick; ductus ejaculatorius subapically without bulbus. **Intraspecific variation**. Length 3.59–3.64 mm. Female rostrum dorsally subglabrous, sparsely punctate. Abdominal ventrite 5 flat, subglabrous, sparsely punctate.

##### Material examined.

Holotype (SMNK): ARC1829 (EMBL # HE616106), PAPUA NEW GUINEA, Eastern Highlands Prov., Okapa, Kimiagomo village, Verefare, S06°24.760', E145°35.575', 1940 m, 18-III-2010. Paratypes (NAIC, SMNK, ZSM): PAPUA NEW GUINEA, Eastern Highlands Prov.: 37 exx, same data as holotype; 22 exx, ARC1824 (EMBL # HE616101), ARC1825 (EMBL # HE616102), Okapa, Kimiagomo village, Afiyaleto, S06°25.593', E145°34.862', S06°25.212', E145°35.498', 1911 m, 18-III-2010; 36 exx, Okapa, Nakaloyate village, S06°24.760', E145°35.575', 1940 m, 18-III-2010; 8 exx, Okapa, Kofare village, S06°25.212', E145°35.498', 2140 m, 18-III-2010, beaten; 7 exx, Okapa, Anurite village, S06°24.760', E145°35.575', 1940 m, 18-III-2010, beaten; 10 exx, Okapa, Verefare village, S06°24.760', E145°35.575', 1940 m, 18-III-2010, beaten; 4 exx, Okapa, Afiyaleto village, S06°25.593', E145°34.862', 1940 m, 18-III-2010, beaten; 3 exx, Okapa, Afiyaleto village, S06°25.593', E145°34.862' to S06°25.212', E145°35.498', 1911 m, 18-III-2010, beaten; 4 exx, Okapa, Mayakumate village, 2100 m, 18-III-2010, beaten; 3 exx, Okapa, Isimomo, S06°25.003', E145°34.527', 2131 m, 18-III-2010; 3 exx, Okapa, Hamegoya, S06°25.727', E145°35.455' to S06°25.117', E145°35.225', 1891–2131 m, 18-III-2010; 7 Ex, ARC1826 (EMBL # HE616103), ARC1827 (EMBL # HE616104), ARC1828 (EMBL # HE616105), Aiyura, S06°21.033', E145°54.597', 2169 m, 06-II-2010; 1 ex, ARC1057 (EMBL # HE615688), Goroka, Mt. Gahavisuka, S06°00.864', E145°24.779', 2150–2250 m, 24-X-2009.

##### Distribution.

Eastern Highlands Prov. (Aiyura, Okapa). Elevation: 1911–2169 m.

##### Biology.

Beaten from foliage of montane forests.

##### Etymology.

This species is dedicated to the people of Papua New Guinea. The epithet is based on the family name Tiale, found on page 341 of the Papua New Guinea Telephone Directory of 2010 and treated in genitive plural.

##### Notes.

*Trigonopterus tialeorum* Riedel, sp. n. was coded as “*Trigonopterus* sp. 59” by Tänzler et al. (2012).

#### 91. Trigonopterus tibialis

Riedel sp. n.

urn:lsid:zoobank.org:act:6ABBA0D1-8197-4BFA-9C26-A4F7B3A4732C

http://species-id.net/wiki/Trigonopterus_tibialis

##### Diagnostic description.

**Figure 91.**
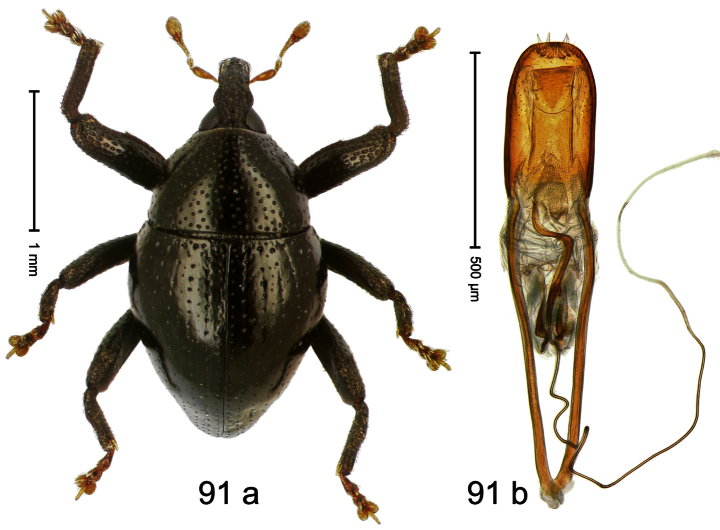
*Trigonopterus tibialis* Riedel, sp. n., holotype; (**a**) Habitus (**b**) Aedeagus.

Holotype, male (Fig. 91a). Length 2.25 mm. Color black; legs deep ferruginous; antenna and tarsi light ferruginous. Body subovate; with weak constriction between pronotum and elytron; in profile evenly convex. Rostrum dorsally rugose-punctate, at middle with low median ridge and pair of submedian ridges. Eyes large. Pronotum punctate; interstices usually larger than diameter of punctures. Elytra punctate with small punctures; striae weakly impressed; intervals with minute punctures; lateral stria behind humeri with dense row of deep punctures. Femora with small tooth at middle. Metafemur dorsoposteriorly punctate-denticulate; subapically with stridulatory patch. Tibiae dorsally carinate, with rows of dark scales partly projecting over dorsal edge. Abdominal ventrite 5 flat, densely punctate. Aedeagus (Fig. 91b) with sides converging in apical half; apex rounded, sparsely setose; transfer-apparatus asymmetrical, spiniform, curved somewhat S-shaped, subequal to body of aedeagus; ductus ejaculatorius without bulbus. **Intraspecific variation**. Length 1.86–2.58 mm. Female rostrum dorsally without distinct ridges, with rows of punctures.

##### Material examined.

Holotype (MZB): ARC0428 (EMBL # FN429139), WEST NEW GUINEA, Jayapura Reg., Cyclops Mts, Sentani, S02°31.2', E140°30.5', 1420–1520 m, 30-XI-2007, beaten. Paratypes (ARC, SMNK, ZSM): WEST NEW GUINEA, Jayapura Reg., Cyclops Mts, Sentani: 15 exx, ARC0429 (EMBL # FN429140), ARC0430 (EMBL # FN429141), same data as holotype; 16 exx, S02°31.3', E140°30.5', 1200–1420 m, 30-XI-2007; 2 exx, S02°31.281', E140°30.535', 1420 m, 30-XI-2007; 1 ex, 1100–1600 m, 05-X-1991; 2 exx, 950–1450 m, 03-X-1992; 2 exx, 300–1400 m, 10-VIII-1991; 5 exx (1 marked as “ARC00410”) 1100–1600 m, 05-X-1991; 1 ex, 1200–1400 m, 09-VIII-1992.

##### Distribution.

Jayapura Reg. (Cyclops Mts). Elevation: 1400–1420 m.

##### Biology.

Collected by beating foliage in montane forests.

##### Etymology.

This epithet is based on the Latin noun *tibia* (shinbone) and refers to the species´ diagnostic tibial morphology.

##### Notes.

*Trigonopterus tibialis* Riedel, sp. n. was coded as “*Trigonopterus* sp. 16” by Riedel et al. (2010) and Tänzler et al. (2012), respectively “*Trigonopterus* spp” in the EMBL/GenBank/DDBJ databases.

#### 92. Trigonopterus tridentatus

Riedel sp. n.

urn:lsid:zoobank.org:act:CE40CDC8-9F6A-4CD5-9499-DAB3D4A4A365

http://species-id.net/wiki/Trigonopterus_tridentatus

##### Diagnostic description.

**Figure 92.**
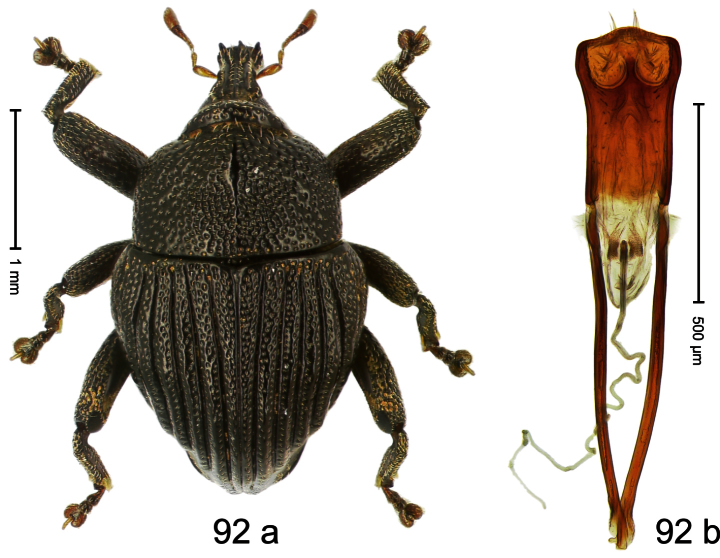
*Trigonopterus tridentatus* Riedel, sp. n., holotype; (**a**) Habitus (**b**) Aedeagus.

Holotype, male (Fig. 92a). Length 2.90 mm. Color black; antenna light ferruginous, legs dark ferruginous. Body subovate, with marked constriction between pronotum and elytron; in profile almost evenly convex. Rostrum in basal half with median carina continued to forehead; with pair of shorter submedian costae; at middle with anteriorly hollowed, heart-shaped protuberance; between protuberance and epistome relatively flat, with sparse erect setae; epistome with transverse, angulate ridge provided with three teeth, median tooth shorter than lateral ones. Pronotum with subapical constriction; disk densely punctate-rugose, lower parts microreticulate, median line subglabrous; laterally above procoxa with cavity. Elytra with striae deeply incised, intervals costate, densely punctate. Femora edentate. Metafemur with denticulate dorsoposterior edge, subapically with stridulatory patch. Abdominal ventrite 2 projecting dentiform over elytral edge in profile. Aedeagus (Fig. 92b) before angulate apex widened; transfer apparatus small, spiniform; ductus ejaculatorius without bulbus. **Intraspecific variation**. Length 2.14–2.90 mm. Female rostrum dorsally with low median costa bearing double-row of punctures, with lateral pair of furrows; epistome simple.

##### Material examined.

Holotype (MZB): ARC0765 (EMBL # HE615448), WEST NEW GUINEA, Manokwari, Mt. Meja, S00°51.497', E134°04.949', 220 m, 05-XII-2007, sifted. Paratypes (ARC, SMNK, ZSM): WEST NEW GUINEA, Manokwari, Mt. Meja: 2 exx, ARC0606 (EMBL # HE615332), ARC0607 (EMBL # HE615333), same data as holotype; 11 exx, S00°51.400', E134°04.918', 225 m, 06-XII-2007, sifted; 1 ex, 22-23-IX-1990, sifted; 9 exx, 200 m, 19-IV-1993, sifted; 2 exx, 200 m, 30-XII-2000, sifted; 8 exx, 200 m, 30-XII-2004, sifted.

##### Distribution.

Manokwari Reg. (Mt. Meja). Elevation: 200–225 m.

##### Biology.

Sifted from leaf litter in lowland forest.

**Etymology.** This epithet is composed of the Latin prefix *tri*- (three) and the participle *dentatus* (toothed) and refers to the three apical teeth of the male rostrum.

##### Notes.

*Trigonopterus tridentatus* Riedel, sp. n. was coded as “*Trigonopterus* sp. 265” by Tänzler et al. (2012).

#### 93. Trigonopterus uniformis

Riedel sp. n.

urn:lsid:zoobank.org:act:AD26DAEC-6157-4ECA-9B7D-2B6E4968C677

http://species-id.net/wiki/Trigonopterus_uniformis

##### Diagnostic description.

**Figure 93.**
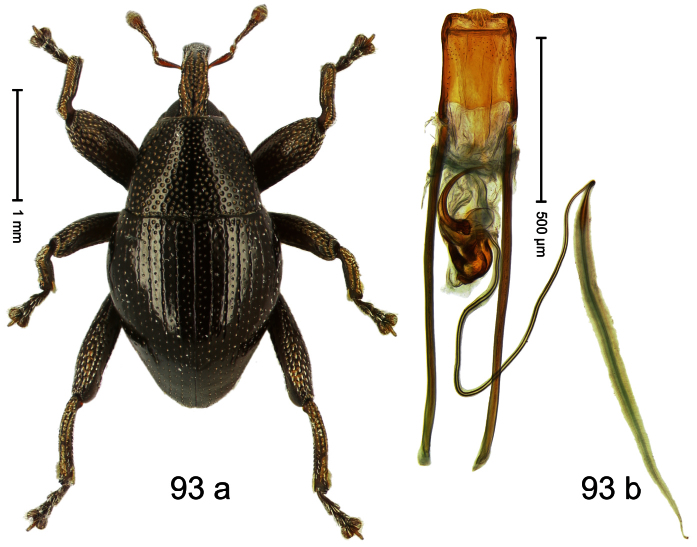
*Trigonopterus uniformis* Riedel, sp. n., holotype; (**a**) Habitus (**b**) Aedeagus.

Holotype, male (Fig. 93a). Length 2.68 mm. Color black; legs deep ferruginous, antenna light ferruginous. Body ovate, with weak constriction between pronotum and elytron; in profile almost evenly convex. Rostrum dorsally in basal half rugose-punctate, in apical half punctate. Pronotum densely punctate except along partly impunctate midline. Elytra densely punctate with small punctures; striae impressed as fine lines; intervals with confused punctures; lateral stria behind humeri with row of deep punctures. Femora in apical 1/3 with anteroventral ridge terminating as tooth. Metafemur with denticulate dorsoposterior edge; subapically with stridulatory patch. Metatibia apically with uncus, premucro, and supra-uncal tooth. Aedeagus (Fig. 93b) medially weakly extended; transfer-apparatus asymmetrical, spiniform, curved; endophallus basally with shell-shaped sclerite; ductus ejaculatorius with bulbus. **Intraspecific variation**. Length 2.50–2.78 mm. Female rostrum dorsally subglabrous, sparsely punctate, basally sparsely squamose.

##### Material examined.

Holotype (MZB): ARC0769 (EMBL # HE615452), WEST NEW GUINEA, Manokwari, Mt. Meja, S00°51.497', E134°04.949', 220 m, 05-XII-2007. Paratypes (ARC, SMNK, ZSM): WEST NEW GUINEA, Manokwari, Mt. Meja: 2 exx, ARC0220 (EMBL # HE615157), ARC0221 (EMBL # HE615158), 200 m, 31-I-2006; 1 exx, S00°51.400', E134°04.918', 225 m, 06-XII-2007; 2 exx, ARC0770 (EMBL # HE615453), ARC0771 (EMBL # HE615454), same data as holotype; 4 exx, 200 m, 30-XII-2000 – 01-I-2001; 3 exx, 22-23-IX-1990; 7 exx, 200 m, 24-VIII-1991; 1 ex, 200 m, 18-III-1993.

##### Distribution.

Manokwari Reg. (Mt. Meja). Elevation: 200–225 m.

##### Biology.

Beaten from foliage of lowland forest.

##### Etymology.

This epithet is based on the Latin adjective *uniformis* (uniform) and refers its average habitus which is similar to hundreds of other *Trigonopterus* species.

##### Notes.

*Trigonopterus uniformis* Riedel, sp. n. was coded as “*Trigonopterus* sp. 118” by Tänzler et al. (2012).

#### 94. Trigonopterus variabilis

Riedel sp. n.

urn:lsid:zoobank.org:act:87F7E821-34C5-44F3-B2D2-C4A7F2DDBABD

http://species-id.net/wiki/Trigonopterus_variabilis

##### Diagnostic description.

**Figure 94.**
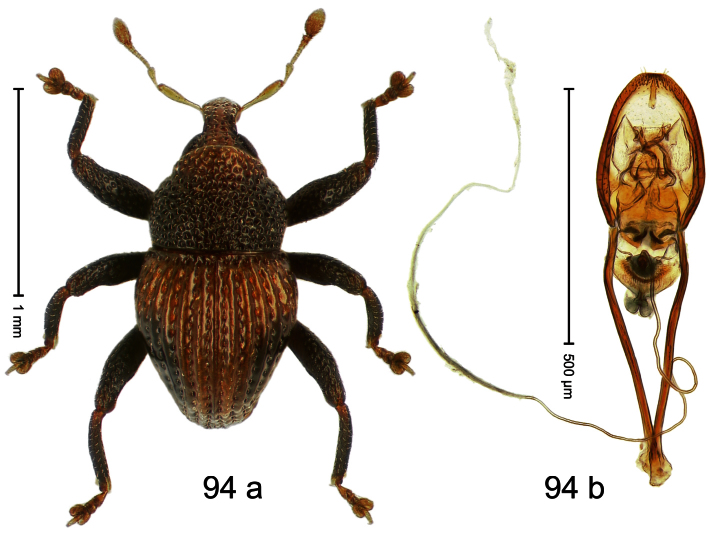
*Trigonopterus variabilis* Riedel, sp. n., holotype; (**a**) Habitus (**b**) Aedeagus.

Holotype, male (Fig. 94a). Length 1.38 mm. Color of head ferruginous; legs and pronotum largely black; elytra ferruginous, in apical half each side with one black spot. Body subovate; in dorsal aspect and in profile with distinct constriction between pronotum and elytron. Eyes large. Rostrum punctate, sparsely setose, without distinct longitudinal ridges; antennal insertion in apical 1/3, anteriorly rostrum with shallow constriction; epistome simple. Pronotum densely punctate-reticulate; each puncture containing one inconspicuous seta. Elytra with striae deeply impressed, intervals costate, subglabrous. Meso- and metafemur ventrally with acute tooth. Tibial uncus simple, curved. Metafemur subapically with indistinct stridulatory patch. Aedeagus (Fig. 94b) with body flattened, almond-shaped, its central portion rather hyaline; transfer apparatus dentiform, encased by capsule stained blue by chlorazol black; ductus ejaculatorius without bulbus. **Intraspecific variation**. Length 1.19–1.66 mm. Color ranging from ferruginous with more or less extensive black pattern to completely black. Female rostrum dorsally subglabrous, punctate, basally with lateral furrows containing sparse row of setae; antennal insertion of females near middle of rostrum.

##### Material examined.

Holotype (SMNK): ARC1096 (EMBL # HE615725), PAPUA NEW GUINEA, Simbu Prov., Karimui Dist., Haia, S06°40.976', E145°00.979', 1135 m, 27-IX-2009, sifted. Paratypes (NAIC, SMNK, ZSM): PAPUA NEW GUINEA, Simbu Prov.: 6 exx, ARC1097 (EMBL # HE615726), ARC1102 (EMBL # HE615731), ARC1103 (EMBL # HE615732), same data as holotype; 13 exx, Haia, S06°40.976', E145°00.979', 1135 m, 04-X-2009, sifted; 7 exx, Haia, S06°41.018', E145°00.995', 1090 m, 04-X-2009, sifted.

##### Distribution.

Simbu Prov. (Haia). Elevation: 1090–1135 m.

##### Biology.

Sifted from leaf litter in primary forest.

##### Etymology.

This epithet is based on the Latin adjective *variabilis* (variable) and refers to the coloration which differs considerably among specimens.

##### Notes.

*Trigonopterus variabilis* Riedel, sp. n. was coded as “*Trigonopterus* sp. 162” by Tänzler et al. (2012).

#### 95. Trigonopterus velaris

Riedel sp. n.

urn:lsid:zoobank.org:act:FB3401F8-39E4-48C8-ABC0-557EDB3C373A

http://species-id.net/wiki/Trigonopterus_velaris

##### Diagnostic description.

**Figure 95.**
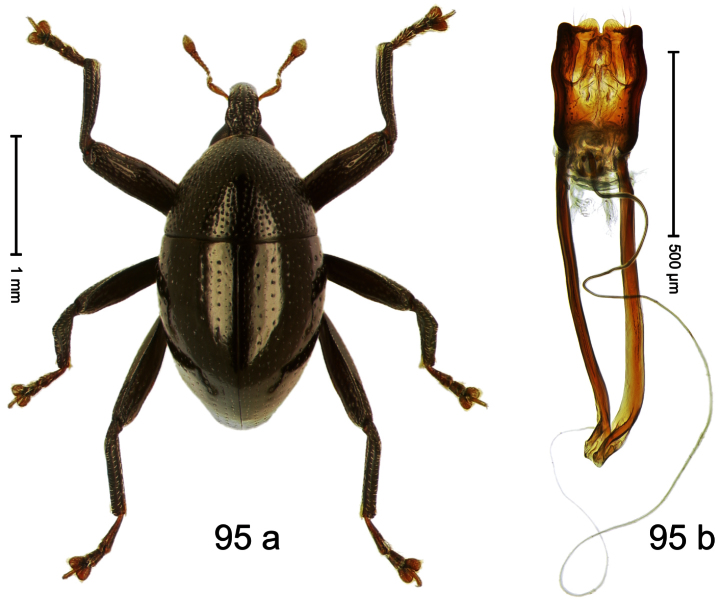
*Trigonopterus velaris* Riedel, sp. n., holotype; (**a**) Habitus (**b**) Aedeagus.

Holotype, male (Fig. 95a). Length 2.45 mm. Color black; legs and rostrum deep ferruginous; antenna light ferruginous. Body laterally somewhat compressed, ovate, without constriction between pronotum and elytron; in profile evenly convex. Rostrum dorsally in basal third with low median ridge and pair of submedian ridges; apically subglabrous. Eyes large. Pronotum densely punctate-rugose, punctures dorsally small, laterally becoming larger, bearing each one minute seta; without scales. Elytra with striae distinct, punctures of stria 1–2 small, laterad strial punctures becoming larger, relatively shallow. Femora with anteroventral ridge. Profemur converging from base to apex. Meso- and metafemur with dorsoposterior edge subapically worn; metafemur subapically without stridulatory patch. Tibiae simple, without rows or brushes of long setae. Metatibia subapically with small suprauncal projection. Metaventrite laterally forming acute process over metacoxa, reaching tibial insertion. Metaventrite and abdominal ventrite 1 subglabrous, with sparse recumbent setae. Abdominal ventrite 2 similar to ventrites 3-4. Abdominal ventrite 5 with deep, transversely ovate cavity almost filling complete ventrite; anterior edge of cavity distinct, swollen. Aedeagus (Fig. 95b) apically sinuate, with deep narrow median incision; ductus ejaculatorius without bulbus. **Intraspecific variation**. Length 2.31–2.59 mm. Female rostrum subglabrous except in basal ¼ with ridges. Female abdominal ventrite 5 flat.

##### Material examined.

Holotype (SMNK): ARC0963 (EMBL # HE615596), PAPUA NEW GUINEA, Central Prov., Moroka area, Kailaki, Mt. Berogoro, S09°24.213', E147°33.870' to S09°23.647', E147°34.244', 500–600 m, 20-IX-2009, beaten. Paratypes (SMNK, NAIC, ZSM): PAPUA NEW GUINEA: 26 exx, ARC0962 (EMBL # HE615595), same data as holotype; 10 exx, Moroka area, Kailaki, Mt. Berogoro, S09°24.213', E147°33.870' to S09°23.647', E147°34.244', 500–565 m, 26-X-2009; 25 exx, Moroka area, Kailaki, Wariaga, S09°25.350', E147°31.047' to S09°25.683', E147°31.707', 650–920 m, 27-X-2009; 5 exx, ARC1079 (EMBL # HE615710), ARC1080 (EMBL # HE615711), Simbu Prov., Karimui Dist., Haia, S06°41.553', E145°00.355' to S06°41.624', E145°00.728', 800–960 m, 25-IX-2009; 2 exx, ARC1165 (EMBL # HE615793), Simbu Prov., Karimui Dist., Haia, S06°41.216', E145°00.945' to S06°40.976', E145°00.979', 970–1135 m, 04-X-2009; 1 ex, Haia, S06°40.976', E145°00.979', 1135 m, sifted, 04-X-2009.

##### Distribution.

Central Prov. (Moroka), Simbu Prov. (Haia). Elevation: 600–1135 m.

##### Biology.

Collected by beating foliage in primary forests.

##### Etymology.

This epithet is based on the Latin adjective *velaris* (concealed) and refers to its morphological similarity with sibling species.

##### Notes.

*Trigonopterus velaris* Riedel, sp. n. was coded as “*Trigonopterus* sp. 272” by Tänzler et al. (2012). It is closely related to *Trigonopterus granum* sp. n., *Trigonopterus pseudogranum* sp. n., and *Trigonopterus imitatus* sp. n. from which it can be distinguished by the denser punctation of the pronotum and the structure of the male abdominal ventrite 5. Despite its close morphological similarity its cox1-sequence diverges 12.1–13.1 % from the other species.

#### 96. Trigonopterus verrucosus

Riedel sp. n.

urn:lsid:zoobank.org:act:CDC03F92-0FAC-4C78-AABA-B8CBF3992E2B

http://species-id.net/wiki/Trigonopterus_verrucosus

##### Diagnostic description.

**Figure 96.**
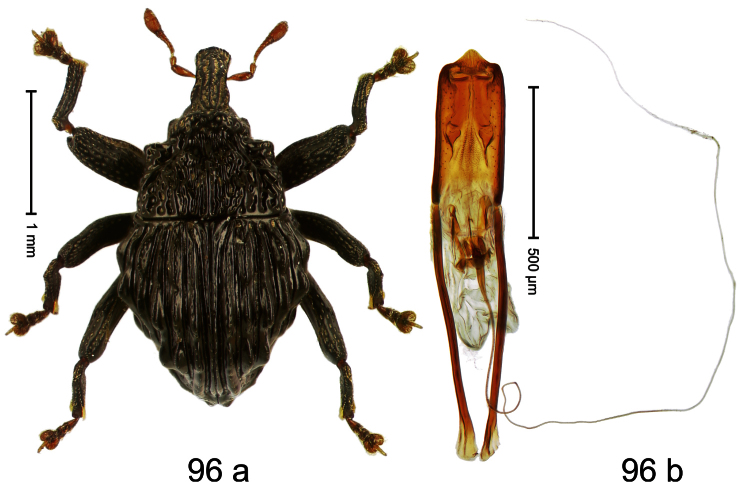
*Trigonopterus verrucosus* Riedel, sp. n., holotype; (**a**) Habitus (**b**) Aedeagus.

Holotype, male (Fig. 96a). Length 2.56 mm. Color black, antenna and tarsi ferruginous. Body subovate, extremely uneven, in dorsal aspect with distinct constriction between pronotum and elytron; in profile without such constriction. Rostrum in basal half medially carinate, coarsely punctate, sparsely setose; epistome with transverse, angulate ridge. Pronotum with marked subapical constriction; laterally projecting with marked angular protrusions; disk longitudinally costate-tuberculate, in basal half medially with prominent ridge. Elytra with striae deeply incised; intervals undulating-costate, some areas markedly elevated, constricted along 2 transverse lines, subglabrous, with sparse minute punctures and setae; apex extended ventrad, slightly beak-shaped, ventrally truncate. Metafemur with dorsoposterior edge simple, in apical third with transverse row of small suberect setae, subapically with stridulatory patch. Disk of abdominal ventrites 1–2 excavated; ventrite 2 projecting dentiform over elytral edge in profile. Aedeagus (Fig. 96b) with apex medially pointed; transfer apparatus complex, symmetrical; ductus ejaculatorius without bulbus. **Intraspecific variation**. Length 2.09–2.56 mm. Female rostrum dorsally with median costa in basal half; epistome simple.

##### Material examined.

Holotype (MZB): ARC0743 (EMBL # HE615426), WEST NEW GUINEA, Jayawijaya Reg., Jiwika, Kurulu, S03°57.161', E138°57.357', 1875 m, 24-XI-2007, sifted. Paratypes (ARC, SMNK): WEST NEW GUINEA, Jayawijaya Reg. Jiwika, Kurulu: 1 ex (marked as ARC0612), 1900–2000 m, 23-IX-1992, sifted; 1 ex, ca. 1700–2300 m, 02-IX-1991, sifted.

##### Distribution.

Jayawijaya Reg. (Jiwika). Elevation: 1875–1900 m.

##### Biology.

Sifted from leaf litter in montane forest.

##### Etymology.

This epithet is based on the Latin adjective *verrucosus* (full of warts) and refers to the species´ remarkable body-sculpture.

##### Notes.

*Trigonopterus verrucosus* Riedel, sp. n. was coded as “*Trigonopterus* sp. 196” by Tänzler et al. (2012).

#### 97. Trigonopterus violaceus

Riedel sp. n.

urn:lsid:zoobank.org:act:154511E2-E70E-4D78-BFBB-EA95A730ACC0

http://species-id.net/wiki/Trigonopterus_violaceus

##### Diagnostic description.

**Figure 97.**
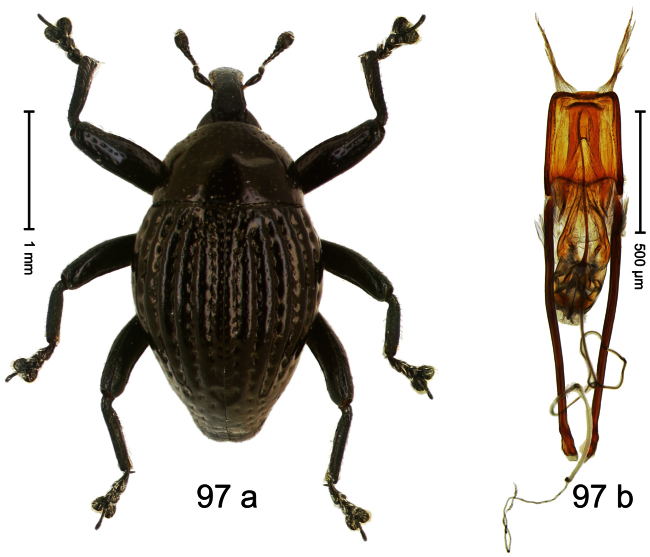
*Trigonopterus violaceus* Riedel, sp. n., holotype; (**a**) Habitus (**b**) Aedeagus.

Holotype, male (Fig. 97a). Length 2.63 mm. Color black, with violet lustre. Body subovate; with weak constriction between pronotum and elytron; in profile evenly convex. Rostrum dorsally relatively flat, sparsely punctate, dorsolaterally with pair of furrows continuing along eye. Pronotum sparsely punctate with minute punctures, subapically punctures larger and denser. Elytra nude, striae deeply impressed with coarse punctures; intervals costate, with sparse minute punctures; intervals 1–3 near base with few larger punctures and coriaceous. Femora edentate. Metafemur subapically with stridulatory patch. Abdominal and thoracic venter with dense erect setae. Aedeagus (Fig. 97b) apically subangulate, with two brushes of long setae; transfer apparatus complex, with flagellum ca. 1.5× as long as body; ductus ejaculatorius without bulbus. **Intraspecific variation**. Length 2.20–2.95 mm. Color ranging from greenish-blue to dark violet. Female rostrum dorsally subglabrous, with punctures smaller than in males. Elytral intervals more or less costate, rarely almost flat.

##### Material examined.

Holotype (MZB): ARC1813 (EMBL # HE616090), WEST NEW GUINEA, Jayawijaya Reg., W Wamena, road to Lake Habbema, S04°07.625', E138°49.992', 2520 m, 20-VII-2010. Paratypes (ARC, SMNK, ZSM): WEST NEW GUINEA, Jayawijaya Reg.: 8 exx, ARC1812 (EMBL # HE616089), ARC1814 (EMBL # HE616091), same data as holotype; 1 ex, ARC1775 (EMBL # HE616052), Poga, S03°47.575', E138°33.155' to S03°47.473', E138°33.163', 2620–2715 m, 15-VII-2010; 4 exx, Ilugwa, Melanggama, trail to Pass valley, 2100–2300 m, 09-10-IX-1990; 2 exx, Ilugwa, trail to Pass valley, 1900–2500 m, 14-IX-1990; 4exx, Kurima, Yohosim – Kiroma, 2500–2700 m, 13-IX-1991.

##### Distribution.

Jayawijaya Reg. (Poga, Ilugwa, L. Habbema, Kurima). Elevation: 2300–2620 m.

##### Biology.

Beaten from foliage of upper montane forests.

##### Etymology.

This epithet is based on the Latin adjective *violaceus* (violet-colored) and refers to the coloration of the species.

##### Notes.

*Trigonopterus violaceus* Riedel, sp. n. was coded as “*Trigonopterus* sp. 123” by Tänzler et al. (2012).

#### 98. Trigonopterus viridescens

Riedel sp. n.

urn:lsid:zoobank.org:act:884E60CB-8362-44DC-9FF1-FF0834E054B9

http://species-id.net/wiki/Trigonopterus_viridescens

##### Diagnostic description.

**Figure 98.**
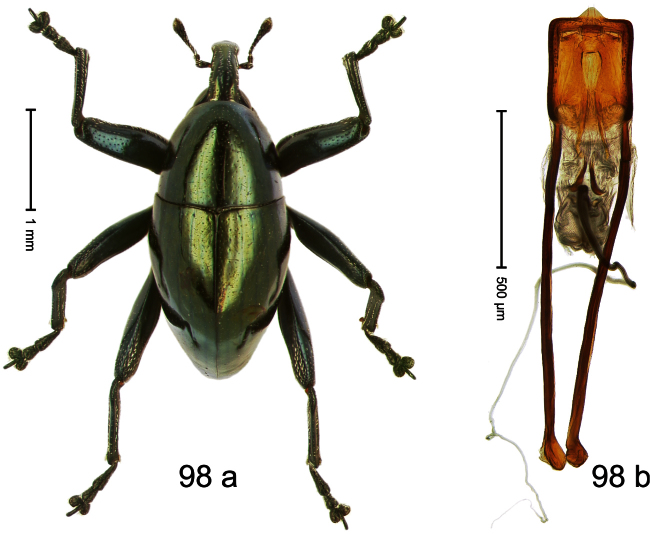
*Trigonopterus viridescens* Riedel, sp. n., holotype; (**a**) Habitus (**b**) Aedeagus.

Holotype, male (Fig. 98a). Length 2.86 mm. Color black with marked greenish lustre. Body slender, ovate; without constriction between pronotum and elytron; in profile evenly convex. Rostrum weakly sculptured, dorsally in basal half with pair of shallow sublateral furrows, with sparse rows of mesad-directed scales, sparsely punctate. Forehead laterally with pair of cavities bordering eyes. Eyes with dorsal margin carinate. Pronotum subglabrous, sparsely punctate with minute punctures. Elytra subglabrous, striae hardly visible. Femora subglabrous, sparsely punctate and squamose, without teeth. Metafemur with smooth dorsoposterior edge; subapically without stridulatory patch. Mesotibia subapically with premucro larger than uncus. Metatibia with premucro somewhat smaller than uncus. Aedeagus (Fig. 98b). Apex symmetrical, with median, pointed extension; transfer apparatus spiniform, apically bordered by pair of curved sclerites; ductus ejaculatorius without bulbus. **Intraspecific variation**. Length 2.48–3.05 mm. Female meso- and metatibia subapically with minute premucro.

##### Material examined.

Holotype (MZB): ARC0434 (EMBL # FN429145), WEST NEW GUINEA, Jayapura Reg., Cyclops Mts, Sentani, S02°31.2', E140°30.5', 1420–1520 m, 30-XI-2007, beaten. Paratypes (ARC, SMNK, ZSM): WEST NEW GUINEA, Jayapura Reg., Cyclops Mts, Sentani: 2 exx, ARC0433 (EMBL # FN429144), ARC0435 (EMBL # FN429146), same data as holotype; 1 ex, 1100–1600 m, 05-X-1991; 5 exx, ARC0671 (PCR failed), ARC0672 (PCR failed), S02°31.3', E140°30.5', 1200–1420 m, 30-XI-2007.

##### Distribution.

Jayapura Reg. (Cyclops Mts). Elevation: 1420–1520 m.

##### Biology.

Collected by beating foliage in montane crippled forests.

##### Etymology.

This epithet is based on the Latin participle *viridescens* (greenish) and refers to the species´ coloration.

##### Notes.

*Trigonopterus viridescens* Riedel, sp. n. was coded as “*Trigonopterus* sp. 24” by Riedel et al. (2010) and Tänzler et al. (2012), respectively “*Trigonopterus* spx” in the EMBL/GenBank/DDBJ databases.

#### 99. Trigonopterus wamenaensis

Riedel sp. n.

urn:lsid:zoobank.org:act:8ACAA7C2-667F-47CE-BC45-1002891F195B

http://species-id.net/wiki/Trigonopterus_wamenaensis

##### Diagnostic description.

**Figure 99.**
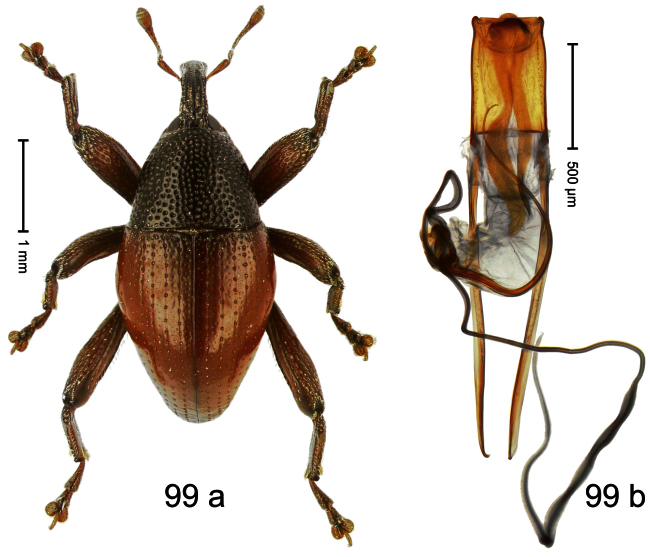
*Trigonopterus wamenaensis* Riedel, sp. n., holotype; (**a**) Habitus (**b**) Aedeagus.

Holotype, male (Fig. 99a). Length 3.17 mm. Color orange-ferruginous; dorsal surface of head and pronotum black. Body subrhomboid, almost without constriction between pronotum and elytron; in profile evenly convex. Rostrum with distinct median and pair of submedian carinae, furrows containing sparse rows of mesad-directed scales. Pronotum densely punctate. Elytra with striae 1–6 distinct, marked by dense rows of small punctures, intervals with minute punctures; lateral striae obsolete; sides with sparse, confused punctures. Femora edentate; anteroventral ridge terminating at middle with inconspicuous denticle. Metafemur with crenulate dorsoposterior edge; subapically with stridulatory patch. Protibia with relatively long uncus. Metatibia apically with uncus and premucro, in apical 1/3 dorsal edge with 4–5 large setiferous punctures. Ventrite 5 densely punctate, medially with broad impression. Aedeagus (Fig. 99b) with apex medially pointed; ostium with intensely-sclerotized asymmetrical sclerite and connected to it with pair of two elongate undulating sclerites reaching far into endophallus; flagelliform transfer-apparatus longer than body of aedeagus; ductus ejaculatorius with bulbus. **Intraspecific variation**. Length 2.72–3.28 mm. Female rostrum dorsally in apical half subglabrous, with submedian rows of punctures. Impression of female abdominal ventrite 5 less marked.

##### Material examined.

Holotype (MZB): ARC0744 (EMBL # HE615427), WEST NEW GUINEA, Jayawijaya Reg., Jiwika, Kurulu, S03°57.161', E138°57.357', 1875 m, 24-XI-2007. Paratypes (ARC, NHMB, NKME, SMNK, ZSM): WEST NEW GUINEA, Jayawijaya Reg. Jiwika, Kurulu: 1 ex, ARC1713 (EMBL # HE615993), S03°57.161', E138°57.357' to S03°56.977', E138°57.441', 1875–1990 m, 12-VII-2010; 1 ex, ARC1735 (EMBL # HE616012), S03°57.161', E138°57.357', 1875 m, 11-VII-2010; 4 exx, Jiwika, ca. 1700–2300 m, 02-IX-1991; 12 exx, Jiwika, 1700–2300 m, 11-IX-1991; 1 ex (marked as ARC0035), Jiwika, 1800–2300 m, 31-V-1998; 7 exx, Wamena, 1600 m, 31-VIII-1990; 6 exx, Jiwika, 1750–2100 m, 05-VII-1994; 3 exx, Jiwika, 1700–2100 m, 05-XII-1995; 1 ex, Ibele river vall., S04°02.283', E138°50.533', 1600 m, 25-I-1999; 504 exx, Baliem-vall., ca. 1700 m, III-1992.

##### Distribution.

Jayawijaya Reg. (Jiwika, Wamena, Ibele vall.). Elevation: 1600–1875 m.

##### Biology.

Beaten from foliage.

##### Etymology.

This epithet is based on the name of Wamena, the main town of the Balim-valley.

##### Notes.

*Trigonopterus wamenaensis* Riedel, sp. n. was coded as “*Trigonopterus* sp. 179” by Tänzler et al. (2012).

#### 100. Trigonopterus wariorum

Riedel sp. n.

urn:lsid:zoobank.org:act:B9671ED6-13B0-43CC-9D42-FE6CECFB07AB

http://species-id.net/wiki/Trigonopterus_wariorum

##### Diagnostic description.

**Figure 100.**
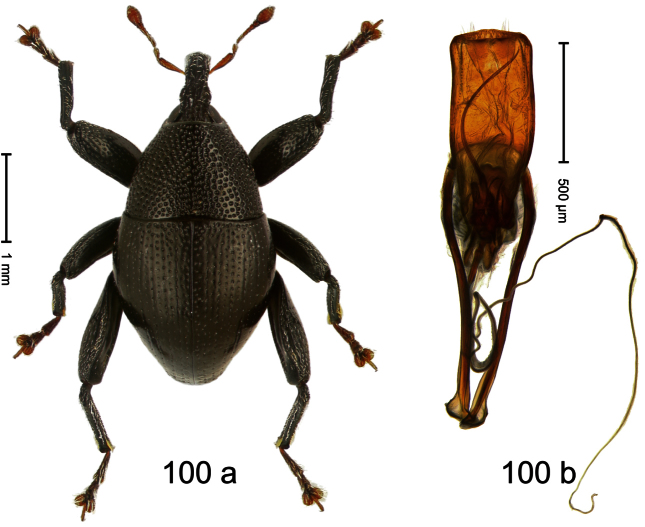
*Trigonopterus wariorum* Riedel, sp. n., holotype; (**a**) Habitus (**b**) Aedeagus.

Holotype, male (Fig. 100a). Length 3.05 mm. Color black; antenna and tarsi ferruginous. Body ovate; in dorsal aspect almost without constriction between pronotum and elytron; in profile evenly convex. Rostrum in basal half with distinct median ridge and pair of submedian ridges, furrows with sparse rows of yellowish scales; apically weakly punctate, sparsely setose. Pronotum densely punctate-reticulate. Elytra densely punctate; striae distinct, consisting of small punctures; intervals with row of smaller punctures; near base and suture with additional, larger, interspersed punctures; surface dull, microgranulate, almost nude. Femora somewhat widened, edentate. Metafemur dorsally with sparse rows of silvery scales; with almost smooth dorsoposterior edge; subapically with stridulatory patch. Metatibia apically with uncus, without premucro. Abdominal ventrite 5 with dense erect setae. Aedeagus (Fig. 100b) apically rounded, subglabrous; complex transfer-apparatus containing flagellum slightly longer than body; ductus ejaculatorius basally swollen, with indistinct bulbus. **Intraspecific variation**. Length 2.45–3.05 mm. Female rostrum slender, apical 2/3 dorsally subglabrous, punctate, sublaterally with furrow containing row of setae. Female abdominal ventrite 5 with subrecumbent setae.

##### Material examined.

Holotype (SMNK): ARC1853 (EMBL # HE616130), PAPUA NEW GUINEA, Eastern Highlands Prov., Okapa, Kimiagomo village, Hamegoya, S06°25.727', E145°35.455', S06°25.117', E145°35.225', 1891–2131 m, 18-III-2010. Paratypes (NAIC, SMNK, ZSM): PAPUA NEW GUINEA, Eastern Highlands Prov.: 1 ex, ARC1854 (EMBL # HE616131), same data as holotype; 2 exx, ARC1855 (EMBL # HE616132), ARC1856 (EMBL # HE616133), Okapa, Kimiagomo village, Afiyaleto, S06°25.593', E145°34.862', S06°25.212', E145°35.498', 1911 m, 18-III-2010; 2 exx, ARC1882 (EMBL # HE616159), ARC1883 (EMBL # HE616160), Okapa, Konafi to Isimomo, S06°25.593', E145°34.862', S06°25.003', E145°34.527', 1911–2131 m, 18-III-2010.

##### Distribution.

Eastern Highlands Prov. (Okapa). Elevation: 1911 m.

##### Biology.

Beaten from foliage of montane forests.

##### Etymology.

This species is dedicated to the people of Papua New Guinea. The epithet is based on the family name Wari, found on page 356 of the Papua New Guinea Telephone Directory of 2010 and treated in genitive plural.

##### Notes.

*Trigonopterus wariorum* Riedel, sp. n. was coded as “*Trigonopterus* sp. 217” by Tänzler et al. (2012).

#### 101. Trigonopterus zygops

Riedel sp. n.

urn:lsid:zoobank.org:act:ECEFF208-6A4F-4F07-ACAD-BC5E5840C169

http://species-id.net/wiki/Trigonopterus_zygops

##### Diagnostic description.

**Figure 101.**
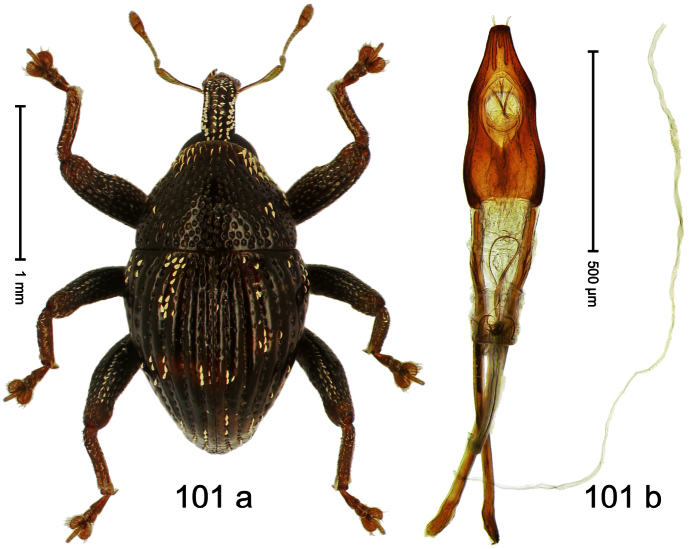
*Trigonopterus zygops* Riedel, sp. n., holotype; (**a**) Habitus (**b**) Aedeagus.

Holotype, male (Fig. 101a). Length 2.06 mm. Color of antenna, tarsi, tibiae, and apex of femora ferruginous; head and pronotum black; elytra black, with ferruginous patches near base of intervals 3–4, transverse irregular band near middle and indistinct patches near apex. Body subovate; with weak constriction between pronotum and elytron; in profile with distinct constriction. Eyes large. Rostrum with median ridge and pair of submedian ridges, lateral furrow with row of overlapping almond-shaped white scales. Pronotum densely punctate, subapically squamose. Elytra with striae deeply impressed, intervals costate, sparsely squamose. Femora ventrally weakly dentate. Tibial uncus simple, curved. Metafemur subapically with stridulatory patch. Aedeagus (Fig. 101b) with sides of body bisinuate, converging; apex extended, pointed, curved ventrad; orifice well-defined, ovate; transfer apparatus flagelliform, subequal to body of aedeagus; ductus ejaculatorius basally swollen, without bulbus. **Intraspecific variation**. Length 1.68–2.16 mm. Ferruginous patches on elytra more or less numerous, in some specimens absent. Female rostrum dorsally subglabrous, punctate, with longitudinal ridges, only basally with scales.

##### Material examined.

Holotype (MZB): ARC0516 (EMBL # FN429222), WEST NEW GUINEA, Jayapura Reg., Cyclops Mts, Sentani, S02°32.031', E140°30.412', 710 m, 02-XII-2007, sifted. Paratypes (ARC, SMNK, ZSM): WEST NEW GUINEA, Jayapura Reg., Cyclops Mts, Sentani: 3 exx, ARC0517 (EMBL # FN429223), ARC0687 (EMBL # FN429332), ARC0688 (EMBL # FN429333), same data as holotype; 4 exx, ARC0533 (EMBL # FN429239), ARC0615 (EMBL # HE615336), ARC0617 (EMBL # FN429274), Doyo, S02°32.478', E140°28.835', 365 m, 27-XI-2007, sifted; 8 exx, ARC0618 (EMBL # FN429275), ARC0619 (EMBL # FN429276), ARC0620 (EMBL # FN429277), Sentani, S02°31.594', E140°30.407', 1065 m, 21-XI-2007, sifted; 2 exx, ARC0643 (EMBL # HE615355), ARC0644 (EMBL # FN429292), Sentani, S02°32.221', E140°30.526', 575 m, 19-XI-2007, sifted; 1 ex, ARC0648 (EMBL # FN429296), Sentani, S02°32.291', E140°30.505', 515 m, 19-XI-2007, sifted; 5 exx, S02°32.031', E140°30.412', 710 m, 02-XII-2007, sifted; 3 exx, S02°31.912', E140°30.416', 785 m, 02-XII-2007, sifted; 1 ex, ARC0668 (EMBL # FN429315), Sentani, S02°31.603', E140°30.434', 1095 m, 28-XI-2007, sifted; 6 exx, S02°31.683', E140°30.281', 960 m, 21-XI-2007, sifted; 4 exx (1 marked ARC00116), Sentani, 1100 m, 23-XII-2004, sifted; 2 exx, Sentani, 1000 m, 23-XII-2004, sifted; 4 exx, Sentani, S02°32.221', E140°30.526', 575 m, 19-XI-2007, sifted; 3 exx, Sentani, S02°31.776', E140°30.215', 945 m, 21-XI-2007, sifted.

##### Distribution.

Jayapura Reg. (Cyclops Mts). Elevation: 365–1095 m.

##### Biology.

Sifted from leaf litter in primary forest.

##### Etymology.

This epithet is a noun in apposition and refers to the generic name *Zygops* Schoenherr, a group with similar large eyes.

##### Notes.

*Trigonopterus zygops* Riedel, sp. n. was coded as “*Trigonopterus* sp. 48” by Riedel et al. (2010) and Tänzler et al. (2012), respectively “*Trigonopterus* spav” in the EMBL/GenBank/DDBJ databases. The holotype and one paratype exhibit a relatively high divergence (5–13% uncorrected p-distance) from the other specimens. So far, we could not find other indications that they represent a cryptic species different from the remainder of paratypes, but the possibility cannot be ruled out.

#### Overview of the species-groups of *Trigonopterus* in New Guinea

The following catalogue and key to species groups is intended as a provisional aid to recognize major species groups of *Trigonopterus*. It is neither comprehensive nor will it be free of mistakes. Results of a preliminary molecular phylogenetic analysis are incorporated to ensure that mainly monophyletic groups are defined. However, additional data and further analyses are required to arrive at a stable hypothesis of the infrageneric classification of *Trigonopterus*.

#### Provisional key to species groups of *Trigonopterus* in New Guinea

**Table:** 

1	Eyes divided into dorsal and ventral portions by deep incision of posterior margin. Edaphic habitat	*Trigonopterus scissops*-group
–	Outline of eyes without deep incision of posterior margin	2
2 (1)	Metafemur subapically without stridulatory patch	3
–	Metafemur subapically with stridulatory patch	11
3 (2)	Aedeagus usually with asymmetrical tip; ventral rim of basal orifice with protruding rim; transfer processes in repose curved basad. Edaphic habitat	subgenus *Mimidotasia* Voss
–	Aedeagus with tip usually symmetrical; transfer processes in repose directed apicad	4
4 (3)	Edaphic species	5
–	Species found on foliage	8
5 (4)	Metafemur with denticulate dorsoposterior edge	*Trigonopterus basalis*-group
–	Metafemur with simple dorsoposterior edge	6
6 (5)	Rostrum at middle with protuberance and at epistome with dorsoposteriad directed horn	*Trigonopterus dentirostris*-group
–	Rostrum dorsally simple, at most with weak median ridge	7
7 (6)	Body small, 1.13–1.59 mm; densely punctate, with sparse minute setae	subgenus *Microgymnapterus* Voss
–	Body polished, with or without scales; 2.09–2.48 mm	*Trigonopterus curtus*-group
8 (4)	Eyes in thanatosis largely covered by pronotum	9
–	Eyes medially approximate, in thanatosis partly exposed	10
9 (8)	Rostrum dorsally weakly sculptured; with two pairs of longitudinal furrows containing sparse mesad directed scales or setae. Disk of pronotum subglabrous. Dorsal edge of metafemur basally contiguous with elytron during thanatosis	*Trigonopterus politus*-group
–	Rostrum dorsally at base densely squamose. Disk of pronotum punctate. Metafemur distant from elytron during thanatosis	*Trigonopterus oblongus*-group
10 (8)	Pronotum and elytra evenly convex. Dorsal edge of metafemur at least basally contiguous with elytron during thanatosis	*Trigonopterus vanus*-group
–	With weak constriction between pronotum and elytra. Metafemur distant from elytron during thanatosis	*Trigonopterus nasutus*-group
11 (2)	Edaphic species	12
–	Species found on foliage	18
12 (11)	Rostrum basally above eyes with pair of protrusions. Tarsomere 3 asymmetrical, with anterior lobe much larger than posterior lobe	*Trigonopterus ptolycoides*-group
–	Rostrum basally above eyes without pair of protrusions. Tarsomere 3 symmetrical	13
13 (12)	Body elongate, with distinct constriction between pronotum and elytron. Tarsomere 3 small, hardly larger than tarsomere 2	*Trigonopterus strombosceroides*-group
–	Body more compact. Tarsomere 3 distinctly larger than tarsomere 2	14
14 (13)	Pronotum with preapical constriction (indistinct in *Trigonopterus hitoloorum* Riedel, sp. n.)	15
–	Pronotum without preapical constriction	16
15 (14)	Rostrum medially costate, at least basally. Femora edentate. Pronotum apically extended over head, or with distinct preapical constriction	*Trigonopterus sulcatus*-group
–	Rostrum coarsely punctate, with median wrinkle. Femora dentate. Pronotum with preapical constriction	*Trigonopterus nothofagorum*-group
16 (14)	Eyes large, exposed in thanatosis. Rostrum dorsally densely squamose. Sides of aedeagus converging to pointed apex	part of *Trigonopterus zygops*-group
–	Eyes smaller. Rostrum at most sparsely squamose. Shape of aedeagus different	17
17 (15)	Male rostrum at epistome with horn. Body roundish. Pronotum basally angulate	*Trigonopterus rhinoceros*-group
–	Male rostrum at epistome without horn	“*Trigonopterus edaphus*-group”
18 (11)	Habitus elongate	19
–	Habitus subovate or subrhomboid	20
19 (18)	Femora edentate	*Trigonopterus honestus*-group
–	Femora dentate	*Trigonopterus dilaticollis*-group
20 (18)	Eyes large, half-exposed in thanatosis	21
–	Eyes smaller, largely concealed in thanatosis	22
21 (20)	Legs relatively long. Metatibial uncus markedly hook-shaped. Species of mid-montane forests	part of *Trigonopterus zygops*-group
–	Legs of normal length. Metatibial uncus not markedly extended. Species of upper montane forests and subalpine vegetation	*Trigonopterus ascendens*-group
22 (20)	All femora edentate	23
–	At least one pair of femora dentate	25
23 (22)	Rostrum dorsally relatively flat, with rows of punctures or shallow furrows. Thoracic venter and abdominal ventrites 1-2 with long erect setae	*Trigonopterus gonatocerus*-group
–	Rostrum with distinct ridges or swollen in basal half. Thoracic venter and abdominal ventrites 1-2 sparsely setose with subrecumbent setae or subglabrous	24
24 (23)	Rostrum dorsally swollen at base; in profile dorsal contour sinuate	part of *Trigonopterus vandekampi*-group
–	Rostrum in basal half with distinct median ridge and pair of submedian ridges. in profile evenly convex	*Trigonopterus montivagus*-group
25 (22)	Pronotum subglabrous, sparsely punctate. Meso- and metafemur ventrally with denticulate or serrate ridges and knobs	*Trigonopterus balimensis*-group
–	Pronotum densely punctate. Meso- and metafemur ventrally with simple tooth	26
26 (25)	Pronotum coarsely punctate, elytron subglabrous	*Trigonopterus illex*-group
–	Difference of sculpture between pronotum and elytron less evident	part of *Trigonopterus vandekampi*-group

#### Provisional catalogue of species groups of *Trigonopterus* in New Guinea

**subgenus *Microgymnapterus* Voss**: *Trigonopterus micros* Riedel

**subgenus *Mimidotasia* Voss**: *Trigonopterus aeneipennis* Riedel, sp. n., *Trigonopterus aeneus* Riedel, sp. n., *Trigonopterus inflatus* Riedel, sp. n., *Trigonopterus myops* Riedel, sp. n., *Trigonopterus parvulus* Riedel, sp. n., *Trigonopterus striatus* Riedel, sp. n., *Trigonopterus oblitus* Riedel, *Trigonopterus vossi* Riedel.

***Trigonopterus ascendens*-group**: *Trigonopterus ascendens* Riedel, sp. n.

***Trigonopterus balimensis*-group**: *Trigonopterus balimensis* Riedel, sp. n., *Trigonopterus crassicornis* Riedel, sp. n.

***Trigonopterus basalis*-group**: *Trigonopterus agathis* Riedel, sp. n., *Trigonopterus amplipennis* Riedel, sp. n., *Trigonopterus basalis* Riedel, sp. n., *Trigonopterus dromedarius* Riedel, sp. n., *Trigonopterus ixodiformis* Riedel, sp. n., *Trigonopterus parumsquamosus* Riedel, sp. n., *Trigonopterus plicicollis* Riedel, sp. n., *Trigonopterus signicollis* Riedel, sp. n., *Trigonopterus taenzleri* Riedel, sp. n., *Trigonopterus talpa* Riedel, sp. n.

***Trigonopterus curtus*-group**: *Trigonopterus basimaculatus* (Voss), *Trigonopterus curtus* (Voss), *Trigonopterus flavomaculatus* (Voss), *Trigonopterus simulans* Riedel, sp. n., *Trigonopterus subglabratus* Riedel, sp. n.

***Trigonopterus dentirostris*-group**: *Trigonopterus dentirostris* Riedel, sp. n.

***Trigonopterus dilaticollis*-group**: *Trigonopterus apicalis* Riedel, sp. n., *Trigonopterus difficilis* (Faust), *Trigonopterus dilaticollis* (Faust), *Trigonopterus morokensis* (Faust)

***Trigonopterus*** **“*edaphus*-group”**: assemblage of probably unrelated species of uncertain affinities: *Trigonopterus discoidalis* Riedel, sp. n., *Trigonopterus echinus* Riedel, sp. n., *Trigonopterus edaphus* Riedel, sp. n., *Trigonopterus kurulu* Riedel, sp. n., *Trigonopterus oviformis* Riedel, sp. n., *Trigonopterus sordidus* Riedel, sp. n.

***Trigonopterus gonatoceros*-group**: *Trigonopterus ferrugineus* Riedel, sp. n., *Trigonopterus gonatoceros* Riedel, sp. n., *Trigonopterus rufibasis* Riedel, sp. n., *Trigonopterus violaceus* Riedel, sp. n.

***Trigonopterus honestus*-group**: *Trigonopterus angustus* Riedel, sp. n., *Trigonopterus honestus* (Pascoe), *Trigonopterus irregularis* Riedel, sp. n., *Trigonopterus lineellus* Riedel, sp. n., *Trigonopterus rubiginosus* Riedel, sp. n.

***Trigonopterus illex*-group**: *Trigonopterus densatus* (Faust), *Trigonopterus illex* (Faust)

***Trigonopterus montivagus*-group**: *Trigonopterus ampliatus* (Pascoe), *Trigonopterus kanawiorum* Riedel, sp. n., *Trigonopterus mimicus* Riedel, sp. n., *Trigonopterus montivagus* Riedel, sp. n., *Trigonopterus phoenix* Riedel, sp. n., *Trigonopterus ragaorum* Riedel, sp. n., *Trigonopterus rubripennis* Riedel, sp. n., *Trigonopterus strigatus* Riedel, sp. n., *Trigonopterus wariorum* Riedel, sp. n.

***Trigonopterus nasutus*-group**: *Trigonopterus augur* Riedel, sp. n., *Trigonopterus cribratus* (Faust), *Trigonopterus ephippiatus* (Faust), *Trigonopterus femoralis* (Faust), *Trigonopterus gibbirostris* (Faust), *Trigonopterus helios* Riedel, sp. n., *Trigonopterus illitus* (Faust), *Trigonopterus insularis* Riedel, sp. n., *Trigonopterus impar* (Faust), *Trigonopterus maculatus* Riedel, sp. n., *Trigonopterus melas* (Faust), *Trigonopterus moreaorum* Riedel, sp. n., *Trigonopterus nasutus* (Pascoe), *Trigonopterus pseudonasutus* Riedel, sp. n., *Trigonopterus salubris* (Faust), *Trigonopterus sellatus* (Faust), *Trigonopterus soiorum* Riedel, sp. n., *Trigonopterus tialeorum* Riedel, sp. n.

***Trigonopterus nothofagorum*-group**: *Trigonopterus nothofagorum* Riedel, sp. n.

***Trigonopterus oblongus*-group**: *Trigonopterus cuneatus* (Faust), *Trigonopterus oblongus* (Pascoe), *Trigonopterus similis* (Heller)

***Trigonopterus politus*-group**: *Trigonopterus cuneipennis* Riedel, sp. n., *Trigonopterus durus* Riedel, sp. n., *Trigonopterus katayoi* Riedel, sp. n., *Trigonopterus obnixus* (Faust), *Trigonopterus politoides* Riedel, sp. n., *Trigonopterus politus* (Faust), *Trigonopterus viridescens* Riedel, sp. n.

***Trigonopterus ptolycoides*-group**: *Trigonopterus ptolycoides* Riedel, sp. n.

***Trigonopterus rhinoceros*-group**: *Trigonopterus rhinoceros* Riedel, sp. n.

***Trigonopterus scissops*-group**: *Trigonopterus constrictus* Riedel, sp. n., *Trigonopterus scissops* Riedel, sp. n.

***Trigonopterus strombosceroides*-group**: *Trigonopterus costicollis* Riedel, sp. n., *Trigonopterus strombosceroides* Riedel, sp. n.

***Trigonopterus sulcatus*-group**: *Trigonopterus angulatus* Riedel, sp. n., *Trigonopterus costatus* Riedel, sp. n., *Trigonopterus hitoloorum* Riedel, sp. n., *Trigonopterus lineatus* Riedel, sp. n., *Trigonopterus scabrosus* Riedel, sp. n., *Trigonopterus sulcatus* Riedel, sp. n., *Trigonopterus tridentatus* Riedel, sp. n., *Trigonopterus verrucosus* Riedel, sp. n.

***Trigonopterus vandekampi*-group**: *Trigonopterus armatus* Riedel, sp. n., *Trigonopterus monticola* Riedel, sp. n., *Trigonopterus pulchellus* (Pascoe), *Trigonopterus vandekampi* Riedel

***Trigonopterus vanus*-group**: *Trigonopterus agilis* Riedel, sp. n., *Trigonopterus glaber* Riedel, sp. n., *Trigonopterus granum* Riedel, sp. n., *Trigonopterus imitatus* Riedel, sp. n., *Trigonopterus neglectus* (Faust), *Trigonopterus proximus* (Voss), *Trigonopterus pseudogranum* Riedel, sp. n., *Trigonopterus sejunctus* (Faust), *Trigonopterus vanus* (Faust), *Trigonopterus velaris* Riedel, sp. n.

***Trigonopterus zygops*-group**: *Trigonopterus ancoruncus* Riedel, sp. n., *Trigonopterus euops* Riedel, sp. n. *Trigonopterus lekiorum* Riedel, sp. n., *Trigonopterus scharfi* Riedel, sp. n., *Trigonopterus squamirostris* Riedel, sp. n., *Trigonopterus variabilis* Riedel, sp. n., *Trigonopterus zygops* Riedel, sp. n.

## Discussion

For the last few decades it has been common practice for taxonomists maintaining a good reputation to revise monophyletic groups with all the known species, to prepare elaborate descriptions bearing in mind the potential value of every tiny character for a phylogenetic analysis, and to make great efforts on the preparation of elaborate identification keys based on these characters. As a consequence, hyperdiverse taxa such as the genus *Trigonopterus* are usually avoided, simply because it appears impossible to get this task completed during a lifetime. Such considerations are a lesser issue for a minority of taxonomists with a lower quality-standard, the so called “mass-describers”, usually publishing their works in journals without peer-review. Often, they do not refrain from proposing new names based on specimens without sufficient diagnostic characters, such as unique females, relying on the community to later sort out the resulting identification problems. This exacerbates the deterrence of such “difficult” taxa which are then prime examples and causes of a “taxonomic impediment”, in this case not only an impediment to the end-users of taxonomy, but also to the taxonomists themselves (Ebach et al. 2011, Godfray 2002). In fact, at this stage taxonomic information becomes rather a burden to science than a useful tool. We believe that technologies developed within the past decade enable us to stop this vicious circle. Two components are of fundamental importance, i.e. online wiki databases and molecular systematics (Riedel et al. 2013).

Online wiki databases such as the Species-Id portal (http://species-id.net/wiki/Main_Page ) are not recognized as means of publication by the International Code of Zoological Nomenclature (1999), so their significance needs some explanation. Journals such as “ZooKeys” make a new name available with a traditional paper publication, simultaneously creating a wiki with the same content. This wiki can be updated later anytime with additional data, be it an elaborate 3D-model or a “quantum contribution” (Maddison et al. 2012) such as fixing a typo of the original description or adding a simple collecting record. At the time the species becomes formally named there is no urgency to provide the description with all possible data. It should contain a reasonable basis, so that its diagnosis is guaranteed. We expect that most users will later rather consult the online working description, gradually being supplemented with additional data. Thus, the formal species description is like a healthy newborn which is expected to grow into an adult with the help of its environment. In the case of *Trigonopterus*, characters such as the functional morphology of thanatosis or the morphology of the metendosternite, surely of great interest but of little diagnostic value can be added at a later stage without compromising their visibility. This approach does in fact bundle useful features of numerous other initiatives such as the Encyclopedia of Life (eol.org).

The impact of molecular systematics on species-descriptions is twofold and can be divided into reconstruction of species relationships and attempts to diagnose species. But let us start one step earlier, with the advent of phylogenetic systematics (Hennig 1966) and phenetics (Sneath and Sokal 1973). Both had a profound but little-noticed effect on the preparation of species descriptions. Since more and more taxonomic revisions incorporated phylogenetic analyses, it was attempted to maximize the number of informative characters. Thus, even characters of little value for species diagnosis were included in the descriptions. Another consequence was that species descriptions within a study were sought to be standardized, best illustrated by the program Delta (Partridge et al. 1993). Negative character states (i.e. the absence of a character) were often explicitly stated. Often enough, all this time-consuming procedure did not increase the usability of descriptions for the purpose of diagnosis, but rather inflated them. And after all, standardization among different authors was never achieved not to mention failure to introduce an urgently needed minimum standard.

Although in some taxa, phylogenies based on morphological data are still needed, in recent years the trend clearly goes towards purely molecular phylogenies. In the case of *Trigonopterus* we feel that our molecular data set is strong enough to produce a stable phylogeny without morphological characters included. So, for us it is time to ask – do we really need to describe every character with the hope that this might be a valuable addition to our character-matrix? For us, the answer is “no”! With changing needs on a species description, taxonomists should reflect if they want to carry on like during the past decades, or if it is time to adjust procedures and streamline descriptions to the purpose of diagnosis.

The potential of using a standard DNA marker for species identification, also known as “DNA barcoding”, was recognized almost ten years ago (Hebert et al. 2003). Despite some initial criticism it proved to be a powerful tool. In many taxa, the *cox1* sequence will pinpoint the correct species without additional information. In others it may not delineate species unambiguously (Hendrich et al. 2010), but even then it is possible to safely pinpoint a group of e.g. 5–10 species. For many taxa, be it nematodes, moss mites or rove beetles, a non-expert would hardly achieve this within reasonable time using traditional keys. After all, in combination with a few morphological characters the species can be safely identified in most cases. One of the great advantages of sequence data is that they can be databased, searched and accessed anytime from anywhere. The situation with type specimens is quite different: often they are not accessible, or if so, it is very time-consuming both for museum curators and active researchers to send them around the globe. Often enough, they give the only clue what species an insufficient description is referring to, or if the species is placed in the correct genus at all. Such issues could be much faster solved using “DNA barcodes”. We strongly believe that the ICZN should make the publication of genetic data obligatory for the description of new extant species. Until such a decision is made, the contest between descriptions containing DNA barcodes and the ones without may give an answer of what is really needed. The combination of short expert morphological descriptions provided with a few high-resolution photographs and DNA-sequences appears to us as the way to proceed. We predict that similar works are to be expected in the near future on various taxa of hyperdiverse organisms.

## Plates
